# Anti-RAFLS Triterpenoids and Hepatoprotective Lignans From the Leaves of Tujia Ethnomedicine *Kadsura heteroclita* (Xuetong)

**DOI:** 10.3389/fchem.2022.878811

**Published:** 2022-05-10

**Authors:** Mengyun Wang, Sai Jiang, Nusrat Hussain, Salman Zafar, Qingling Xie, Feibing Huang, Linxi Mao, Bin Li, Yuqing Jian, Wei Wang

**Affiliations:** ^1^ TCM and Ethnomedicine Innovation & Development International Laboratory, Innovative Material Medical Research Institute, School of Pharmacy, Hunan University of Chinese Medicine, Changsha, China; ^2^ Department of Chemistry, University of Baltistan Skardu, Skardu, Pakistan; ^3^ Institute of Chemical Sciences, University of Peshawar, Peshawar, Pakistan

**Keywords:** *Kadsura heteroclita*, triterpenoids, lignans, anti-RAFLS activity, hepatoprotective activity

## Abstract

A pair of 3,4-*seco*-cycloartane triterpenoid isomers with a rare peroxy bridge, namely, xuetonins A and B (**1** and **2**), four new lignans xuetonlignans A–D (**3**–**6**), a new sesquiterpene xuetonpene (**7**), and a new natural product xuetonin C (**8**), along with 43 known compounds, were obtained from the leaves of Tujia ethnomedicine, *Kadsura heteroclita*. Their structures and configurations were determined with the help of a combination of 1D- and 2D-NMR, HRESIMS spectra, electronic circular dichroism (ECD), and X-ray diffraction data. Compounds **2**, **10**, **13**–**15**, and **17**–**19** showed moderate-to-potent activity against rheumatoid arthritis fibroblast-like synoviocytes (RAFLS) with IC_50_ values of 19.81 ± 0.26, 12.73 ± 0.29, 5.70 ± 0.24, 9.25 ± 0.79, 5.66 ± 0.52, 11.91 ± 0.44, 13.22 ± 0.27, and 15.94 ± 0.36 μM, respectively. Furthermore, compounds **22**, **25**, and **31** exhibited significant hepatoprotective effects against *N*-acetyl-*p*-aminophenol (APAP)–induced toxicity in HepG2 cells at 10 μM, and the cell viability increased by 12.93, 25.23, and 13.91%, respectively, compared with that in the model group (cf. bicyclol, 12.60%).

## 1 Introduction

*Kadsura heteroclita* (Roxb.) Craib (Schizandraceae) is an important ingredient of traditional Chinese medicine (TCM), which was widely distributed in the southwest part of China (Cao et al., 2019b). The plant is locally called “Xuetong” in Tujia ethnomedicine to treat rheumatoid arthritis (RA) and hepatitis (Cao et al., 2019a; Cao et al., 2019c; Wang et al., 2020). Previous phytochemical investigations have indicated that the main bioactive chemical constituents of *K. heteroclita* are dibenzocyclooctadienes and spirobenzofuranoid dibenzocyclooctadienes lignans, lanostanes, and cycloartane triterpenoids exhibiting various bioactivities such as anti-RA, anti-inflammation and analgesic, hepatoprotection, anti-HIV, anticancer, and anti-HBV (Liu Y. B et al., 2018; Wang et al., 2020). Previous reports from our research group on the stem of *K. heteroclita* describe the isolation of a series of triterpenoids and lignans (Wang et al., 2006b; Cao et al., 2019b).

**FIGURE 1:**
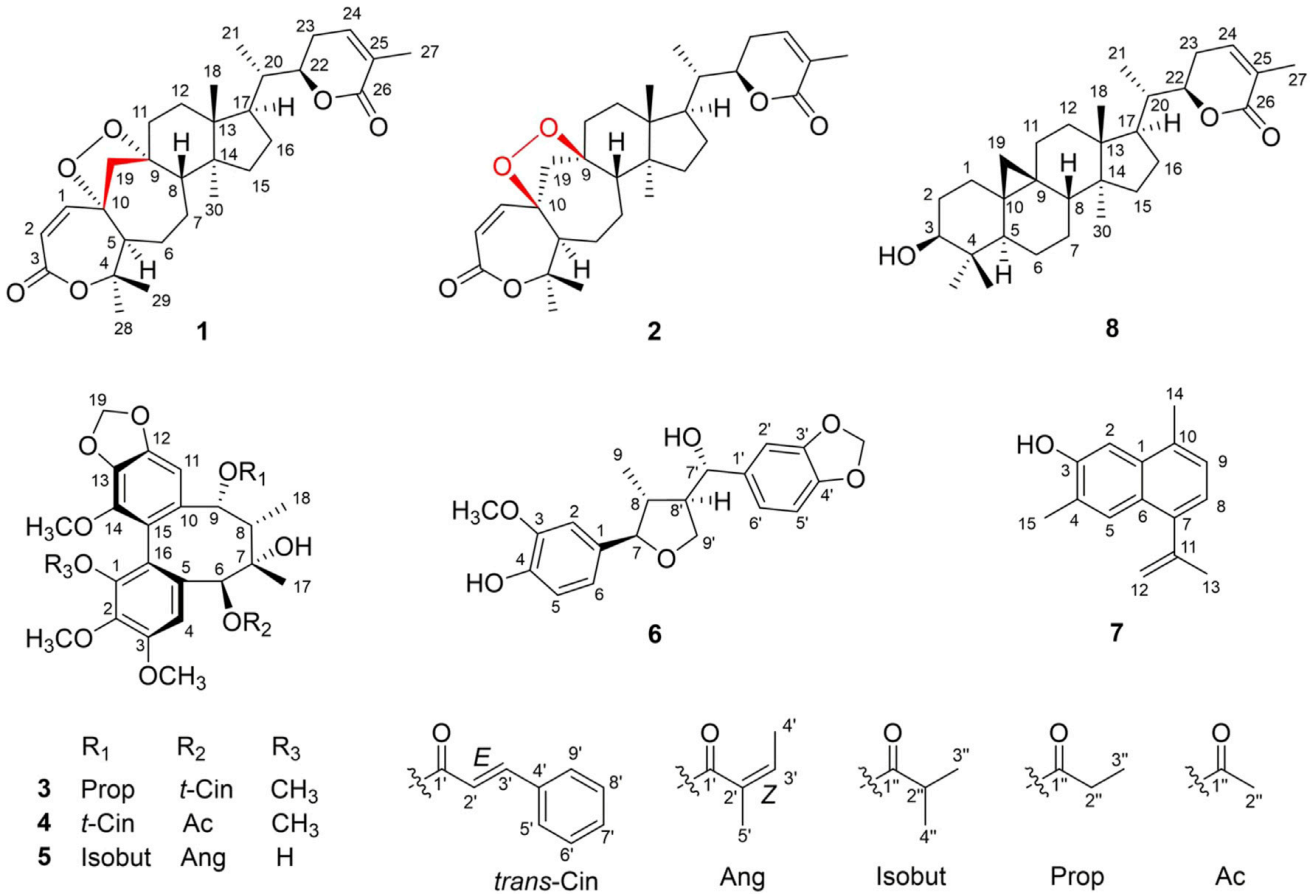
Structures of new compounds **(1–7)** and a natural product **(8)**.

The stem of the plant has always been used for medicinal purposes. Moreover, studies have also been carried out on its chemical constituents and pharmacological potential over the years (Wang et al., 2020). However, there is no specific literature on the phytochemistry and bioactivities of the leaves of *K. heteroclita*. Thus, in order to comprehend and understand the importance of the plant, the leaves of the plant were studied in this research endeavor, leading to the isolation of seven new compounds (**1**–**7**), one natural product (**8**) (Figure 1), and 43 known compounds*.* Furthermore, these secondary metabolites were tested for their anti-RAFLS effect and hepatoprotective potential. Compounds **2**, **10**, **13**–**15**, and **17**–**19** exhibited a moderate-to-potent anti-RAFLS activity. Furthermore, compounds **22**, **25**, and **31** exhibited significant hepatoprotective effects against APAP-induced toxicity in HepG2 cells. Herein, the isolation, identification, bioactivity evaluation, and molecular docking studies of these isolated compounds are presented.

## 2 Materials and Methods

### 2.1 General Experimental Procedures

Optical rotations were recorded on a Perkin–Elmer 341-MC digital polarimeter at room temperature. A TU-1900 spectrophotometer (Shimadzu Europa GmbH, Duisburg, Germany) was used for obtaining the UV/Vis spectrum; IR spectra were scanned using a Hitachi 260–30 spectrometer. A Jasco J-810 circular dichroism spectropolarimeter was used to measure the ECD spectra at room temperature. 1D- and 2D-NMR spectra were obtained on a Bruker ARX-600 spectrometer (Bruker Technology Co., Ltd., Karlsruhe, Germany). The HRESIMS spectra were acquired using the UPLC/xevo G2 Qtof spectrometer (Waters Corporation, Milford, MA, United States). Semi-preparative HPLC was conducted on an Agilent 1,260 liquid chromatography (Santa Clara, CA, United States) with an Agilent C_18_ column (250 mm × 34 mm). Silica gels (80–100 and 300–400 meshes) were obtained from Qingdao Marine Chemical Inc. (Qingdao, China). All analytical-grade solvents were obtained from Shanghai Titan Scientific Co., Ltd., Shanghai, China. HPLC-grade methanol and acetonitrile were purchased from Merck KGaA (Darmstadt, Germany).

### 2.2 Plant Material

The leaves of *Kadsura heteroclita* (Schisandraceae) were collected in Shimen county, Changde city, Hunan province, China, during March 2014 and identified by Prof. Wei Wang, School of Pharmacy, Hunan University of Chinese Medicine. The voucher specimen number (KH-shimen-201403) has been deposited in the School of Pharmacy, Hunan University of Chinese Medicine, Changsha city, Hunan province, P. R. China.

### 2.3 Extraction and Isolation

The air-dried leaves of *K. heteroclita* (8 kg) were powdered and extracted thrice with 90% EtOH (24.0 L) for 1.5 h each using ultrasonic extraction. Then, all the extract solvents were evaporated under reflux condition to obtain the crude EtOH extract (750.1 g). The crude extract was then suspended in H_2_O (3.2 L) and successively partitioned with dichloromethane (DCM) and ethyl acetate (EtOAc) to yield DCM-soluble (70.1 g) and EtOAc-soluble (55.9 g) fractions, respectively.

The DCM fraction was then subjected to silica gel column chromatography (CC), which was eluted with petroleum ether (PE)–ethyl acetate (EA) (1:0, 100:1, 50:1, 20:1, 10:1, 5:1, 2:1, 1:1, and 0:1 gradient systems) to obtain six fractions (Fr. A–Fr. F). Fraction B (9.7 g) was further subjected to CC over silica gel, eluting with PE–EA (1:0–0:1) to yield four sub-fractions (Fr. B1–Fr. B4). Fr. B2 (2.6 g) after successive separation on a silica gel column, a Sephadex LH-20 column, and preparative TLC afforded the pure compounds **16** (33.8 mg), **28** (1.5 mg), **40** (80.2 mg), **45** (45.7 mg), and **48** (1.0 mg). Fr. B3 (3.2 g) was repeatedly purified on a silica gel column and a Sephadex LH-20 column to obtain the pure compounds **7** (2.1 mg), **13** (5.5 mg), **17** (6.1 mg), **18** (4.0 mg), **19** (14.1 mg), and **20** (7.0 mg). Fraction C (12.8 g) was separated on a silica gel CC using PE–EA (1:0–0:1) as elution solvents to afford six fractions (Fr. C1–Fr. C6). Fr. C3 (3.8 g) was subjected to successive separations, and ultimately compounds **4** (3.2 mg, retention time = *t*_R_ 26.21 min), **21** (39.6 mg, *t*_R_ 27.71 min), **23** (4.1 mg, *t*_R_ 29.66 min**)**, and **25** (8.6 mg, *t*_R_ 39.28 min) were separated by semi-preparative HPLC with 72% MeOH/H_2_O at a flow rate of 2 ml/min. Fr. C4 (4.1 g) after successive chromatography on silica gel and a Sephadex LH-20 column yielded pure compounds **22** (2.9 mg, *t*_R_ 15.74 min), **5** (1.8 mg, *t*_R_ 18.10 min), **24** (5.5 mg, *t*_R_ 21.01 min), and **3** (6.5 mg, *t*_R_ 25.03 min) on semi-prep HPLC with the 65% ACN/H_2_O solvent system at a flow rate of 2 ml/min. Furthermore, compound **26** (12.1 mg) was also separated from the same sub-fraction on a silica gel CC with PE–EA (in a gradient manner from 1:0 to 0:1). Fraction D (9.6 g) was then isolated through a silica gel CC eluted with PE–EA (from 1:0 to 0:1) to obtain four sub-fractions (Fr. D1–Fr. D4). Fr. D2 (2.3 g) was further separated on a silica gel CC eluted with PE–EA (1:0–0:1) to afford eight sub-fractions (Fr. D2.1–Fr. D2.8). Compounds **30** (3.9 mg, *t*_R_ 18.13 min), **31** (1.0 mg, *t*_R_ 18.96 min), **6** (1.7 mg, *t*_R_ 21.60 min), and **27** (2.0 mg, *t*_R_ 22.22 min) were isolated from Fr. D2.3 (157.2 mg) by semi-prep HPLC with 65% MeOH/H_2_O. Compound **12** (1.6 mg, *t*_R_ 27.39 min) was purified from Fr. D2.4 (135.3 mg) by semi-prep HPLC with ACN-H_2_O (55: 45). Compounds **49** (1.6 mg, *t*_R_ 8.84 min) and **46** (1.3 mg, *t*_R_ 9.82 min) were obtained from Fr. D2.5 (95.3 mg) by semi-prep HPLC with ACN-H_2_O (45: 55). Compounds **1** (5.8 mg) and **2** (6.1 mg) were purified from Fr. D2.5 (216.7 mg) by CC over silica gel eluted with hexane–acetone (from 9:1 to 7:3). Fr. D3 (3.7 g) yielded compounds **15** (3.5 mg), **29** (100.4 mg), and **41** (50.0 mg) by a series of silica gel CC, Sephadex LH-20 CC, and preparative TLC. Fraction E (16.9 g) was further separated on a silica gel CC eluted with DCM–MeOH (from 1:0 to 0:1) to afford six fractions (Fr. E1–Fr. E6). Fr. E3 (2.6 g) was purified by a silica gel column, a Sephadex LH-20 column, and preparative TLC method to obtain pure compounds **9** (4.1 mg), **10** (3.0 mg), **11** (11.0 mg), **38** (1.0 mg), and **39** (37.7 mg). Fr. E4 (5.7 g) was then subjected to successive silica gel CC, Sephadex LH-20 CC, ODS CC, and preparative TLC to obtain compounds **14** (30.0 mg), **32** (3.6 mg), **33** (7.3 mg), **34** (1.2 mg), **35** (4.7 mg), **36** (2.0 mg), **37** (1.2 mg), **42** (8.1 mg), **43** (3.3 mg), **44** (11.1 mg), and **47** (1.0 mg).

The EA fraction was then separated through silica gel CC using a gradient system of PE/EA (1:0, 50:1, 20:1, 10:1, 5:1, 2:1, 1:1, and 0:1) for elution to yield 10 fractions (Fr. A–Fr. J). Fraction C (860.8 mg) was isolated through a series of CC experiments over silica gel by gradient elution of PE–EA (1:0–0:1) to obtain five sub-fractions (Fr. C1–Fr. C5). Compound **8** (13.8 mg) was obtained from Fr. C2 (286.4 mg) and Fr. C3 (101.2 mg), which was subjected to silica gel CC using DCM/MeOH (from 1:0 to 0:1). Fraction F (2.4 g) was isolated through a silica gel CC eluted with PE–EA (from 1:0 to 0:1) to afford compound **51** (21.5 mg). Fraction J (44.33 g) after successive chromatography on a silica gel column using a gradient elution of DCM–MeOH (from 1:0 to 0:1) afforded three sub-fractions (Fr. J1–Fr. J3). Fr. J3 (40.3 g) was eluted on ODS CC with a gradient solvent system of MeOH–H_2_O (0:1–1:0) to yield compound **50** (10.0 g).

#### 2.3.1 Xuetonin A

White amorphous powder; ${\left[\alpha \right]}_{D}^{24}$ +44.3° (*c* = 0.1, CH_2_Cl_2_); UV (CH_2_Cl_2_) *λ*_max_ (log *ε*): 209 (3.21) nm; IR *ν*_max_: 2,919, 1710, 1,686, 1,396, 1,379, 1,123, and 729 cm^−1^; (+) HRESIMS: *m/z* 499.3066 [M + H]^+^, calcd for C_30_H_43_O_6_, 499.3060; ^1^H and ^13^C NMR data: see Table 1.

**TABLE 1:** ^1^H (600 MHz) and ^13^C NMR (150 MHz) data of compounds **1**, **2**, and **8** in CDCl_3_ (*J* in Hz).

NO	1		2		8	
	*δ*_H_	*δ*_C_	*δ*_H_	*δ*_C_	*δ*_H_	*δ*_C_
1	6.22, d (12.6)	146.9	6.24, d (12.6)	146.0	1.87, m	27.6
					1.02, m	
2	5.94, d (12.6)	119.6	5.97, d (12.6)	120.1	1.93, m	28.7
					1.65, m	
3	—	165.4	—	166.2	3.47, t (2.4)	77.2
4	—	82.9	—	84.6	—	39.7
5	2.42, m	49.3	2.45, m	54.8	1.83, m	41.2
6	2.20, m	29.6	2.07, m	27.8	1.73, m	27.2
	1.34, m				1.38, m	
7	2.28, m	25.1	1.70, m	27.3	1.49, m	21.2
	1.59, m		1.58, m		0.78, m	
8	1.83, m	49.5	2.15, m	51.4	1.54, m	48.1
9	—	87.8	—	87.9	—	19.9
10	—	87.0	—	86.7	—	26.6
11	2.12, m	30.8	2.08, m	29.2	1.99, m	26.3
	1.64, m		1.92, m		1.16, m	
12	1.72, m	30.7	1.65, m	31.2	1.63, m	32.9
	1.57, m					
13	—	46.1	—	45.3	—	48.7
14	—	48.9	—	48.8	—	45.9
15	1.32, m	33.9		35.1	1.34, m	35.7
16	1.79, m	26.8	1.79, m	27.0	1.31, m	25.8
	1.41, m		1.45, m		1.12, m	
17	1.59, m	46.5	1.58, m	48.2	1.61, m	48.3
18	0.86, s	14.5	1.00, s	16.3	1.00, s	18.0
19	2.74, d (12.6)	55.1	2.76, d (12.6)	58.9	0.52, d (4.2)	29.9
	2.18, d (12.6)		2.22, d (12.6)		0.36, d (4.2)	
20	2.05, m	39.3	2.04, m	39.2	2.03, m	39.3
21	0.98, d (6.6)	13.7	0.96, d (6.6)	13.3	0.97, d (6.6)	13.2
22	4.46, dt (13.2, 3,6)	80.5	4.45, m	80.5	4.47, dt (13.2, 3,6)	80.8
23	2.37, m	23.6	2.38, m	23.7	2.37, m	23.6
	2.07, m		2.08, m		2.09, m	
24	6.61, d-like (6.6)	139.5	6.59, d-like (6.6)	139.3	6.60, d-like (6.6)	139.6
25	—	128.4	—	128.6	—	128.4
26	—	166.7	—	166.6	—	166.8
27	1.92, s	17.1	1.92, s	17.2	1.91, s	17.2
28	1.42, s	30.2	1.42, s	21.5	0.95, s	26.0
29	1.40, s	21.6	1.40, s	30.9	0.88, s	21.4
30	0.98, s	17.6	0.83, s	18.4	0.89, s	19.6

#### 2.3.2 Xuetonin B

White amorphous powder; ${\left[\alpha \right]}_{D}^{24}$ +46.8° (*c* = 0.1, CH_2_Cl_2_); UV (CH_2_Cl_2_) *λ*_max_ (log *ε*): 209 (3.36) nm; IR *ν*_max_: 2,920, 1710, 1,686, 1,395, 1,123, 828, and 730 cm^−1^; (+) HRESIMS: *m/z* 499.3068 [M + H]^+^, calcd for C_30_H_43_O_6_, 499.3060; ^1^H and ^13^C NMR data: see Table 1.

#### 2.3.3 Xuetonlignan A

White amorphous powder; ${\left[\alpha \right]}_{D}^{24}$ +9.9° (*c* = 0.1, MeOH); UV (MeOH) *λ*_max_ (log *ε*): 218 (3.69) nm; IR *ν*_max_: 3,569, 2,942, 1712, 1,623, 1,464, 1,371, 1,251, 1,161, 1,105, 1,045, and 733 cm^−1^; ECD [*λ*_max_ (Δ*ε*)]: 227 (+1.02), 252 (−1.09) nm; (+) HRESIMS: *m/z* 652.2758 [M + NH_4_]^+^, calcd for C_35_H_38_O_11_NH_4_, 652.2758; ^1^H and ^13^C NMR data: see Tables 2, 3.

**TABLE 2:** ｜^1^H NMR (600 MHz) data of compounds **3–6** in CD_3_OD and **7** in CDCl_3_ (*J* in Hz).

NO	3	4	5	6	7
	*δ*_H_	*δ*_H_	*δ*_H_	*δ*_H_	*δ*_H_
2	—	—	—	6.93, s	7.24, s
4	6.85, s	6.89, s	6.60, s	—	—
5	—	—	—	6.77, m	7.80, s
6	5.71, s	5.65, s	5.59, s	6.77, m	—
7	—	—	—	4.17, m	—
8	2.30, q (7.2)	2.17, q (7.2)	2.19, m	1.75, m	7.03, d (7.2)
9	5.78, s	5.89, s)	5.70, s	0.62, d (6.6)	7.17, d (7.2)
10	—	—	—		—
11	6.63, s	6.60, s	6.51, s		—
12	—	—	—		5.36, s
					5.01, s
13	—	—	—		2.18, s
14	—	—	—		2.58, s
15	—	—	—		2.42, s
17	1.38, s	1.35, s	1.36, s		
18	1.30, d (7.2)	1.27, d (7.2)	1.27, d (6.6)		
OCH_2_O	5.72, d, (1.2)	5.98, s	5.94, d (0.6)	5.93, s	
	5.15, d (1.8)		5.90, d (1.2)		
1-OCH_3_	3.66, s	3.85, s	—		
2-OCH_3_	3.86, s	3.58, s	3.84, s		
3-OCH_3_	3.94, s	3.96, s	3.93, s	3.87, s	
14-OCH_3_	3.58, s	3.41, s	3.76, s		
2′	6.06, d (16.2)	5.97, d (15.6)	—	6.89, s	
3′	6.98, d (16.2)	7.06, d (15.6)	6.00, m	—	
4′	—	—	1.81, m	—	
5′	7.53, m	7.44, m	1.42, m	6.77 (1H, m)	
6′	7.44, m	7.39, m		6.81 (1H, m)	
7′	7.44, m	7.39, m		4.52, d (7.8)	
8′	7.44, m	7.39, m		2.27, m	
9′	7.53 (1H, m)	7.44, m		4.19, m	
				3.98, t (8.4)	
2″	1.97, m	1.62, s	1.93, m		
	1.75, m				
3″	0.84, t (7.8)		0.88, d (6.6)		
4″			0.87, d (7.2)		

**TABLE 3:** ｜^13^C NMR (150 MHz) data of compounds **3–6** in CD_3_OD and **7** in CDCl_3_.

NO	3	4	5	6	7
	*δ*_C_	*δ*_C_	*δ*_C_	*δ*_C_	*δ*_C_
1	141.8	141.9	149.4	133.4	133.0
2	142.5	142.7	136.9	111.1	106.4
3	153.3	153.1	152.4	149.0	152.6
4	112.2	112.4	108.2	147.5	125.5
5	131.5	131.9	131.3	115.9	128.1
6	86.2	86.3	86.7	120.7	126.5
7	75.2	74.9	75.3	90.6	140.1
8	44.5	44.7	44.6	46.0	122.1
9	84.5	83.8	84.7	15.4	126.0
10	134.5	134.6	134.4		131.4
11	103.1	103.5	103.6		145.3
12	150.1	150.2	150.2		115.8
13	136.9	137.1	137.4		25.6
14	152.4	152.3	142.4		19.7
15	121.8	122.1	121.7		16.7
16	123.4	123.6	118.0		
17	29.4	29.7	29.5		
18	17.2	17.1	17.3		
OCH_2_O	102.2	102.6	102.4	102.3	
1-OCH_3_	59.4	59.8			
2-OCH_3_	61.0	60.9	60.9		
3-OCH_3_	56.6	56.5	56.5	56.4	
14-OCH_3_	61.1	60.7	59.6		
1′	166.4	166.9	167.4	139.2	
2′	118.2	118.4	128.5	107.8	
3′	146.0	146.7	140.3	149.2	
4′	135.5	135.5	15.9	148.4	
5′	129.4	129.4	20.3	108.8	
6′	130.0	129.9		121.1	
7′	131.7	131.6		76.7	
8′	130.0	129.9		55.8	
9′	129.4	129.4		71.3	
1″	174.2	171.2	176.8		
2″	27.7	20.3	34.7		
3″	8.8		18.4		
4″			15.9		

#### 2.3.4 Xuetonlignan B

White amorphous powder; ${\left[\alpha \right]}_{D}^{24;}$ +10.8° (*c* = 0.1, MeOH); UV (MeOH) *λ*_max_ (log *ε*): 218 (3.29) nm; IR *ν*_max_: 3,377, 2,944, 2,836, 1715, 1,623, 1,464, 1,371, 1,233, 1,105, 1,023, 770, and 683 cm^−1^; ECD [*λ*_max_ (Δ*ε*)]: 227 (+1,45), 257 (−1.16) nm; (+) HRESIMS: *m/z* 638.2596 [M + NH_4_]^+^, calcd for C_34_H_43_O_11_NH_4_, 638.2601; ^1^H and ^13^C NMR data: see Tables 2, 3.

#### 2.3.5 Xuetonlignan C

White amorphous powder; ${\left[\alpha \right]}_{D}^{24}$ +12.6° (*c* = 0.1, MeOH); UV (MeOH) *λ*_max_ (log *ε*): 219 (1.28) nm; IR *ν*_max_: 3,568, 2,941, 1717, 1,613, 1,463, 1,377, 1,226, 1,138, 1,110, 1,070, 1,038, and 733 cm^−1^; ECD [*λ*_max_ (Δ*ε*)]: 220 (+18.87), 226 (−35.17), 250 (−9.59) nm; (+) HRESIMS: *m/z* 604.2754 [M + NH_4_]^+^, calcd for C_31_H_38_O_11_NH_4_, 604.2758; ^1^H and ^13^C NMR data: see Tables 2, 3.

#### 2.3.6 Xuetonlignan D

White amorphous powder; ${\left[\alpha \right]}_{D}^{24}$ +24.2° (*c* = 0.1, MeOH); UV (MeOH) *λ*_max_ (log *ε*): 204 (4.39), 284 (4.28) nm; IR *ν*_max_: 3,505, 2,882, 1,610, 1,503, 1,431, 1,232, 1,037, 863, and 646 cm^−1^; ECD [*λ*_max_ (Δ*ε*)]: 216 (+16.35), 230 (−7.30), 244 (−6.23) nm; (+) HRESIMS: *m/z* 381.1310 [M + Na]^+^, calcd for C_20_H_22_O_6_Na, 381.1314; ^1^H and ^13^C NMR data: see Tables 2, 3.

#### 2.3.7 Xuetonpene

Yellow oily matter; UV (CH_2_Cl_2_) *λ*_max_ (log ε): 204 (3.34), 287 (2.19) nm; IR *ν*_max_: 3,385, 2,925, 1714, 1,489, 1,443, 1,248, 1,038, 935, and 703 cm^−1^; (+) HRESIMS: *m/z* 213.1276 [M + H]^+^, calcd for C_15_H_17_O, 213.1279; ^1^H and ^13^C NMR data: see Tables 2, 3.

#### 2.3.8 Xuetonin C

${\left[\alpha \right]}_{D}^{24}$ +64.8° (*c* = 0.1, CH_2_Cl_2_); UV (CH_2_Cl_2_) *λ*_max_ (log *ε*): 228 (3.46) nm; IR *ν*_max_: 3,489, 2,923, 2,858, 1709, 1,379, and 1,141 cm^−1^; (+) HRESIMS: *m/z* 477.3335 [M + Na]^+^, calcd for C_30_H_42_O_6_Na, 477.3345; ^1^H and ^13^C NMR data: see Table 1.

### 2.4 X-Ray Crystallographic Analysis

Colorless crystals were obtained from methanol at room temperature by slow evaporation. The X-ray crystallographic data of the compound were obtained using a SuperNova, Dual, Cu at zero, AtlasS2 diffractometer. The structures were determined by direct methods and refined anisotropically with a full-matrix least-squares based on *F* ^*2*^ using the SHELXL-2018 procedure *via* Olex2 software (Zhao et al., 2020). Crystallographic data for **21** have been deposited at the Cambridge Crystallographic Data Center (CCDC: 2102216).

#### 2.4.1 Crystallographic Data of 21

C_32_H_34_O_11_ (*M* = 594.59 g/mol): monoclinic, space group P2_1_ (no. 4), *a* = 9.8206 (2) Å, *b* = 16.2506 (2) Å, *c* = 10.6303 (2) Å, *α* = 90°, *β* = 117.374 (3)°, *γ* = 90°, *V* = 1,506.55 (6) Å^3^, *Z* = 2, *T* = 149.99 (10) K, *μ* (Cu–Kα) = 0.829 mm^−1^, *ρ*_*calc*_ = 1.311 g/cm^3^, 11,564 reflections measured (9.368° ≤ 2Θ ≤ 147.24°), 5,370 unique (*R*_int_ = 0.0190, R_sigma_ = 0.0200), which were used in all calculations. The final *R*_1_ was 0.0294 (I > 2σ(I)) and *wR*_2_ was 0.0767 (all data). The goodness of fit on *F* ^*2*^ was 1.056. Flack parameter: 0.05 (4).

### 2.5 Anti-Rheumatoid Arthritis Fibroblast-Like Synoviocyte Activity Assay

Human HFLS-RA cells were cultured in DME/F-12 with 10% fetal calf serum at 37°C in a constant temperature incubator with 5% CO_2_. The cells were then digested by 0.25% trypsin in 0.02% EDTA. HFLS-RA cells were seeded into each well of 96-well multiplates. After 12 h of incubation at 37°C, the cells were administrated with different doses of compounds (0, 2.5, 5, 7.5, 10, 12.5, 15, and 20 µM) and incubated for another 48 h. The cells were subjected to the MTT assay. Methotrexate was used as the positive control substance (Ding et al., 2019).

### 2.6 Hepatoprotective Activity Assay

Human HepG2 hepatoma cells were cultured in DMEM supplemented with 10% fetal calf serum at 37°C in a humidified atmosphere of 5% CO_2_. HepG2 cells were seeded into 96-well cell culture plates. After overnight incubation, 10 μM test samples and APAP (final concentration of 5 mm) were added into the wells and incubated for another 24 h. The cell viability was determined by the MTT assay. Bicyclol was used as the positive control (Hao et al., 2012).

### 2.7 Molecular Docking Study

The crystal structure of the receptor activator of nuclear factor κ-B ligand (RANKL) (PDB ID: 3urf) was downloaded from the RCSB Protein Data Bank (http://www.rcsb.org/) (Ganesan and Rasool, 2019). The structures of compounds **13** and **15** were drawn by Chemdraw and generated to 3D structures with energy minimization using the MM2 minimize. Docking was performed using Autodock, and structure visualization was performed with Pymol and Discovery Studio software.

## 3 Results and Discussion

**FIGURE 2:**
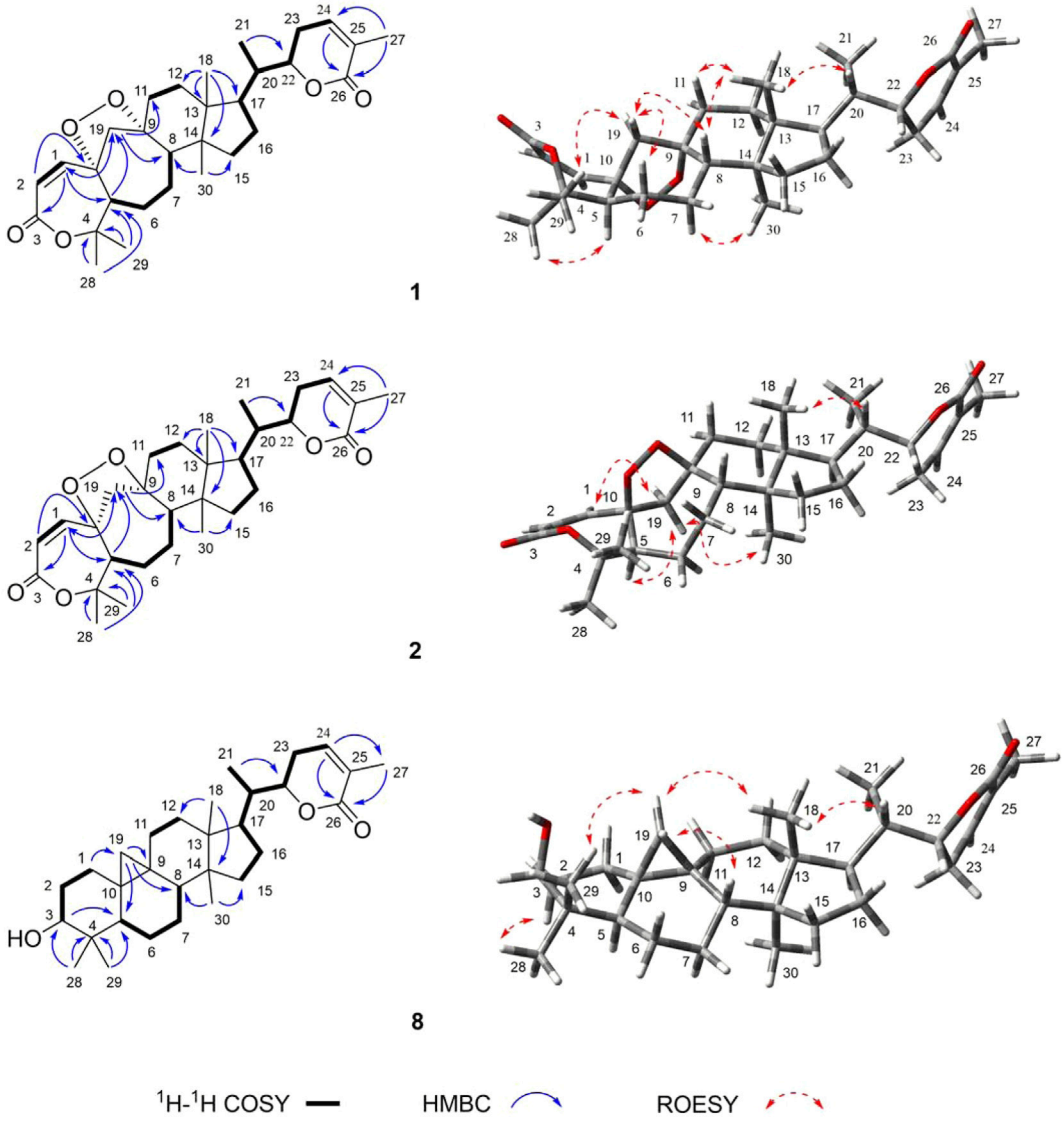
Key ^1^H–^1^H COSY, HMBC, and ROSEY correlations of **1, 2**, and **8**.

**FIGURE 3:**
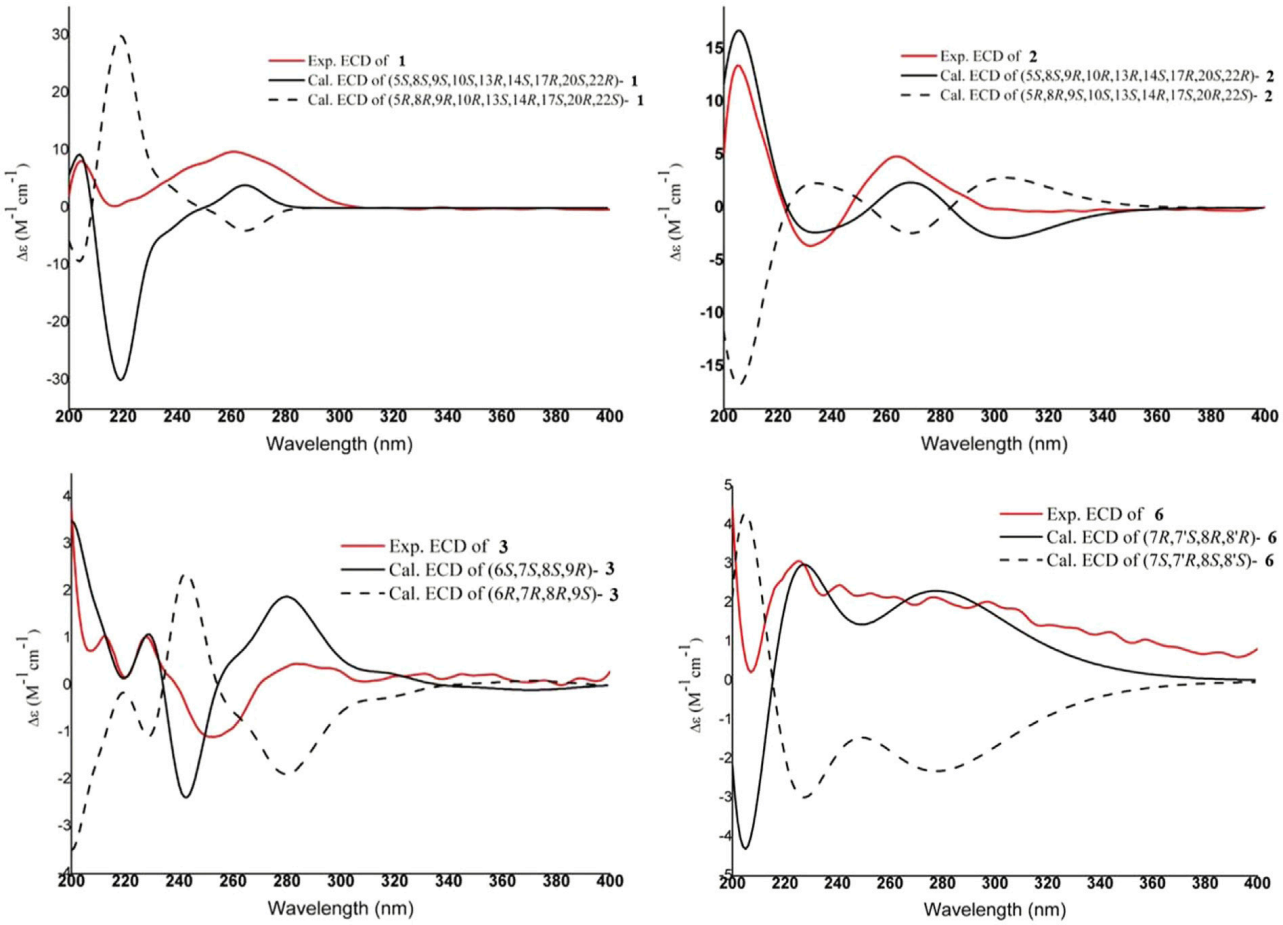
Experimental and calculated ECD spectra of compounds **1–3** and **6**

Compound **1** (xuetonin A) was isolated as a white amorphous powder and was shown to have a molecular formula of C_30_H_42_O_6_ by a positive HRESIMS peak at *m/z* 499.3066 ([M + H]^+^, calcd. 499.3060). The absorption maximum (209 nm) in the UV spectrum was attributed to the α,β-unsaturated ester system. The ^1^H NMR data of **1** showed three olefinic protons at *δ*_H_ 6.61 (1H, d-like, *J* = 6.6 Hz), 6.22 (1H, d, *J* = 12.6 Hz), and 5.94 (1H, d, *J* = 12.6 Hz) that were attributed to two double bonds. An oxygenated methine signal appeared at *δ*_H_ 4.46 (1H, dt, *J* = 13.2, 3.6 Hz) along with six methyl singlets (3H each, *δ*_H_ 1.92, 1.42, 1.40, 0.98, 0.98, and 0.86). The ^13^C NMR and DEPT-135° data displayed 30 carbon signals, including two conjugated carbonyl carbons at *δ*_C_ 166.7 and 165.4, four olefinic carbons at *δ*_C_ 146.9, 139.5, 128.4, and 119.6, three oxygenated quaternary carbons at *δ*_C_ 87.8, 87.0, and 82.9, one oxygenated methenyl carbon at *δ*_C_ 80.5, and six methyl carbons at *δ*_C_ 30.2, 21.6, 17.6, 17.1, 14.5, and 13.7. The NMR data of **1** resembled those of schisanlactone A (Liu et al., 1983a), except for the presence of a peroxy bridge between C-9 and C-10. This was confirmed by the HRESIMS. Moreover, two doublets for the C-19 methylene group resonance signals occurred at *δ*_H_ 2.74 (1H, d, *J* = 12.6 Hz) and 2.26 (1H, d, *J* = 12.6 Hz) due to the effect of the peroxy bridge. This was further evidenced by HMBC correlations of H-2 (*δ*_H_ 5.94)/H-5 (*δ*_H_ 2.42) with C-10 (*δ*_C_ 87.0) and of H-19b (*δ*_H_ 2.18)/H-12b (*δ*_H_ 1.57) with C-9 (*δ*_C_ 87.8) (Figure 2). Thus, the planar structure of **1** was determined as a 3,4-*seco*-cycloartane with a rare peroxy bridge by the 1D-NMR, ^1^H–^1^H COSY, HSQC, and HMBC spectral analyses. The *β*-configuration of H-19 was deduced by the ROESY cross peaks between H-19a (*δ*_H_ 2.74), H-8 (*δ*_H_ 1.63) and CH_3_-29 (*δ*_H_ 1.40). Conversely, the peroxy bridge was deduced to be in the *α*-orientation. Moreover, the absolute configuration of **1** was determined to be 5*S*, 8*S*, 9*S*, 10*S*, 13*R*, 14*S*, 17*R*, 20*S*, and 22*R* by comparing the experimental and calculated ECD spectra (Figure 3). Thus, compound **1** was established and named as xuetonin A.

Compound **2** (xuetonin B) was isolated as a white amorphous powder with the molecular formula C_30_H_42_O_6_, as determined by HRESIMS from the peak at *m/z* 499.3068 ([M + H]^+^, calcd. 499.3060). Comparison of the HRESIMS, UV, 1D-, and 2D-NMR spectra of **2** with those of **1** suggested that they are a pair of 3,4-*seco*-cycloartane isomers with the same planar structure. The differences are the configurations of C-9 and C-10. In compound **2**, the peroxy bridge on C-9 and C-10 was found to be in the *β*-orientation, deduced from ROESY correlations of H-19 (*δ*_H_ 2.76) with H-5*α* (*δ*_H_ 2.45) (Figure 2). Consequently, the absolute configuration of **2** was determined to be 5*S*, 8*S*, 9*R*, 10*R*, 13*R*, 14*S*, 17*R*, 20*S*, and 22*R* based on the comparisons of the experimental ECD curves and calculated ones (Figure 3). Thus, compound **2** was established, and it was named xuetonin B. Compounds **1** and **2** were identified as new 3,4-*seco*-cycloartane triterpenoids with a rare peroxy bridge between C-9 and C-10. To date, only one cycloartane-derived triterpenoid (schinalactone A) containing the peroxy bridge has been found from *Schisandra sphenanthera* (He et al., 2010).

Compound **3** (xuetonlignan A)**,** isolated as white amorphous powders, had the molecular formula C_35_H_38_O_11_ deduced from its HRESIMS analysis (*m/z* 652.2758, [M + NH_4_]^+^, calcd for 652.2758). The UV data of **3** provided a characteristic peak (*λ*_max_ 218) of dibenzocyclooctadiene lignan (Luo et al., 2017). The ^1^H NMR data (Table 1) displayed two aromatic protons for a biphenyl moiety at *δ*_H_ 6.85 and 6.63, two characteristic signals of a methylenedioxy moiety at *δ*_H_ 5.72 and 5.15 ppm, and four singlets for methoxy moiety at *δ*_H_ 3.94, 3.86, 3.66, and 3.58 ppm. A cyclooctadiene ring was deduced. Furthermore, two oxymethine (*δ*_H_ 5.78 and 5.71 ppm), a methine (*δ*_H_ 2.30 ppm), and two methyl signals (*δ*_H_ 1.38 and 1.30 ppm) also appeared in the spectrum. The ^13^C NMR spectrum of **3** showed 35 carbon signals, including 12 aromatic carbons belonging to the biphenyl moiety (*δ*_C_ 153.3, 152.4, 150.1, 142.5, 141.8, 136.9, 134.5, 131.5, 123.4, 121.8, 112.2, and 103.1), a methylenedioxy signal (*δ*_C_ 102.2), three oxymethine carbons (*δ*_C_ 86.2, 84.5, and 75.2), four methoxy groups (*δ*_C_ 61.1, 61.0, 59.4, and 56.6), one methine carbon (*δ*_C_ 44.5), two methyl carbons (*δ*_C_ 29.4 and 17.2) and a *trans*-cinnamoyl group (*δ*_C_ 166.4, 146.0, 135.5, 131.7, 130.0, 130.0, 129.4, 129.4, and 118.2) and a propionyl group (*δ*_C_ 174.2, 27.7, and 8.8) (Dong et al., 2012). The above data indicated that **3** is a C_18_-dibenzocyclooctadiene lignan with a *trans*-cinnamoyl group and a propionyl group. The locations of groups were confirmed by ^1^H–^1^H COSY and HMBC data. The HMBC correlations from H-11 (*δ*_H_ 6.63) to C-12 and C-13 and from the four methoxy protons to C-1, C-2, C-3, and C-14 showed that the methylenedioxy moiety is connected to C-12 and C-13, and the four methoxy moieties are connected to C-1, C-2, C-3, and C-14. The presence of a *trans*-cinnamoyl group at C-6 and a propionyl group at C-9 was deduced by the HMBC correlations from H-6 (*δ*_H_ 5.71) to C-1' (*δ*_C_ 166.4) and C-4 (*δ*_C_ 112.2) and from H-9 (*δ*_H_ 5.78) to C-1'' (*δ*_C_ 174.2) and C-11 (*δ*_C_ 103.1). Furthermore, CH_3_-17 at C-7 and CH_3_-18 at C-8 can together be confirmed by the HMBC correlations between H_3_-17 (*δ*_H_ 1.38, s) and C-6, C-7, C-8, and H_3_-18 (*δ*_H_ 1.30, d) with C-9, C-8, and C-7; and the spin system of H_3_-18/H-8/H-9 in the ^1^H–^1^H COSY.

The absolute configuration of **3** was established with the help of ECD combined with ROESY data. The ECD experiment exhibited a negative cotton effect (CE) around 252 nm and a positive CE at 227 nm, suggesting the *S*-biphenyl configuration of **3** (Luo et al., 2017). The ROESY correlations between H-6/H-4, H-11/H-9/H-8, and H-8/H_3_-17 indicated that H-6 and CH_3_-18 were *α*-oriented, while H-8, CH_3_-17, and H-9 were *β*-oriented. The ROESY and ECD data of **3** were found to be similar to those of **21** (heteroclitalignan D) (Wang et al., 2006b). X-ray crystallographic analysis of **21** eventually established the stereochemistry of **3**, especially at C-6, C-7, C-8, and C-9. Futhermore, based on the comparisons of the experimental and calculated ECD spectra, the absolute configuration of **3** was found to be 6*S*, 7*S*, 8*S*, and 9*R* (Figure 3). Therefore, the structure of **3** was established for xuetonlignan A.

Compound **4** (xuetonlignan B) possesses the molecular formula C_34_H_36_O_11_ through analysis of the HRESIMS (*m/z* 638.2596 [M + NH_4_]^+^). The UV, NMR, and ECD data of **4** indicated the presence of an *S*-biphenyl–configured dibenzocyclooctadiene lignan with almost identical data and the same planar structure to arisanschinin C (Liu et al., 2010). The only difference between them was in the configurations of C-7 and C-8. This can be confirmed by the ROESY correlations of H-4 with H-6, of H-11 with H-9 and H-8, and of H-8 with H-17. This was further confirmed from the similarity between ROESY and ECD spectra of **4** and **3**. Based on the data, the absolute configuration of **4** was shown as 6*S*, 7*S*, 8*S*, and 9*R*. Accordingly, the structure of **4** was established for xuetonlignan B.

Compound **5** (xuetonlignan C) was determined to have the formula C_31_H_38_O_11_ by deducing from its HRESIMS at *m/z* 604.2754 [M + NH_4_]^+^ (calcd for 604.2758). The UV, 1D-NMR, and ECD data showed that **5** is an *S*-biphenyl–configured dibenzocyclooctadiene lignan. Comparison of the spectral data of **9** with kadsuphilol R (Cheng et al., 2011) exhibited the presence of the isobutyryl moiety instead of the angeloyl moiety at C-9 in **5**. The HMBC correlations from H-9 (*δ*_H_ 5.70) to C-1'' (*δ*_C_ 176.8) and from H-2'' (*δ*_H_ 1.93), H-3'' (*δ*_H_ 0.88), and H-4'' (*δ*_H_ 0.87) to C-1'' (*δ*_C_ 176.8) in **5** established the locations of the isobutyryl group at C-9. The ROESY correlations of H-4 with H-6 and 3-OCH_3_, of H-11 with H-9 and H-8, and of H-8 with H_3_-17 indicated that H-6 and CH_3_-18 were *α*-oriented and that H-9, H-8, and CH_3_-17 were *β*-oriented. This was further evidenced from the lack of ROESY correlation between CH_3_-17 and CH_3_-18. Thus, the structure of xuetonlignan C (**5**) was established.

Compound **6** (xuetonlignan D) was obtained as white amorphous powders, having the molecular formula C_20_H_22_O_11_ inferred from its HRESIMS analysis (*m/z* 381.1310, [M + Na]^+^, calcd for 381.1314). The ^1^H NMR spectrum exhibited aromatic protons at *δ*_H_ 6.93 (1H, s), 6.89 (1H, s), 6.81 (1H, d, *J* = 7.8 Hz), and 6.77 (3H, m, overlapped) that were attributed to two 1,3,4-trisubstituted phenyl groups. A methylenedioxy group at 5.93 (2H, s), two oxygenated methenyls at *δ*_H_ 4.52 (1H, d, *J* = 7.8 Hz) and 4.17 (1H, m), an oxygenated methylene at *δ*_H_ 4.19 (1H, m) and 3.98 (1H, t, *J* = 8.4 Hz), a methoxyl at *δ*_H_ 3.87 (3H, s), two methenyls at *δ*_H_ 2.27 (1H, m) and 1.75 (1H, m), and a methyl at 0.62 (3H, d, *J* = 6.6 Hz) signals also appeared in the spectrum. These moieties were also identified based on the ^13^C and DEPT-135° NMR data analysis. Comparison of the 1D-NMR spectral data of **6** with the ones of 3-methoxy-3′,4′-methylenedioxy-7,9′-epoxylignane-4,7′,9-triol, isolated from *Asiasarum heterotropoides*, revealed both compounds to be quite similar structurally, except that **6** lacked a hydroxy group at C-9 (Lee et al., 2013). This was determined by the HMBC correlations from H_3_-9 (*δ*_H_ 0.62) to C-8 (*δ*_C_ 46.0), C-7 (*δ*_C_ 90.6), and C-8' (*δ*_C_ 55.8). The relative stereochemistry was confirmed by ROESY data. ROESY correlations of H-9 with H-7 (*δ*_H_ 4.17) and H-8' (*δ*_H_ 2.27) and of H-8 (*δ*_H_ 1.75) with H-7' (*δ*_H_ 4.52) exhibited that H-9, H-7, and H-8′ were of the same orientation; H-8 and H-7′ were of the same orientation. The absolute configuration of **6** was confirmed by comparing the experimental and calculated ECD spectra (Figure 3). Thus, compound **6** was confirmed to be (7*R*,8*R*,7′*S*,8′*R*)-3-methoxy-3′,4′-methylenedioxy-7,9′-epoxylignane-4,7′-diol and named xuetonlignan D.

Compound **7** (xuetonpene) had the molecular formula C_15_H_16_O on the basis of its HRESIMS data at *m/z* 213.1276 [M + H]^+^ (calcd 213.1279). The ^1^H NMR spectroscopic data showed two singlet signals and two double signals for aromatic protons in two phenyl moieties at *δ*_H_ 7.80, 7.24, 7.17, and 7.03, one pair of proton resonances at *δ*_H_ 5.36 and 5.01, and three methyl groups at *δ*_H_ 2.58, 2.42, and 2.18. The ^13^C NMR, DEPT-135°, and HSQC spectra of **7** showed 15 carbon resonances, including 10 aromatic carbons (*δ*_C_ 152, 140.1, 133.0, 131.4, 128.1, 126.5, 126.0, 125.5, 122.0, and 106.4), two olefinic carbons (*δ*_C_ 145.3 and 115.8), and three methyl carbons (*δ*_C_ 25.6, 19.7, and 16.7). The abovementioned data suggested that **7** was an analog of 7-hydroxycadalene, except for the addition of one terminal double bond at C-11 (Sankaram et al., 1981). This was confirmed by the HMBC correlations from H-12 (*δ*_H_ 5.36 and 5.01) to C-13 (*δ*_C_ 25.6) and C-7 (*δ*_C_ 140.1) (Supplementary Figure S3). Therefore, the structure of xuetonpene (**7**) was defined as shown in Figure 1.

Compound **8** (xuetonin C) was determined to have the molecular formula C_30_H_46_O_3_ from HRESIMS (*m/z*, 477.3335, [M + Na]^+^, calcd 477.3345) analysis. The ^1^H and ^13^C NMR data of **8** were the same as those of 3*β-*hydroxycycloart-24*Z*-ene-22(*S*)→26 lactone, which was an enzymatic hydrolysis compound derived from juncoside I (Greca et al., 1994). The structure of **8** was confirmed by the comprehensive analysis of its 2D NMR data. Thus, **8** has the same structure as 3*β-*hydroxycycloart-24*Z*-ene-22(*S*)→26 lactone and is a new natural product named xuetonin C.

**FIGURE 4:**
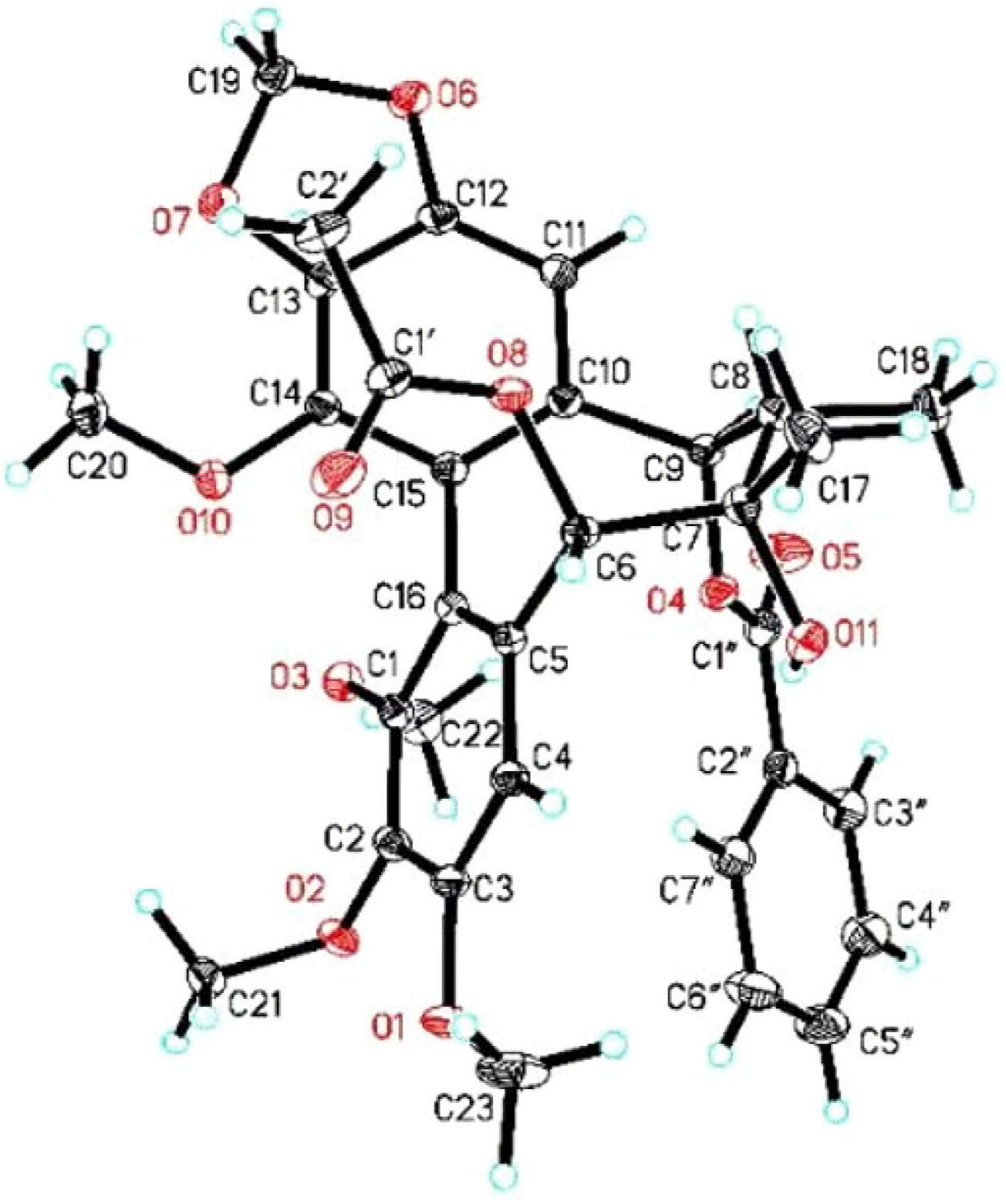
X-ray ORTEP drawing of **21**.

**FIGURE 5:**
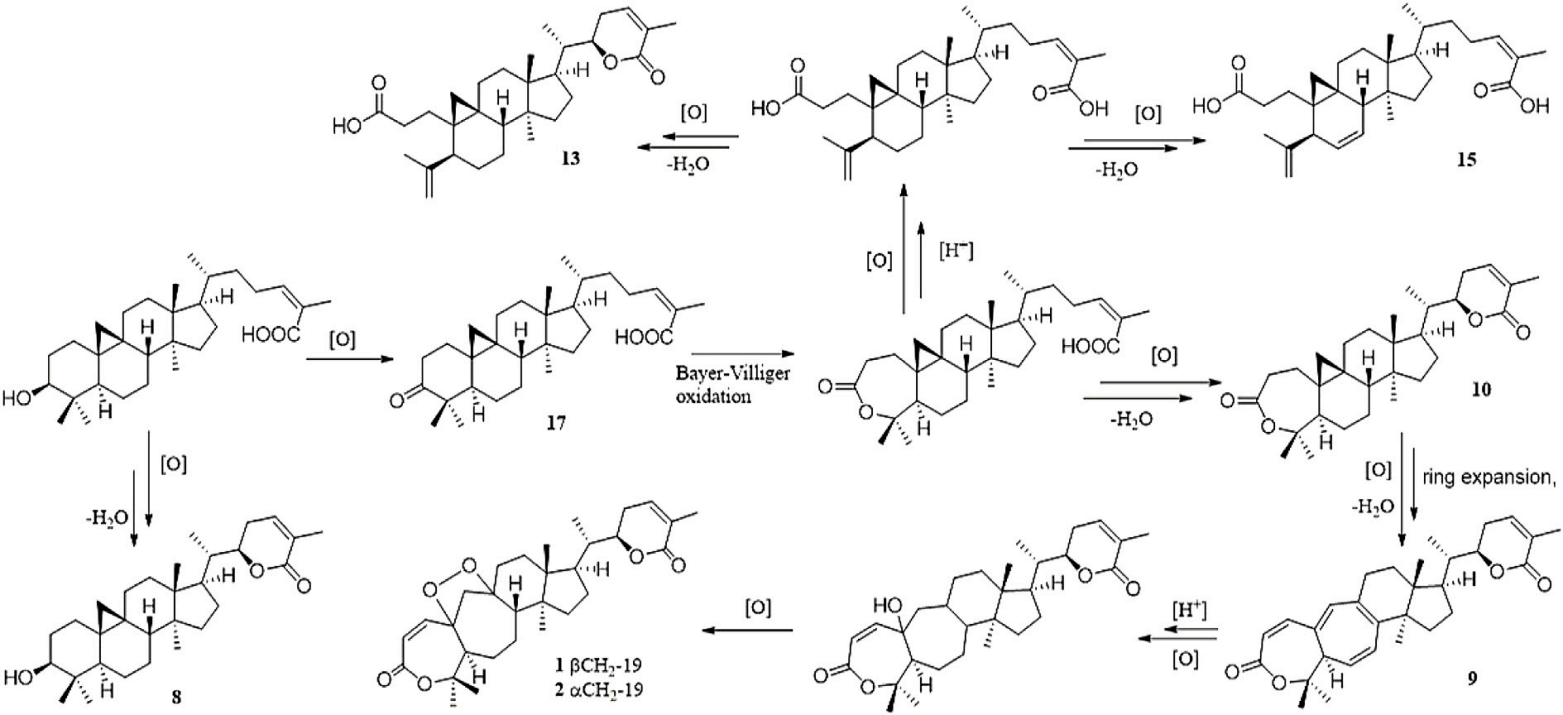
Plausible biosynthetic pathway for **1, 2, 8–10, 13, 15**, and **17**.

Heteroclitalignan D (**21**) was obtained as colorless crystals. The X-ray diffraction data of **21** were reported for the first time in this study (Figure 4). Biosynthetically, mangiferolic acid might be the precursor of compounds **1**, **2**, **8**–**10**, **13**, **15**, and **17** through a series of oxidative cleavage processes *via* esterification, the Baeyer–Villiger oxidation, ring expansion, hydroxylation, cyclization, and epoxidation steps obtained from compounds **1**, **2**, **8**–**10**, **13**, **15**, and **17**, respectively. A plausible biogenetic pathway for **1**, **2**, **8**–**10**, **13**, **15**, and **17** is shown in Figure 5.

Forty-three known compounds isolated during this project were characterized as lancilactone B (**9**) (Chen et al., 1999), kadsudilactone (**10**) (Rui et al., 1991), schisanlactone B (**11**) (Liu et al., 1983b), kadsuphilactone B (**12**) (Shen et al., 2005), xuetongsu (**13**) (Shehla et al., 2020), heteroclitalactone A (**14**) (Wang et al., 2006a), changnanic acid (**15**) (Liu and Huang, 1991), cycloartenone (**16**) (Wang et al., 2006a), schizandronic acid (**17**) (Li et al., 2003), *seco*-coccinic acid F (**18**) (Minh et al., 2014), kadsuracoccinic acid B (**19**) (Li et al., 2008), sorghumol (**20**) (Cambie et al., 1992), heteroclitalignan D (**21**) (Wang et al., 2006b), kadsurarin (**22**) (Chen et al., 1973), kadsuphilol T (**23**) (Cheng et al., 2011), kadsuphilol R (**24**) (Cheng et al., 2011), kadsuphilol C (**25**) (Luo et al., 2017), kadsulignan N (**26**) (Gao et al., 1998), enshizhisu (**27**) (Huang et al., 1982), machilolin A (**28**) (Chen et al., 2009), (+)-pinoresinol (**29**) (Fan et al., 2020), (+)-2-(3,4-dimethoxyphenyl)-6-(3,4-dimethoxyphenyl)-3,7-dioxabicyclo [3,3,0] octane (**30**) (Latip et al., 1999), *meso*-dihydroguaiaretic acid (**31**) (Lu and Chen, 2008), 6α,​9α-​dihydroxycadinan-​4-​en-​3-​one (**32**) (Cao et al., 2019c), (4*R*)-4-hydroxy-1,10-*seco*-muurol-5-ene-1,10-dione (**33**) (Kiem et al., 2014), litseachromolaevane A (**34**) (Zhang et al., 2003), cryptomeridiol (**35**) (Ragasa et al., 2005), (-)-5*β*,11-dihydroxyiphionan-4-one (**36**) (Lin et al., 2019), aromadendrane-4*β*,10*α*-diol (**37**) (Goldsby and Burke, 1987), lochmolin F (**38**) (Tseng et al., 2012), loliolide (**39**) (Kim et al., 2004), *β-*sitosterol (**40**) (Luo et al., 2009), daucosterol (**41**) (Tezuka et al., 1998), stigmasterol (**42**) (Luo et al., 2009), schleicheol 2 (**43**) (Pettit et al., 2000), 7-hydroxy-*β-*sitosterol (**44**) (Chaurasia and Wichtl, 1987), stigmastan-3-one (**45**) (Brasil et al., 2010), mexoticin (**46**) (Chakraborty et al., 1967), pterosonin E (**47**) (Liu R. H et al., 2018), physcion (**48**) (Pang et al., 2016), 5-*O*-methylvisanninol (**49**) (Baba et al., 1981), shikimic acid (**50**) (Talapatra et al., 1989), and protocatechuic acid (**51**) (Guan et al., 2009) by comparing their NMR spectrum with the reported literature.

The anti-RAFLS activities of the isolated terpenoids (**1**–**2**, **7**–**20**, and **32**–**39**) were assessed on the RA fibroblast-like synoviocytes. Compounds **2**, **10**, **13**–**15**, and **17**–**19** displayed evident inhibitory activities on the RA fibroblast-like synoviocytes with IC_50_ values of 19.81 ± 0.26, 12.73 ± 0.29, 5.70 ± 0.24, 9.25 ± 0.79, 5.66 ± 0.52, 11.91 ± 0.44, 13.22 ± 0.27, and 15.94 ± 0.36 μM, respectively, as shown in Table 4. The structure–activity relationship (SAR) study showed that the introduction of the carboxyl moiety enhances the activity. Furthermore, the results also showed that the orientation of C-19 affected the anti-RAFLS effects, as is evident from the data obtained for compounds **1** and **2**. According to the abovementioned bioactivity results, it could be preliminarily deduced that triterpenoids may be the principal chemical constituents responsible for the anti-RAFLS effect of the leaves of *K. heteroclita*.

**TABLE 4:** ｜Effects of compounds **2**, **10**, **13**–**15**, and **17**–**19** on rheumatoid arthritis fibroblast-like synoviocytes.

Compounds	IC_50_ (μM)
**2**	19.81 ± 0.26
**10**	12.73 ± 0.29
**13**	5.70 ± 0.24
**14**	9.25 ± 0.79
**15**	5.66 ± 0.52
**17**	11.91 ± 0.44
**18**	13.22 ± 0.27
**19**	15.94 ± 0.36
Methotrexate^a^	3.10 ± 0.68

a Positive control.

**FIGURE 6:**
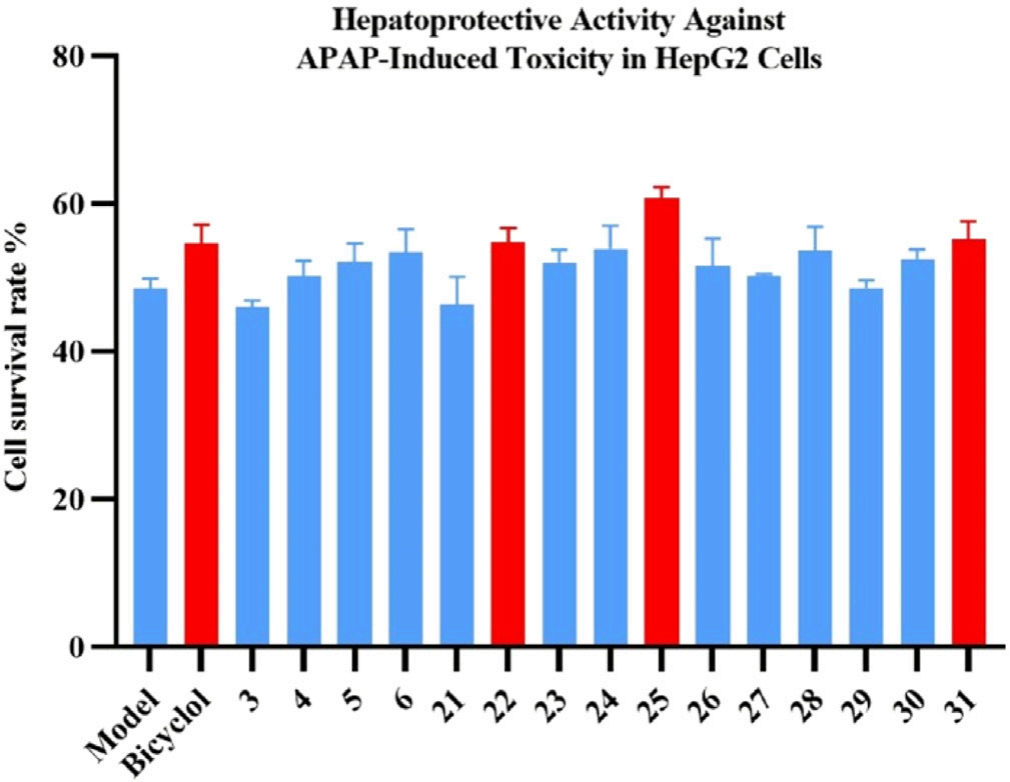
Effects of compounds **3–6** and **21–31** on *N*-acteyl-*p*-aminophenol (APAP)–induced toxicity in HepG2 cells. Data are presented as the mean ± SD (*n* = 3). Bicyclol was used as the positive control.

The hepatoprotective activities of the isolated lignans (**3**–**6**, **21**–**31**) were evaluated in APAP-induced toxicity in HepG2 cells at 10 μM. Compounds **22**, **25**, and **31** showed significant hepatoprotective activity with increasing cell viability by 12.93%, 25.23%, and 13.91% compared with the model group (cf. bicyclol, 12.60%) at 10 μM, respectively, as shown in Figure 6. According to the abovementioned bioactivity results, it could be preliminarily deduced that lignans may be the principal components for the hepatoprotective effect of the leaves of *K. heteroclita*.

### 3.1 Molecular Docking

**FIGURE 7:**
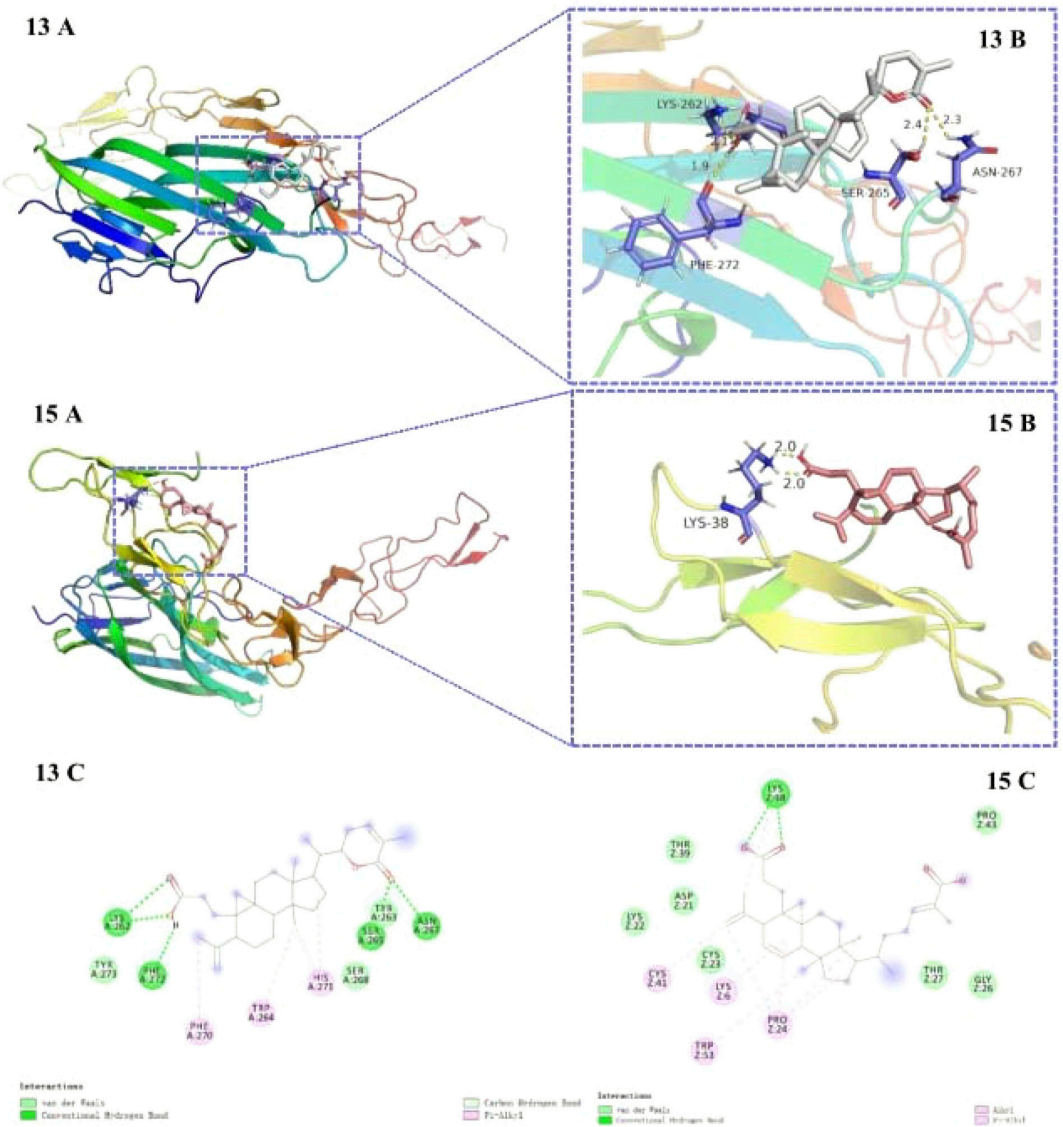
Docking poses **(A)** and interactions **(B, C)** of compounds **13** and **15** at the binding site of RANKL (receptor activator of nuclear factor k-B ligand). Hydrogen bonds and hydrophobic interactions are represented by the green and pink lines, respectively **(c)**.

Compounds **13** and **15** exhibited lesser docking parameters (binding energy: −5.38 and −4.20 kcal/mol, respectively). As shown in Figure 6, compound **13** formed hydrogen bonds with LYS-267, PHE-272, SER-265, and ASN-267 residues and hydrophobic interactions with PHE-270, TRP-264, and HIS-271 residues. Similarly, compound **15** mainly interacted with LYS-38 by hydrogen bonds and with CYS-41, LYS-6, TPR-53, and PRO-24 by hydrophobic interactions. This docking simulation revealed the important role of the carboxyl moiety at C-3 in the structures of compounds **13** and **15** (Figure 7).

## 4 Conclusion

In summary, a total of 51 compounds, including two new highly oxidized cycloartane-type triterpenoids, four new lignans, one new sesquiterpene, and a new natural product, were obtained from the leaves of *K. heteroclita*. Among them, compounds **13**–**15** displayed potent anti-RAFLS activity with IC_50_ values of 5.70 ± 0.24, 9.25 ± 0.79, and 5.66 ± 0.52 μM, respectively, using methotrexate (IC_50_ = 3.10 ± 0.68 μM) as the positive control by the MTT method. In addition, the orientation of CH_3_-17 in dibenzocyclooctadiene lignans was determined by the direct ROE correlation of H-4 but not by the ROE correlation of H-6, even if they had ROE correlations, which were determined by X-ray diffraction of compound **21**. This is the first phytochemical report of the leaves of *K. heteroclita*. It was observed that its main compound types are similar with those of the stem of *K. heteroclita*. It can, thus, be inferred that the leaves may also be used to treat relevant diseases. This study provides a bridge between traditional uses and modern biological studies and offers the experimental basis for the full development of *K. heteroclita*, which is of great significance in terms of scientific value.

## Data Availability

The data sets presented in this study can be found in online repositories. The names of the repository/repositories and accession number(s) can be found in the article/Supplementary Material.
