# Supplementary material for: Anti-RAFLS Triterpenoids and Hepatoprotective Lignans From the Leaves of Tujia Ethnomedicine Kadsura heteroclita (Xuetong)
Source: Front Chem. 2022 May 10;10:878811. doi: 10.3389/fchem.2022.878811 (PMC9127087; doi:10.3389/fchem.2022.878811)
Supplement: Supplementary file 1 [file DataSheet1.PDF]

Anti-RAFLS triterpenoids and hepatoprotective lignans from the leaves of Tujia ethnomedicine *Kadsura heteroclita* (Xuetong)

Mengyun Wang[1], Sai Jiang[1], Nusrat Hussain[2], Salman Zafar[3], Qingling Xie[1], Feibing Huang[1], Linxi Mao[1], Bin Li[1], Yuqing Jian[1,] and Wei Wang[1,]**

[1]*TCM and Ethnomedicine Innovation & Development International Laboratory, Innovative Material Medical Research Institute, School of Pharmacy, Hunan University of Chinese Medicine, Changsha, China,* [2]*Department of Chemistry, University of Baltistan Skardu, Skardu, Pakistan,* [3]*Institute of Chemical Sciences, University of Peshawar, Peshawar, Pakistan*

*Correspondence:

Yuqing Jian

cpujjyq2010@163.com

Wei Wang

wangwei402@hotmail.com

Contents

1. Chemical structures of compounds 1-51 9

Figure S1. Chemical structures of compounds 1-51. 10

2. 2D NMR correlations of compounds 1-8. 11

Figure S2. The key ^{1}H–^{1}H COSY, HMBC, and ROESY correlations of 1, 2 and 8. 11

Figure S3. The key ^{1}H–^{1}H COSY, HMBC, and ROESY correlations of 3-7. 12

3. 1D and 2D NMR spectra of compounds 1-8. 13

Figure S4. The ^{1}H NMR spectrum of 1 in $CDCl_3$ 13

Figure S5. The ^{13}C NMR spectrum of 1 in $CDCl_3$ 13

Figure S6. The DEPT spectrum of 1 in $CDCl_3$ 14

Figure S7. The ^{1}H-^{1}H COSY spectrum of 1 in $CDCl_3$ 14

Figure S8. The HSQC spectrum of 1 in $CDCl_3$ 15

Figure S9. The HMBC spectrum of 1 in $CDCl_3$ 15

Figure S10. The ROESY spectrum of 1 in $CDCl_3$ 16

Figure S11. The HRESIMS spectrum of 1 16

Figure S12. The UV spectrum of 1 in CH_2Cl_2 16

Figure S13. The IR spectrum of 1 17

Figure S14. The ^{1}H NMR spectrum of 2 in $CDCl_3$ 17

Figure S15. The ^{13}C NMR spectrum of 2 in $CDCl_3$ 18

Figure S16. The DEPT spectrum of 2 in $CDCl_3$ 18

Figure S17. The ^{1}H-^{1}H COSY spectrum of 2 in $CDCl_3$ 19

Figure S18. The HSQC spectrum of 2 in $CDCl_3$ 19

Figure S19. The HMBC spectrum of 2 in $CDCl_3$ 20

Figure S20. The ROESY spectrum of 2 in $CDCl_3$ 20

Figure S21. The HRESIMS spectrum of 2 21

Figure S22. The UV spectrum of 2 in CH_2Cl_2 21

Figure S23. The IR spectrum of 2 21

Figure S24. The ^{1}H NMR spectrum of 3 in CD_3OD 22

Figure S25. The ^{13}C NMR spectrum of 3 in CD_3OD 22
Figure S26. The DEPT spectrum of 3 in CD_3OD 23
Figure S27. The ^1H-^1H-COSY spectrum of 3 in CD_3OD 23
Figure S28. The HSQC spectrum of 3 in CD_3OD 24
Figure S29. The HMBC spectrum of 3 in CD_3OD 24
Figure S30. The ROESY spectrum of 3 in CD_3OD 25
Figure S31. The HRESIMS spectrum of 3 in CD_3OD 25
Figure S32. The ECD spectrum of 3 in MeOH 26
Figure S33. The UV spectrum of 3 in MeOH 26
Figure S34. The IR spectrum of 3 26
Figure S35. The ^1H NMR spectrum of 4 in CD_3OD 27
Figure S36. The ^{13}C NMR spectrum of 4 in CD_3OD 27
Figure S37. The DEPT spectrum of 4 in CD_3OD 28
Figure S38. The ^1H-^1H COSY spectrum of 4 in CD_3OD 28
Figure S39. The HSQC spectrum of 4 in CD_3OD 29
Figure S40. The HMBC spectrum of 4 in CD_3OD 29
Figure S41. The ROSEY spectrum of 4 in CD_3OD 30
Figure S42. The HRESIMS spectrum of 4 30
Figure S43. The ECD spectrum of 4 in MeOH 31
Figure S44. The UV spectrum of 4 in MeOH 31
Figure S45. The IR spectrum of 4 31
Figure S46. The ^1H NMR spectrum of 5 in CD_3OD 32
Figure S47. The ^{13}C NMR spectrum of 5 in CD_3OD 32
Figure S48. The DEPT spectrum of 5 in CD_3OD 33
Figure S49. The ^1H-^1H COSY spectrum of 5 in CD_3OD 33
Figure S50. The HSQC spectrum of 5 in CD_3OD 34
Figure S51. The HMBC spectrum of 5 in CD_3OD 34
Figure S52. The ROESY spectrum of 5 in CD_3OD 35
Figure S53. The HRESIMS spectrum of 5 35

Figure S54. The ECD spectrum of 5 in MeOH 36
Figure S55. The UV spectrum of 5 in MeOH...36
Figure S56. The IR spectrum of 5...36
Figure S57. The ^{1}H NMR spectrum of 6 in CD_3OD...37
Figure S58. The ^{13}C NMR spectrum of 6 in CD_3OD 37
Figure S59. The DEPT spectrum of 6 in CD_3OD 38
Figure S60. The ^{1}H-^{1}H COSY spectrum of 6 in CD_3OD....................................38
Figure S61. The HSQC spectrum of 6 in CD_3OD 39
Figure S62. The HMBC spectrum of 6 in CD_3OD 39
Figure S63. The HMBC spectrum of 6 in CD_3OD 40
Figure S64. The HRESIMS spectrum of 6 40
Figure S65. The ECD spectrum of 6 in MeOH 41
Figure S66. The UV spectrum of 6 in MeOH...41
Figure S67. The IR spectrum of 6...41
Figure S68. The ^{1}H NMR spectrum of 7 in $CDCl_3$...42
Figure S69. The ^{13}C NMR spectrum of 7 in $CDCl_3$ 42
Figure S70. The DEPT spectrum of 7 in $CDCl_3$...43
Figure S71. The ^{1}H-^{1}H COSY spectrum of 7 in $CDCl_3$......................................43
Figure S72. The HSQC spectrum of 7 in $CDCl_3$ 44
Figure S73. The HMBC spectrum of 7 in $CDCl_3$ 44
Figure S74. The HRESIMS spectrum of 7 45
Figure S75. The UV spectrum of 7 in CH_2Cl_2..45
Figure S76. The IR spectrum of 7...45
Figure S77. The ^{1}H NMR spectrum of 8 in $CDCl_3$...46
Figure S78. The ^{13}C NMR spectrum of 8 in $CDCl_3$ 46
Figure S79. The DEPT spectrum of 8 in $CDCl_3$...47
Figure S80. The ^{1}H-^{1}H COSY spectrum of 8 in $CDCl_3$......................................47
Figure S81. The HSQC spectrum of 8 in $CDCl_3$ 48
Figure S82. The HMBC spectrum of 8 in $CDCl_3$ 48

Figure S83. The ROESY spectrum of 8 in $CDCl_3$ 49

Figure S84. The HRESIMS spectrum of 8 49

Figure S85. The UV spectrum of 8 in CH_2Cl_2 50

Figure S86. The IR spectrum of 8 50

4. 1D NMR spectra of known compounds 9-51. 51

Figure S87. The ^{1}H NMR spectrum of 9 in Acetone-d_6 51

Figure S88. The ^{13}C NMR spectrum of 9 in Acetone-d_6 51

Figure S89. The ^{1}H NMR spectrum of 10 in Acetone-d_6 52

Figure S90. The ^{13}C NMR spectrum of 10 in Acetone-d_6 52

Figure S91. The ^{1}H NMR spectrum of 11 in Acetone-d_6 53

Figure S92. The ^{13}C NMR spectrum of 11 in Acetone-d_6 53

Figure S93. The ^{1}H NMR spectrum of 12 in CD_3OD 54

Figure S94. The ^{13}C NMR spectrum of 12 in CD_3OD 54

Figure S95. The ^{1}H NMR spectrum of 13 in $CDCl_3$ 55

Figure S96. The ^{13}C NMR spectrum of 13 in $CDCl_3$ 55

Figure S97. The ^{1}H NMR spectrum of 14 in CD_3OD 56

Figure S98. The ^{13}C NMR spectrum of 14 in CD_3OD 56

Figure S99. The ^{1}H NMR spectrum of 15 in CD_3OD 57

Figure S100. The ^{13}C NMR spectrum of 15 in CD_3OD 57

Figure S101. The ^{1}H NMR spectrum of 16 in $CDCl_3$ 58

Figure S102. The ^{13}C NMR spectrum of 16 in $CDCl_3$ 58

Figure S103. The ^{1}H NMR spectrum of 17 in $CDCl_3$ 59

Figure S104. The ^{13}C NMR spectrum of 17 in $CDCl_3$ 59

Figure S105. The ^{1}H NMR spectrum of 18 in $CDCl_3$ 60

Figure S106. The ^{13}C NMR spectrum of 18 in $CDCl_3$ 60

Figure S107. The ^{1}H NMR spectrum of 19 in $CDCl_3$ 61

Figure S108. The ^{13}C NMR spectrum of 19 in $CDCl_3$ 61

Figure S109. The ^{1}H NMR spectrum of 20 in $CDCl_3$ 62

Figure S110. The ^{13}C NMR spectrum of 20 in $CDCl_3$ 62

Figure S111. The ^{1}H NMR spectrum of 21 in CD_3OD 63
Figure S112. The ^{13}C NMR spectrum of 21 in CD_3OD 63
Figure S113. The DEPT spectrum of 21 in CD_3OD 64
Figure S114. The ^{1}H-^{1}H COSY spectrum of 21 in CD_3OD 64
Figure S115. The HSQC spectrum of 21 in CD_3OD 65
Figure S116. The HMBC spectrum of 21 in CD_3OD 65
Figure S117. The ROESY spectrum of 21 in CD_3OD 66
Figure S118. The HRESIMS spectrum of 21 in CD_3OD 66
Figure S119. The ECD spectrum of 21 in MeOH 67
Figure S120. The UV spectrum of 21 in MeOH 67
Figure S121. The ^{1}H NMR spectrum of 22 in CD_3OD 67
Figure S122. The ^{13}C NMR spectrum of 22 in CD_3OD 68
Figure S123. The ^{1}H NMR spectrum of 23 in CD_3OD 68
Figure S124. The ^{13}C NMR spectrum of 23 in CD_3OD 69
Figure S125. The ^{1}H NMR spectrum of 24 in CD_3OD 69
Figure S126. The ^{13}C NMR spectrum of 24 in CD_3OD 70
Figure S127. The ^{1}H NMR spectrum of 25 in CD_3OD 70
Figure S128. The ^{13}C NMR spectrum of 25 in CD_3OD 71
Figure S129. The ^{1}H NMR spectrum of 26 in CD_3OD 71
Figure S130. The ^{13}C NMR spectrum of 26 in CD_3OD 72
Figure S131. The ^{1}H NMR spectrum of 27 in CD_3OD 72
Figure S132. The ^{13}C NMR spectrum of 27 in CD_3OD 73
Figure S133. The ^{1}H NMR spectrum of 28 in $CDCl_3$ 73
Figure S134. The ^{13}C NMR spectrum of 28 in $CDCl_3$ 74
Figure S135. The ^{1}H NMR spectrum of 29 in $CDCl_3$ 74
Figure S136. The ^{13}C NMR spectrum of 29 in $CDCl_3$ 75
Figure S137. The ^{1}H NMR spectrum of 30 in CD_3OD 75
Figure S138. The ^{13}C NMR spectrum of 30 in CD_3OD 76
Figure S139. The ^{1}H NMR spectrum of 31 in CD_3OD 76

Figure S140. The ^{13}C NMR spectrum of 31 in CD_3OD 77
Figure S141. The ^{1}H NMR spectrum of 32 in CD_3OD.. 77
Figure S142. The ^{13}C NMR spectrum of 32 in CD_3OD 78
Figure S143. The ^{1}H NMR spectrum of 33 in CD_3OD.. 78
Figure S144. The ^{13}C NMR spectrum of 33 in CD_3OD 79
Figure S145. The ^{1}H NMR spectrum of 34 in CD_3OD.. 79
Figure S146. The ^{13}C NMR spectrum of 34 in CD_3OD 80
Figure S147. The ^{1}H NMR spectrum of 35 in CD_3OD.. 80
Figure S148. The ^{13}C NMR spectrum of 35 in CD_3OD 81
Figure S149. The ^{1}H NMR spectrum of 36 in CD_3OD.. 81
Figure S150. The ^{13}C NMR spectrum of 36 in CD_3OD 82
Figure S151. The ^{1}H NMR spectrum of 37 in CD_3OD.. 82
Figure S152. The ^{13}C NMR spectrum of 37 in CD_3OD 83
Figure S153. The ^{1}H NMR spectrum of 38 in CD_3OD.. 83
Figure S154. The ^{13}C NMR spectrum of 38 in CD_3OD 84
Figure S155. The ^{1}H NMR spectrum of 39 in CD_3OD.. 84
Figure S156. The ^{13}C NMR spectrum of 39 in CD_3OD 85
Figure S157. The ^{1}H NMR spectrum of 41 in DMSO-d_6.. 85
Figure S158. The ^{13}C NMR spectrum of 41 in DMSO-d_6.. 86
Figure S159. The ^{1}H NMR spectrum of 43 in $CDCl_3$.. 86
Figure S160. The ^{13}C NMR spectrum of 43 in $CDCl_3$ 87
Figure S161. The ^{1}H NMR spectrum of 44 in CD_3OD.. 87
Figure S162. The ^{13}C NMR spectrum of 44 in CD_3OD 88
Figure S163. The ^{1}H NMR spectrum of 45 in CD_3OD.. 88
Figure S164. The ^{13}C NMR spectrum of 45 in CD_3OD 89
Figure S165. The ^{1}H NMR spectrum of 46 in CD_3OD.. 89
Figure S166. The ^{13}C NMR spectrum of 46 in CD_3OD 90
Figure S167. The ^{1}H NMR spectrum of 47 in CD_3OD.. 90
Figure S168. The ^{13}C NMR spectrum of 47 in CD_3OD 91

Figure S169. The ^{1}H NMR spectrum of 48 in $CDCl_3$..91
Figure S170. The ^{13}C NMR spectrum of 48 in $CDCl_3$ 92
Figure S171. The ^{1}H NMR spectrum of 49 in CD_3OD..92
Figure S172. The ^{13}C NMR spectrum of 49 in CD_3OD 93
Figure S173. The ^{1}H NMR spectrum of 50 in DMSO-d_6..93
Figure S174. The ^{13}C NMR spectrum of 50 in DMSO-d_6..94
Figure S175. The ^{1}H NMR spectrum of 51 in CD_3OD..94
Figure S176. The ^{13}C NMR spectrum of 51 in CD_3OD 95

1. Chemical structures of compounds 1-51.

Compounds **1-7** are new compounds, and compound **8** is a new natural product.

Compounds **9**, **19**, **26-28**, **30**, **31**, **33**, **34**, **36-39**, **42-49**, and **51** were isolated from *K. heteroclita* for the first time.

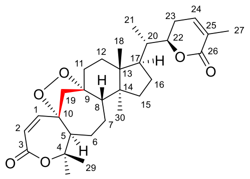

1

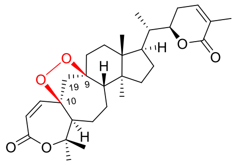

2

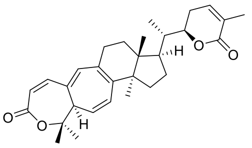

9

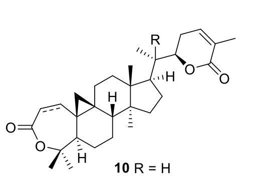

10 R = H
11 R = H, $\Delta^{1(2)}$
12 R = OH, $\Delta^{1(2)}$

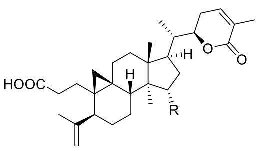

13 R = H
14 R = OAc

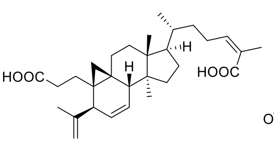

15

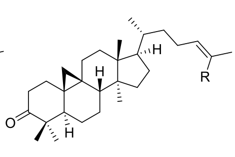

16 R = CH_3
17 R = COOH

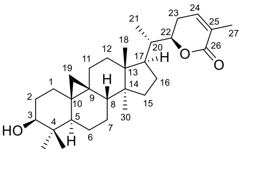

8

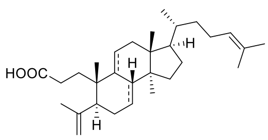

18 $\Delta^{7(8)}$
19 $\Delta^{9(11)}$

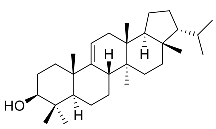

20

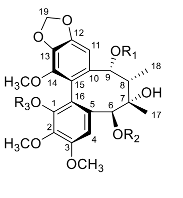

3 R_1 = Prop, R_2 = *t*-Cin, R_3 = CH_3
4 R_1 = *t*-Cin, R_2 = Ac, R_3 = CH_3
5 R_1 = Isobut, R_2 = Ang, R_3 = H
21 R_1 = Bz, R_2 = Ac, R_3 = CH_3
22 R_1 = Ac, R_2 = Ang, R_3 = CH_3
23 R_1 = Bz, R_2 = Ang, R_3 = H
24 R_1 = Ang, R_2 = Ang, R_3= H

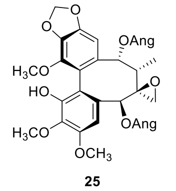

25

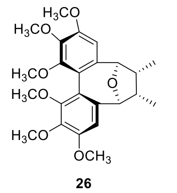

26

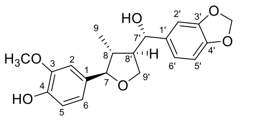

6

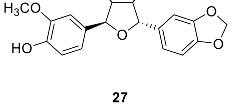

27

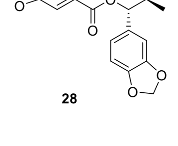

28

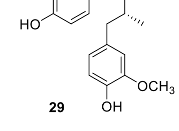

29

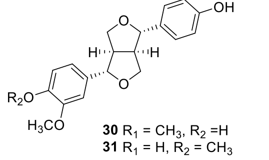

30 R_1 = CH_3, R_2 =H
31 R_1 = H, R_2 = CH_3

trans-Cin = 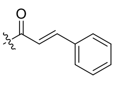

Bz = 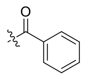

Ang = 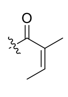

Isobut = 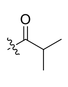

Prop = 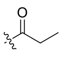

Ac = 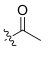

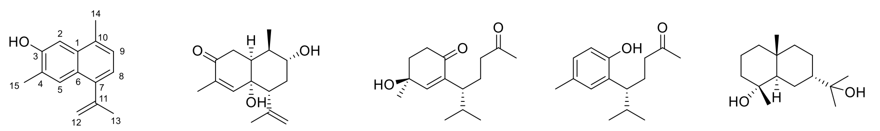

7 32 33 34 35

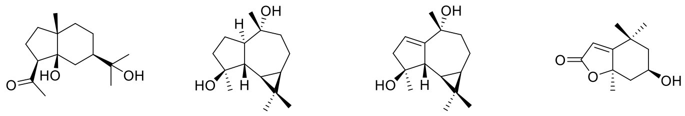

36 37 38 39

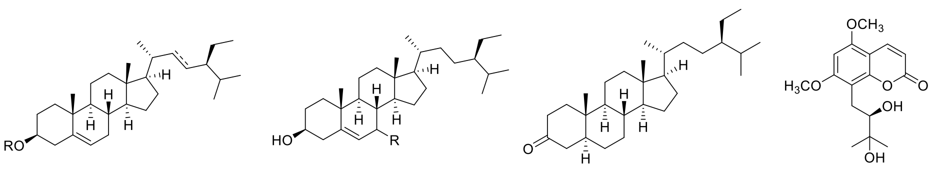

40 R = H
41 R = Glc
42 R = H, $\Delta^{22(23)}$

43 R = αOCH_3
44 R = βOH

45

46

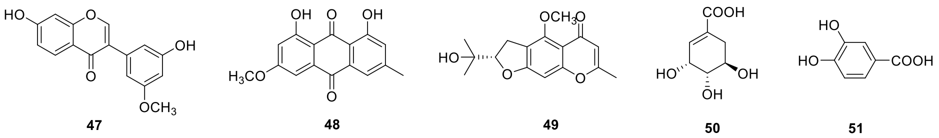

47 48 49 50 51

Figure S1. Chemical structures of compounds 1-51.

Compounds **40** and **42** were identified by TLC methods which compared with standard sample.

2. 2D NMR correlations of compounds 1-8.

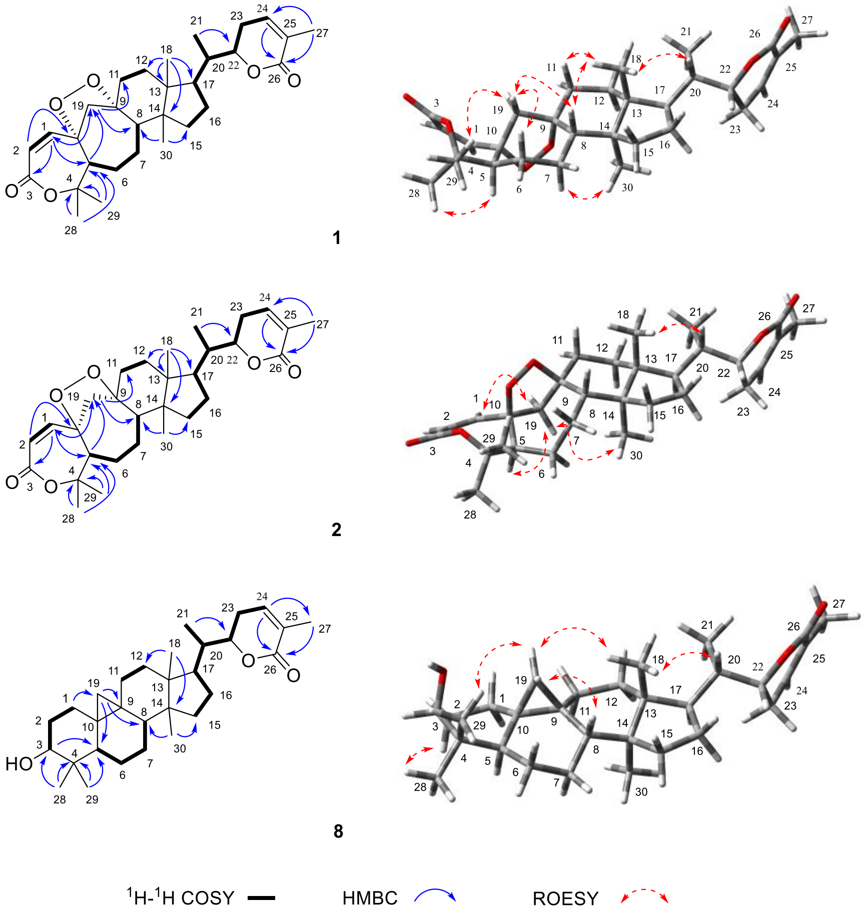

Figure S2. The key ^{1}H–^{1}H COSY, HMBC, and ROESY correlations of 1, 2 and 8.

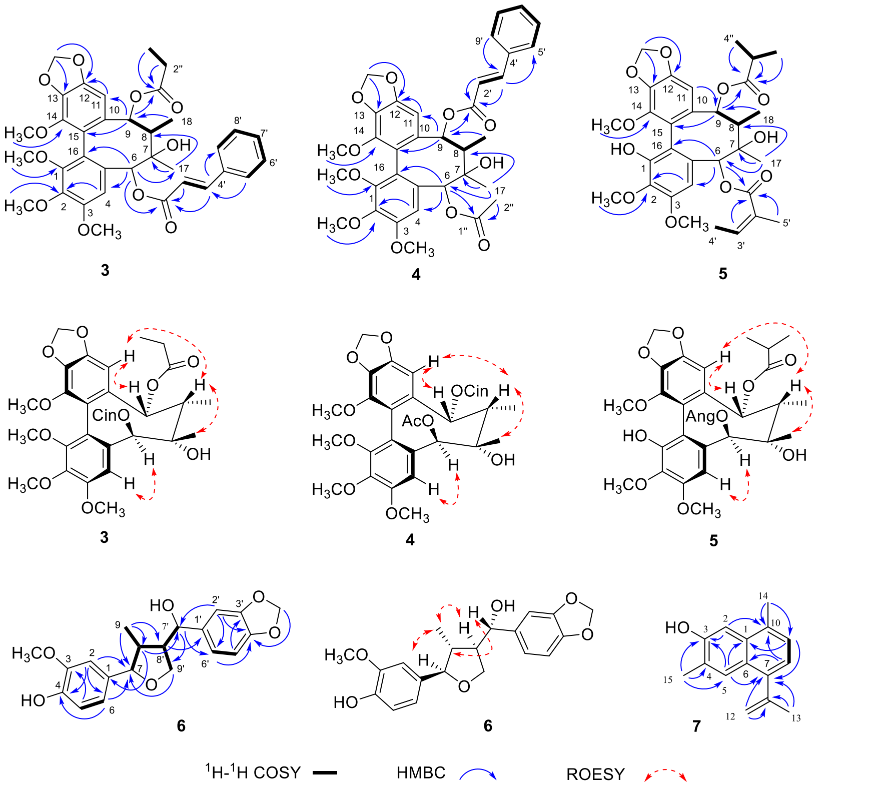

Figure S3. The key $^{1}H-^{1}H$ COSY, HMBC, and ROESY correlations of 3-7.

3. 1D and 2D NMR spectra of compounds 1-8.

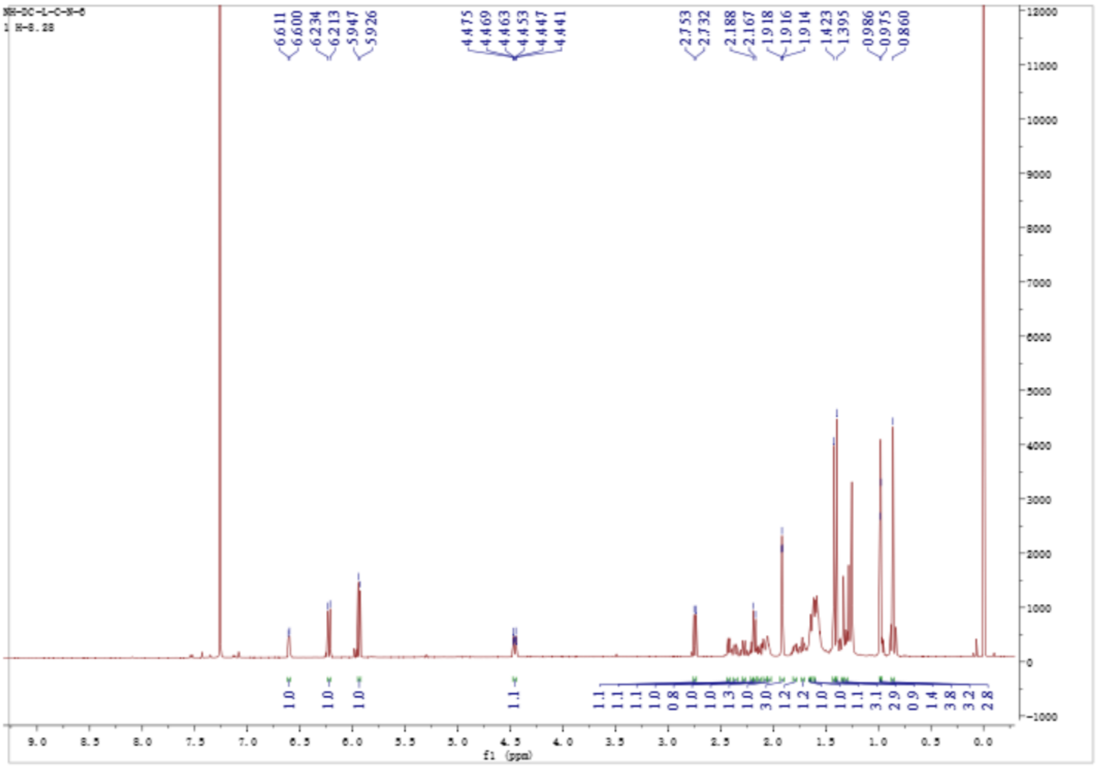

Figure S4. The ^{1}H NMR spectrum of 1 in $CDCl_3$

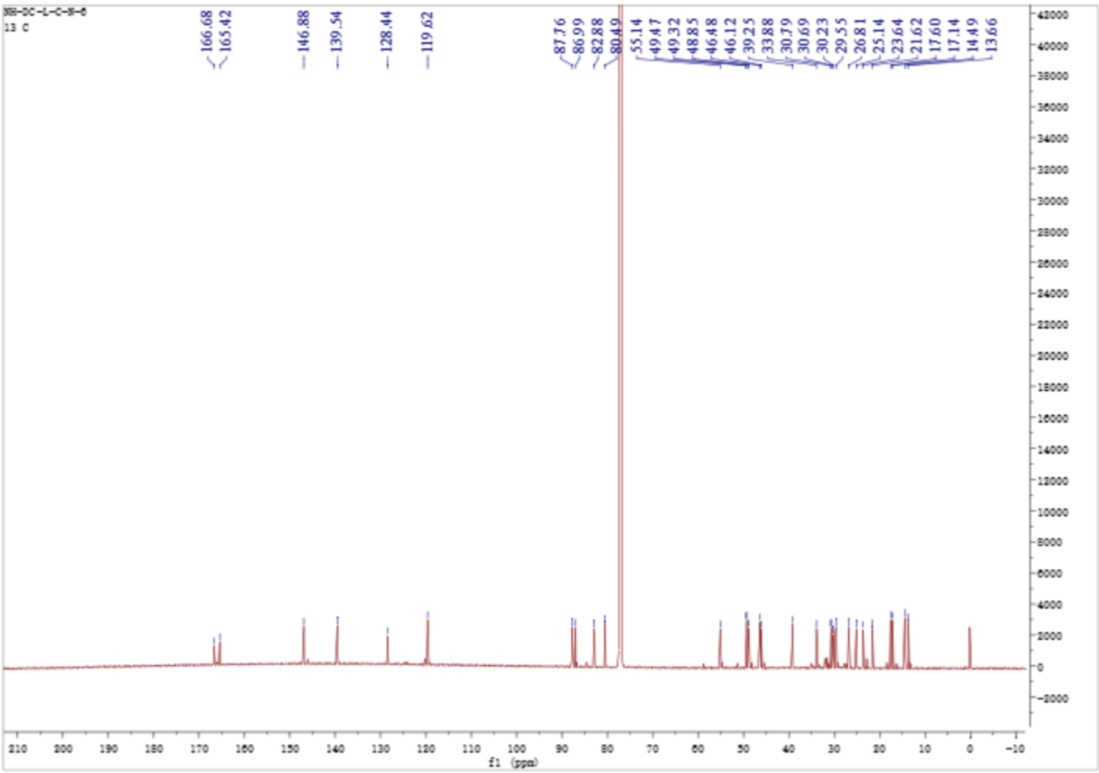

Figure S5. The ^{13}C NMR spectrum of 1 in $CDCl_3$

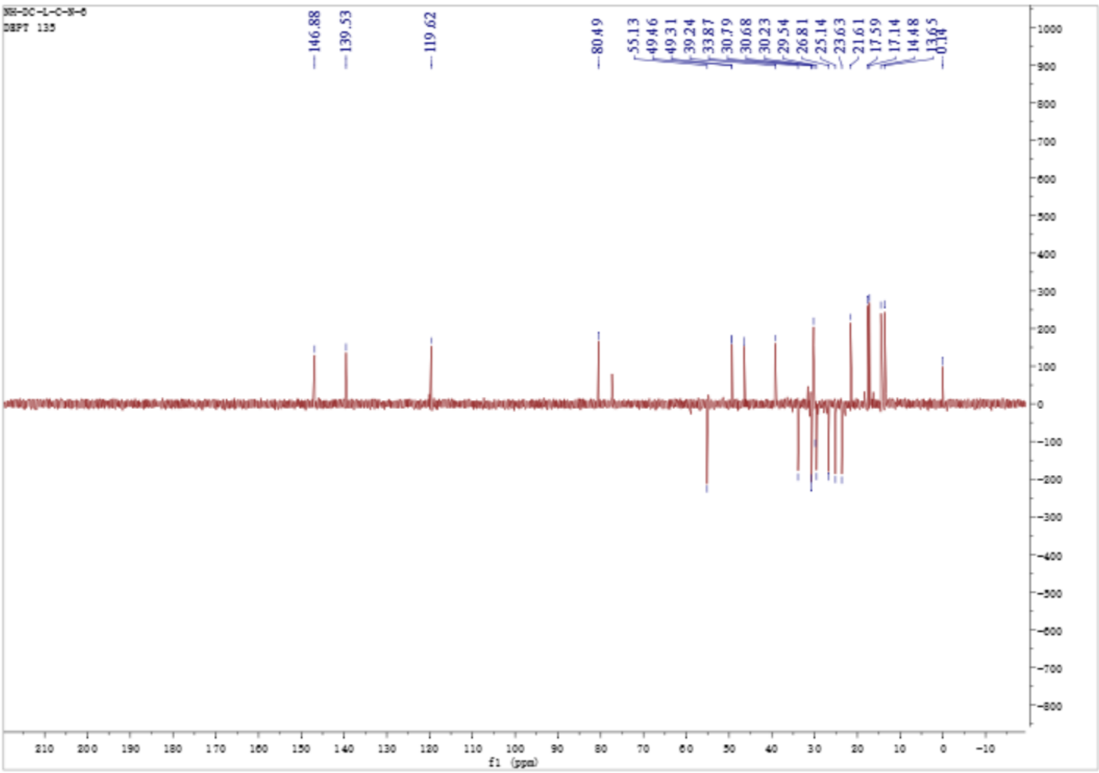

Figure S6. The DEPT spectrum of 1 in CDCl3

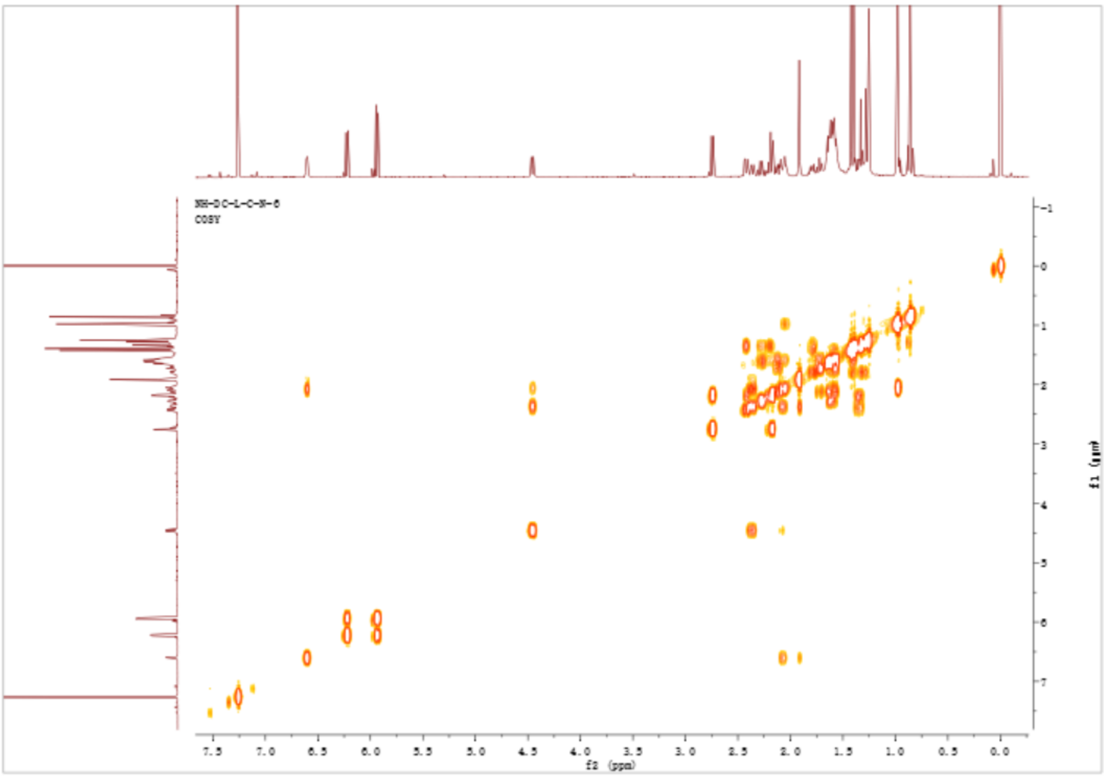

Figure S7. The ^{1}H-^{1}H COSY spectrum of 1 in CDCl3

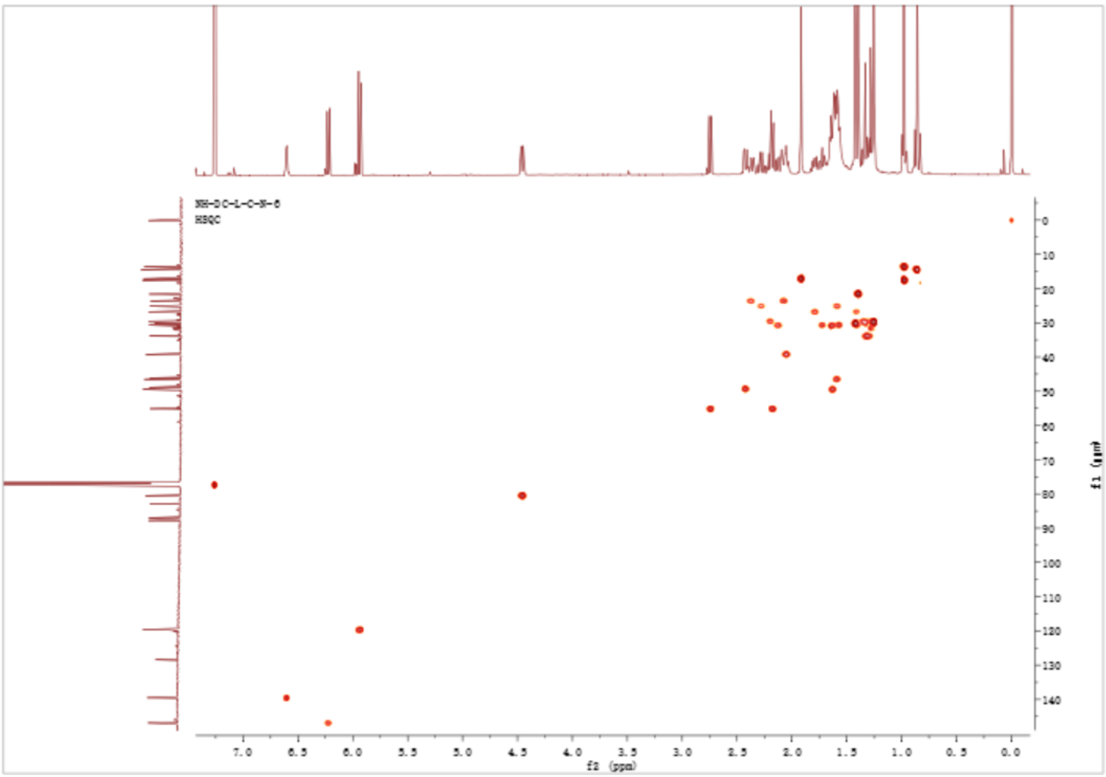

Figure S8. The HSQC spectrum of 1 in $CDCl_3$

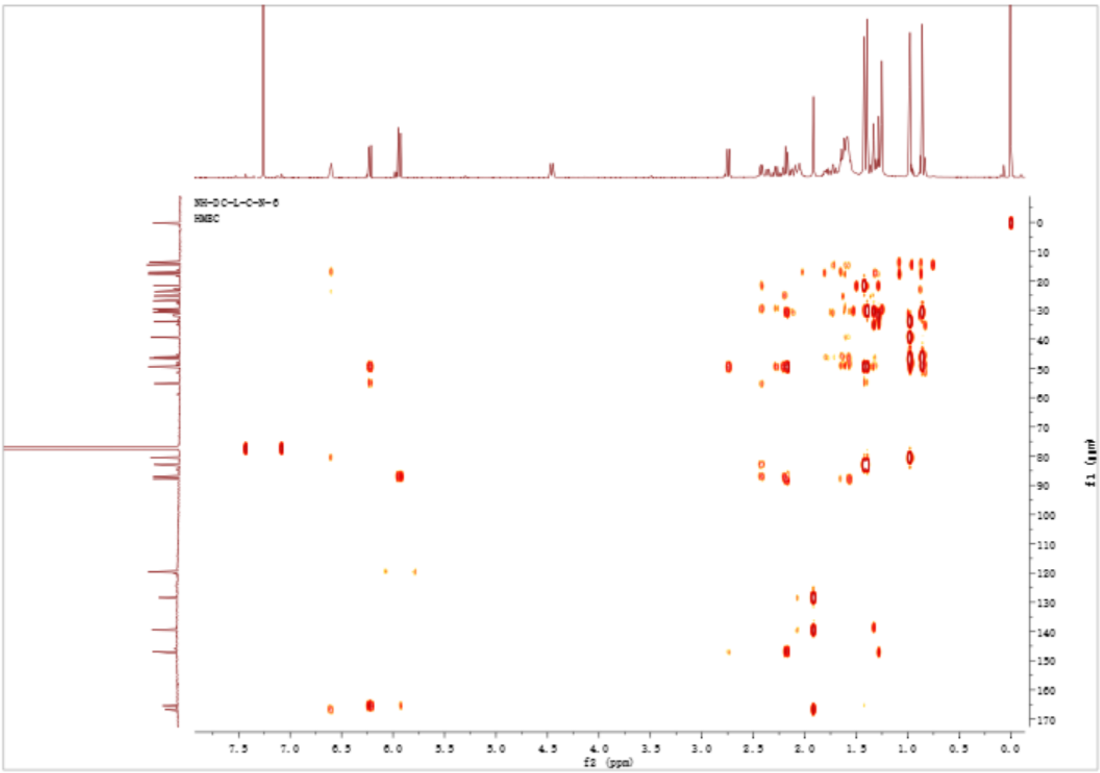

Figure S9. The HMBC spectrum of 1 in $CDCl_3$

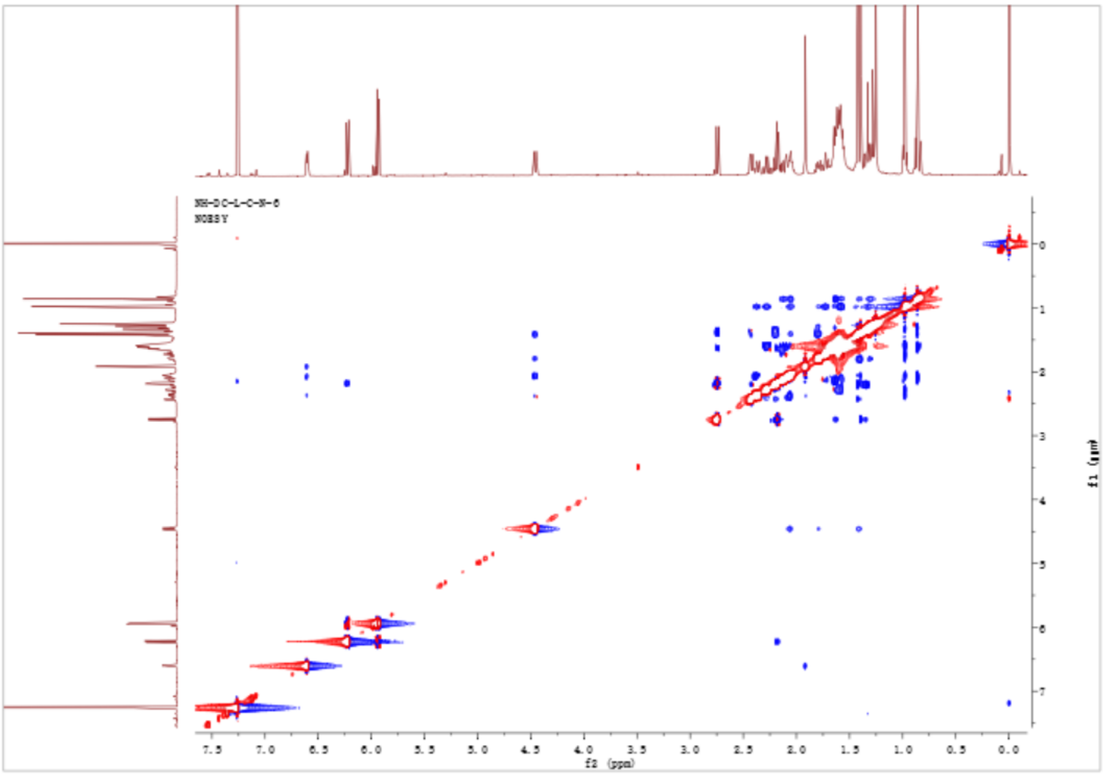

Figure S10. The ROESY spectrum of 1 in $CDCl_3$

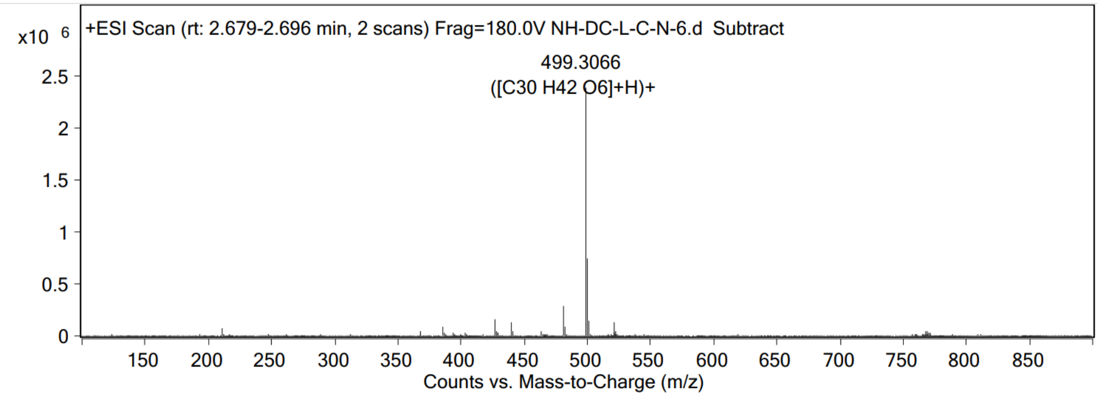

Formula Calculator Results

Formula	Best	Mass	Tgt Mass	Diff (ppm)	Ion Species	Score
C30 H42 O6	True	498.2995	498.2981	-2.65	C30 H43 O6	95.78

Figure S11. The HRESIMS spectrum of 1

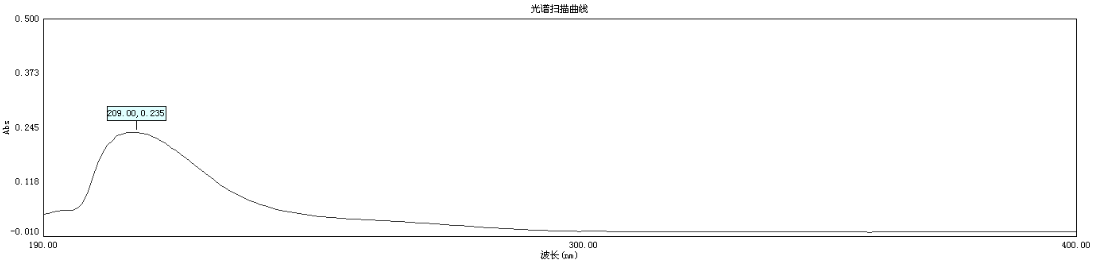

Figure S12. The UV spectrum of 1 in CH_2Cl_2

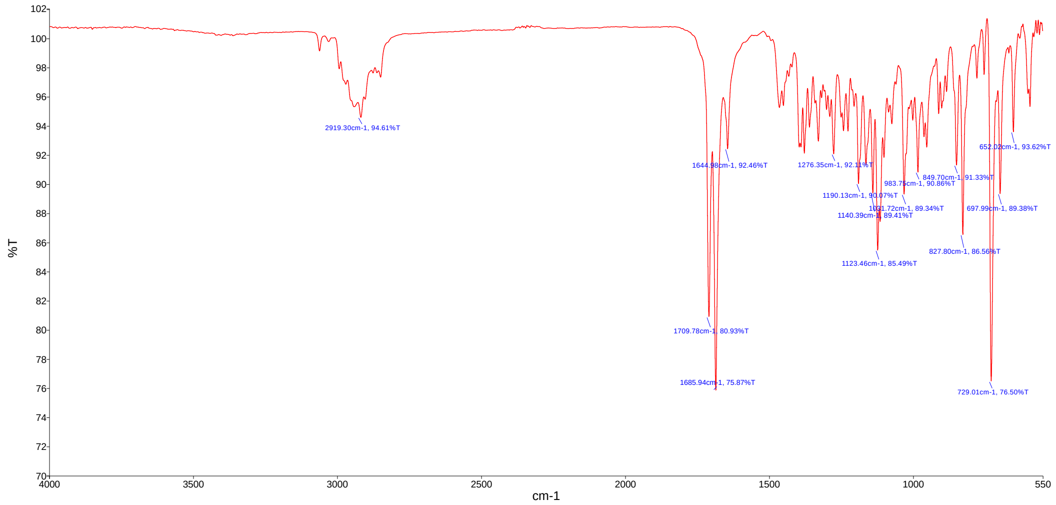

Figure S13. The IR spectrum of 1

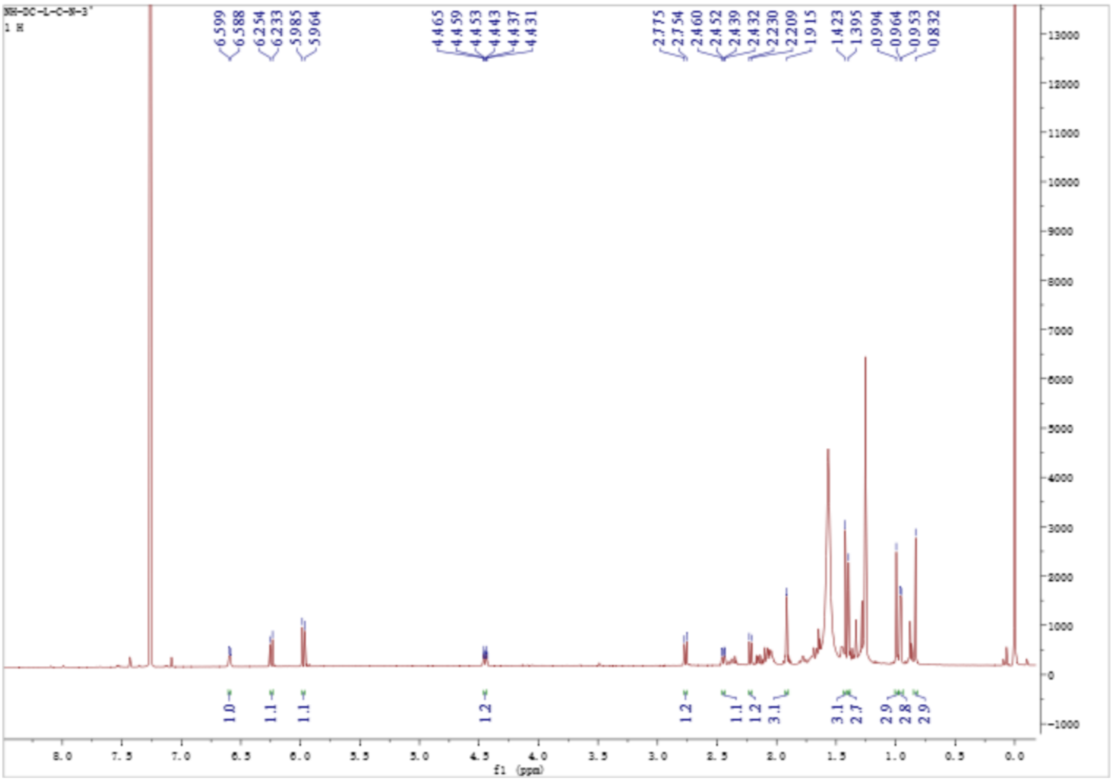

Figure S14. The ^{1}H NMR spectrum of 2 in $CDCl_3$

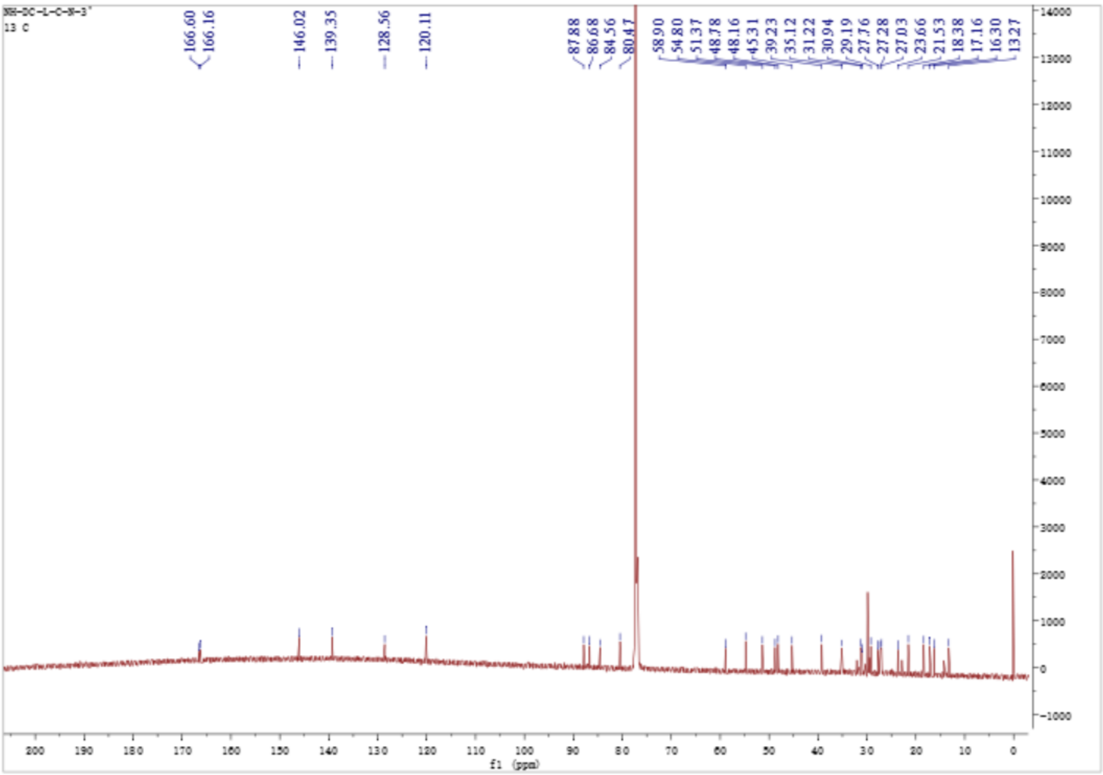

Figure S15. The ^{13}C NMR spectrum of 2 in $CDCl_3$

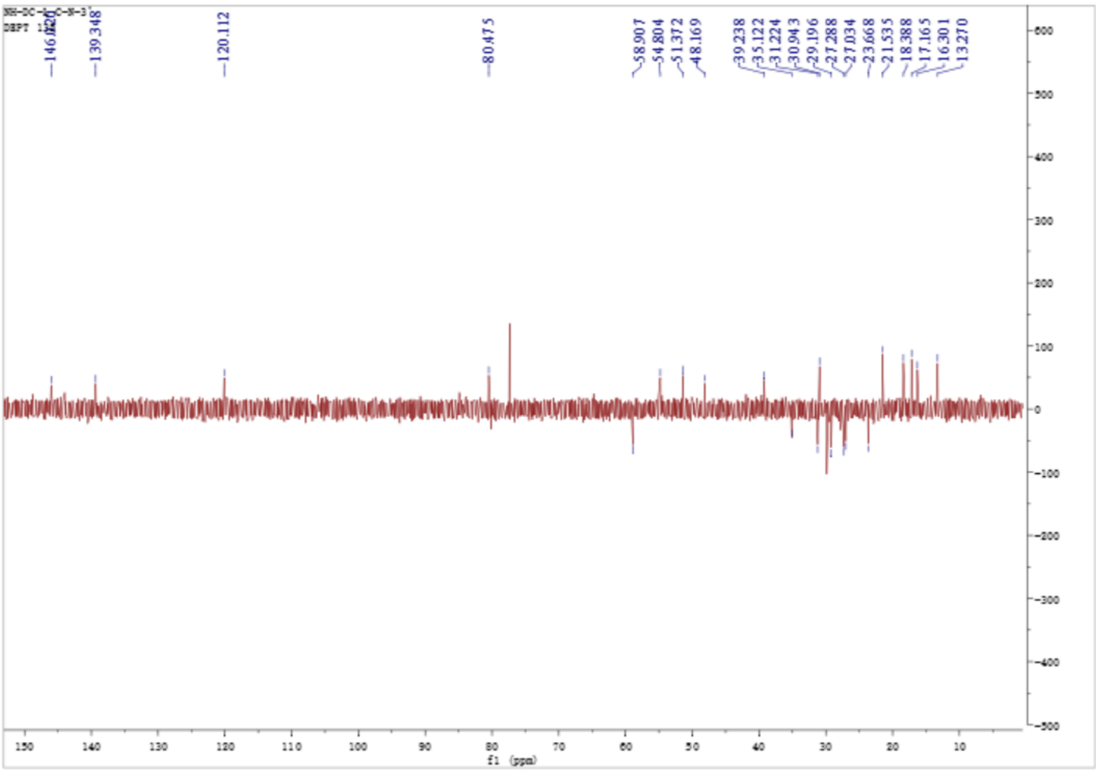

Figure S16. The DEPT spectrum of 2 in $CDCl_3$

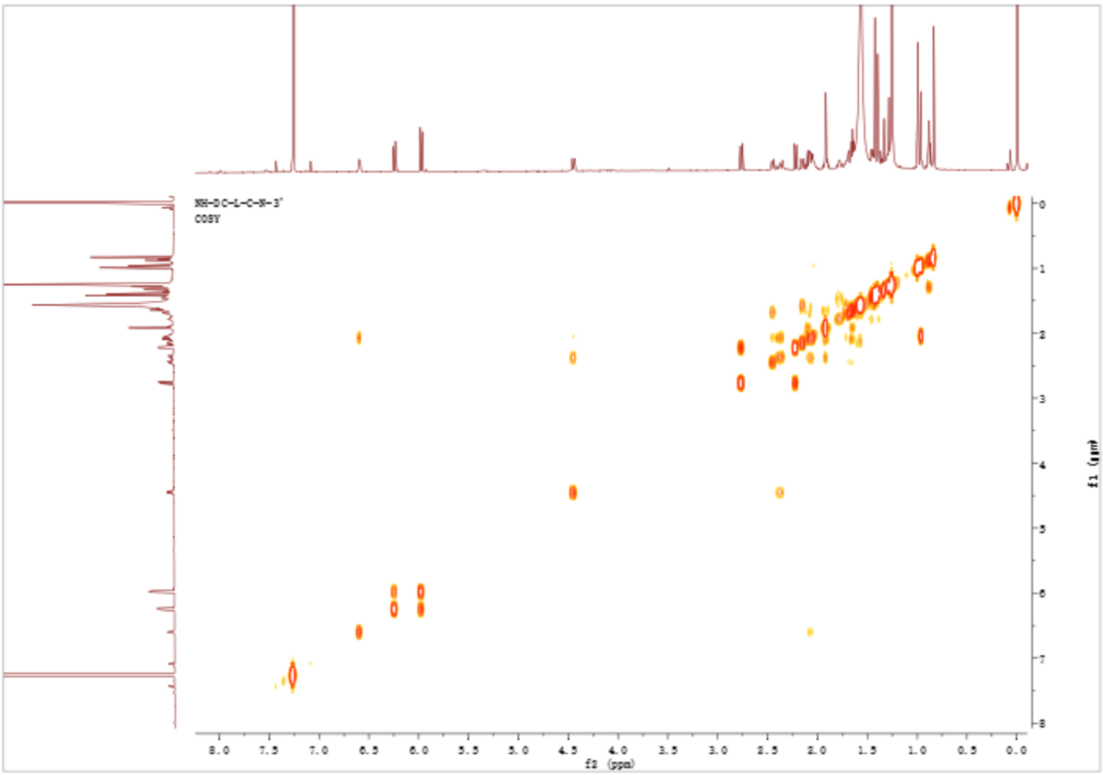

Figure S17. The ^{1}H-^{1}H COSY spectrum of 2 in $CDCl_3$

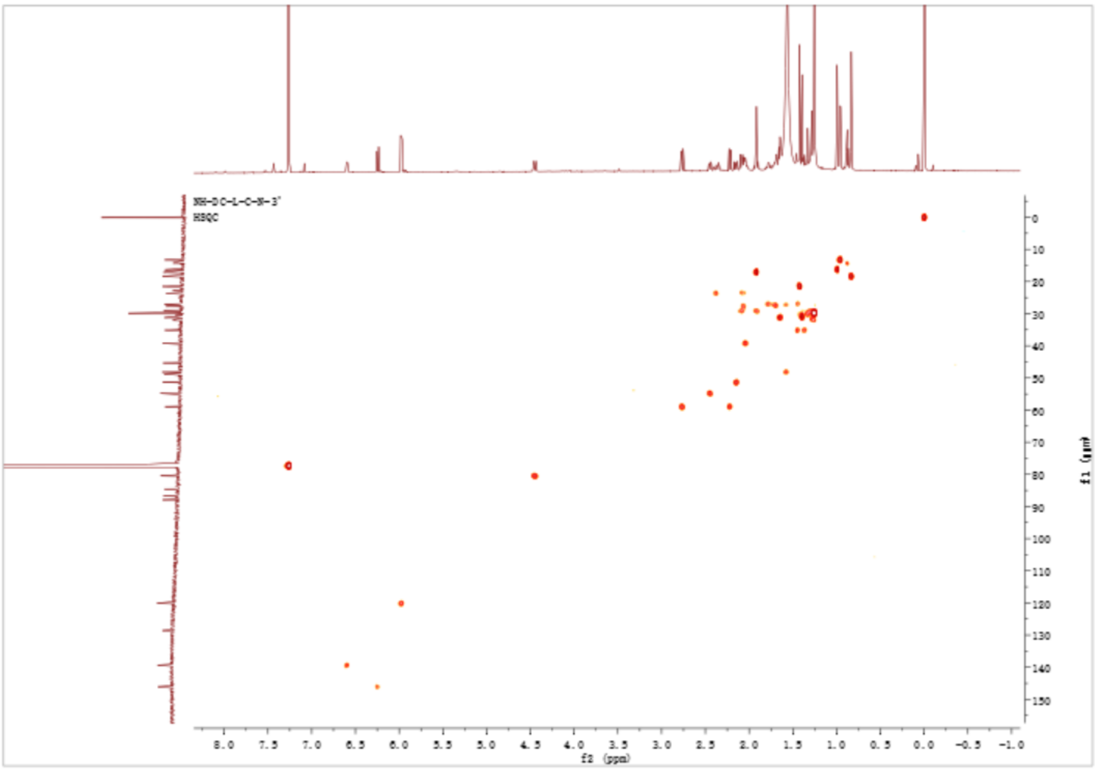

Figure S18. The HSQC spectrum of 2 in $CDCl_3$

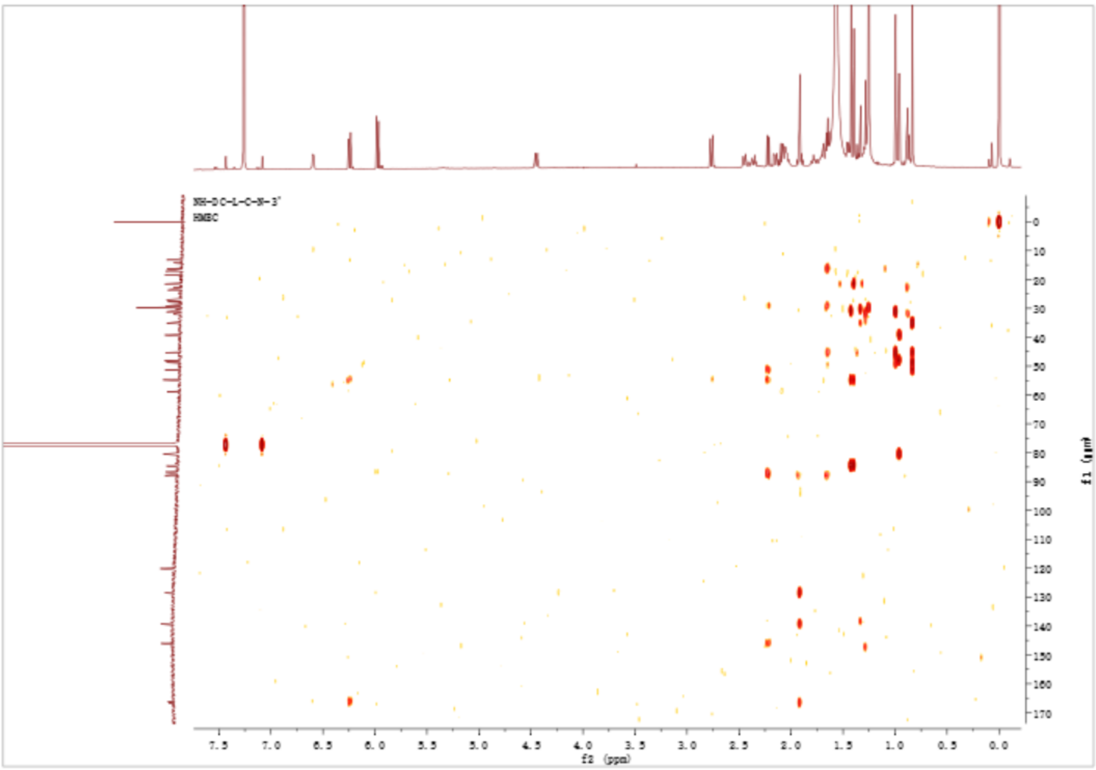

Figure S19. The HMBC spectrum of 2 in $CDCl_3$

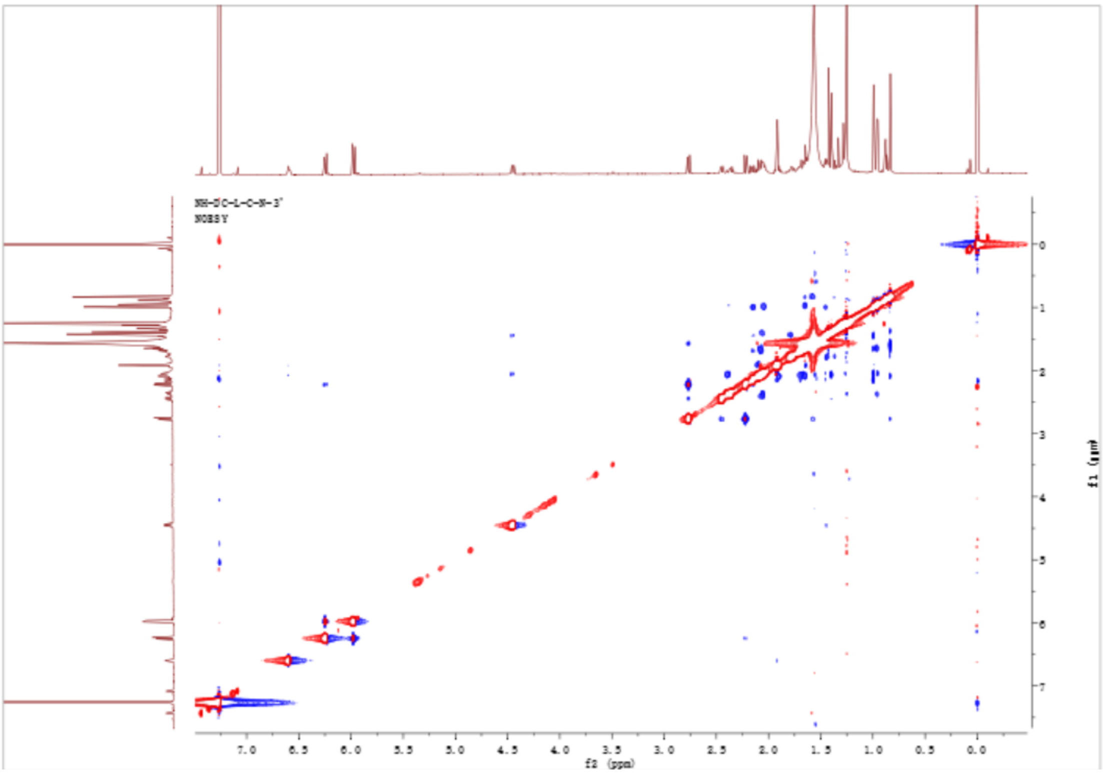

Figure S20. The ROESY spectrum of 2 in $CDCl_3$

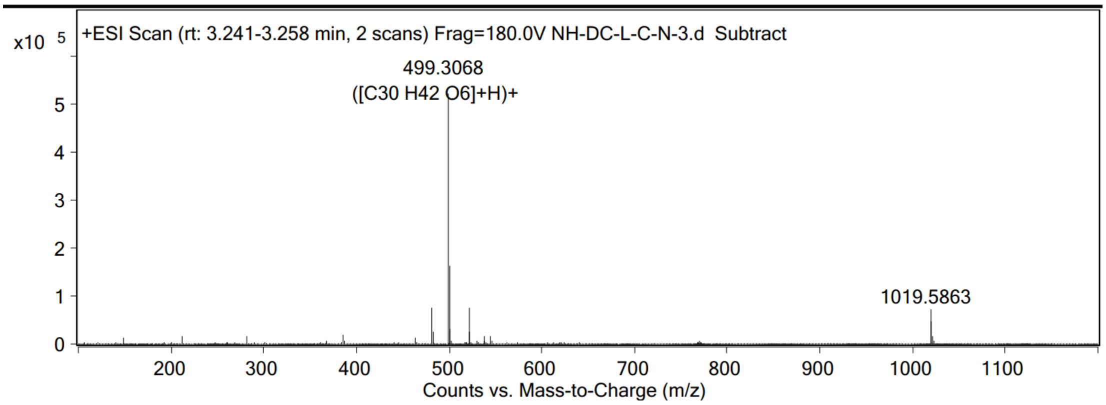

Formula Calculator Results

Formula	Best	Mass	Tgt Mass	Diff (ppm)	Ion Species	Score
C30 H42 O6	True	498.2994	498.2981	-2.5	C30 H43 O6	95.88

Figure S21. The HRESIMS spectrum of 2

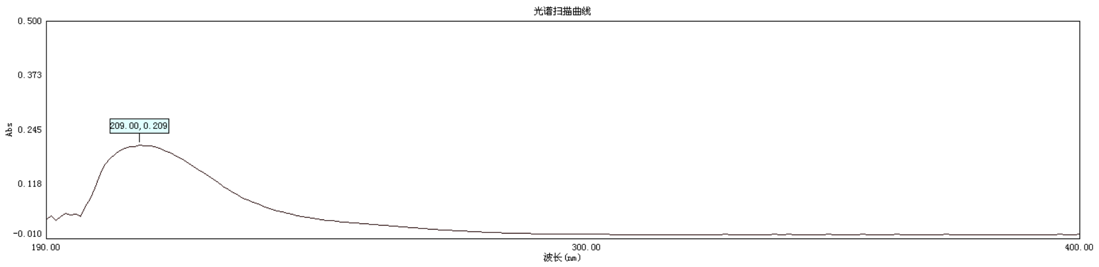

Figure S22. The UV spectrum of 2 in CH_2Cl_2

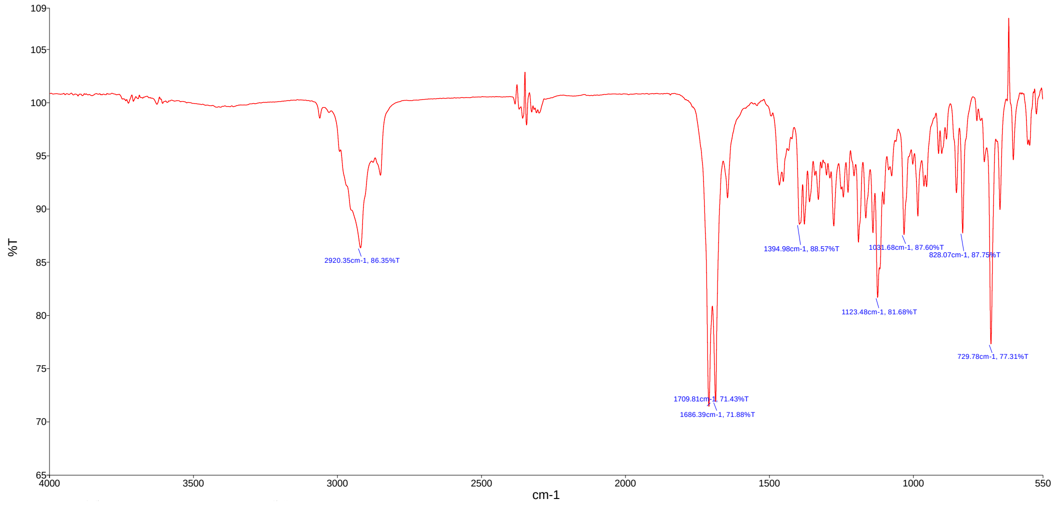

Figure S23. The IR spectrum of 2

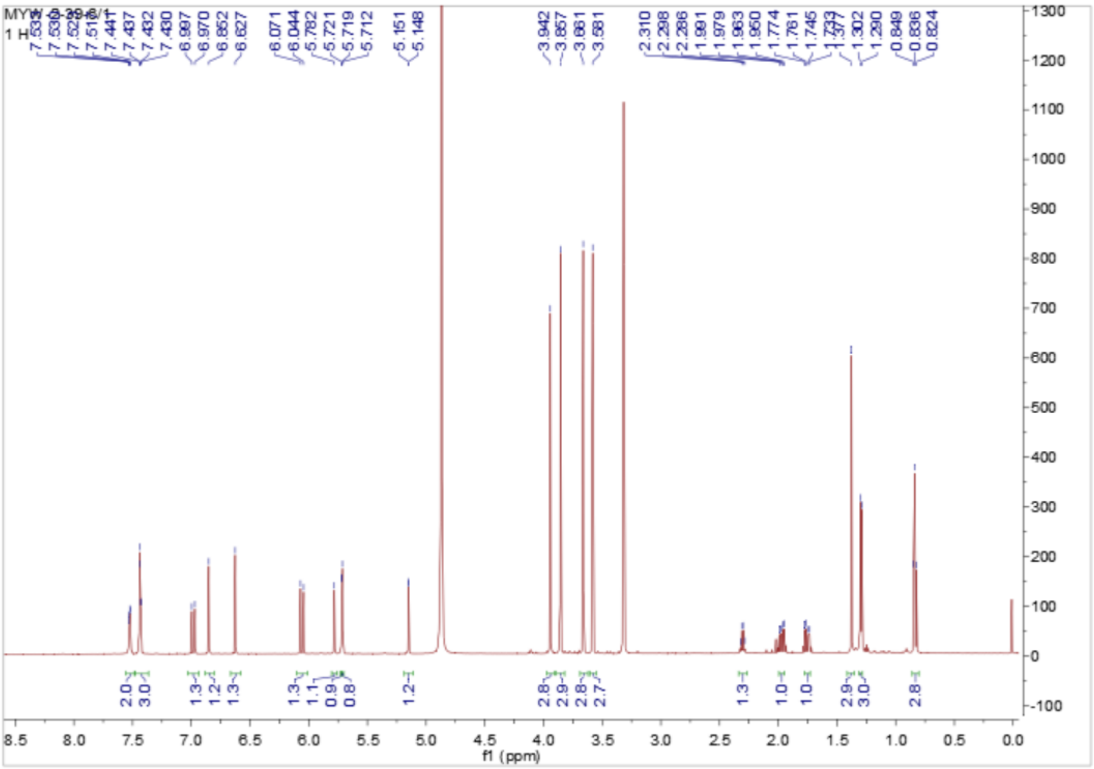

Figure S24. The ^{1}H NMR spectrum of 3 in CD_3OD

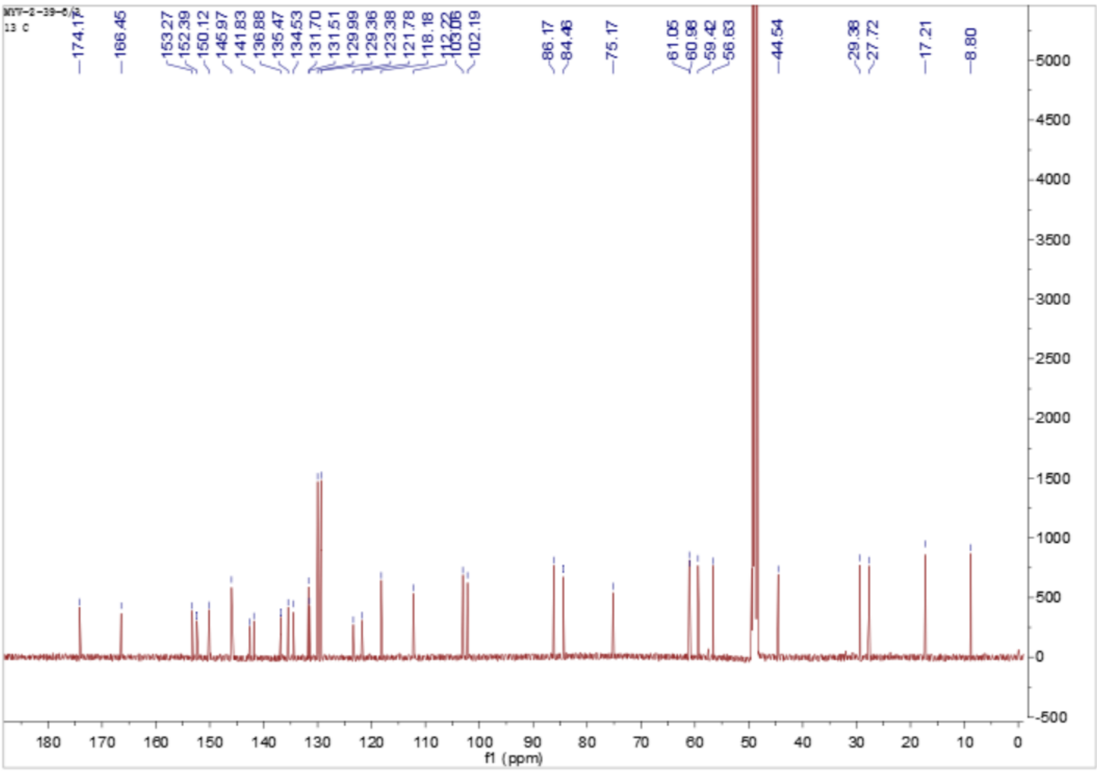

Figure S25. The ^{13}C NMR spectrum of 3 in CD_3OD

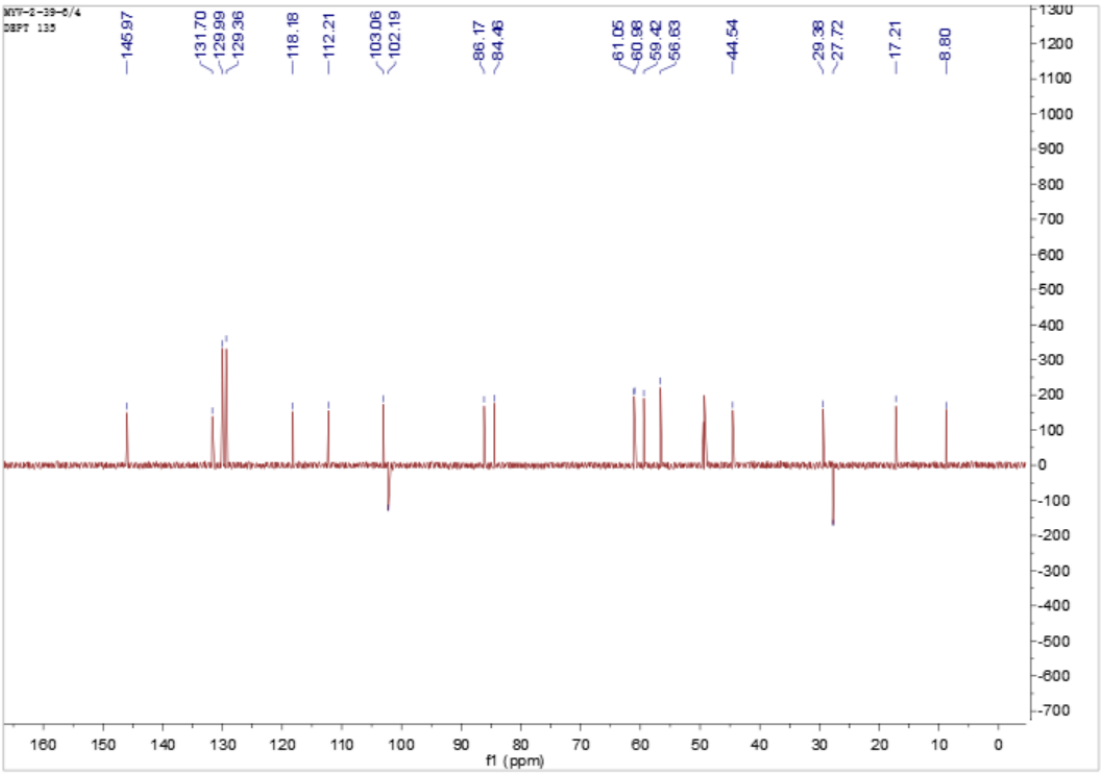

Figure S26. The DEPT spectrum of 3 in CD_3OD

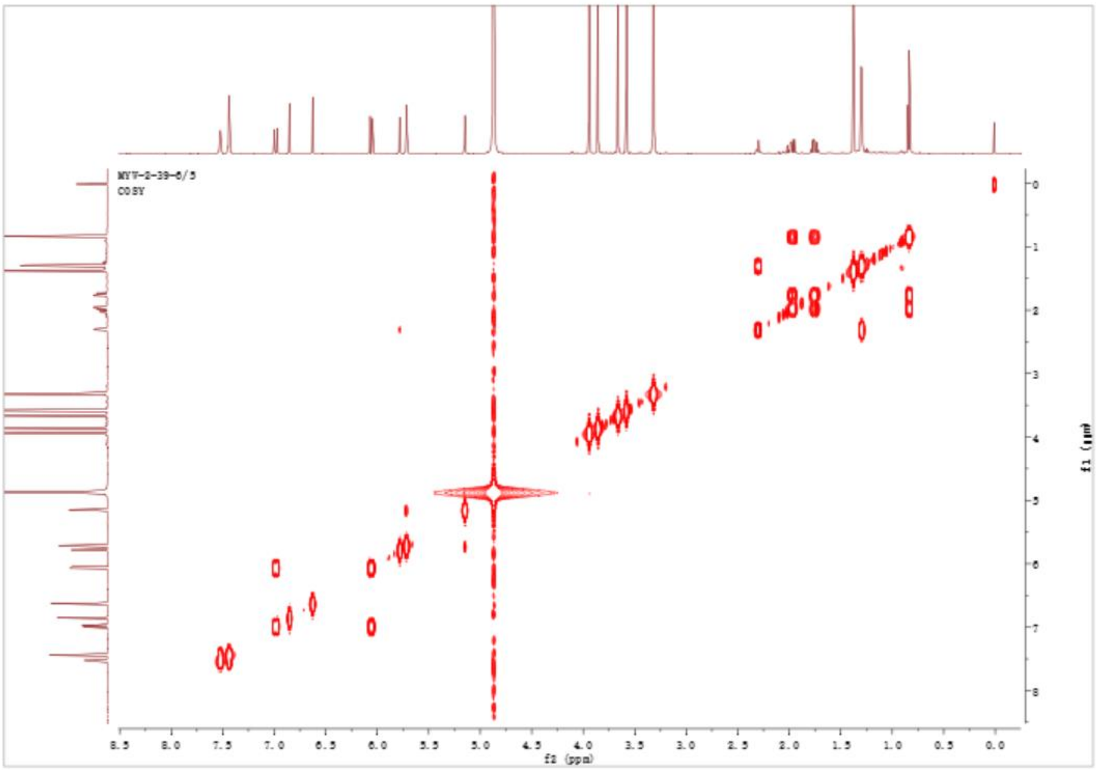

Figure S27. The ^1H-^1H-COSY spectrum of 3 in CD_3OD

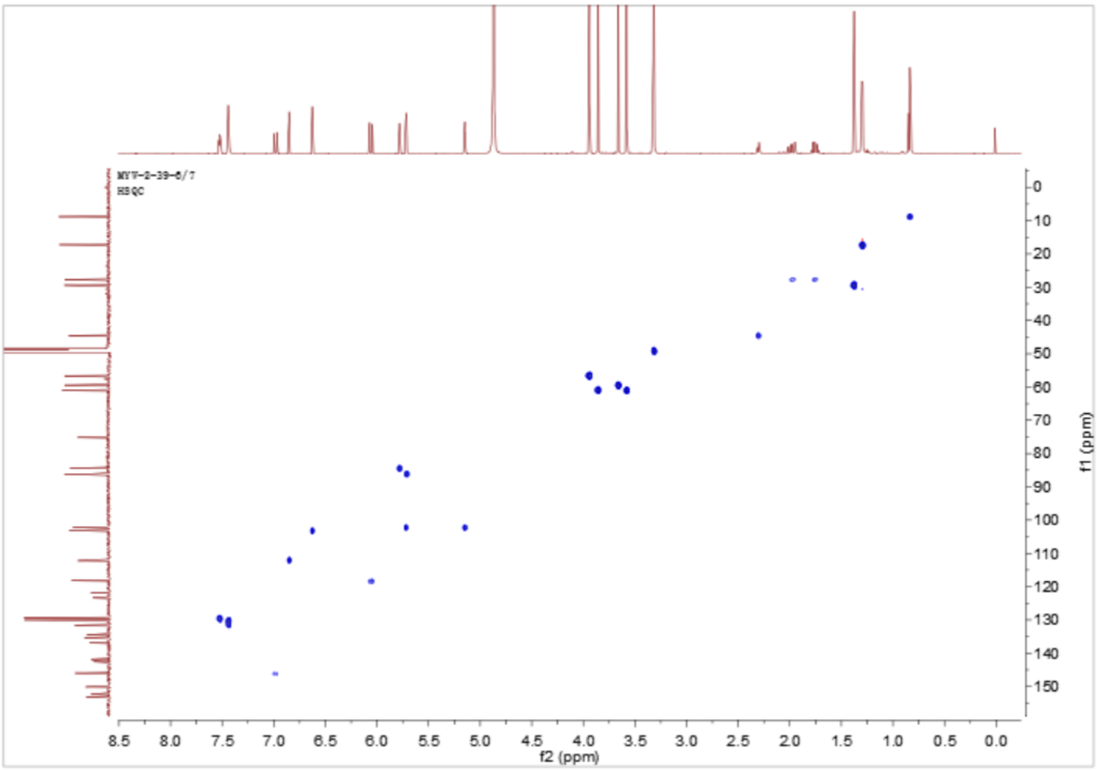

Figure S28. The HSQC spectrum of 3 in CD_3OD

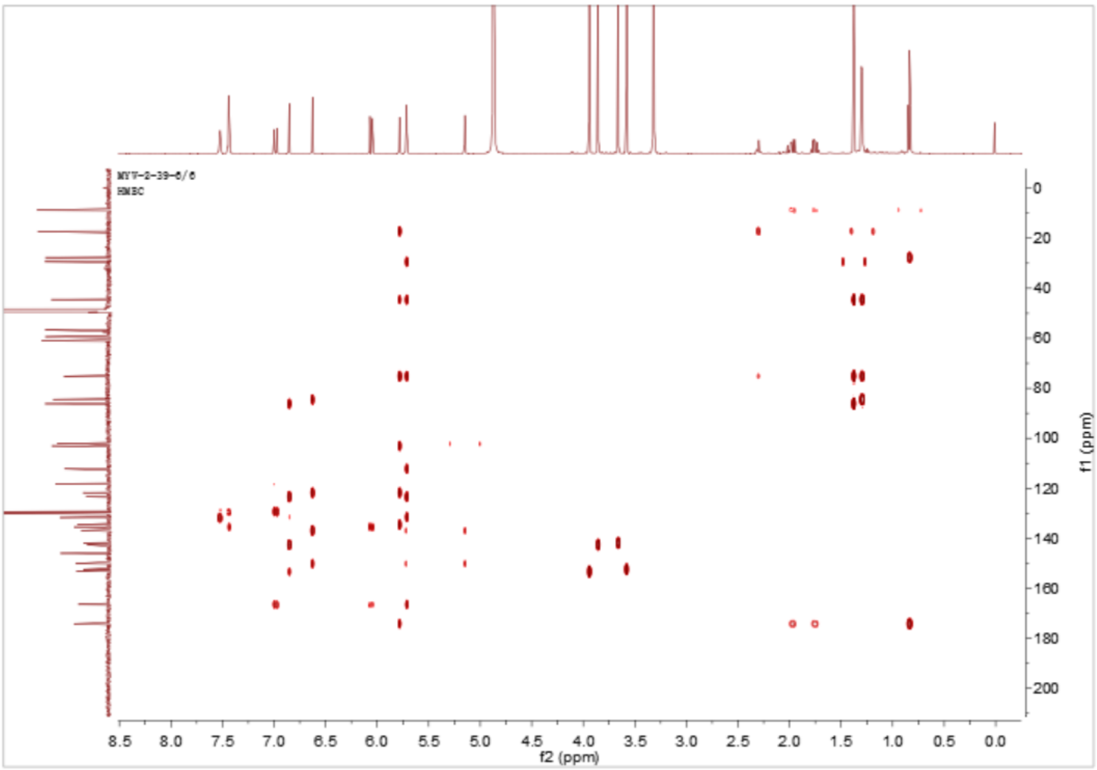

Figure S29. The HMBC spectrum of 3 in CD_3OD

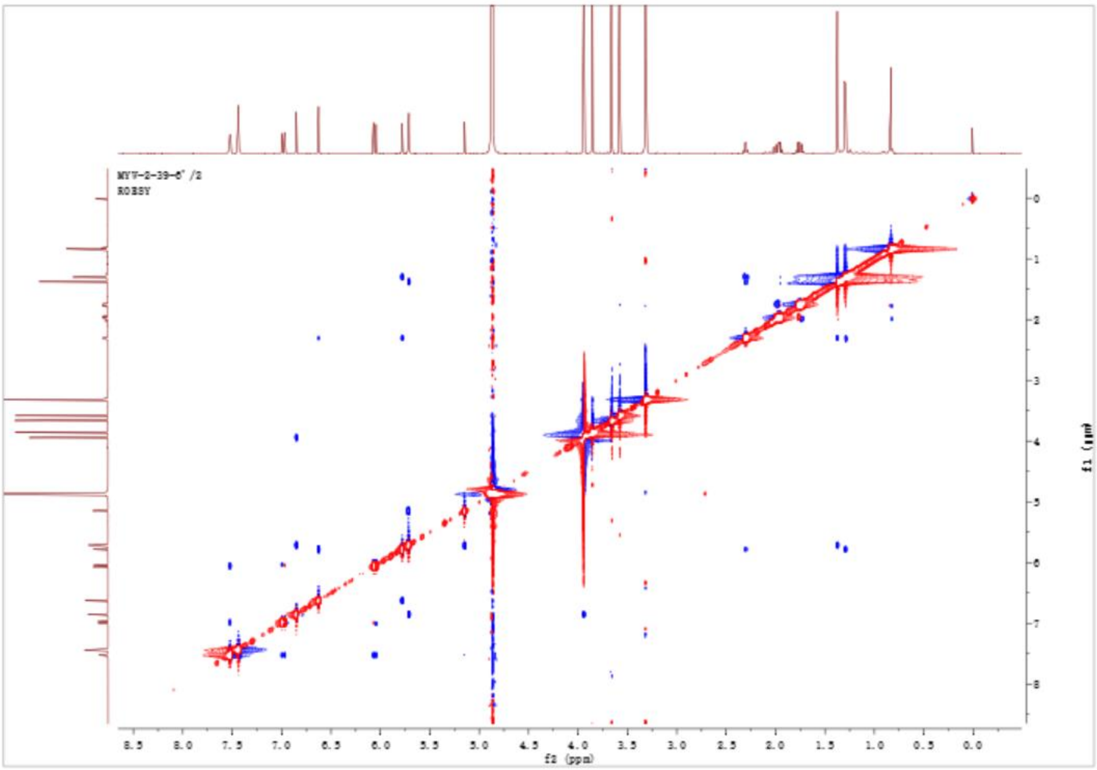

Figure S30. The ROESY spectrum of 3 in CD_3OD

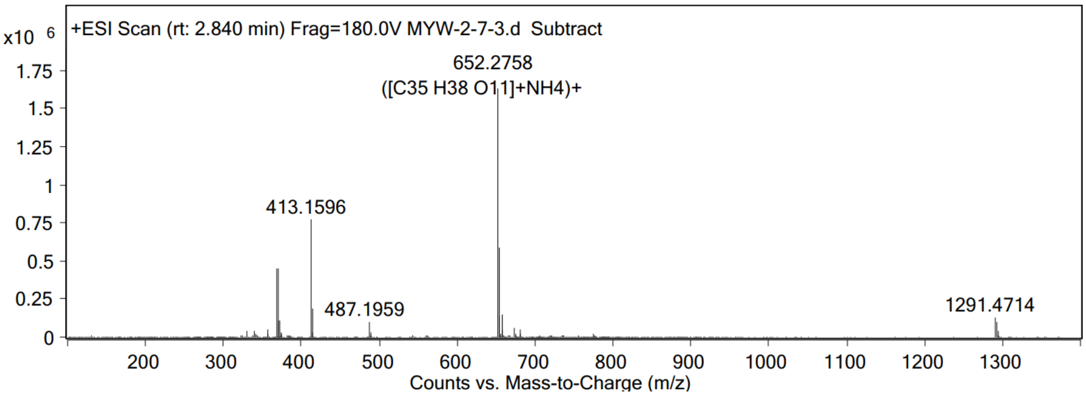

Formula Calculator Results

Formula	Best	Mass	Tgt Mass	Diff (ppm)	Ion Species	Score
C35 H38 O11	True	634.2419	634.2414	-0.83	C35 H42 N O11	98.44

Figure S31. The HRESIMS spectrum of 3 in CD_3OD

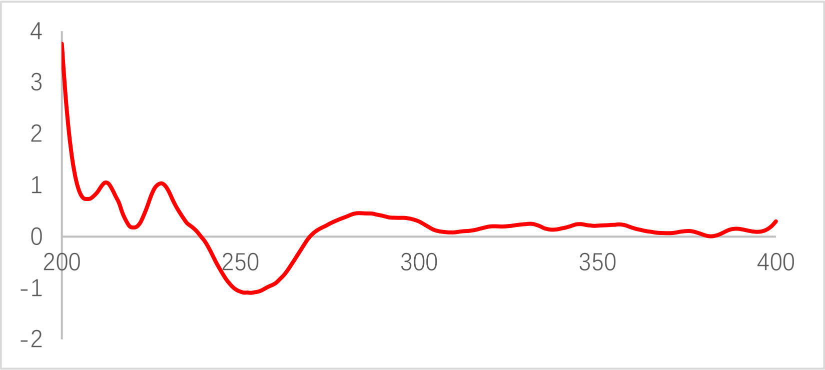

Figure S32. The ECD spectrum of 3 in MeOH

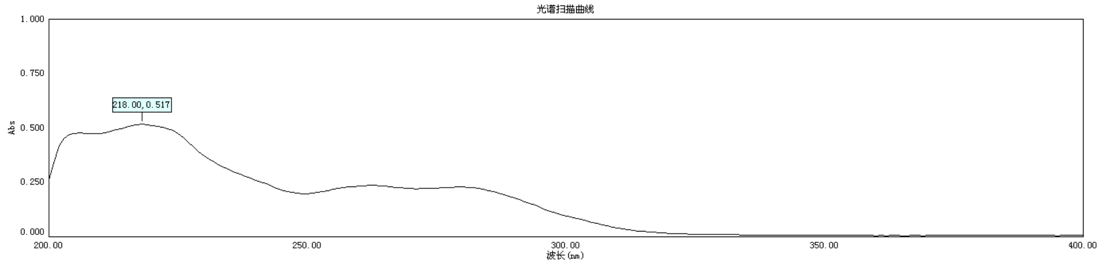

序号	峰/谷	波长(nm)	Abs	注释
1	峰	218.00	0.517	

Figure S33. The UV spectrum of 3 in MeOH

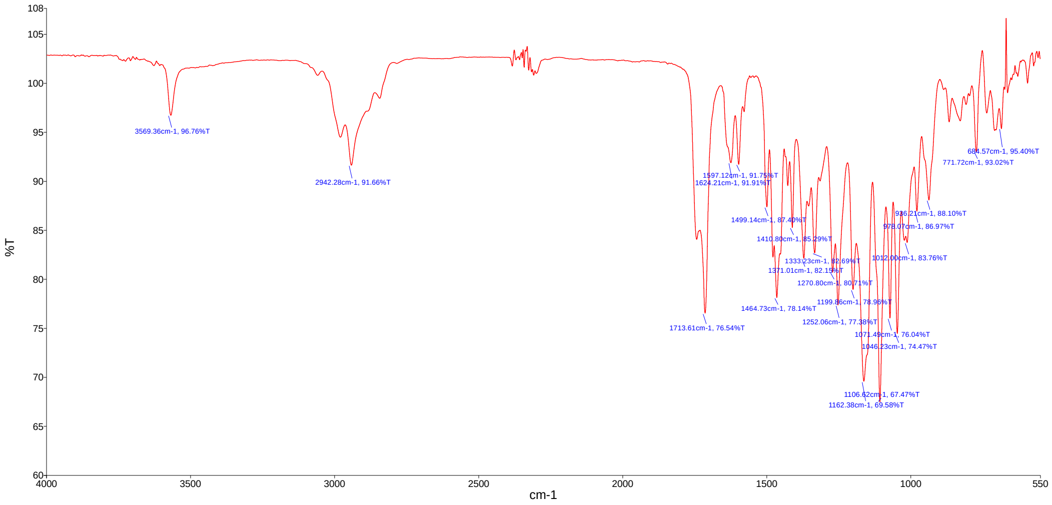

Figure S34. The IR spectrum of 3

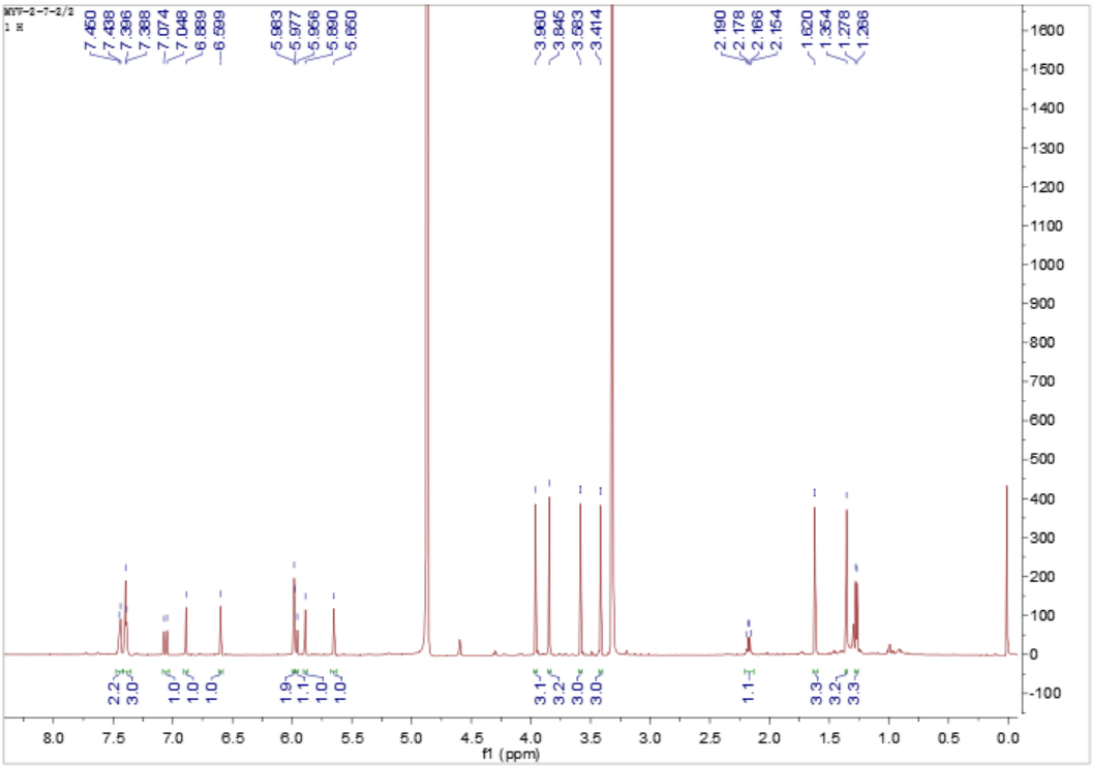

Figure S35. The ^{1}H NMR spectrum of 4 in CD_3OD

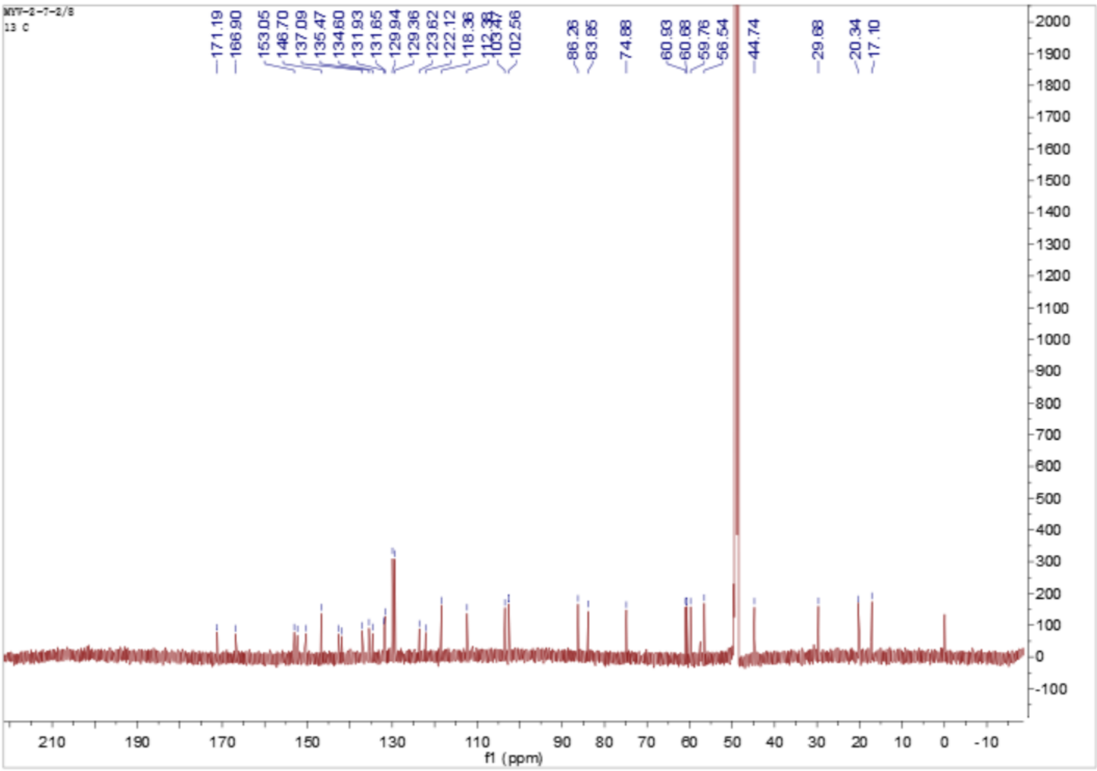

Figure S36. The ^{13}C NMR spectrum of 4 in CD_3OD

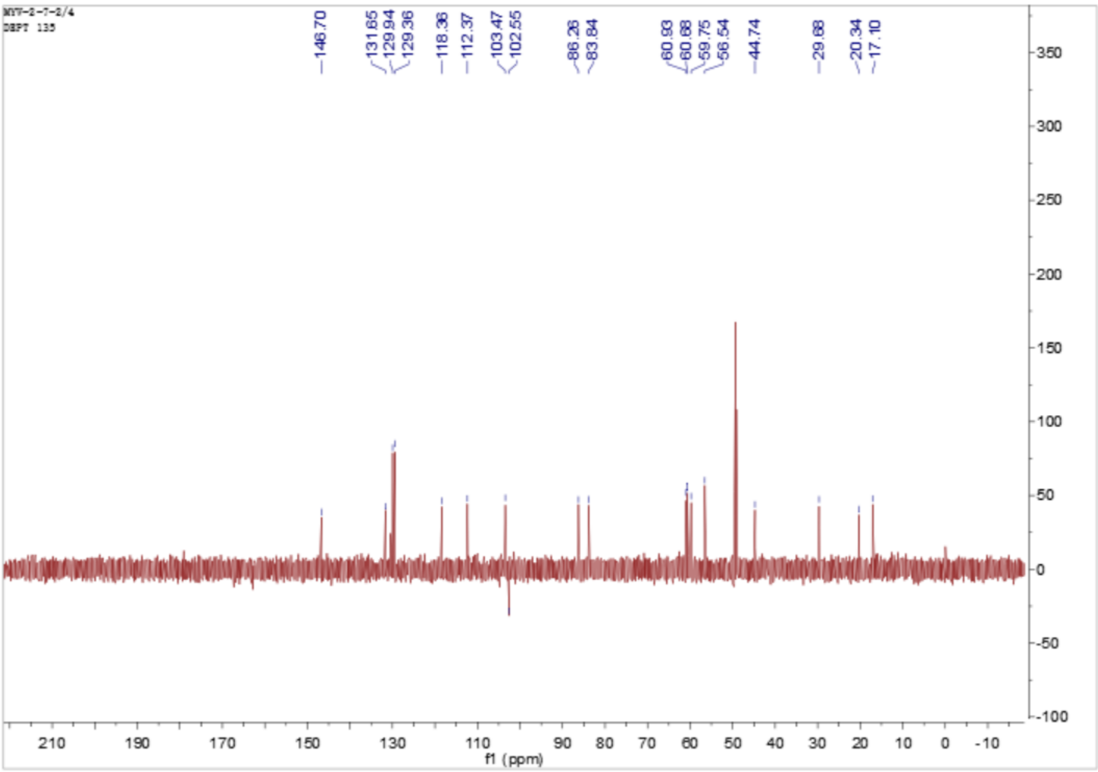

Figure S37. The DEPT spectrum of 4 in CD_3OD

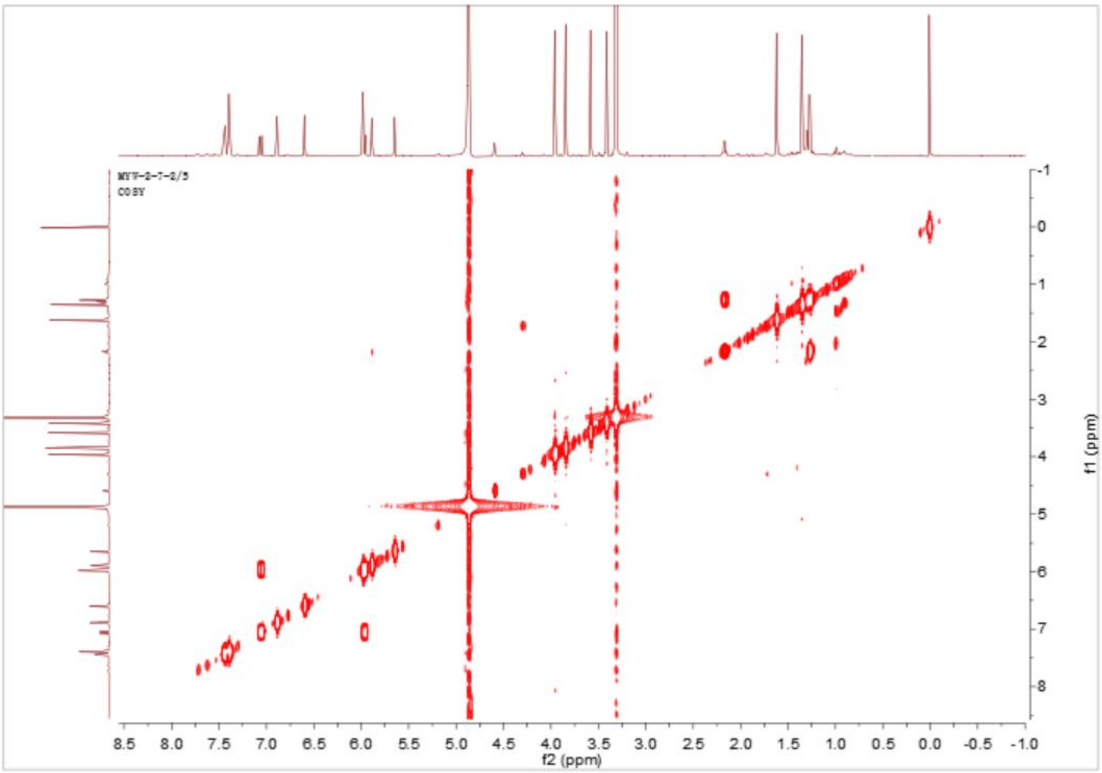

Figure S38. The ^1H-^1H COSY spectrum of 4 in CD_3OD

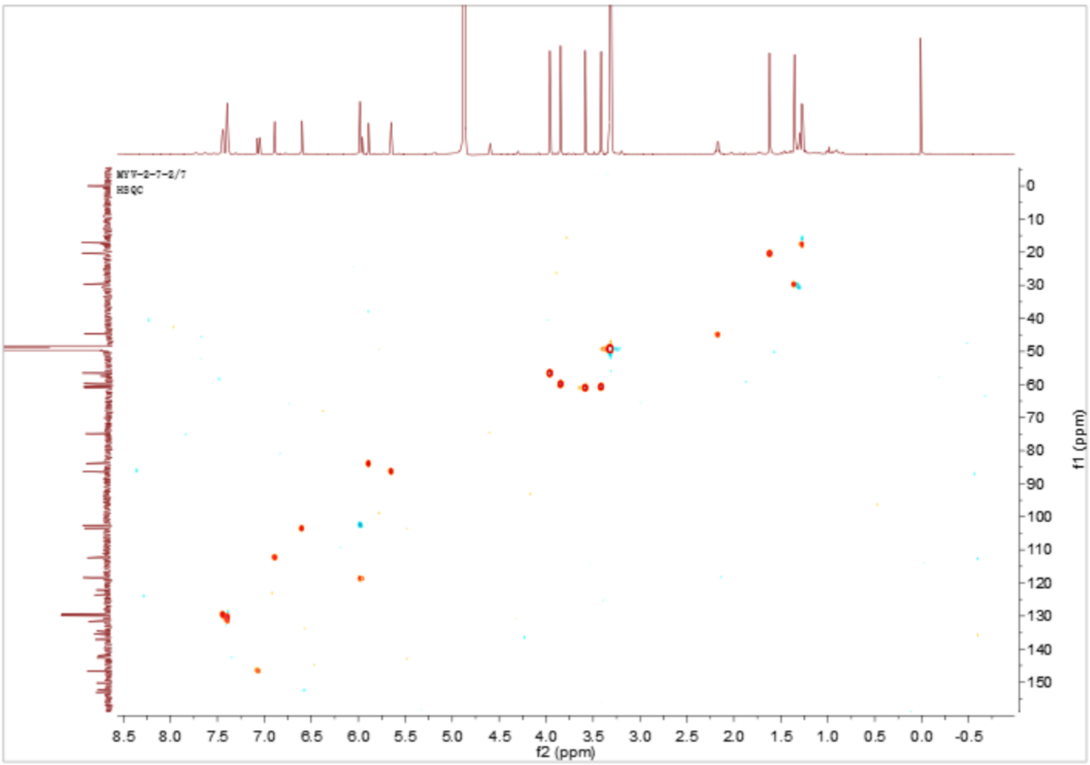

Figure S39. The HSQC spectrum of 4 in CD_3OD

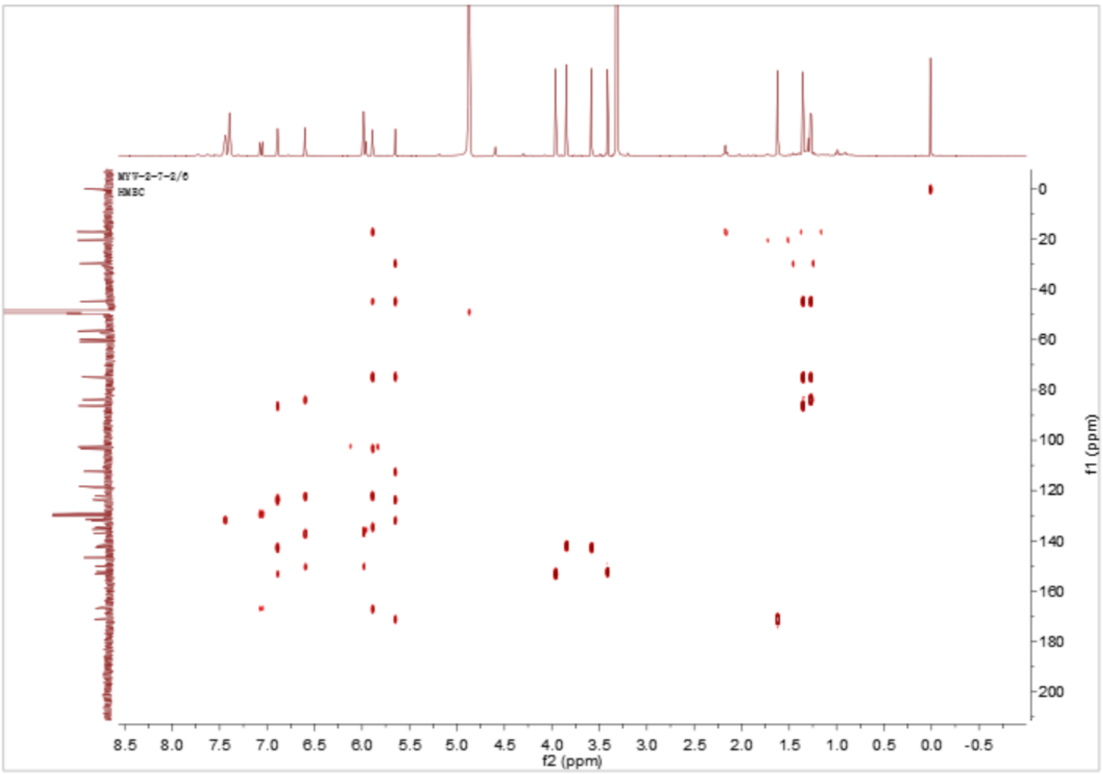

Figure S40. The HMBC spectrum of 4 in CD_3OD

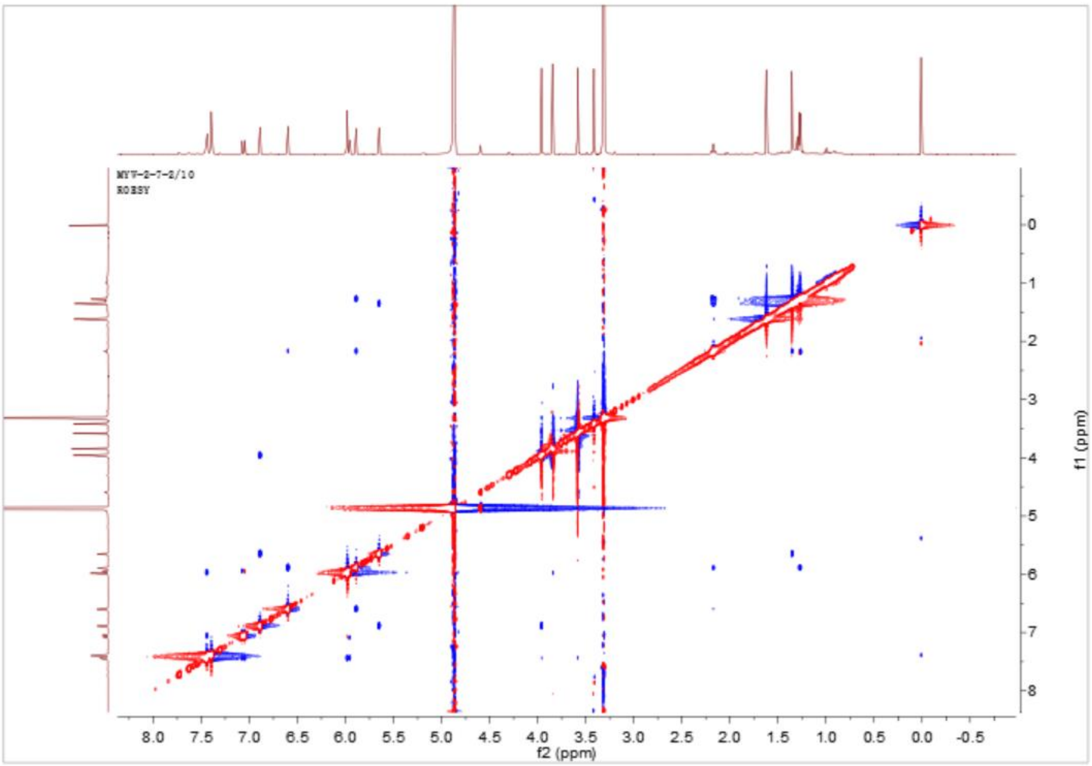

Figure S41. The ROSEY spectrum of 4 in CD_3OD

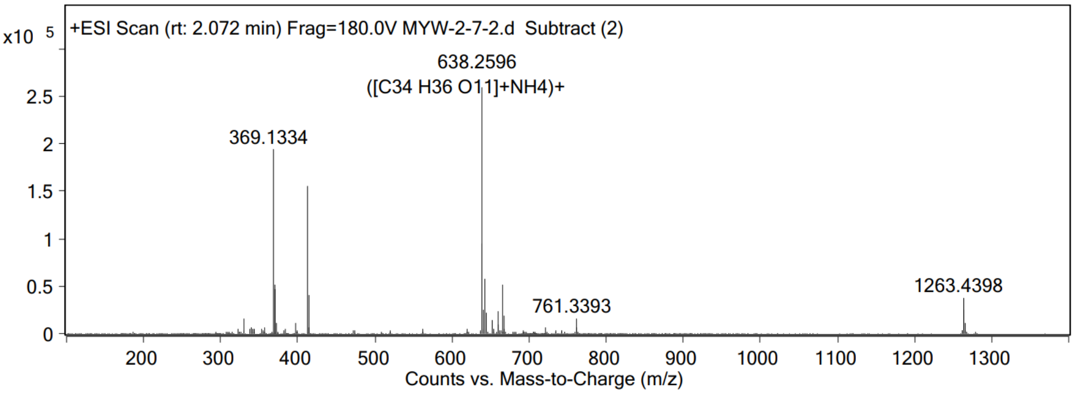

Formula Calculator Results

Formula	Best	Mass	Tgt Mass	Diff (ppm)	Ion Species	Score
C34 H36 O11	True	620.2258	620.2258	0.01	C34 H40 N O11	99.44

Figure S42. The HRESIMS spectrum of 4

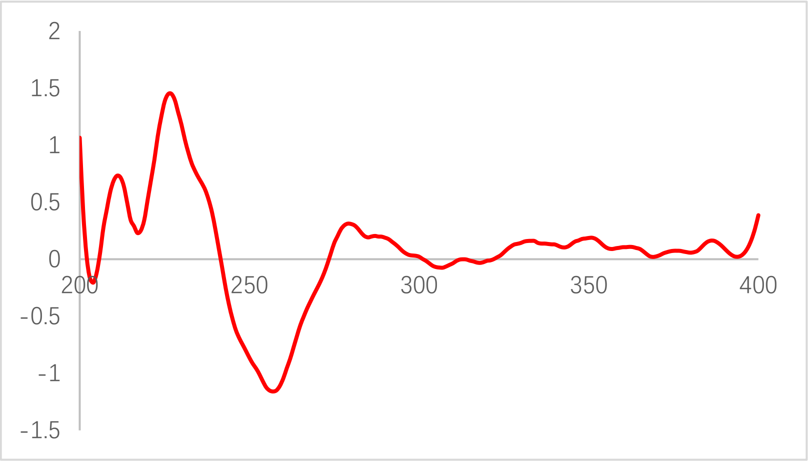

Figure S43. The ECD spectrum of 4 in MeOH

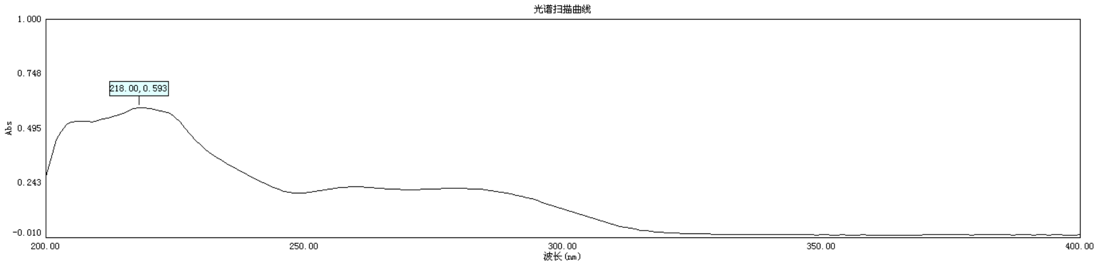

序号	峰/谷	波长(nm)	Abs	注释
1	峰	218.00	0.593	

Figure S44. The UV spectrum of 4 in MeOH

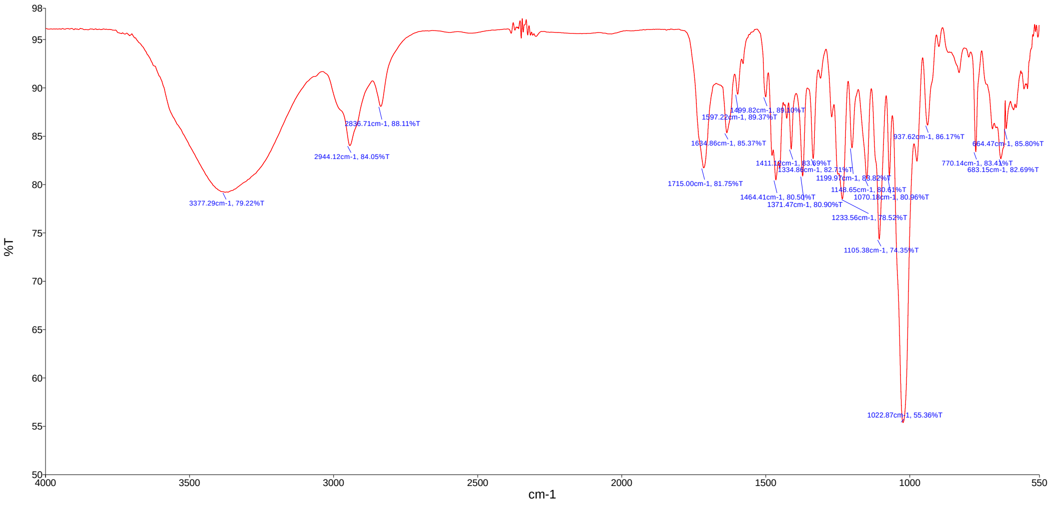

Figure S45. The IR spectrum of 4

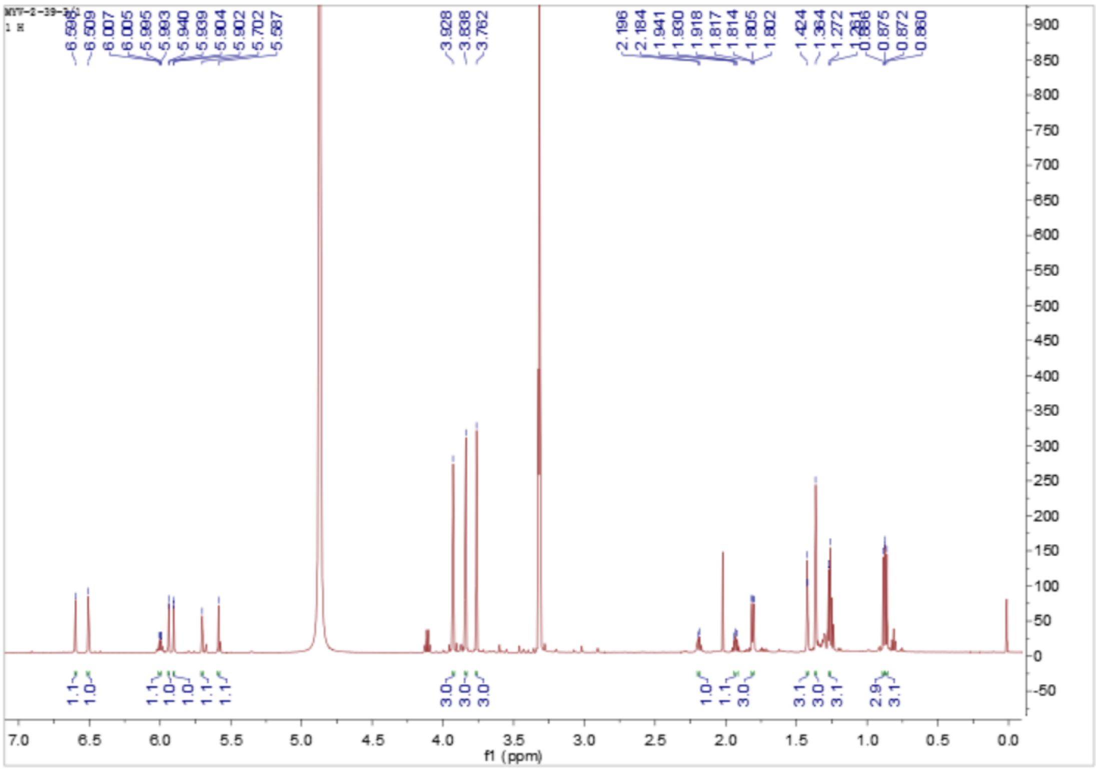

Figure S46. The ^{1}H NMR spectrum of 5 in CD_3OD

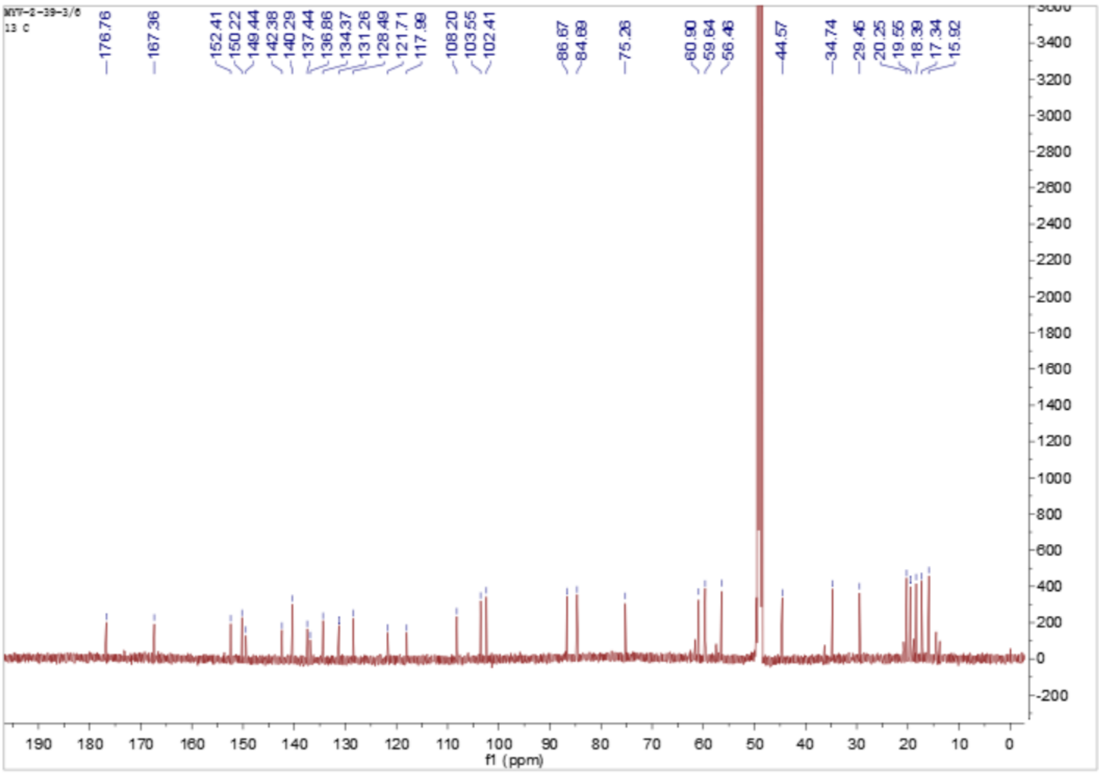

Figure S47. The ^{13}C NMR spectrum of 5 in CD_3OD

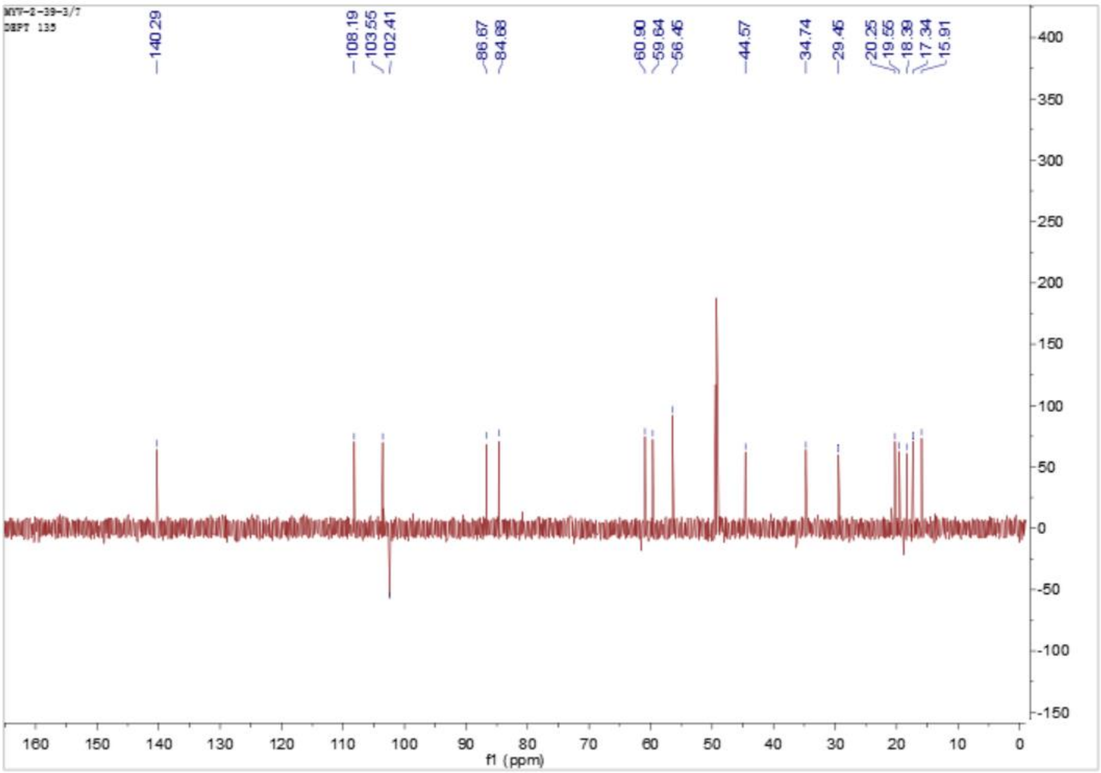

Figure S48. The DEPT spectrum of 5 in CD_3OD

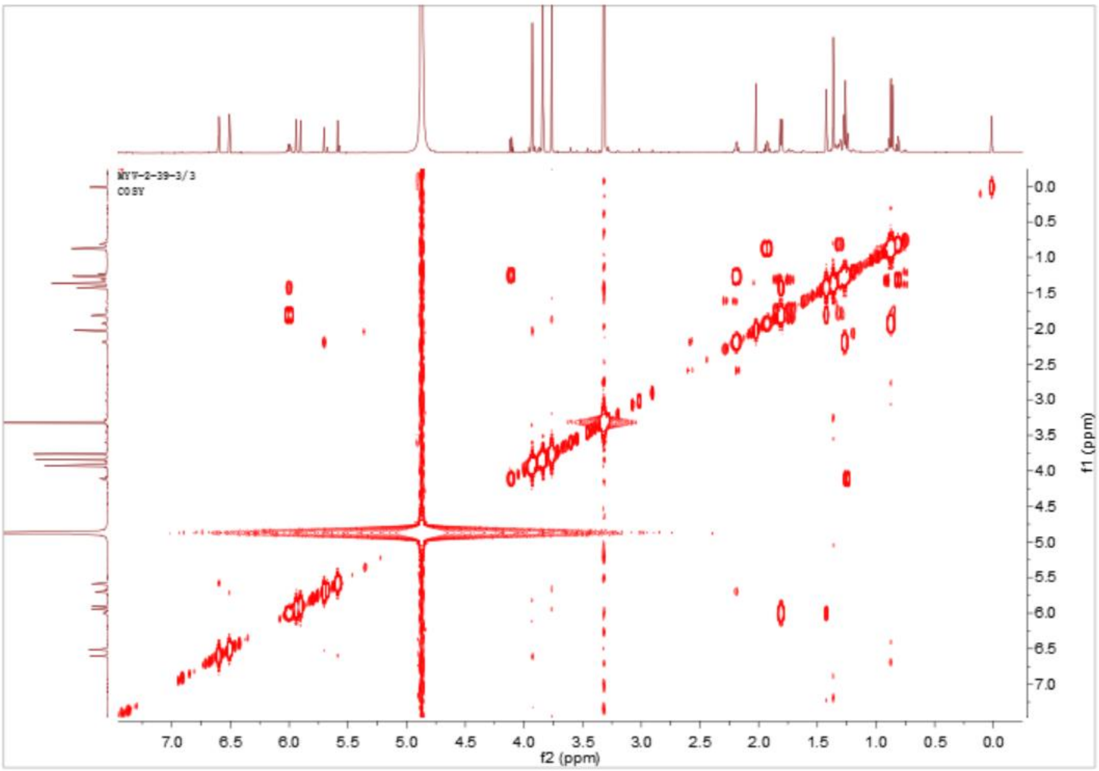

Figure S49. The ^1H-^1H COSY spectrum of 5 in CD_3OD

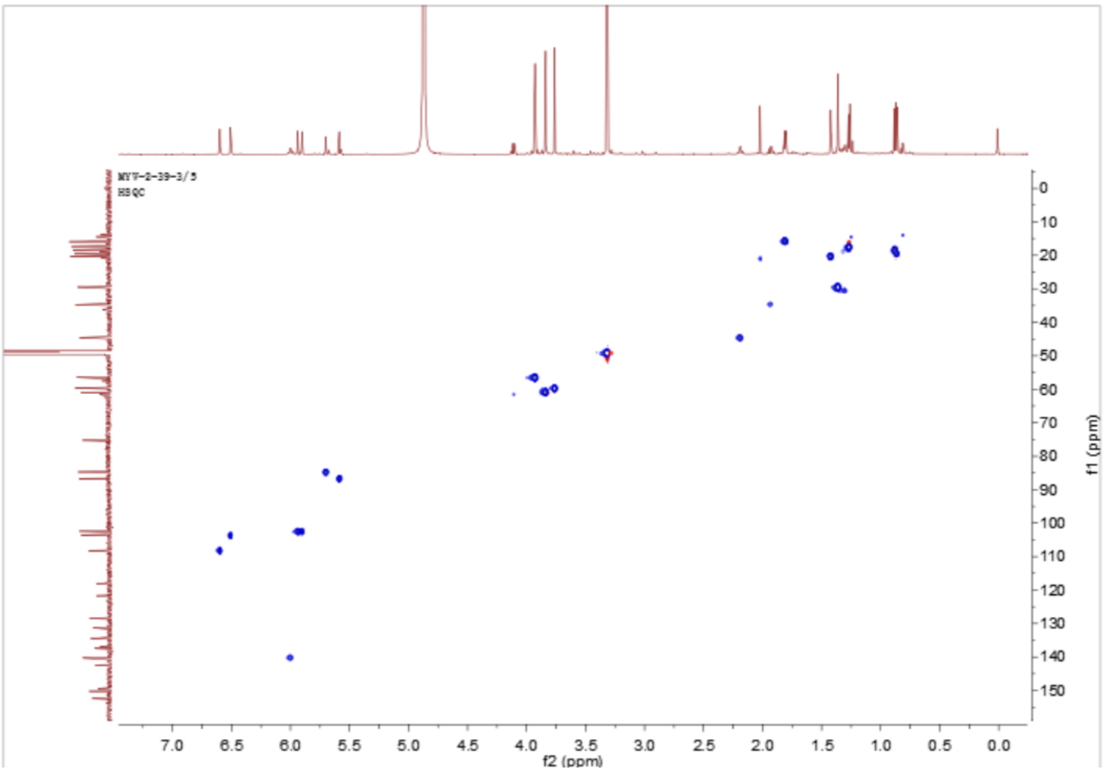

Figure S50. The HSQC spectrum of 5 in CD_3OD

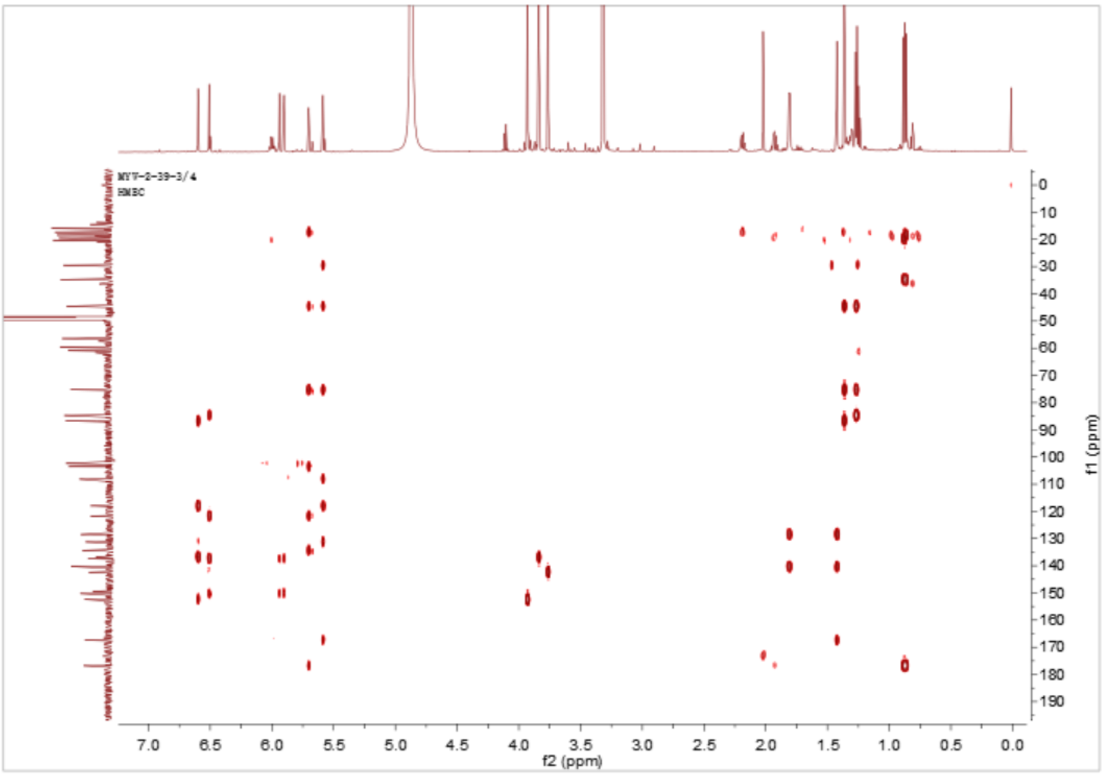

Figure S51. The HMBC spectrum of 5 in CD_3OD

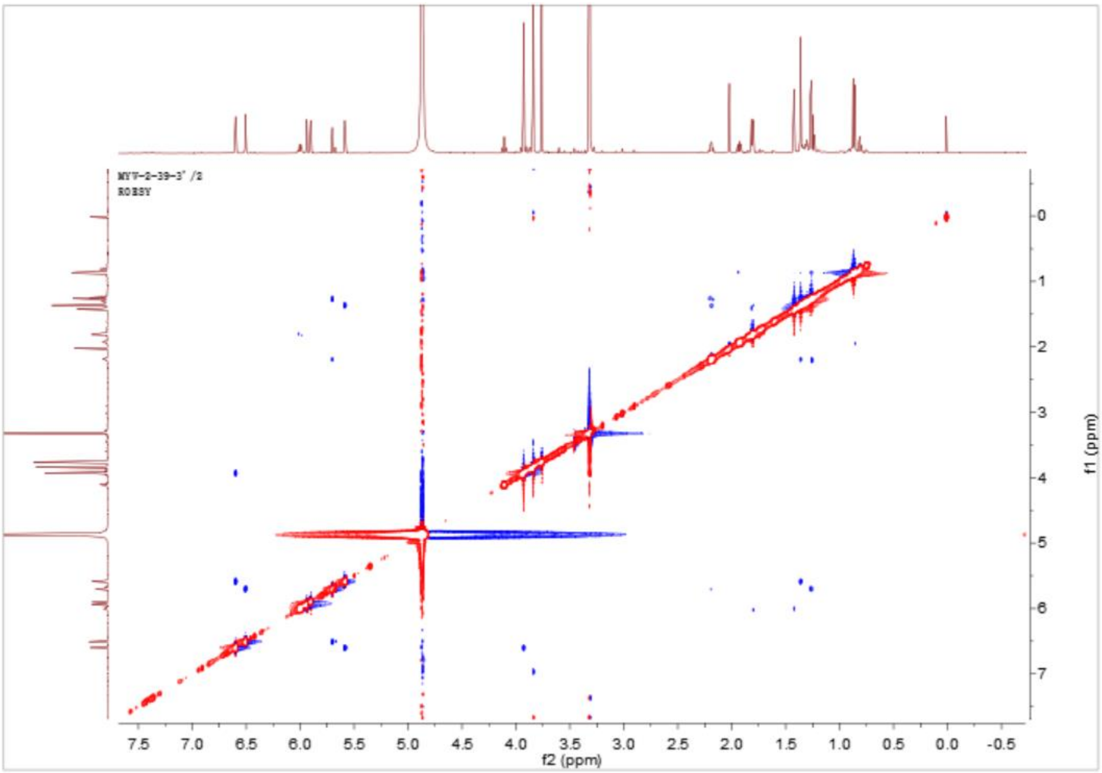

Figure S52. The ROESY spectrum of 5 in CD_3OD

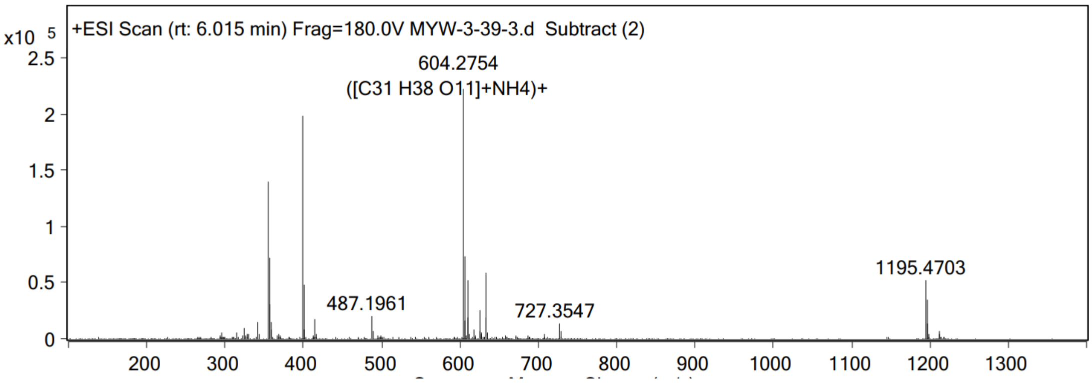

Formula Calculator Results

Formula	Best	Mass	Tgt Mass	Diff (ppm)	Ion Species	Score
C31 H38 O11	True	586.2415	586.2414	-0.15	C31 H42 N O11	99.32

Figure S53. The HRESIMS spectrum of 5

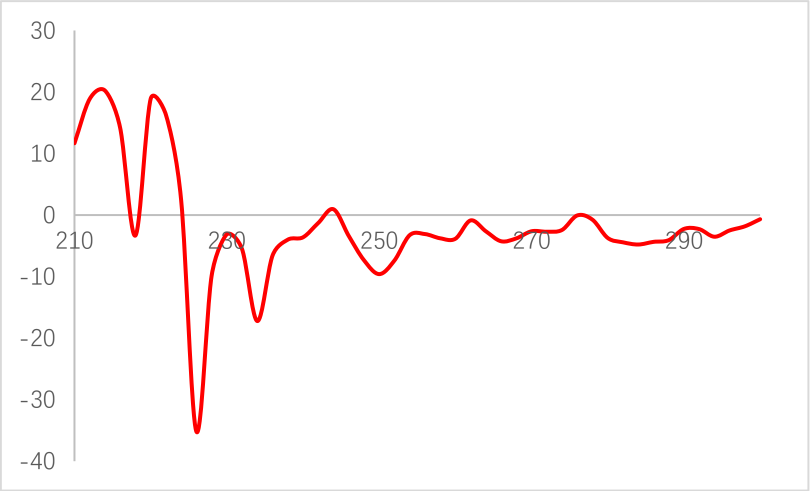

Figure S54. The ECD spectrum of 5 in MeOH

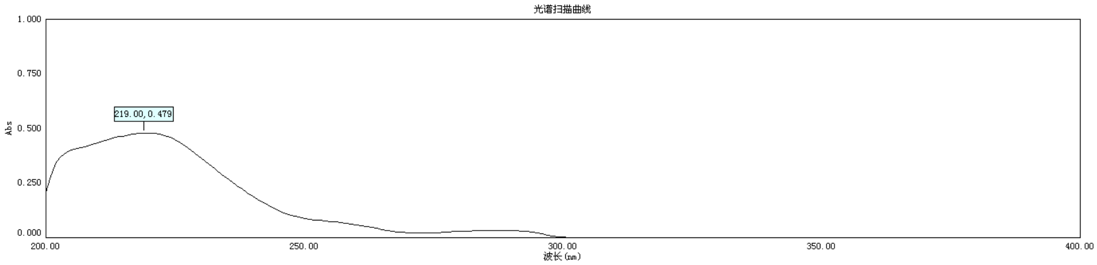

Figure S55. The UV spectrum of 5 in MeOH

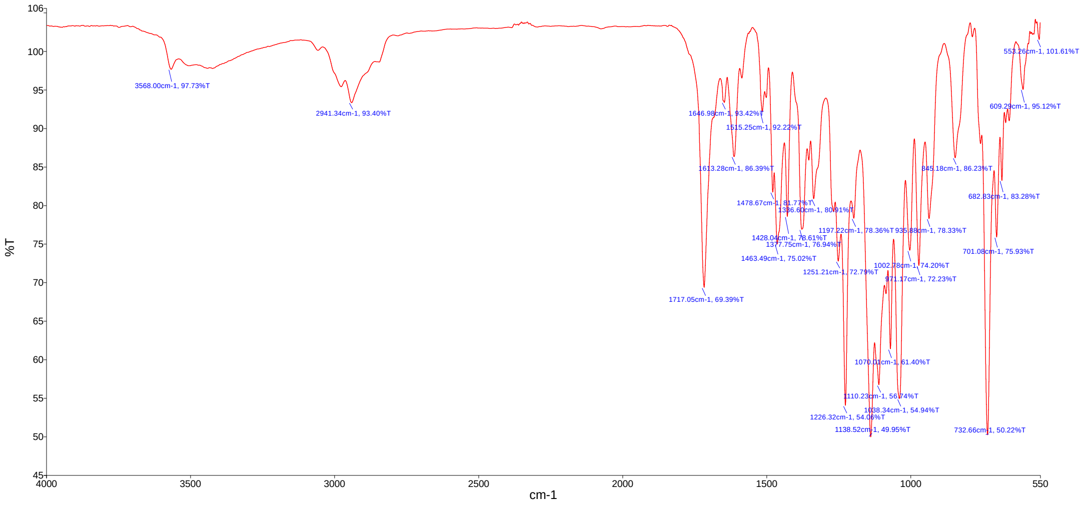

Figure S56. The IR spectrum of 5

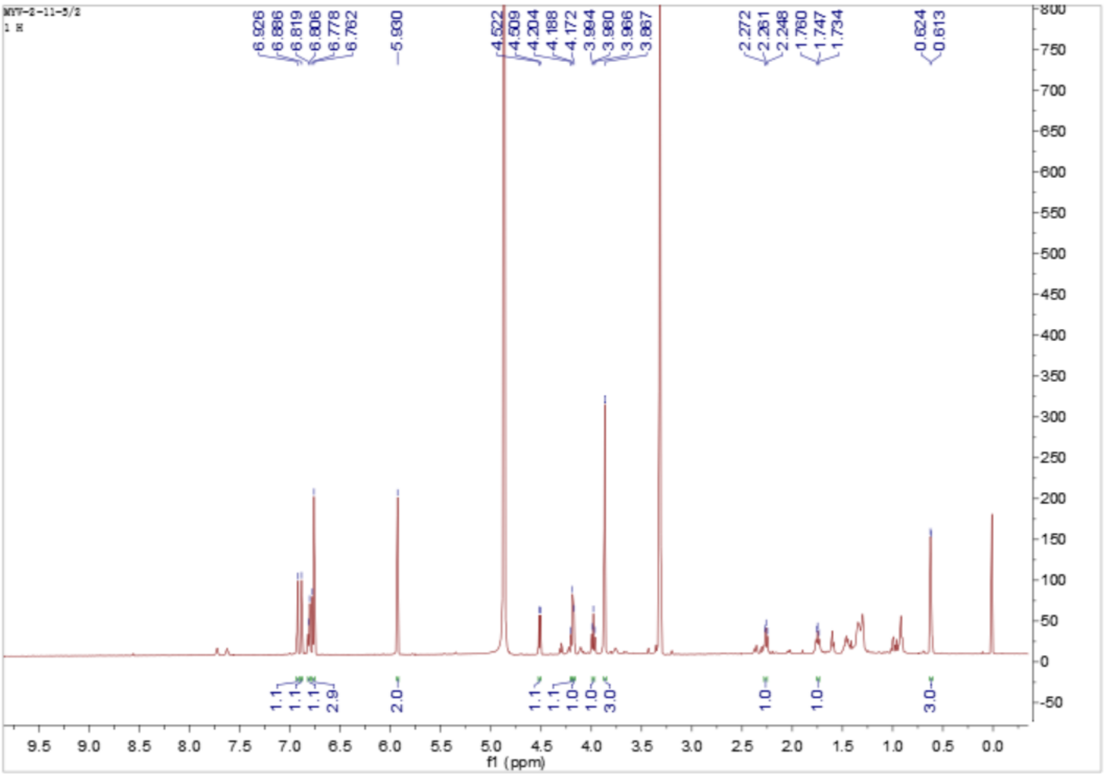

Figure S57. The ^{1}H NMR spectrum of 6 in CD_3OD

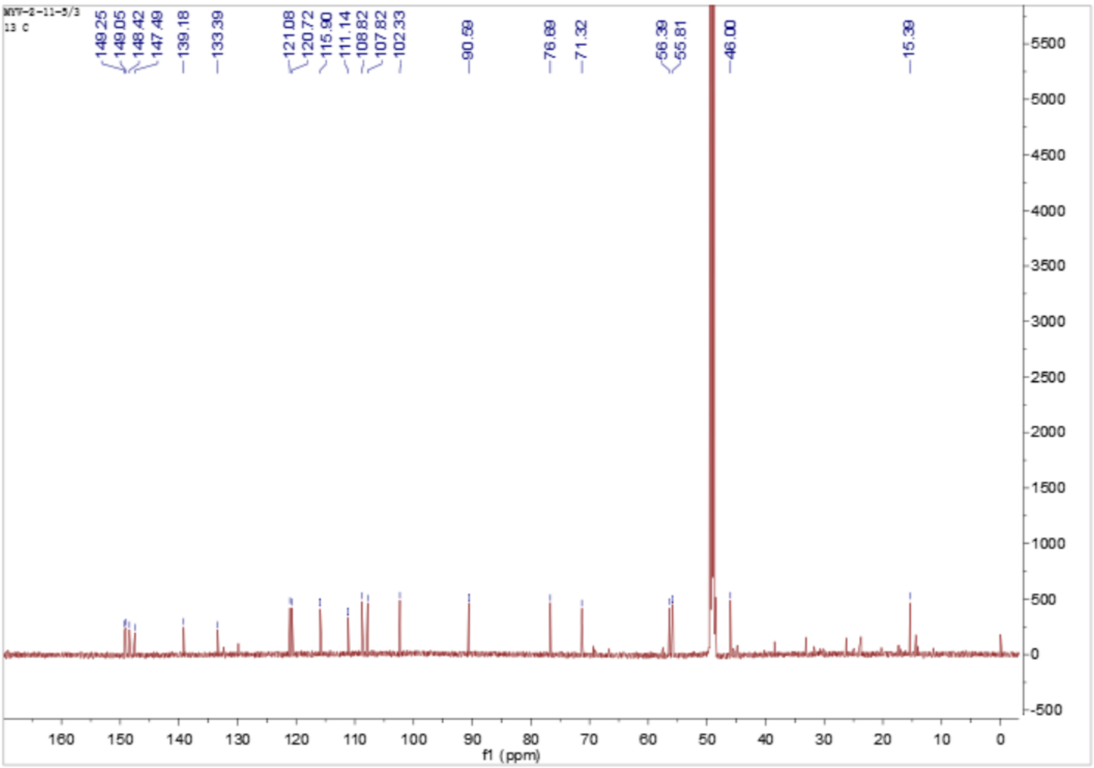

Figure S58. The ^{13}C NMR spectrum of 6 in CD_3OD

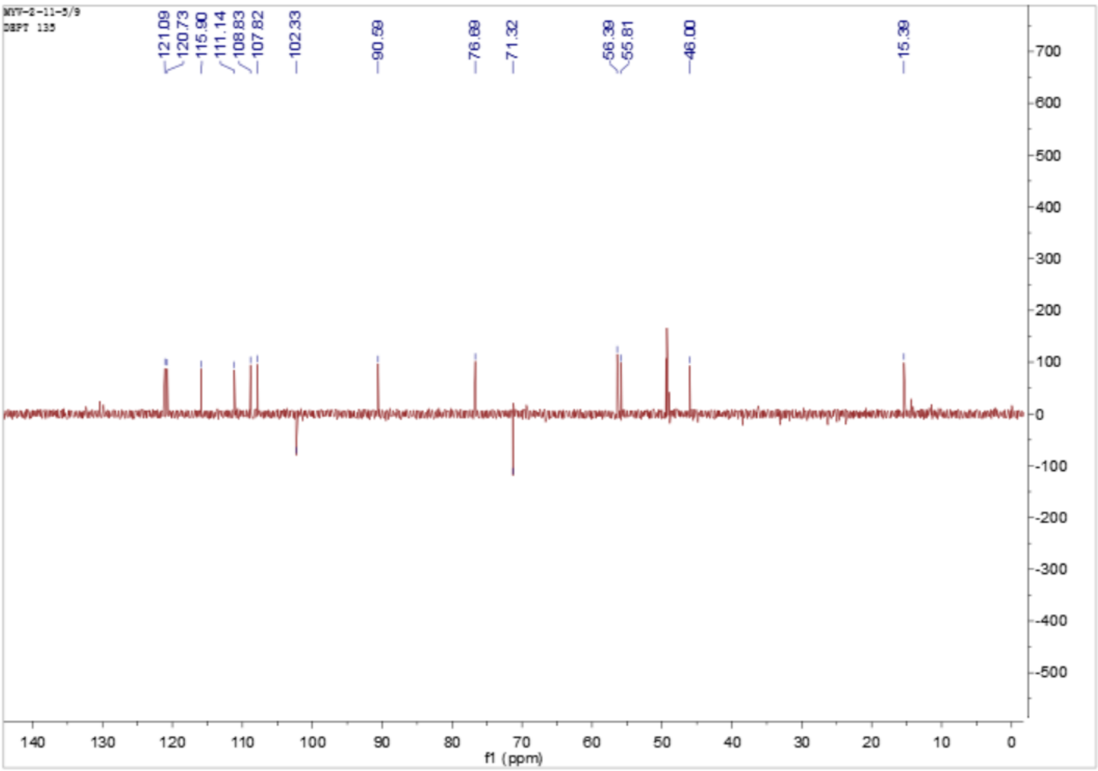

Figure S59. The DEPT spectrum of 6 in CD_3OD

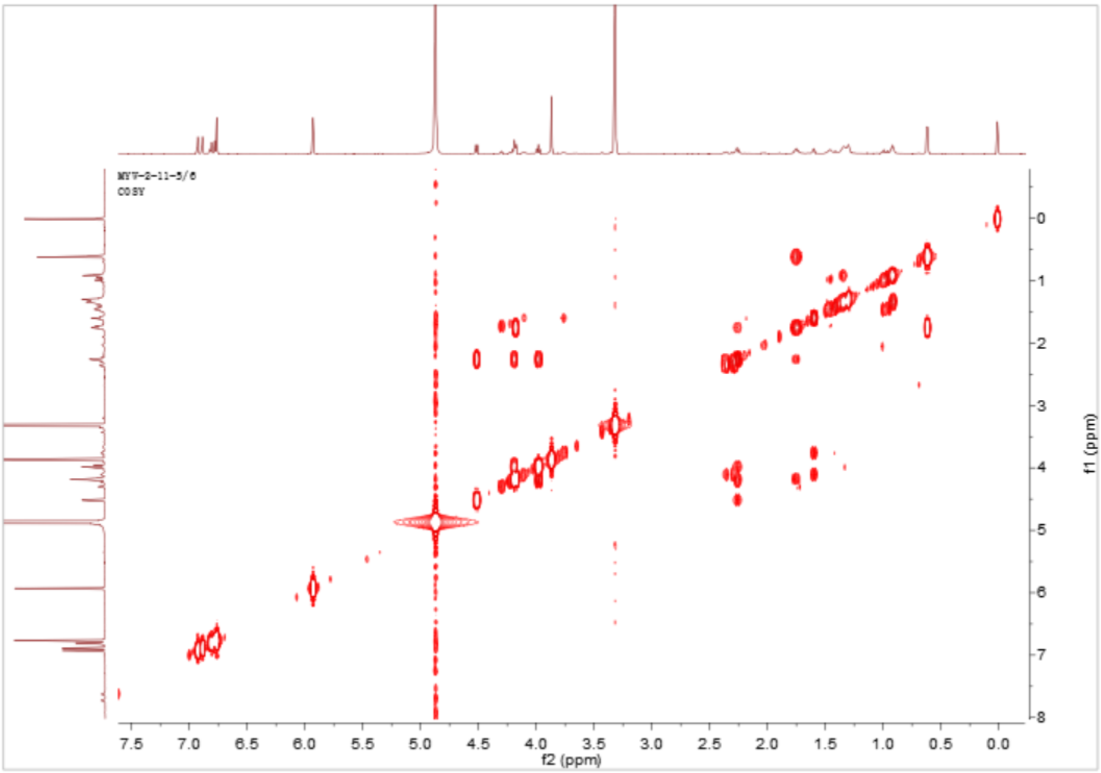

Figure S60. The ^1H-^1H COSY spectrum of 6 in CD_3OD

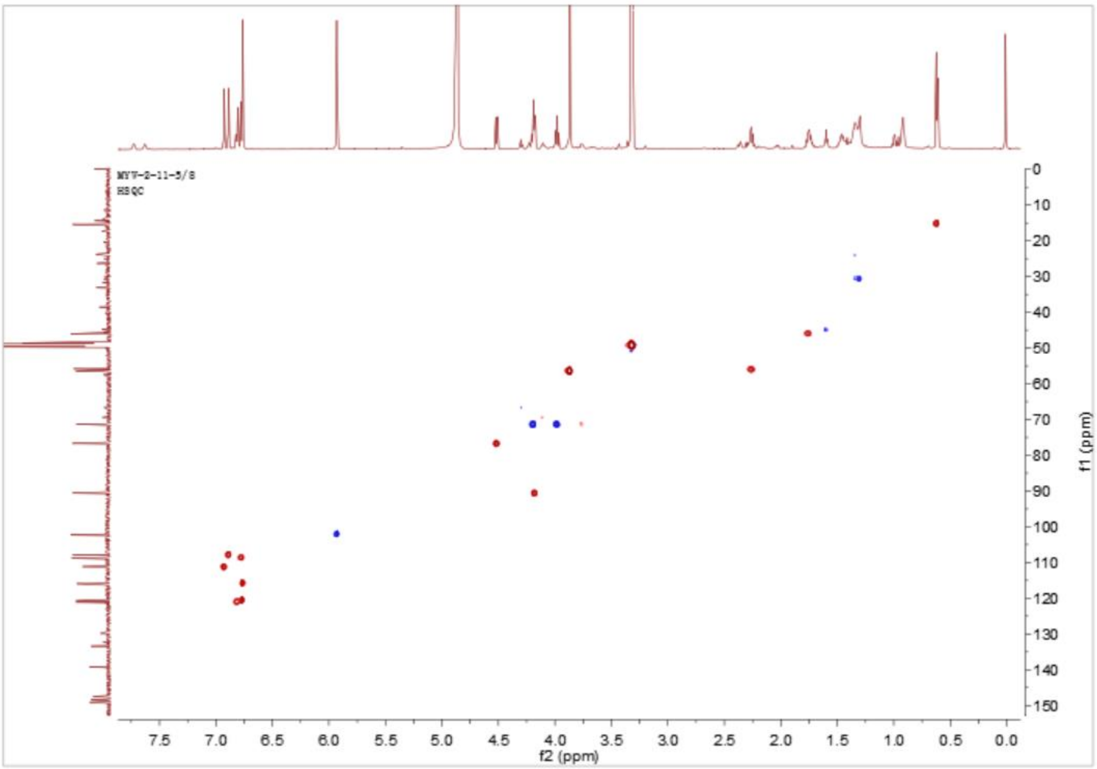

Figure S61. The HSQC spectrum of 6 in CD_3OD

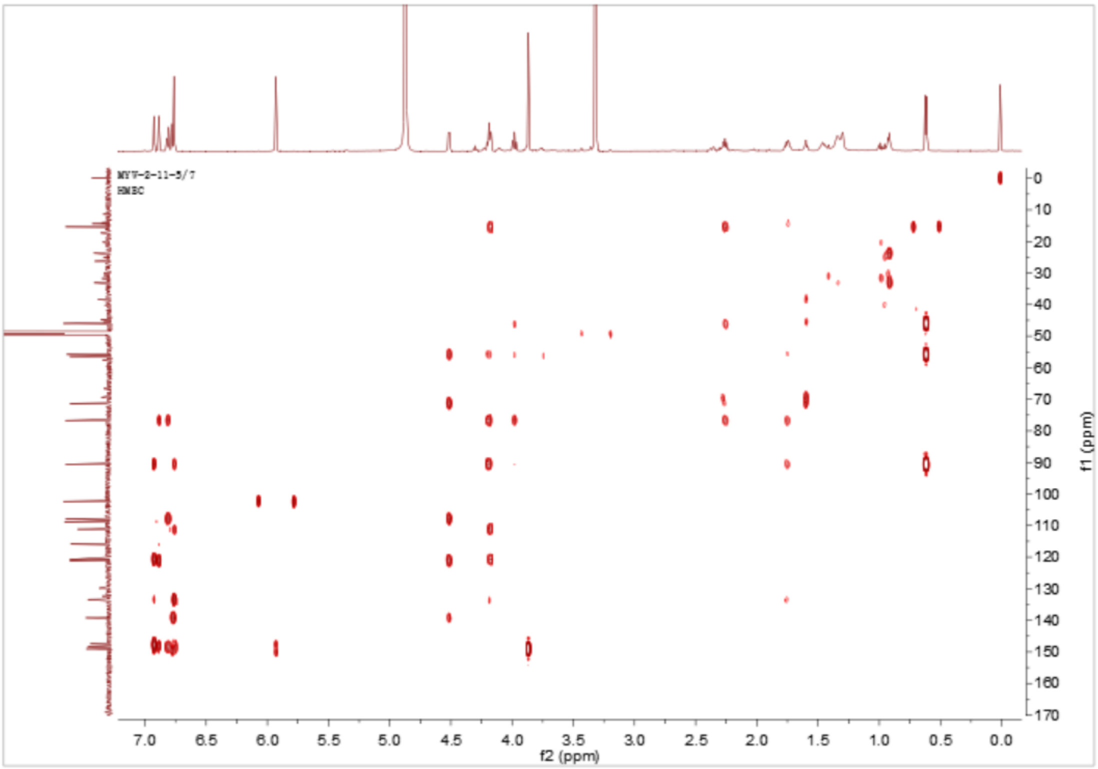

Figure S62. The HMBC spectrum of 6 in CD_3OD

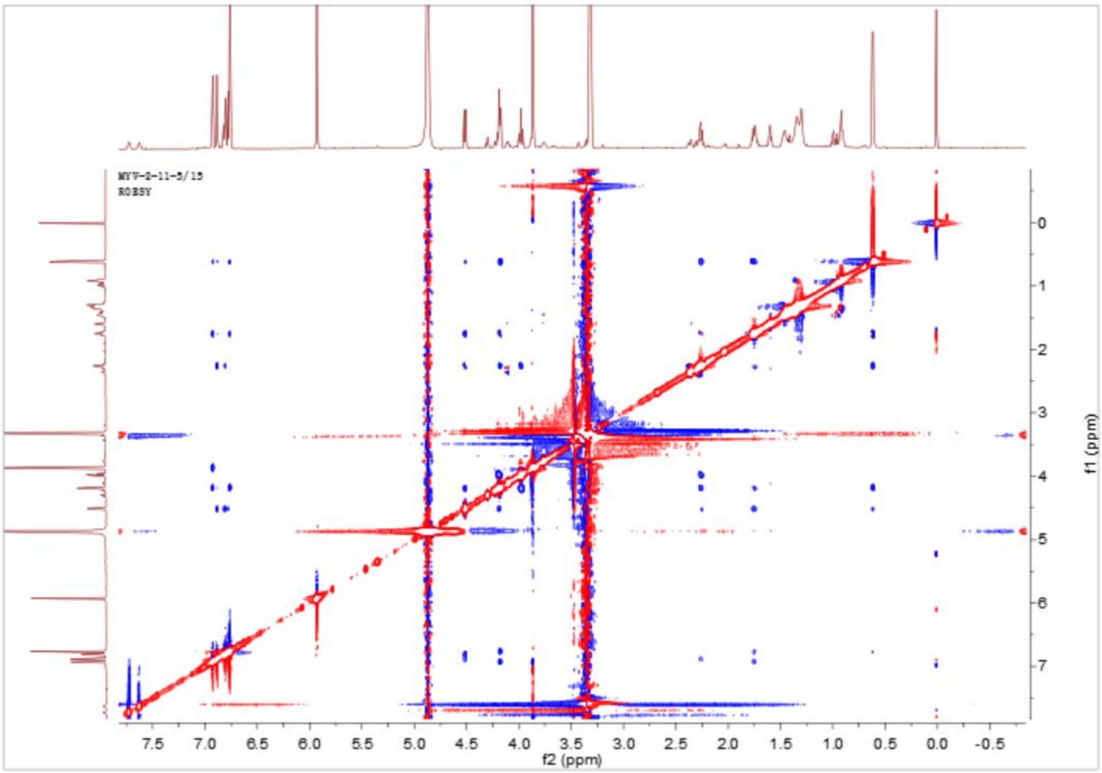

Figure S63. The HMBC spectrum of 6 in CD_3OD

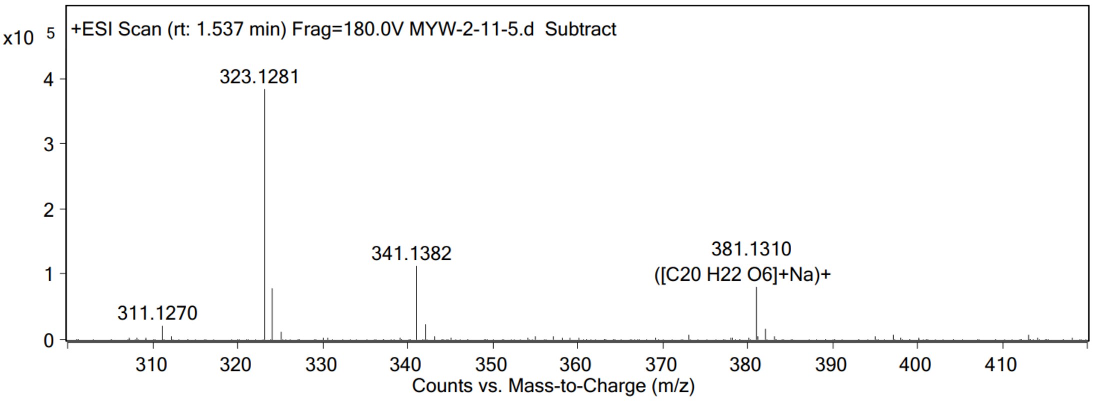

Formula Calculator Results

Formula	Best	Mass	Tgt Mass	Diff (ppm)	Ion Species	Score
C20 H22 O6	True	358.1417	358.1416	-0.23	C20 H22 Na O6	97.49

Figure S64. The HRESIMS spectrum of 6

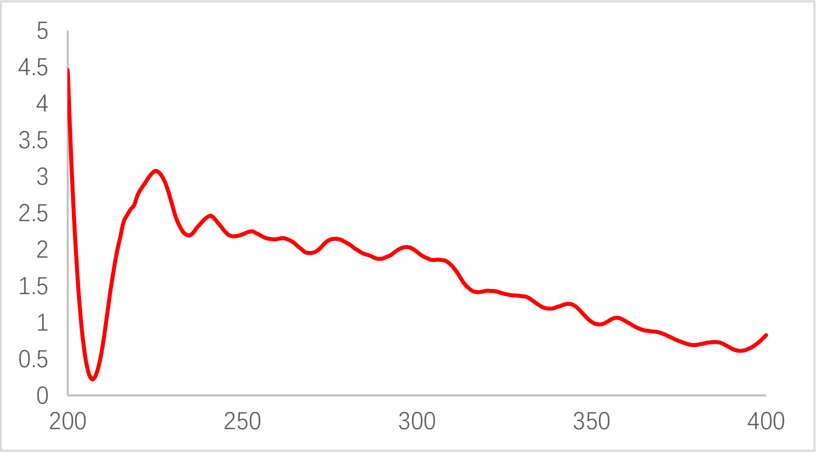

Figure S65. The ECD spectrum of 6 in MeOH

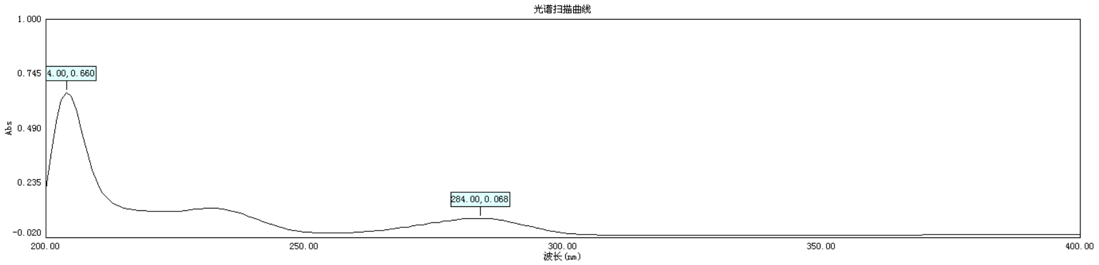

Figure S66. The UV spectrum of 6 in MeOH

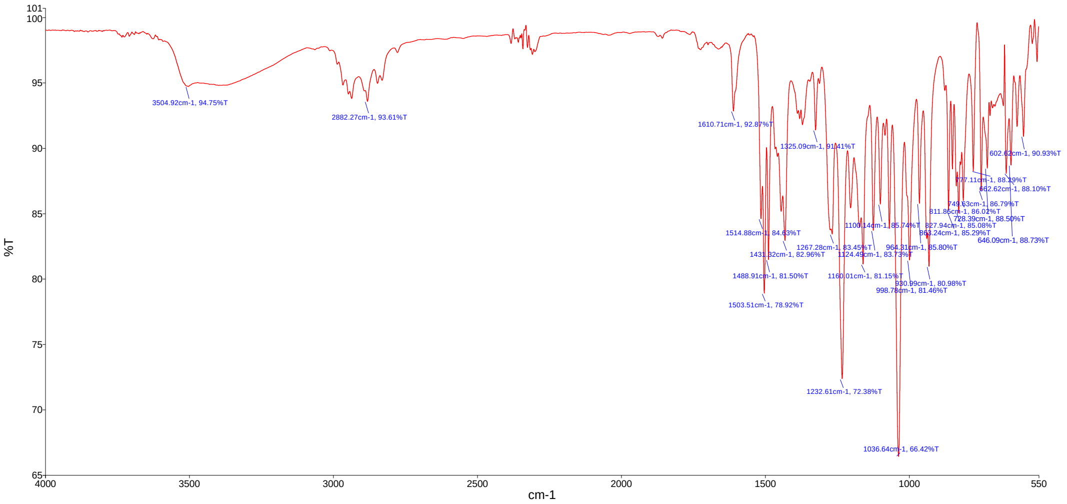

Figure S67. The IR spectrum of 6

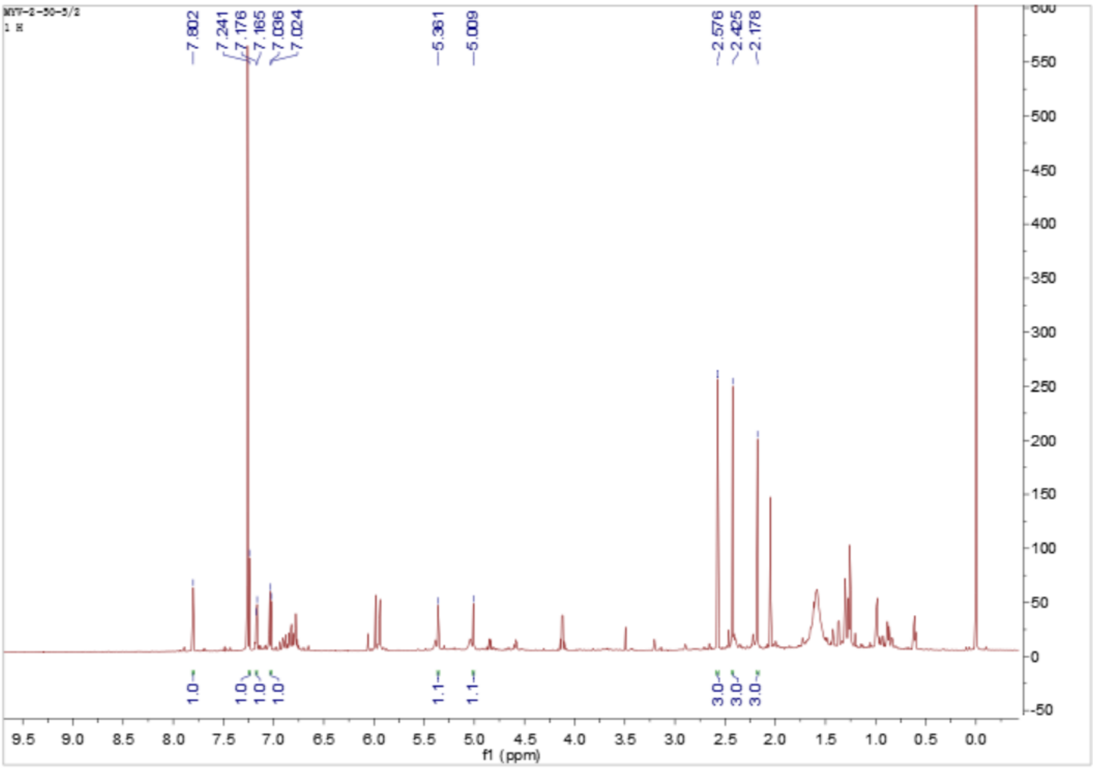

Figure S68. The ^{1}H NMR spectrum of 7 in $CDCl_3$

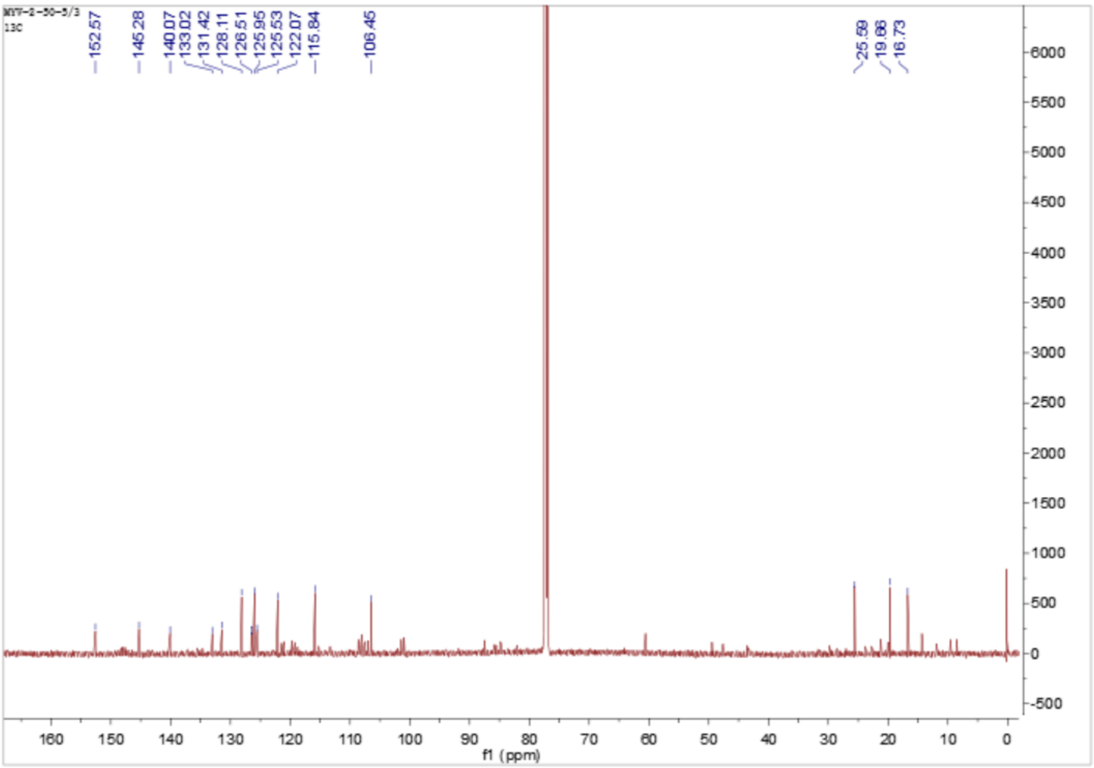

Figure S69. The ^{13}C NMR spectrum of 7 in $CDCl_3$

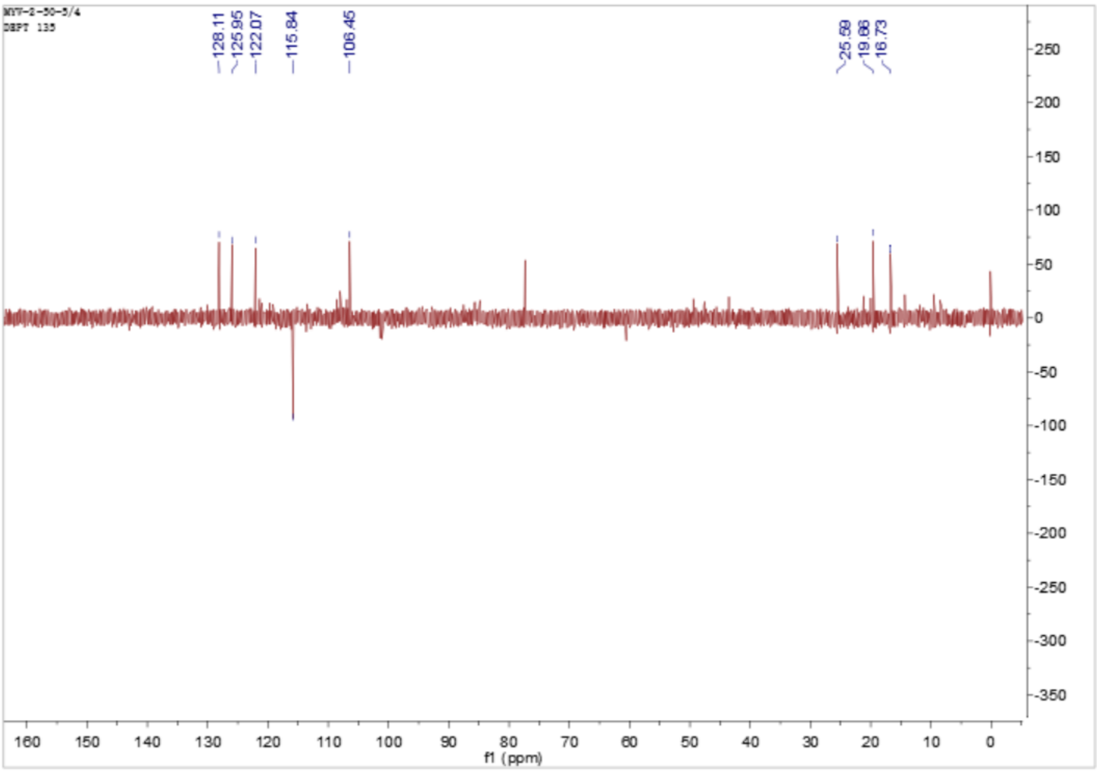

Figure S70. The DEPT spectrum of 7 in $CDCl_3$

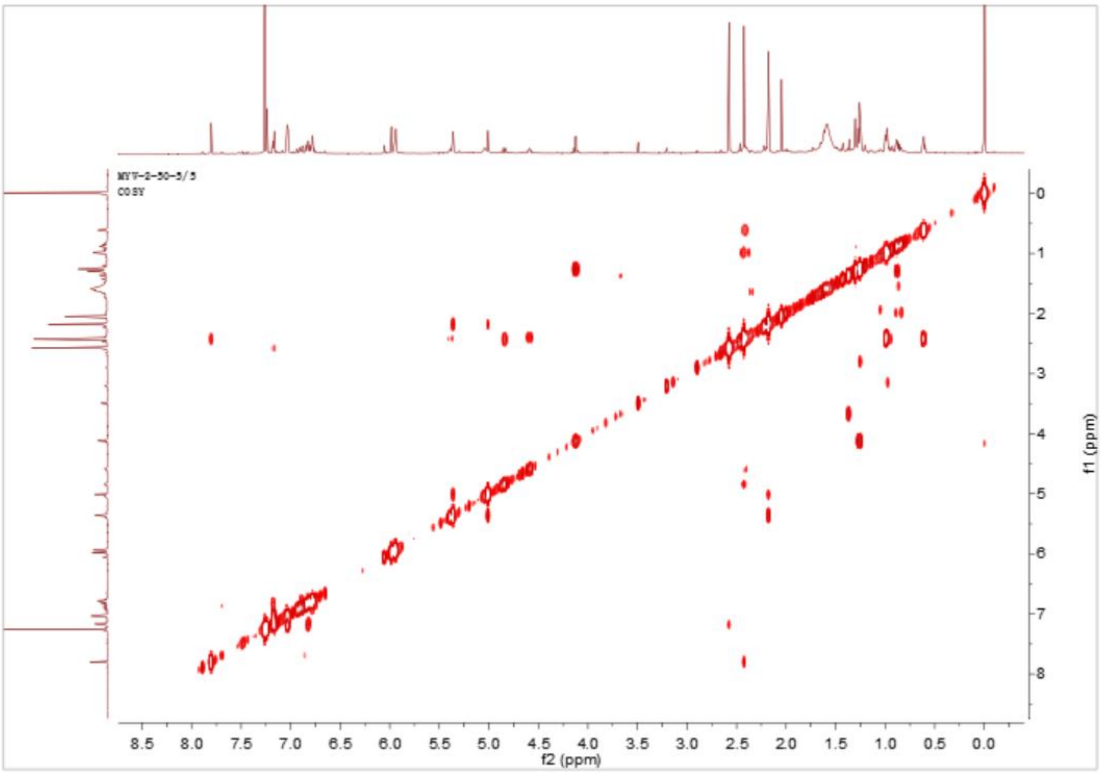

Figure S71. The ^{1}H-^{1}H COSY spectrum of 7 in $CDCl_3$

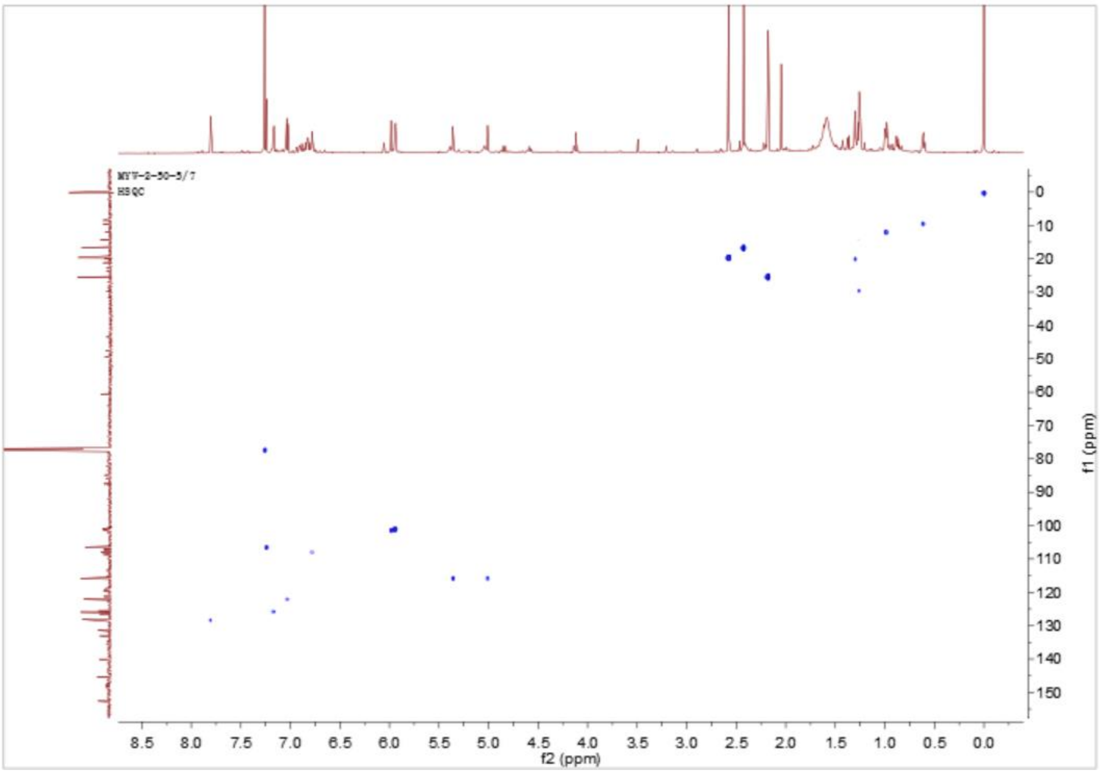

Figure S72. The HSQC spectrum of 7 in $CDCl_3$

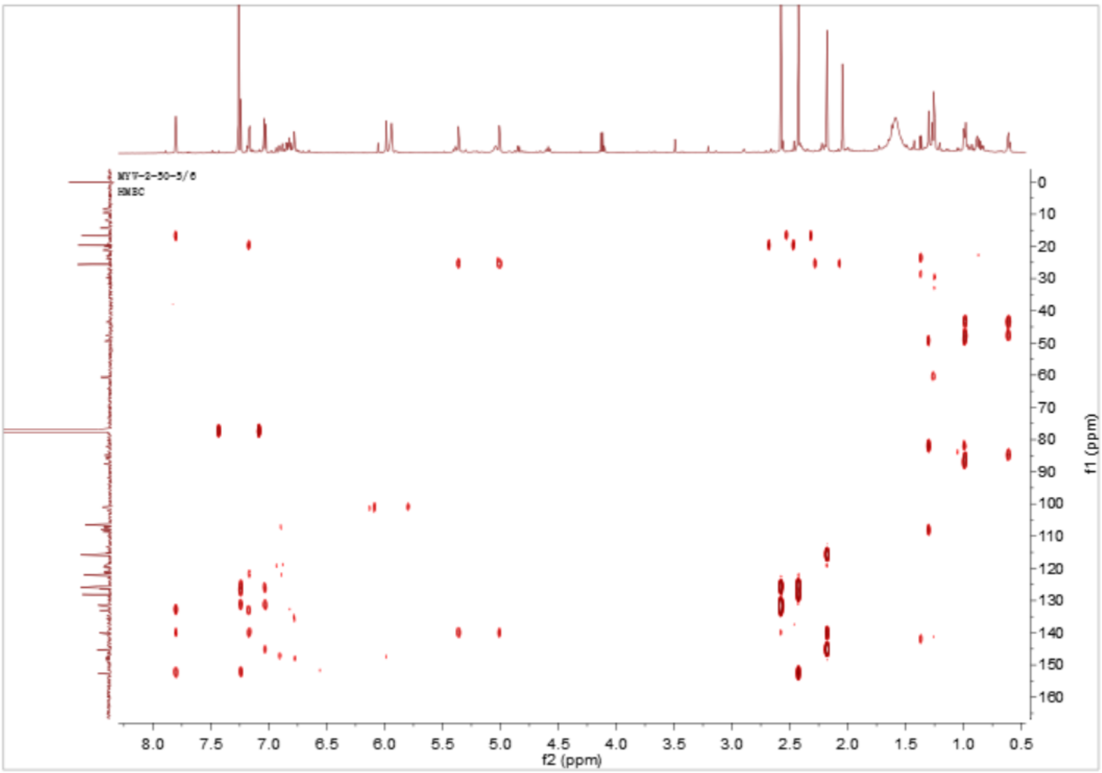

Figure S73. The HMBC spectrum of 7 in $CDCl_3$

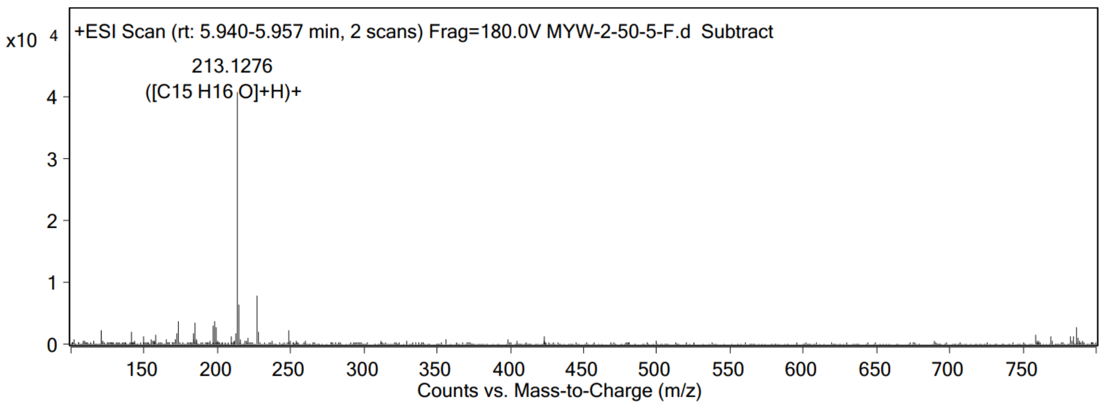

Formula Calculator Results

Formula	Best	Mass	Tgt Mass	Diff (ppm)	Ion Species	Score
C15 H16 O	True	212.1203	212.1201	-1.09	C15 H17 O	99.14

Figure S74. The HRESIMS spectrum of 7

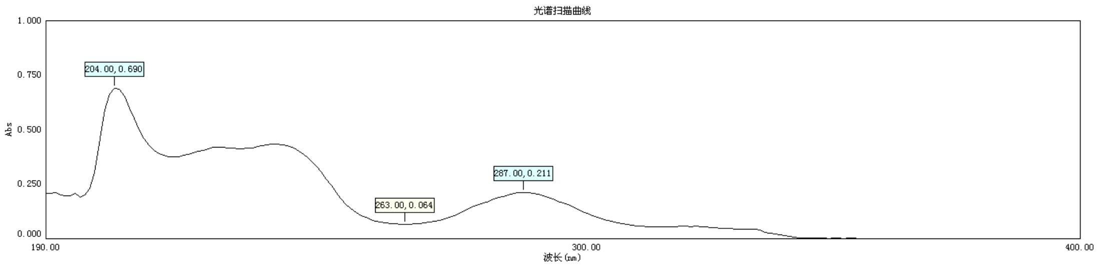

Figure S75. The UV spectrum of 7 in CH_2Cl_2

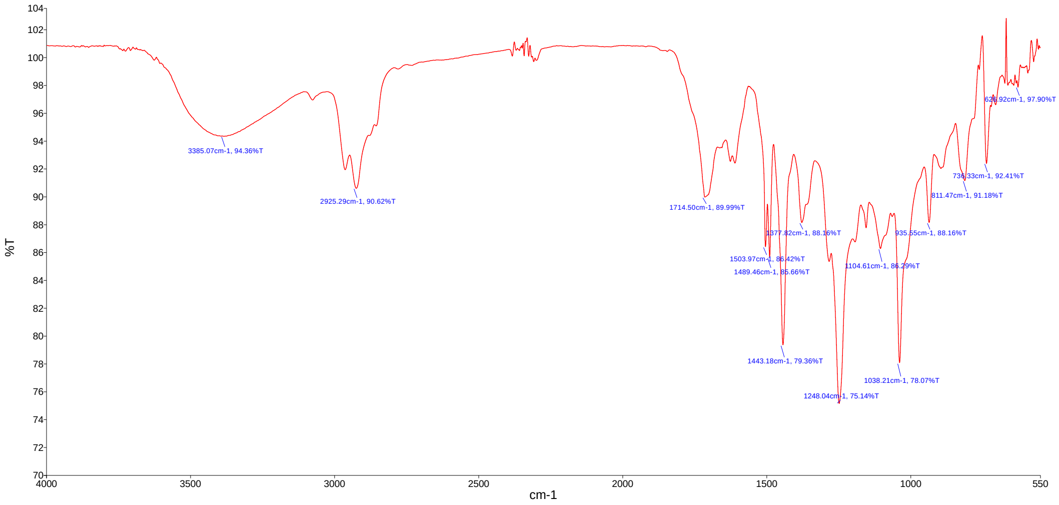

Figure S76. The IR spectrum of 7

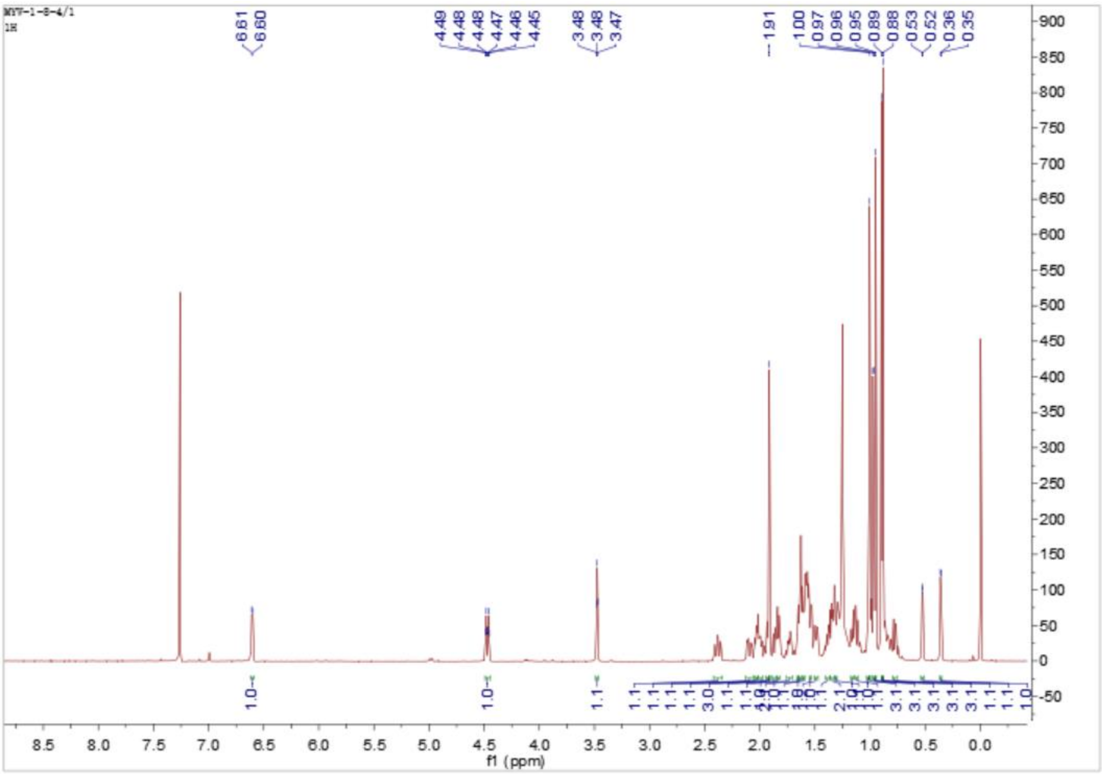

Figure S77. The ^{1}H NMR spectrum of 8 in $CDCl_3$

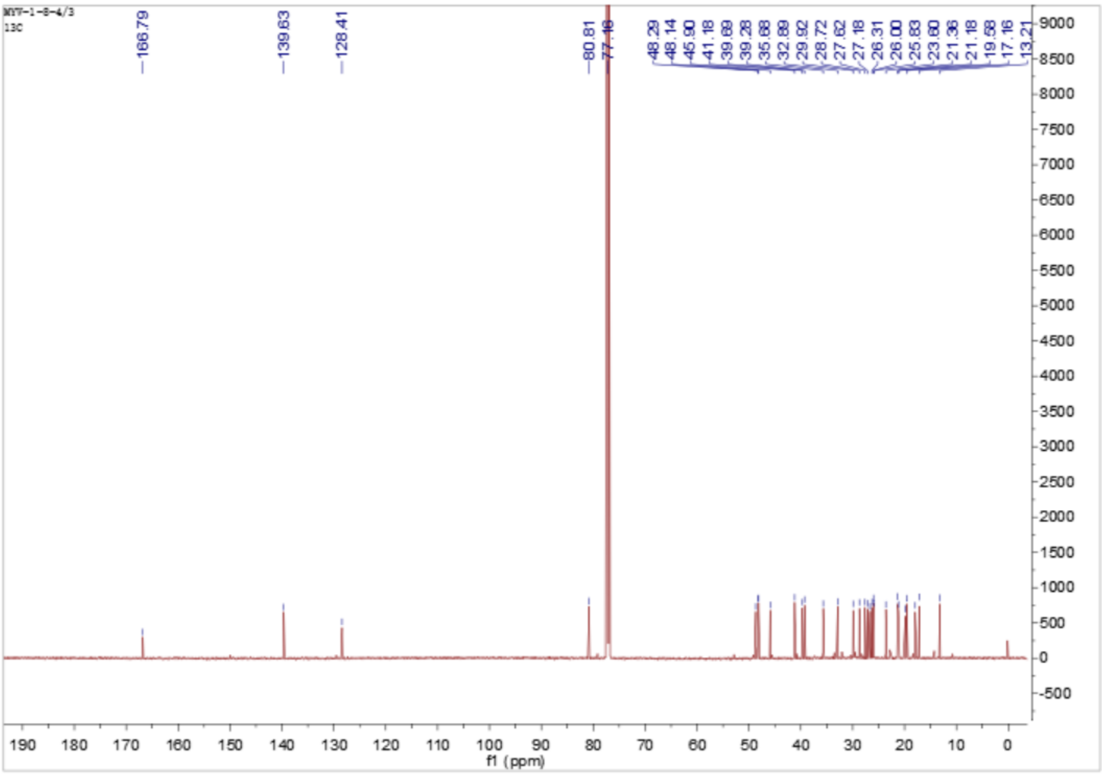

Figure S78. The ^{13}C NMR spectrum of 8 in $CDCl_3$

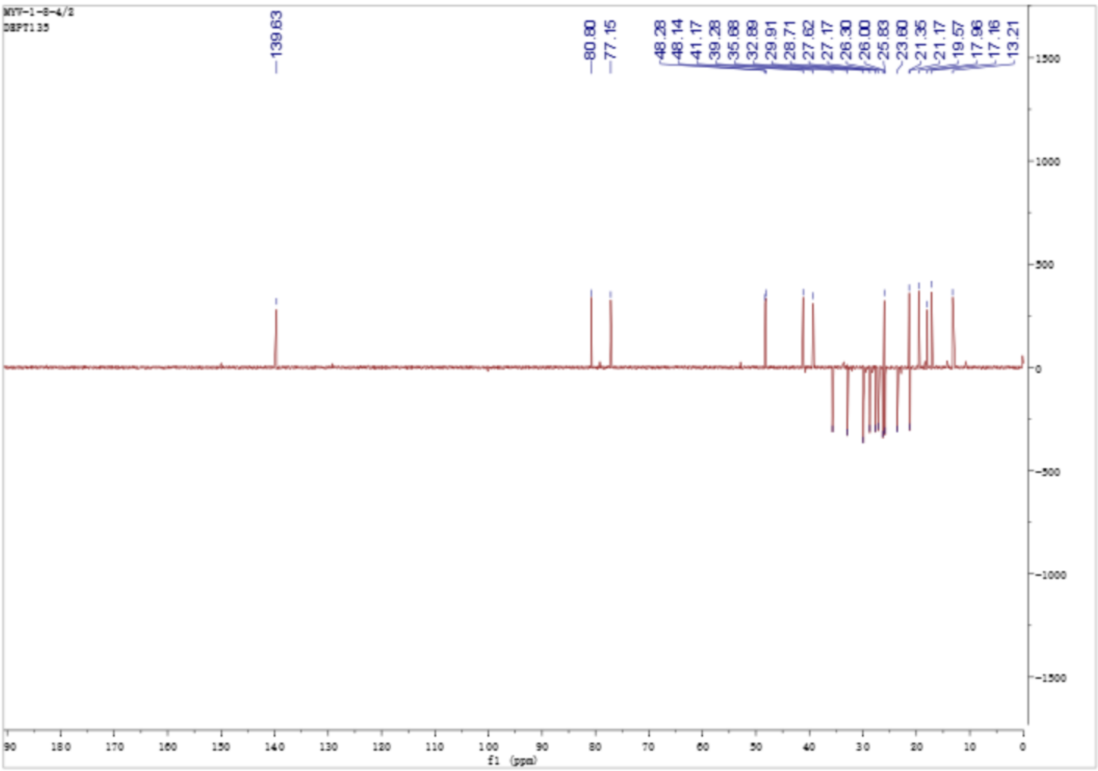

Figure S79. The DEPT spectrum of 8 in $CDCl_3$

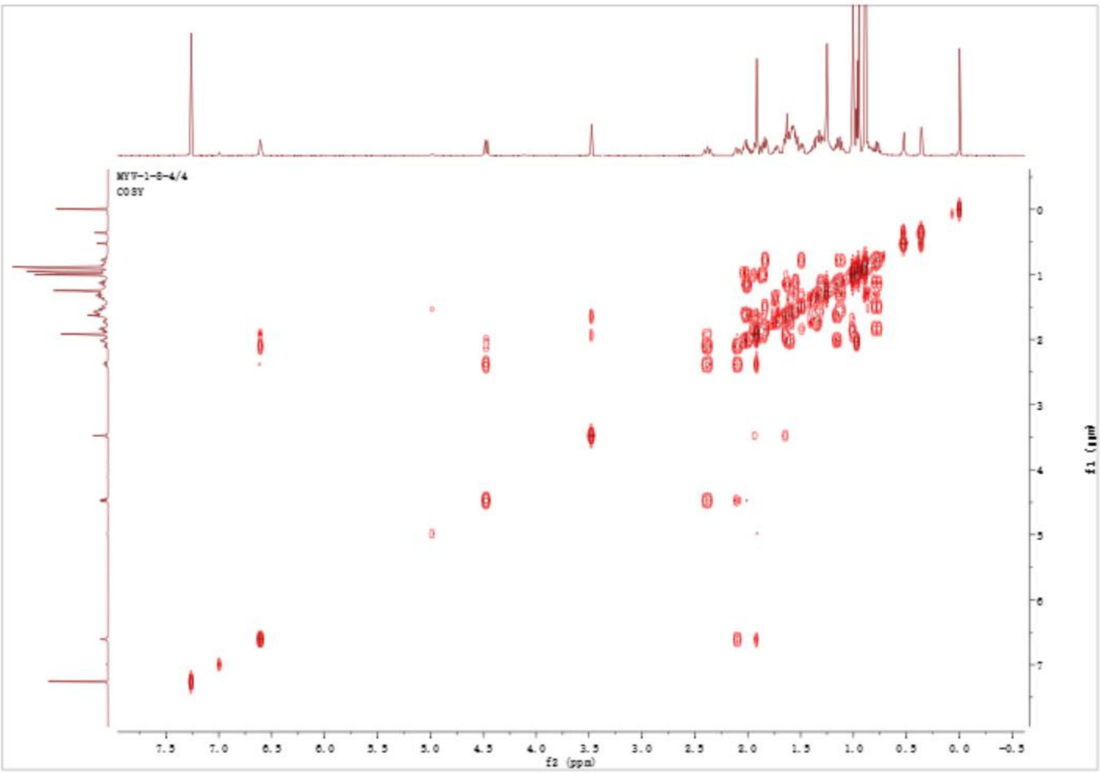

Figure S80. The ^1H-^1H COSY spectrum of 8 in $CDCl_3$

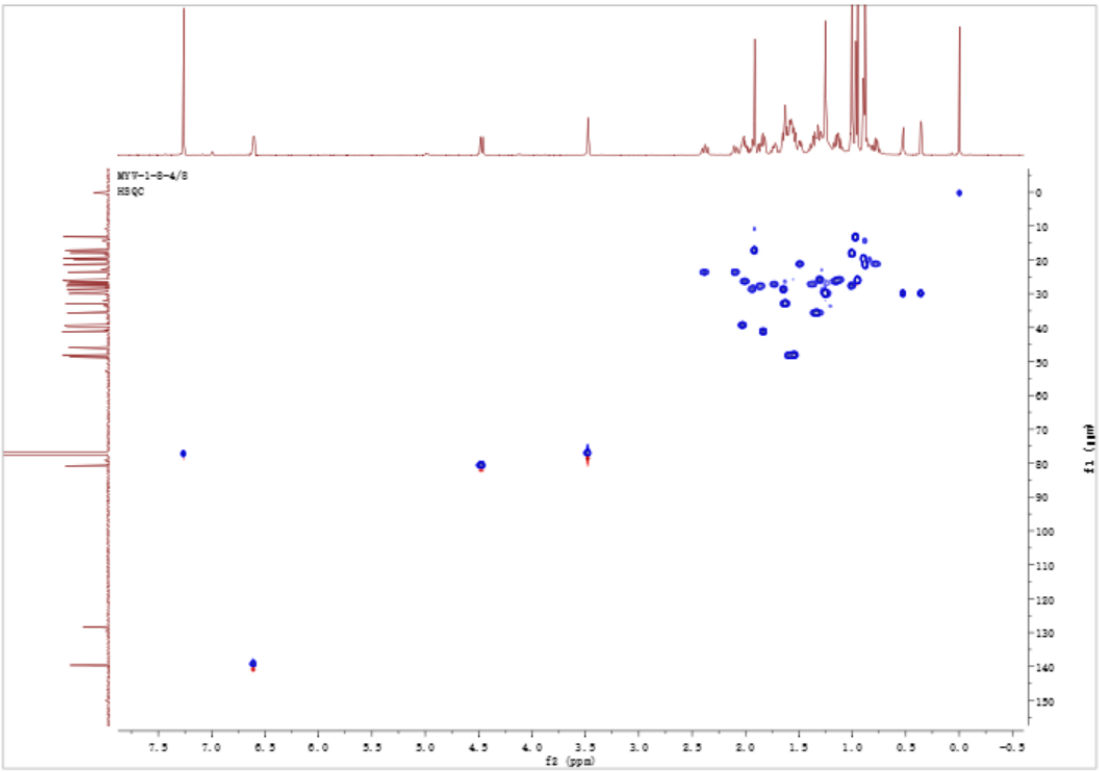

Figure S81. The HSQC spectrum of 8 in $CDCl_3$

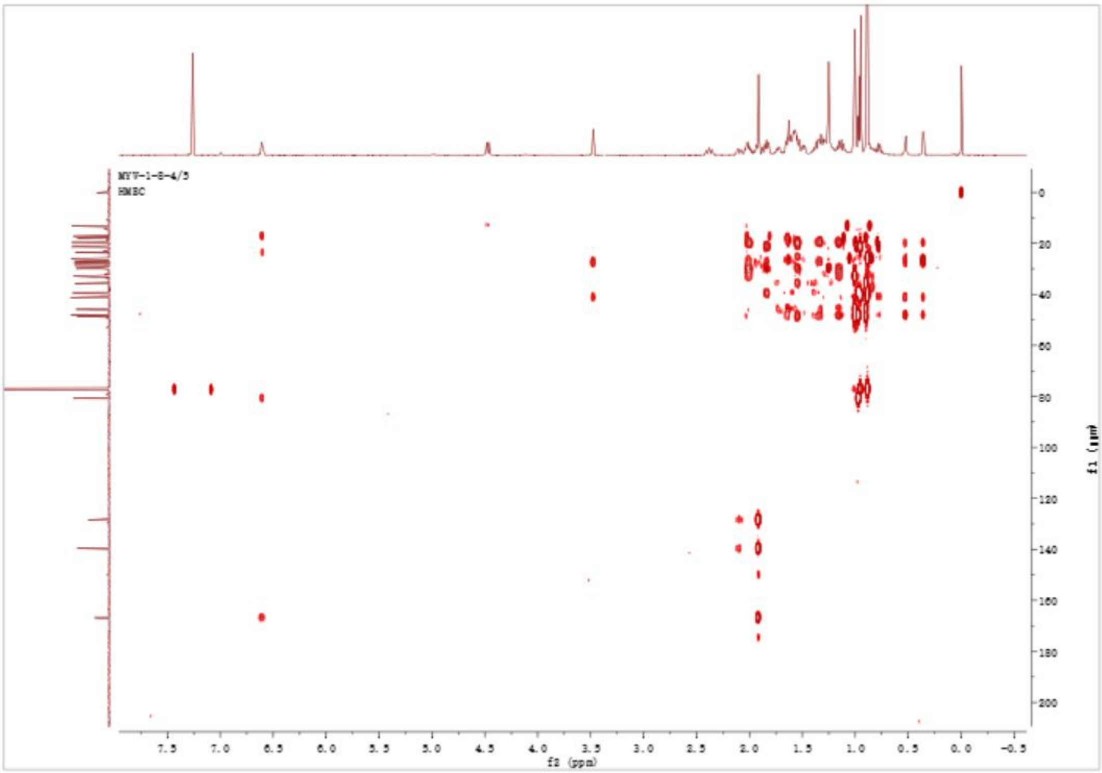

Figure S82. The HMBC spectrum of 8 in $CDCl_3$

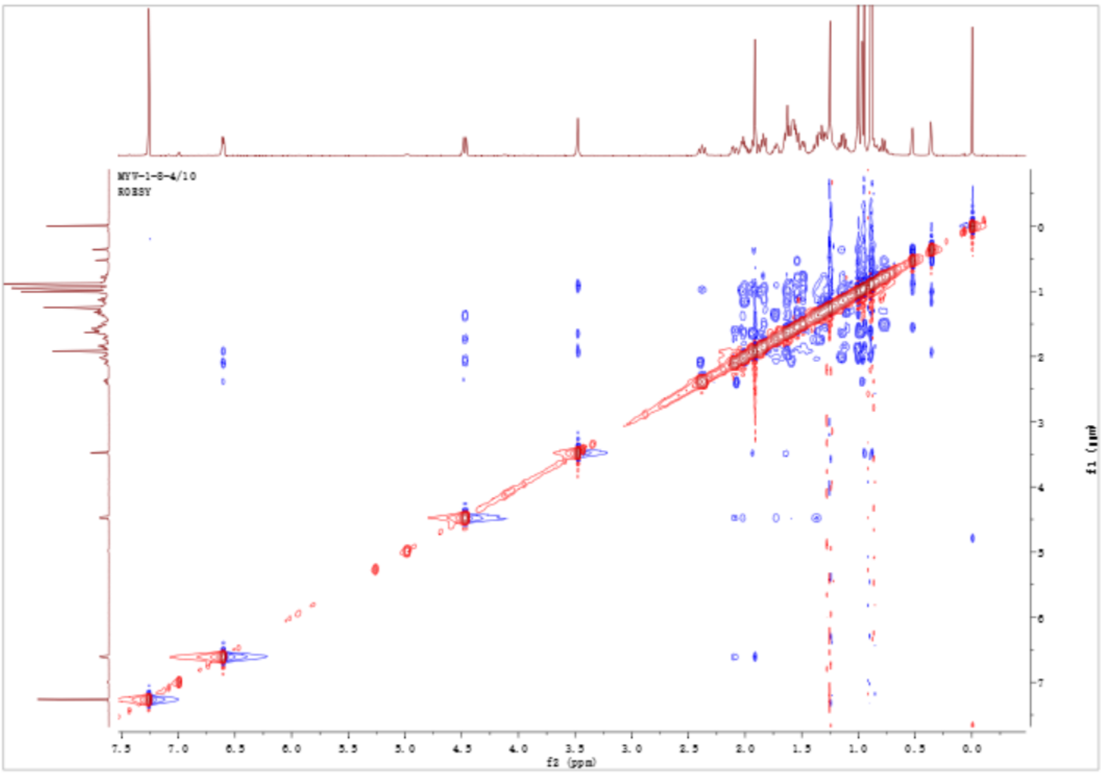

Figure S83. The ROESY spectrum of 8 in $CDCl_3$

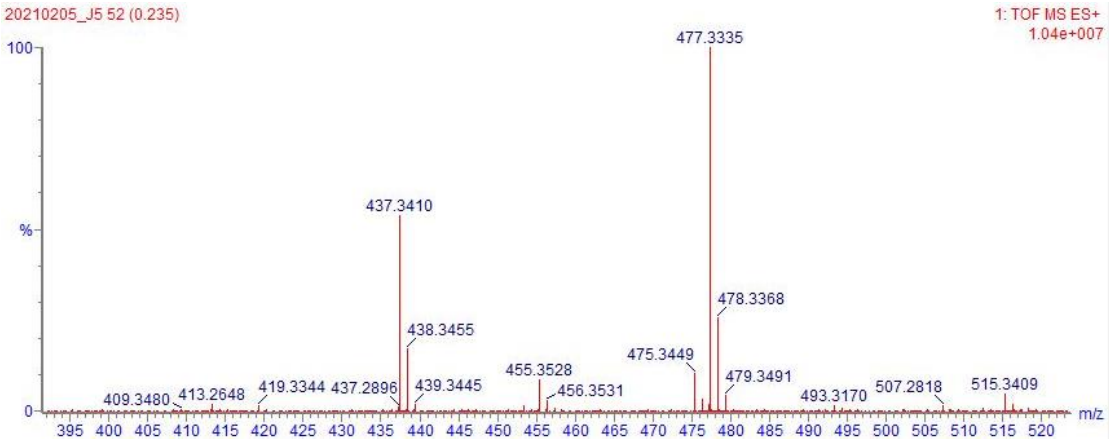

Mass	Calc. Mass	mDa	PPM	DBE	Formula	i-FIT	i-FIT Norm	Fit Conf %	C	H	N	O	Na
477.3335	477.3345	-1.0	-2.1	7.5	C30 H46 O3 Na	550.7	2.013	13.36	30	46		3	1

Figure S84. The HRESIMS spectrum of 8

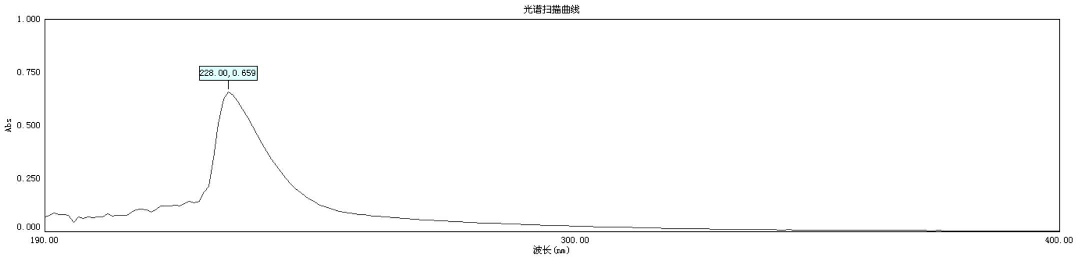

Figure S85. The UV spectrum of 8 in CH_2Cl_2

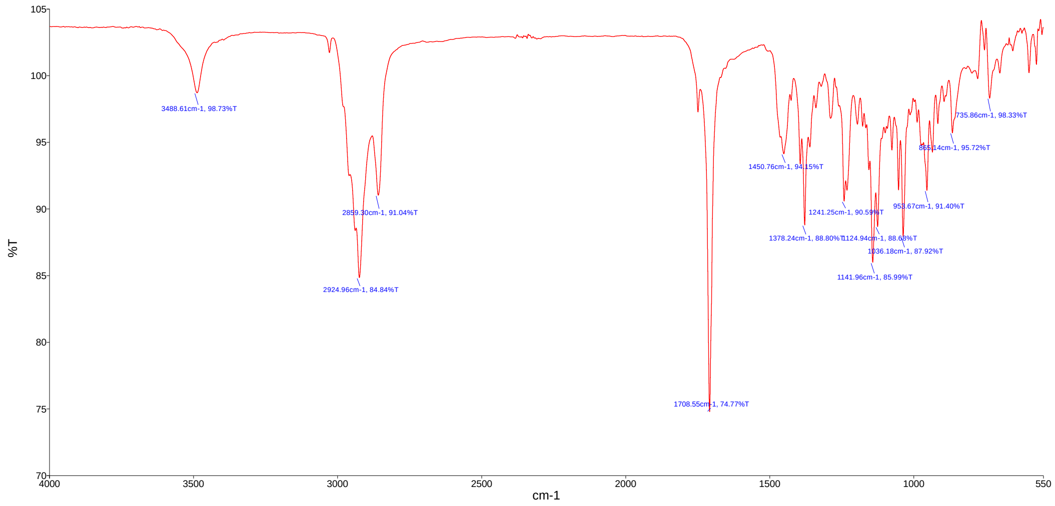

Figure S86. The IR spectrum of 8

4. 1D NMR spectra of known compounds 9-51.

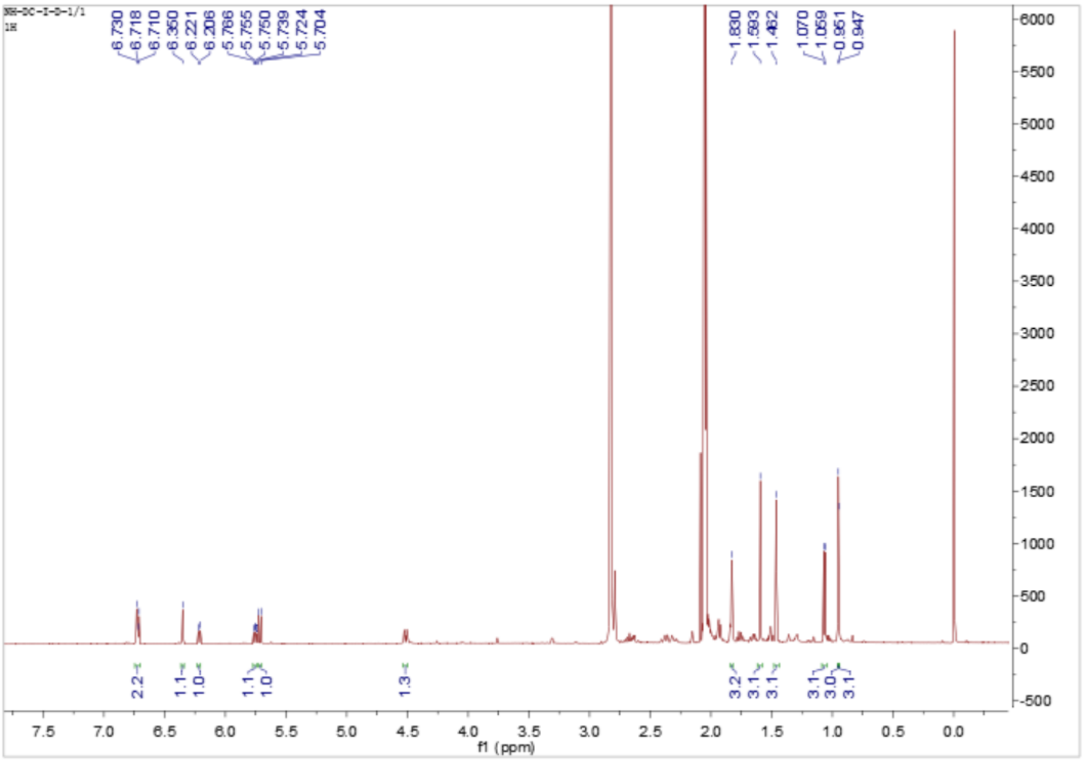

Figure S87. The ^{1}H NMR spectrum of 9 in Acetone-d_6

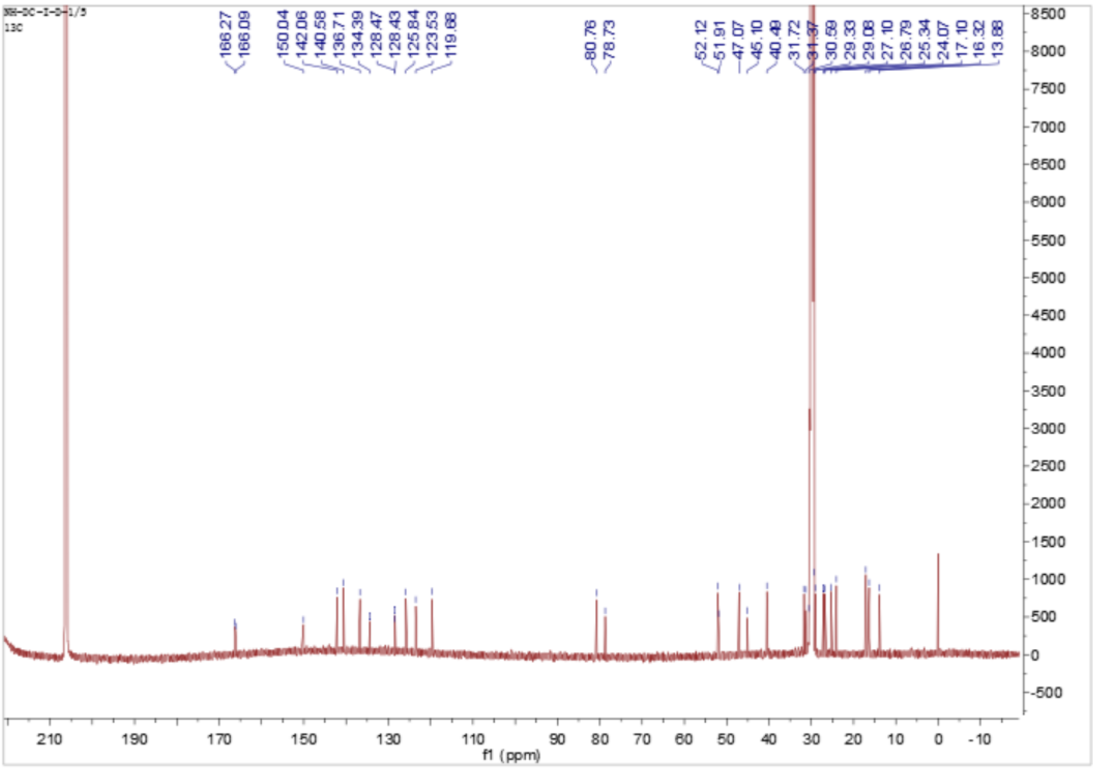

Figure S88. The ^{13}C NMR spectrum of 9 in Acetone-d_6

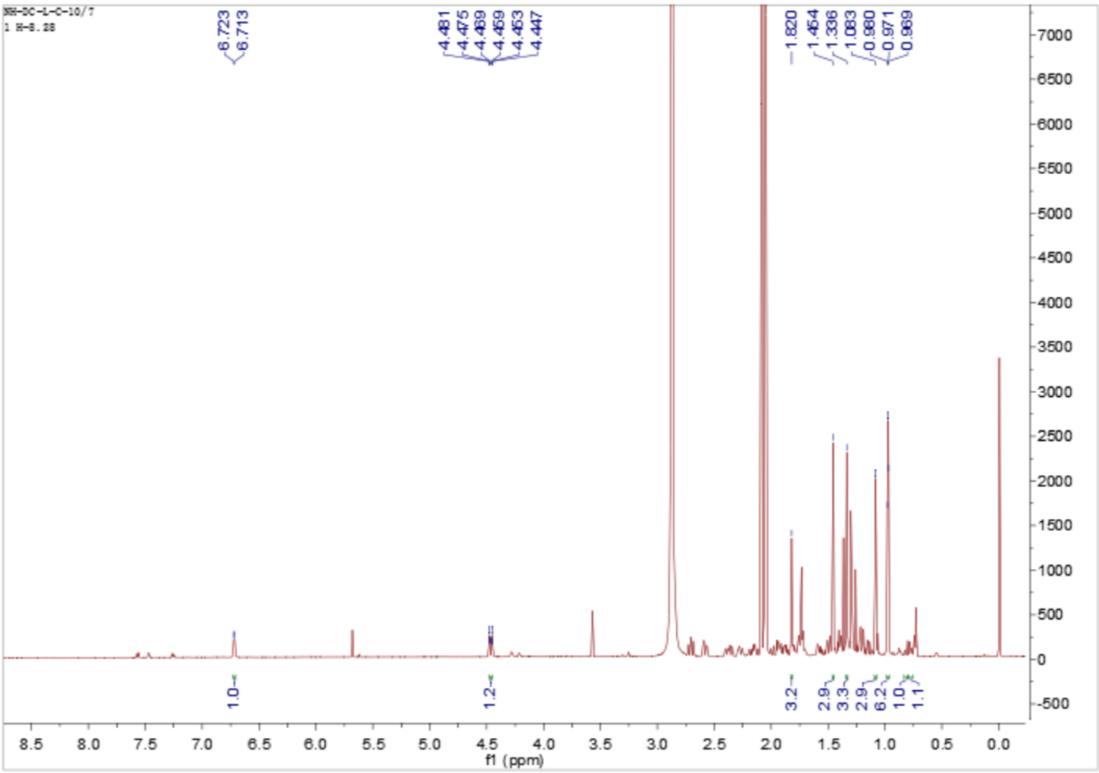

Figure S89. The ^{1}H NMR spectrum of 10 in Acetone-d_6

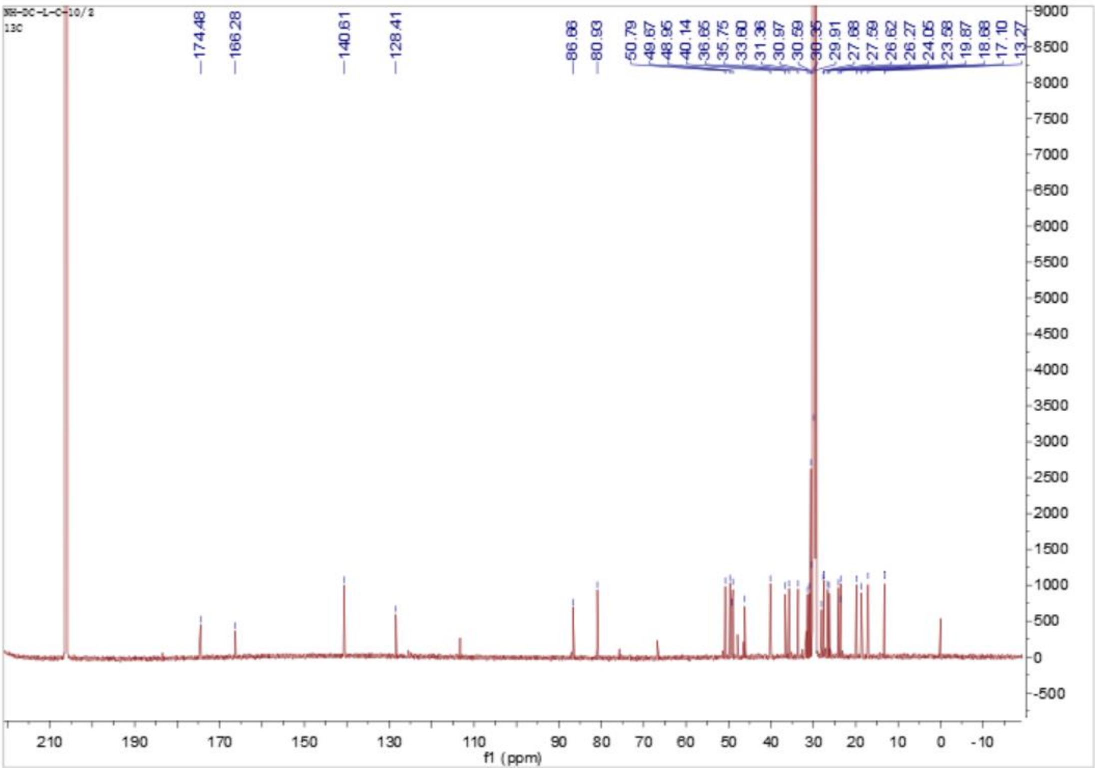

Figure S90. The ^{13}C NMR spectrum of 10 in Acetone-d_6

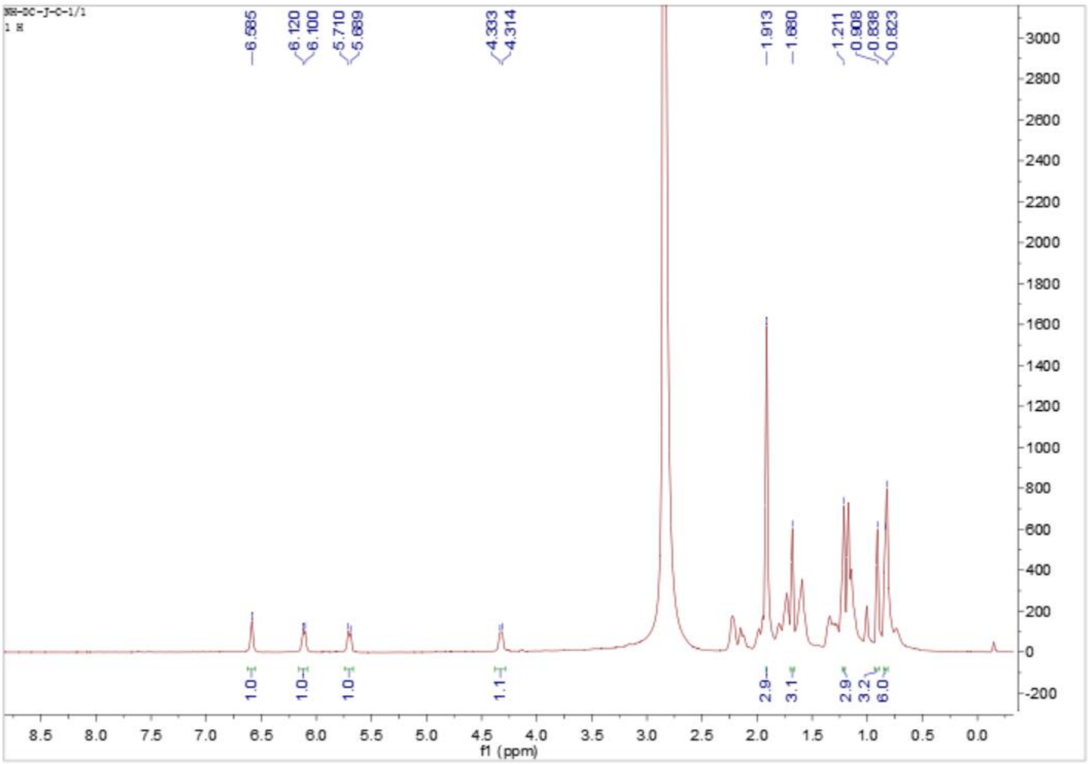

Figure S91. The ^{1}H NMR spectrum of 11 in Acetone-d_6

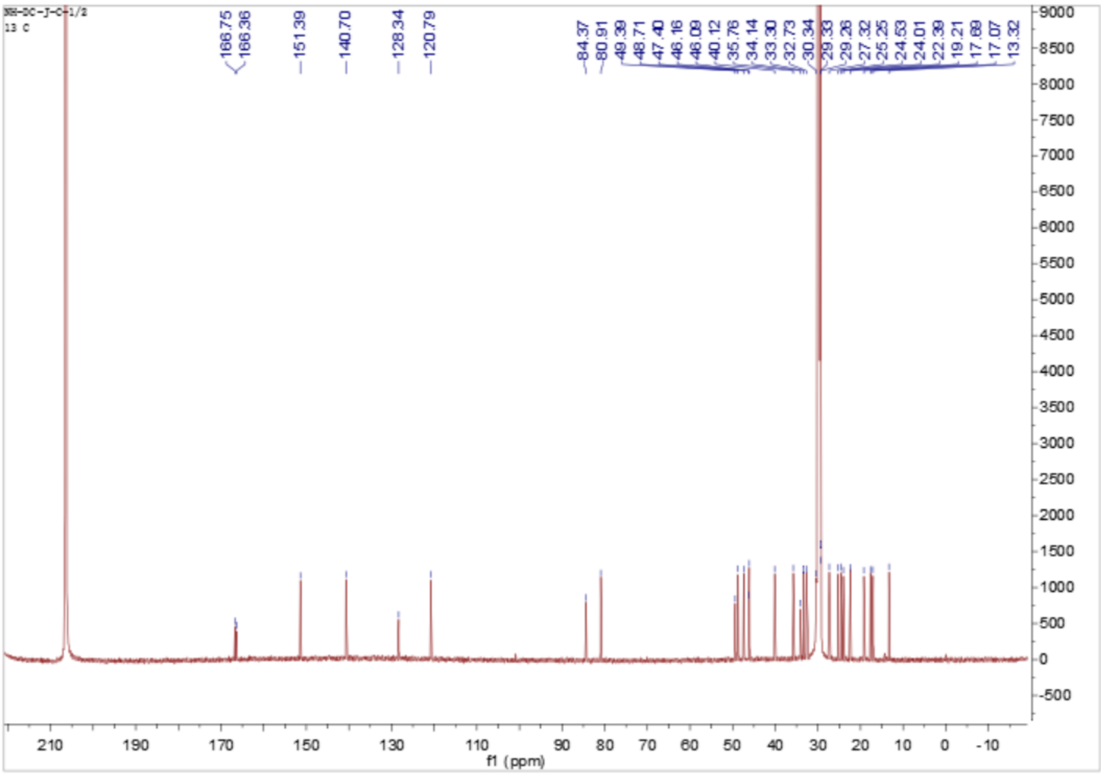

Figure S92. The ^{13}C NMR spectrum of 11 in Acetone-d_6

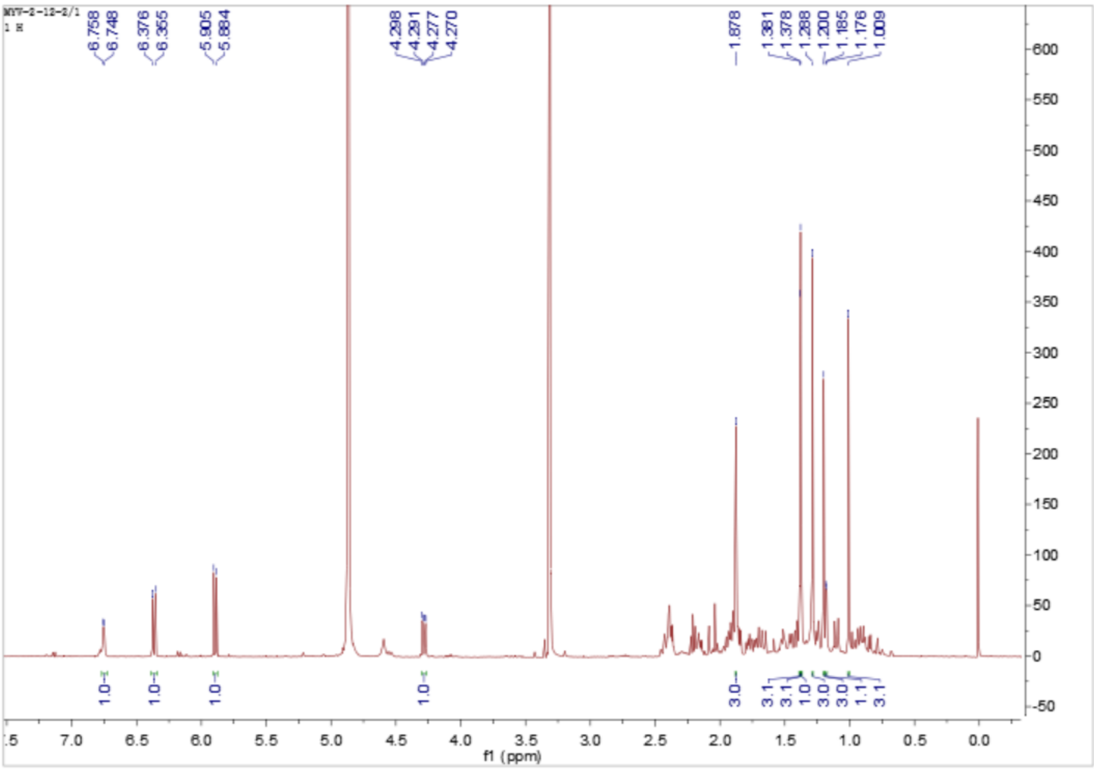

Figure S93. The ^{1}H NMR spectrum of 12 in CD_3OD

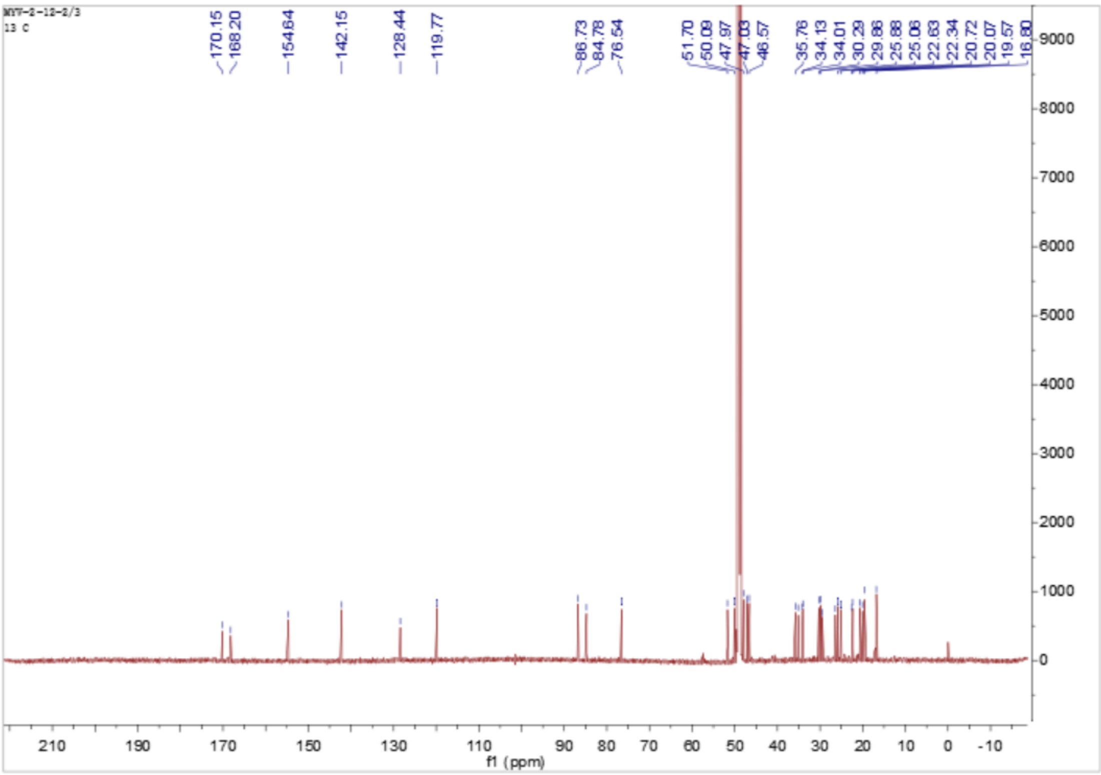

Figure S94. The ^{13}C NMR spectrum of 12 in CD_3OD

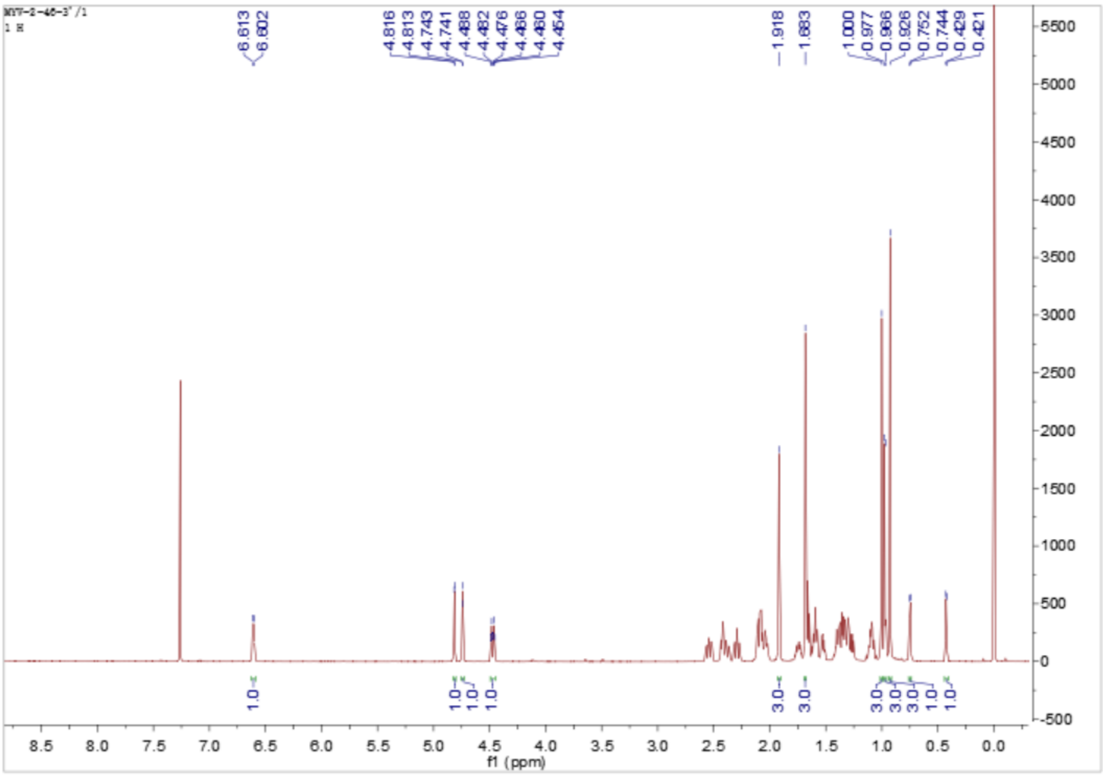

Figure S95. The ^{1}H NMR spectrum of 13 in $CDCl_3$

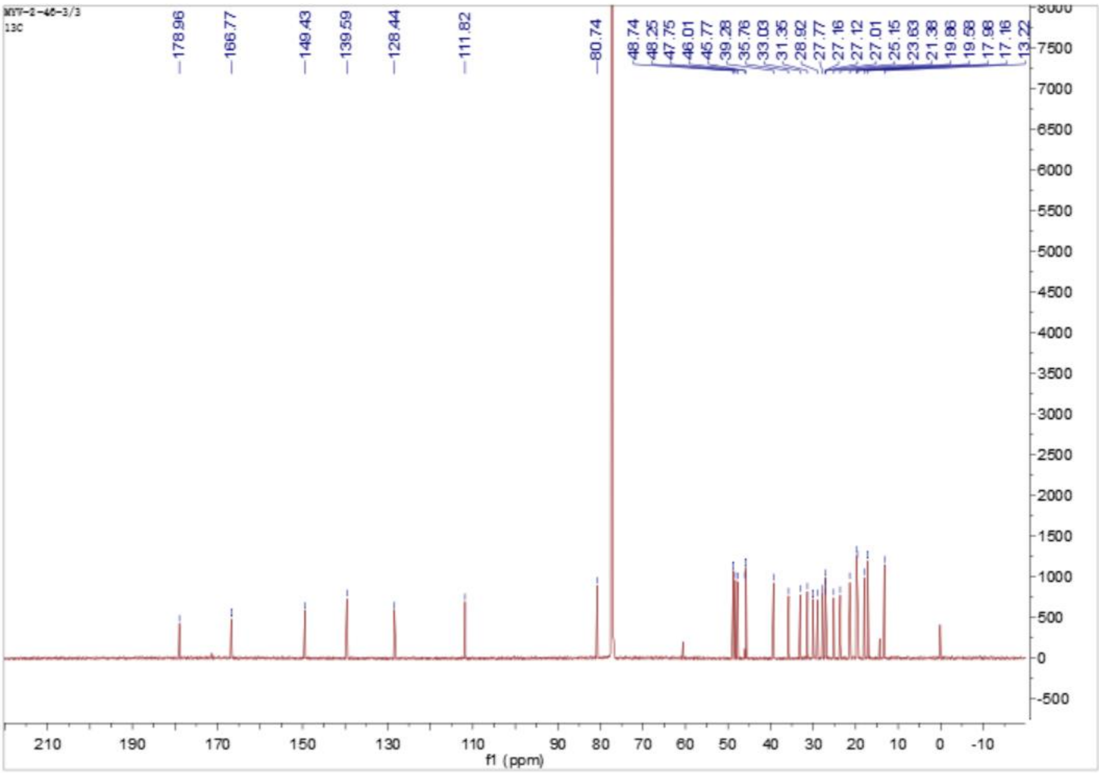

Figure S96. The ^{13}C NMR spectrum of 13 in $CDCl_3$

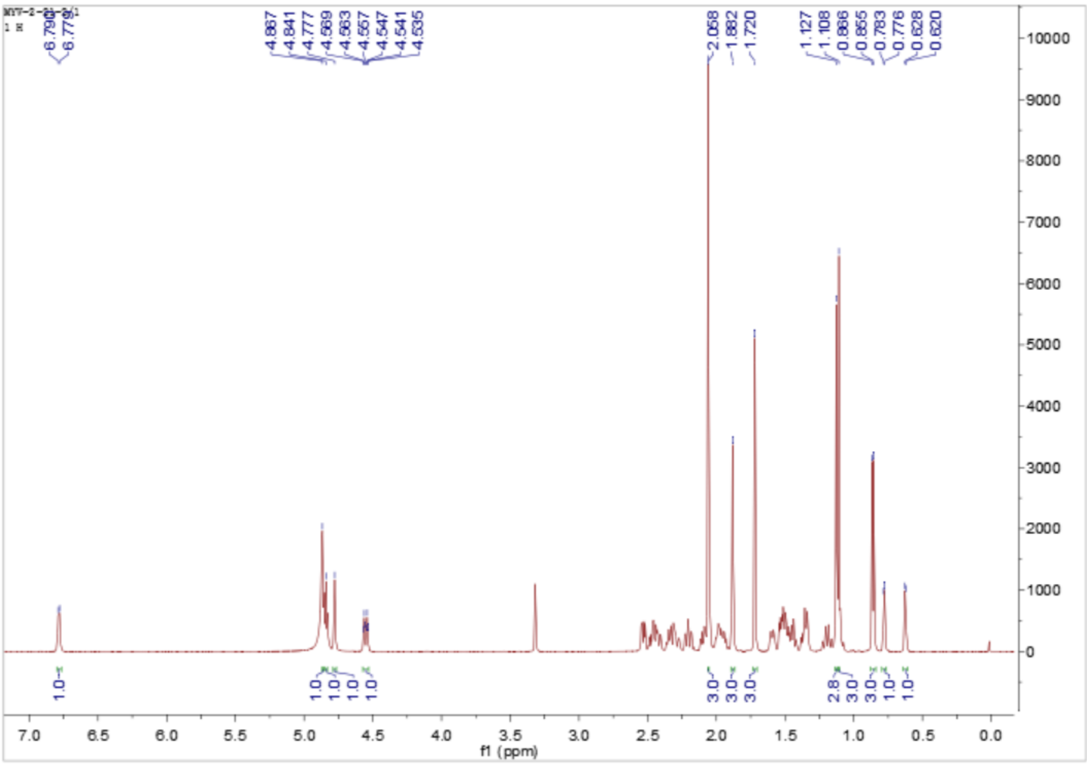

Figure S97. The ^{1}H NMR spectrum of 14 in CD_3OD

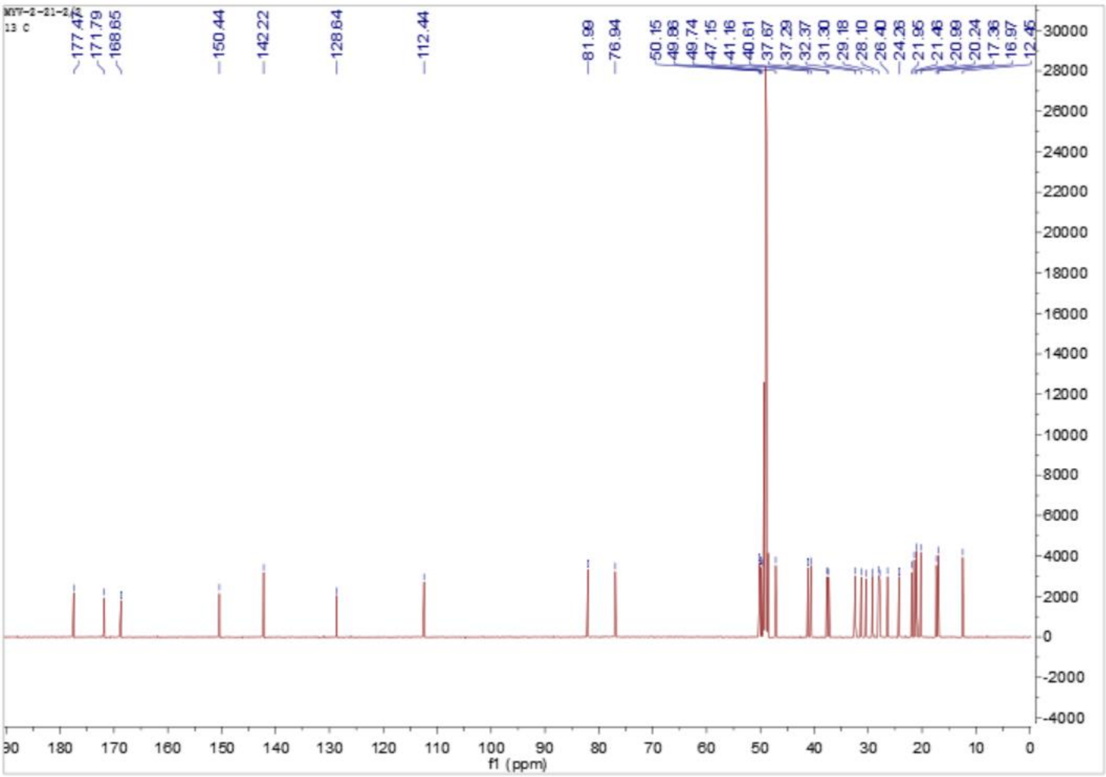

Figure S98. The ^{13}C NMR spectrum of 14 in CD_3OD

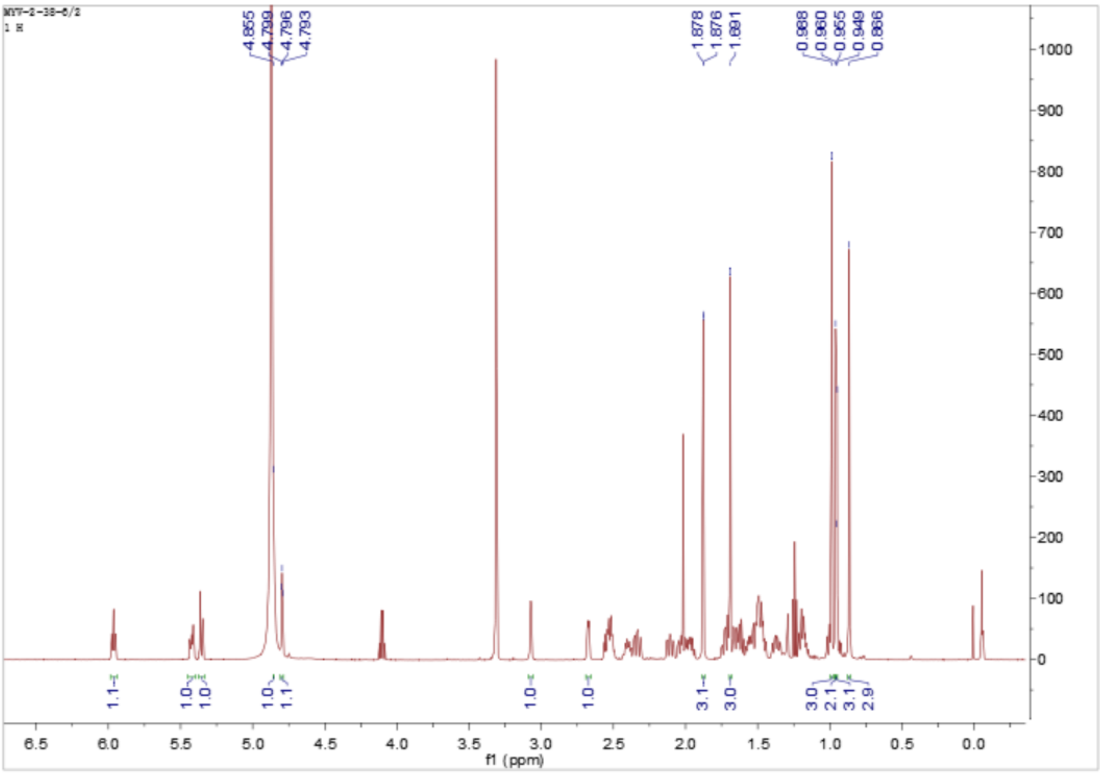

Figure S99. The ^{1}H NMR spectrum of 15 in CD_3OD

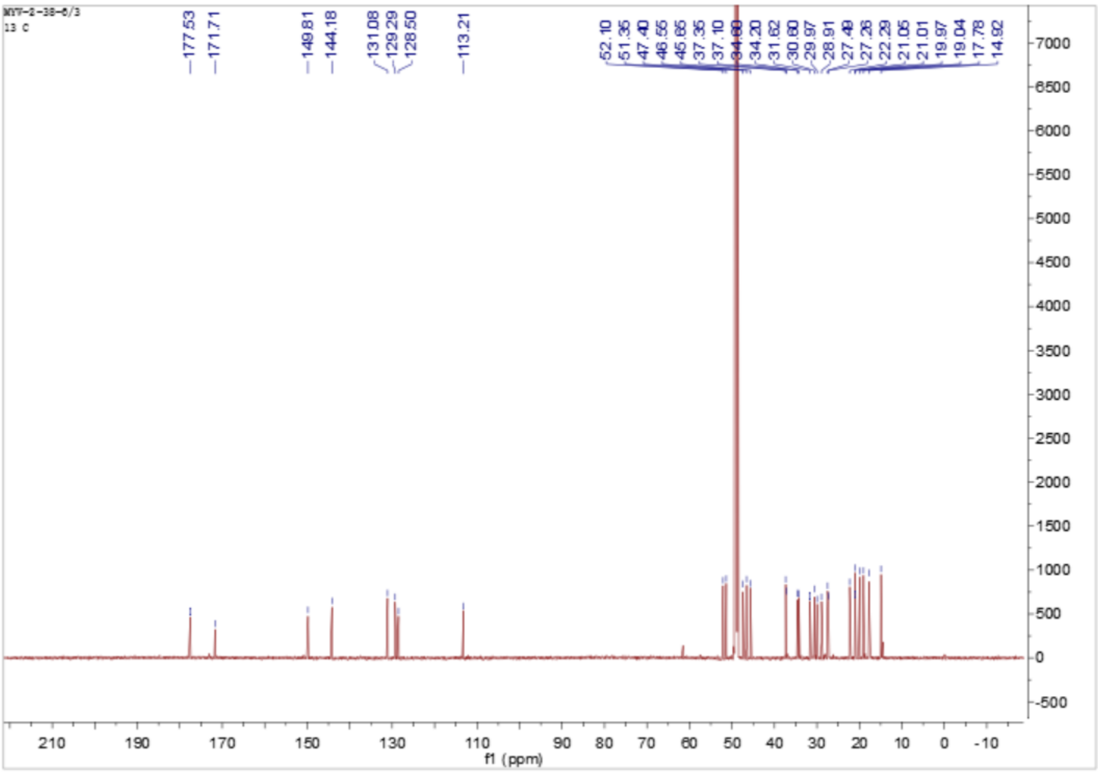

Figure S100. The ^{13}C NMR spectrum of 15 in CD_3OD

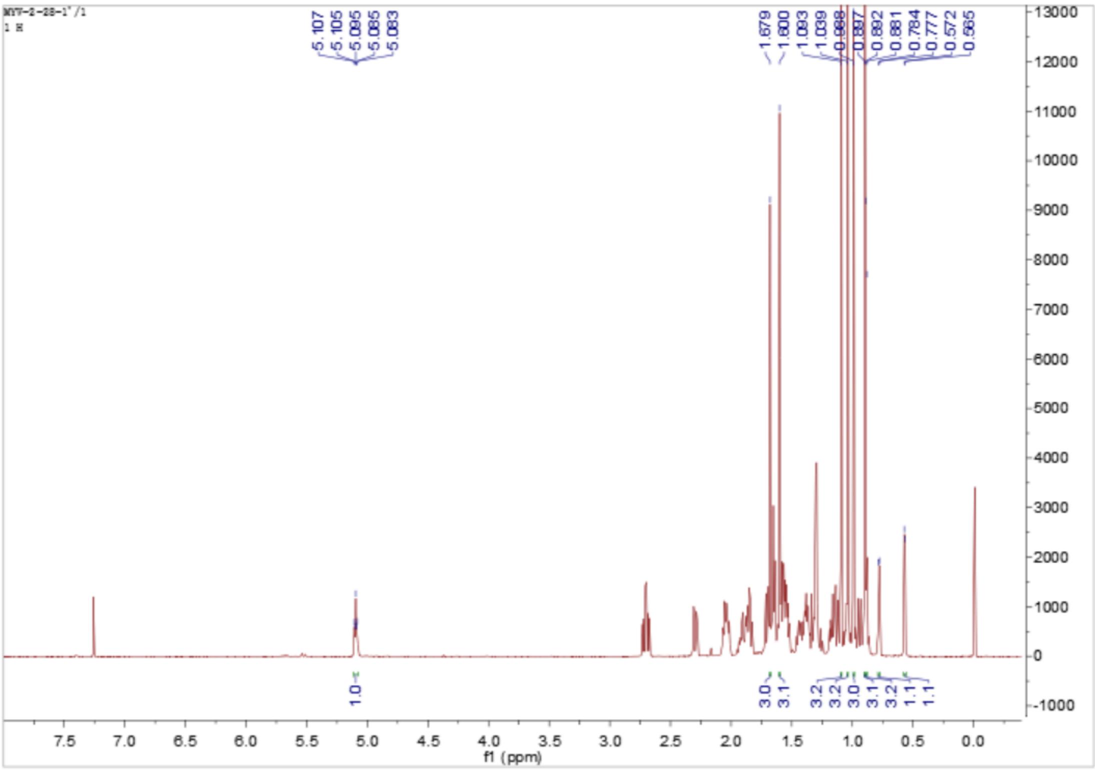

Figure S101. The ^{1}H NMR spectrum of 16 in $CDCl_3$

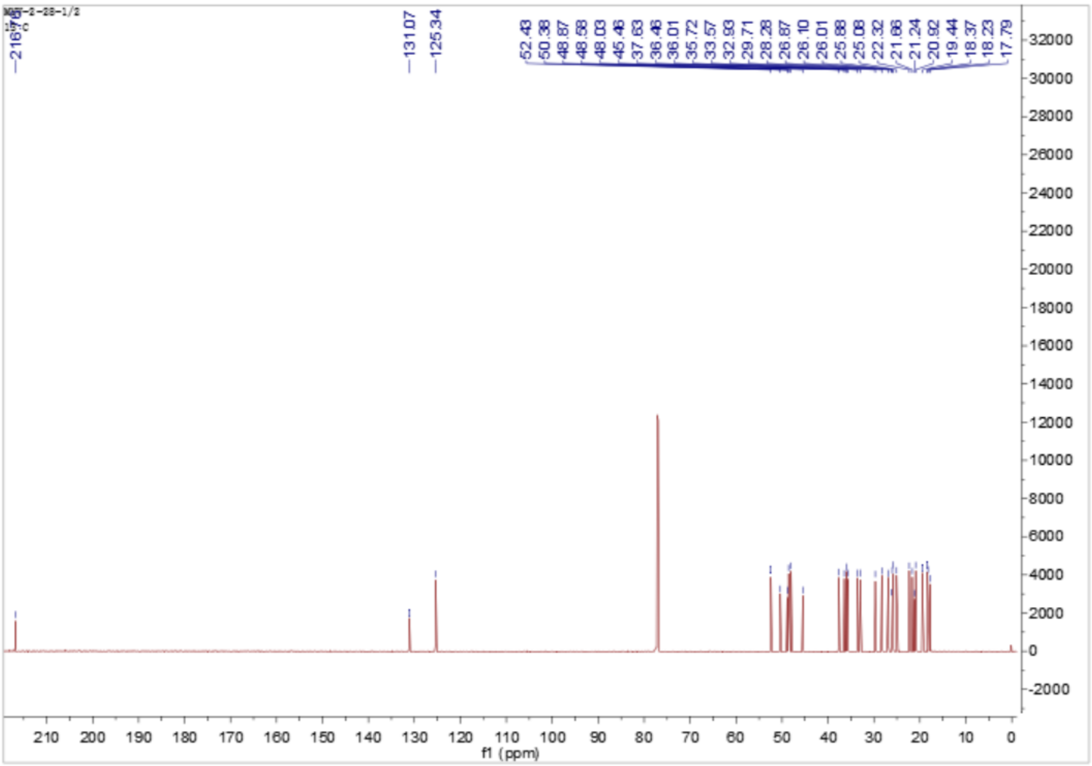

Figure S102. The ^{13}C NMR spectrum of 16 in $CDCl_3$

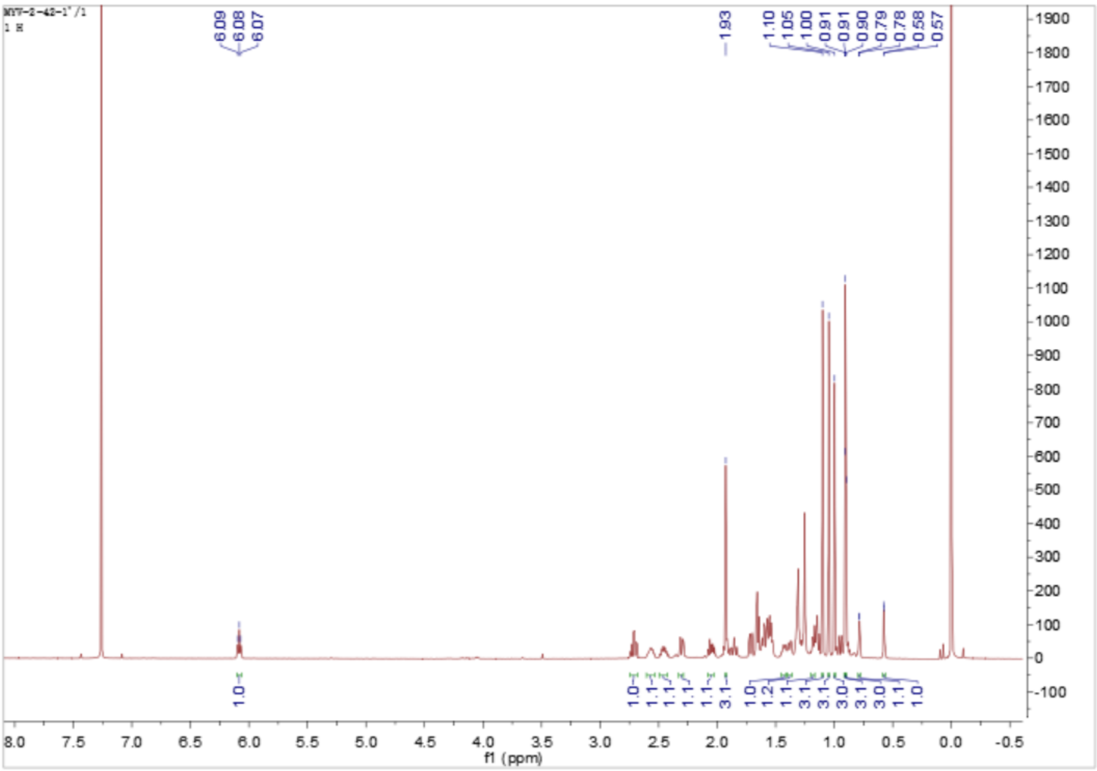

Figure S103. The ^{1}H NMR spectrum of 17 in $CDCl_3$

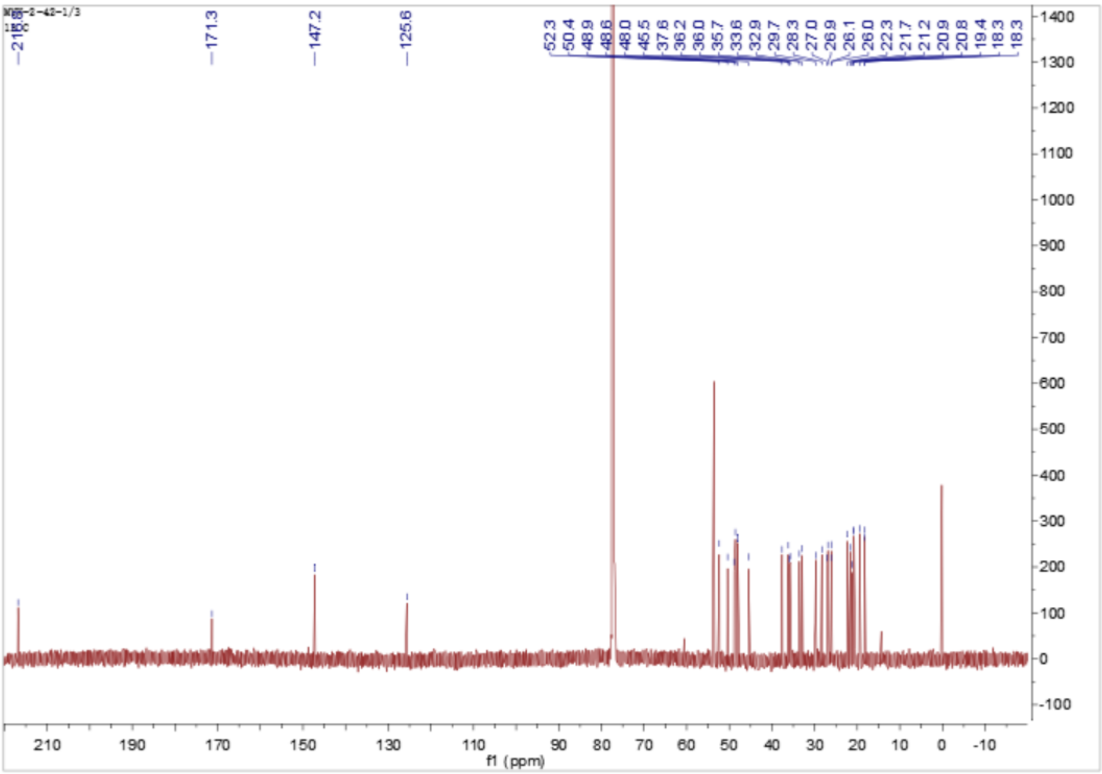

Figure S104. The ^{13}C NMR spectrum of 17 in $CDCl_3$

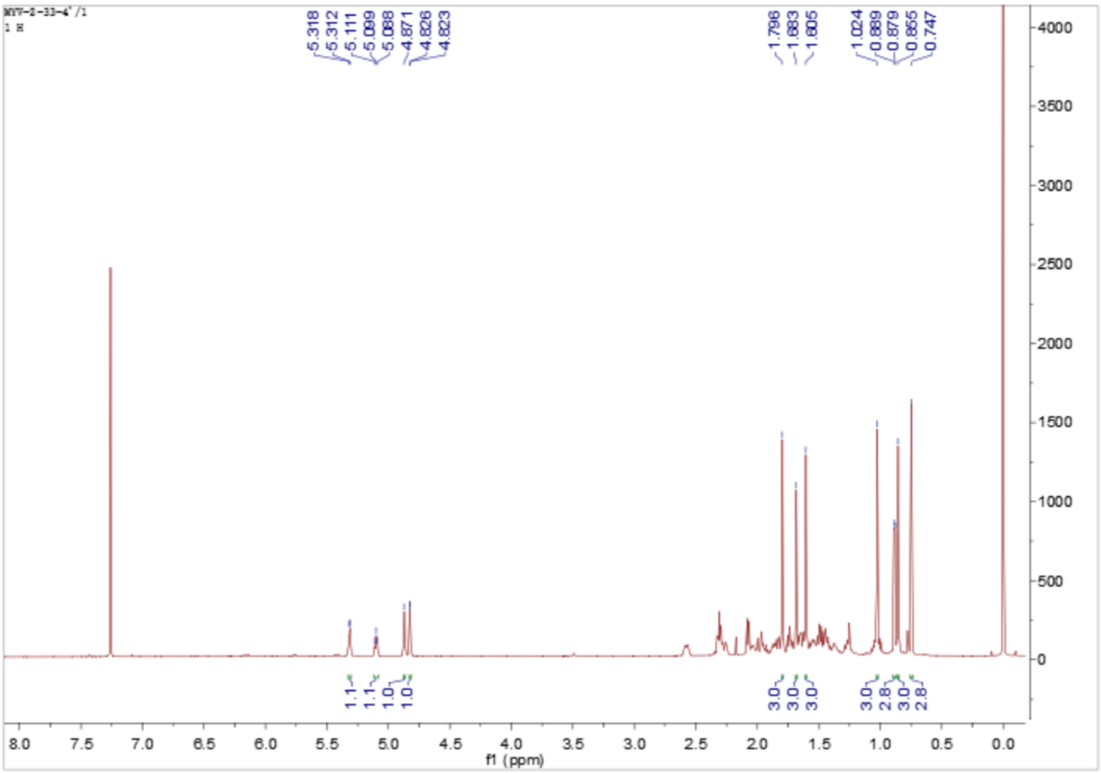

Figure S105. The ^{1}H NMR spectrum of 18 in $CDCl_3$

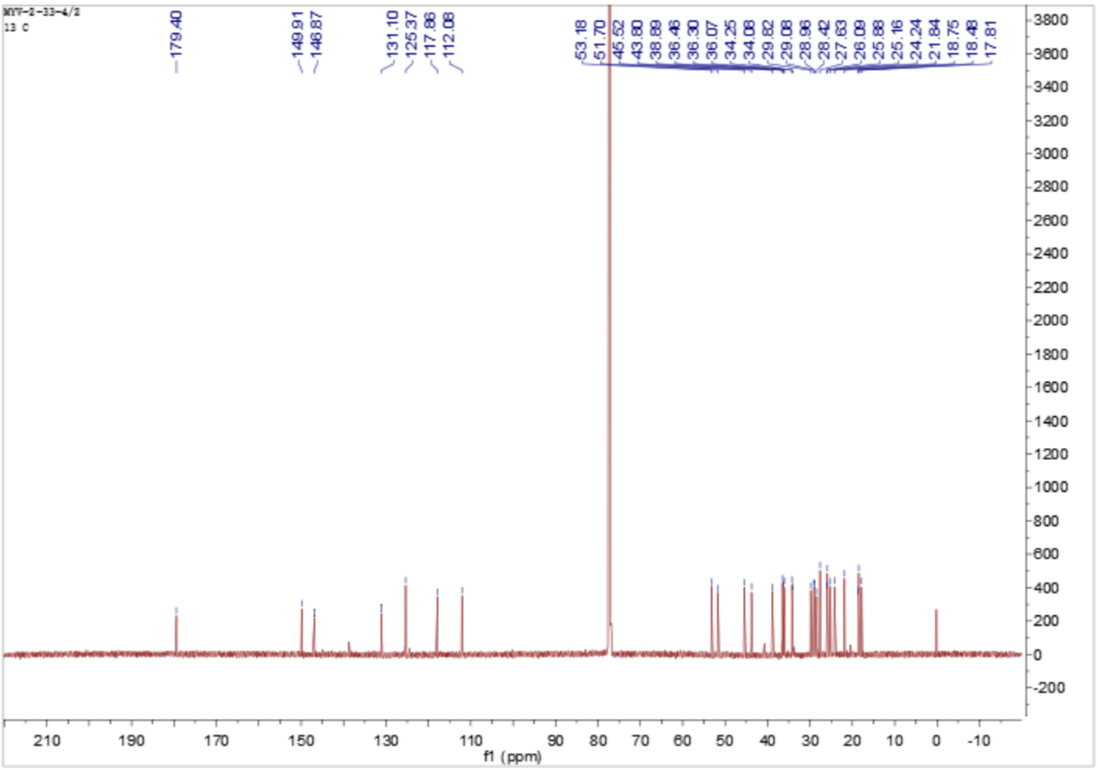

Figure S106. The ^{13}C NMR spectrum of 18 in $CDCl_3$

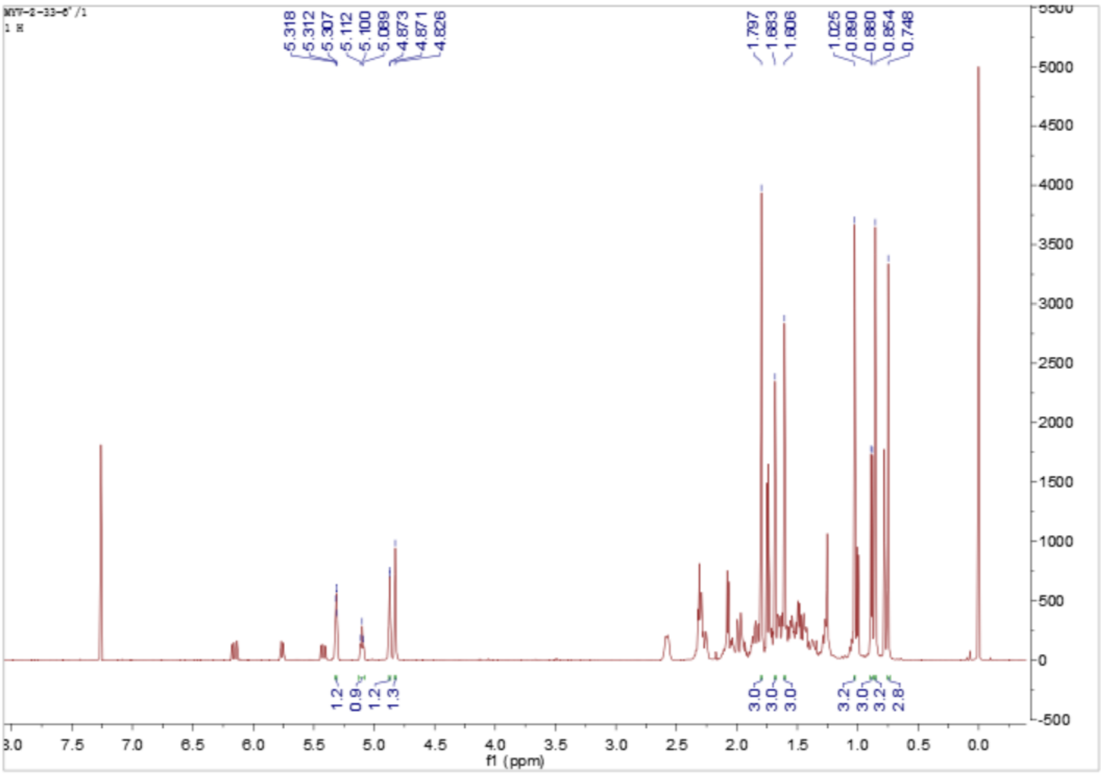

Figure S107. The ^{1}H NMR spectrum of 19 in $CDCl_3$

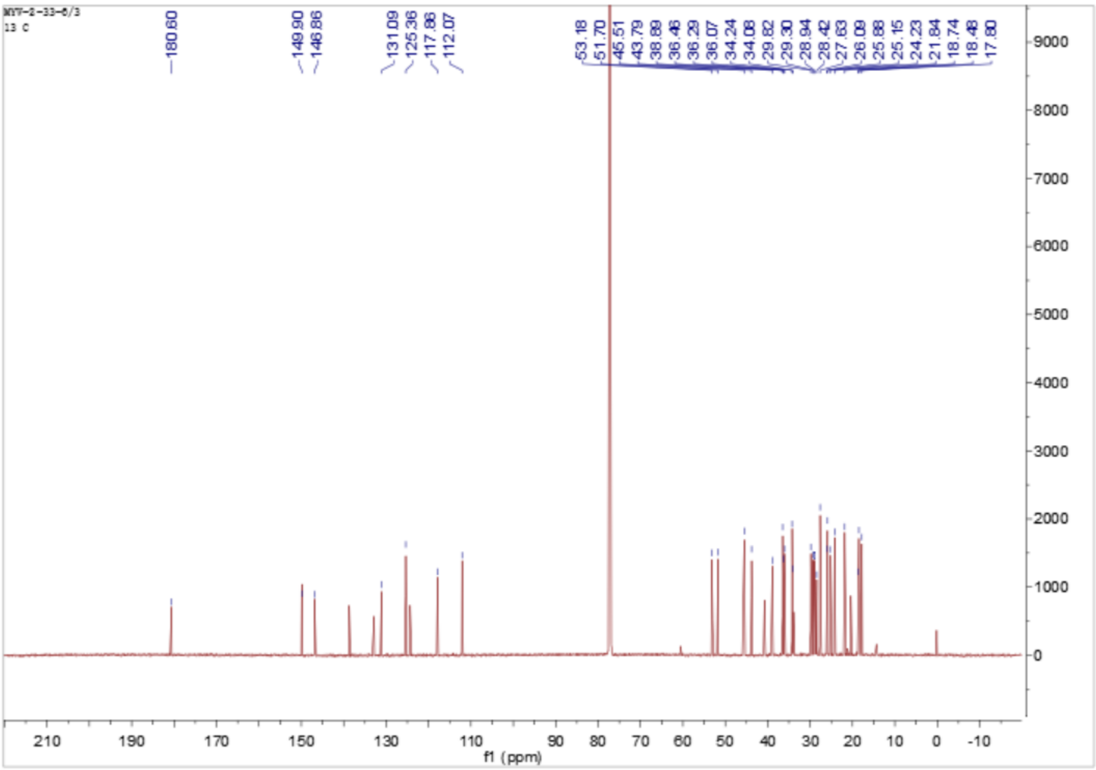

Figure S108. The ^{13}C NMR spectrum of 19 in $CDCl_3$

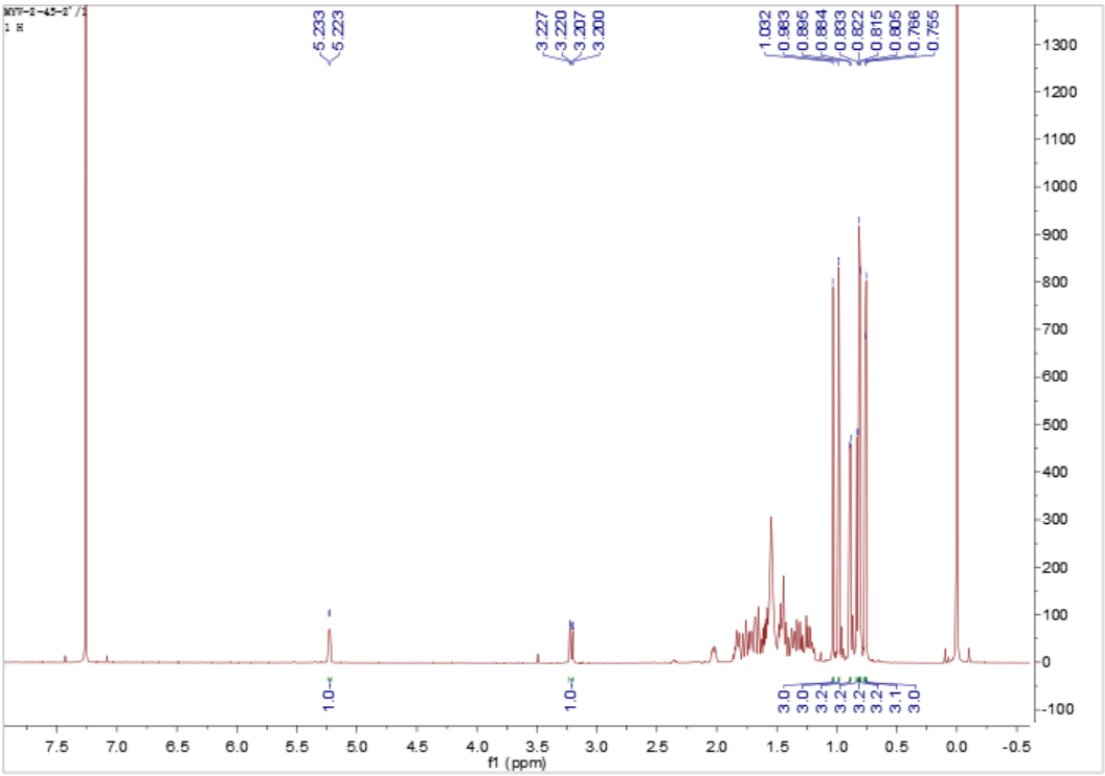

Figure S109. The ^{1}H NMR spectrum of 20 in $CDCl_3$

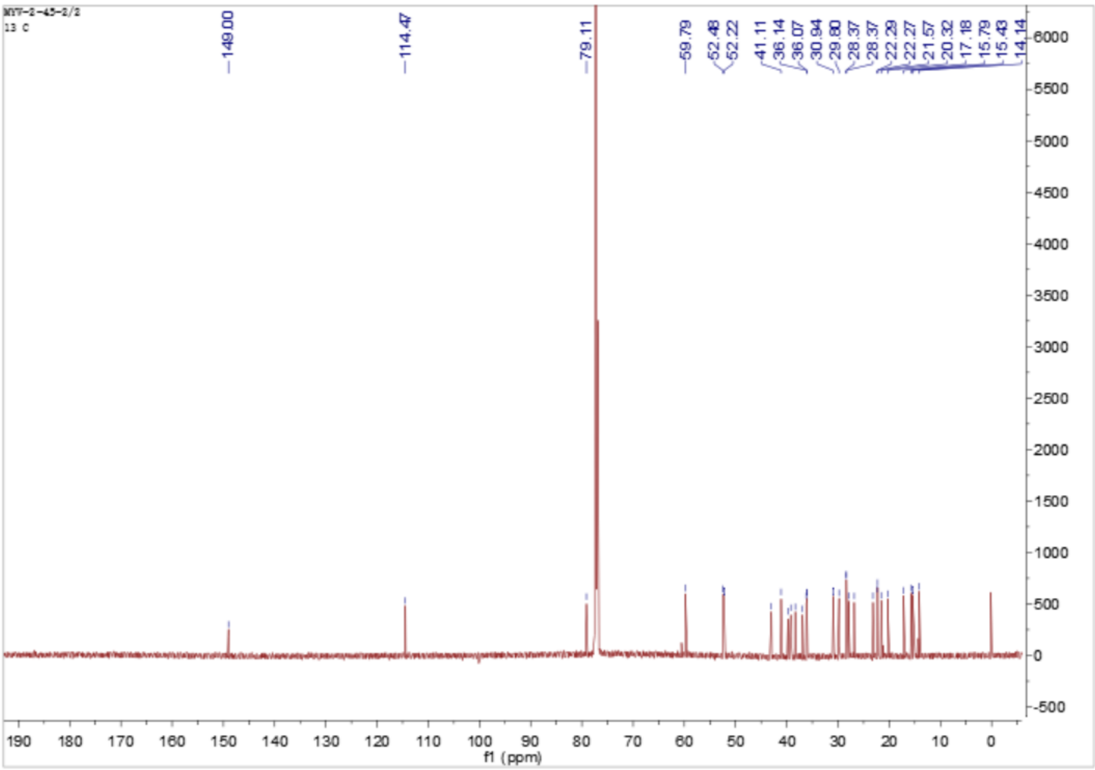

Figure S110. The ^{13}C NMR spectrum of 20 in $CDCl_3$

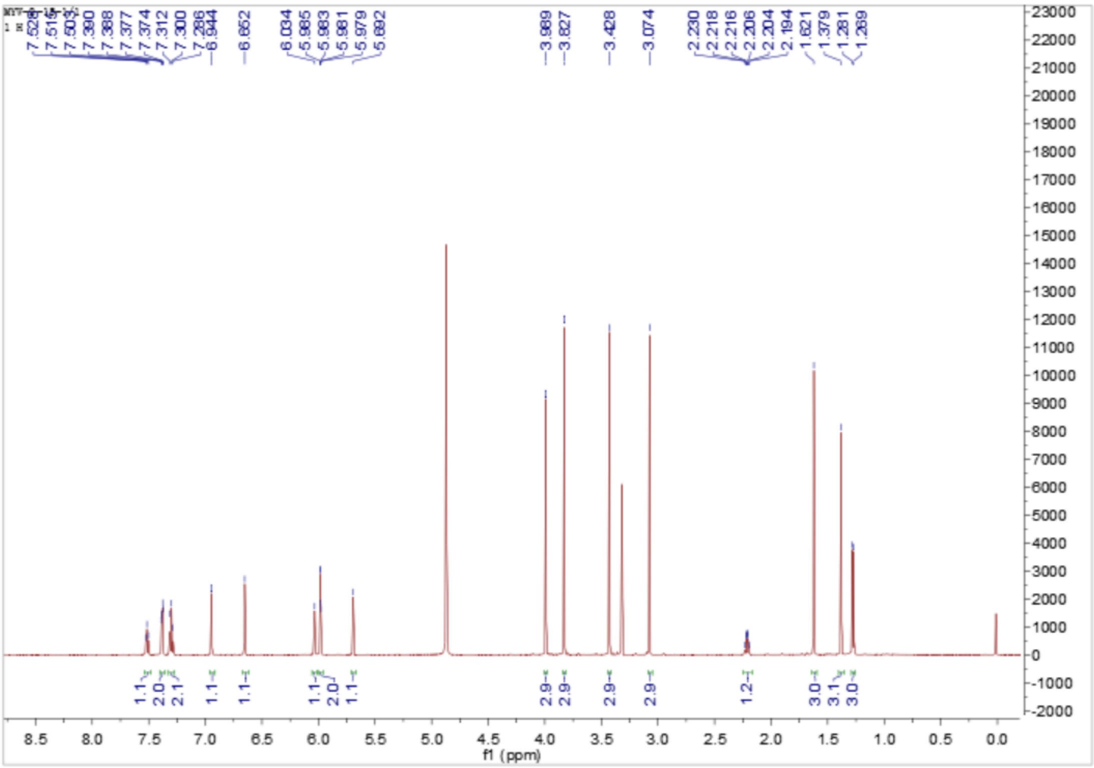

Figure S111. The ^{1}H NMR spectrum of 21 in CD_3OD

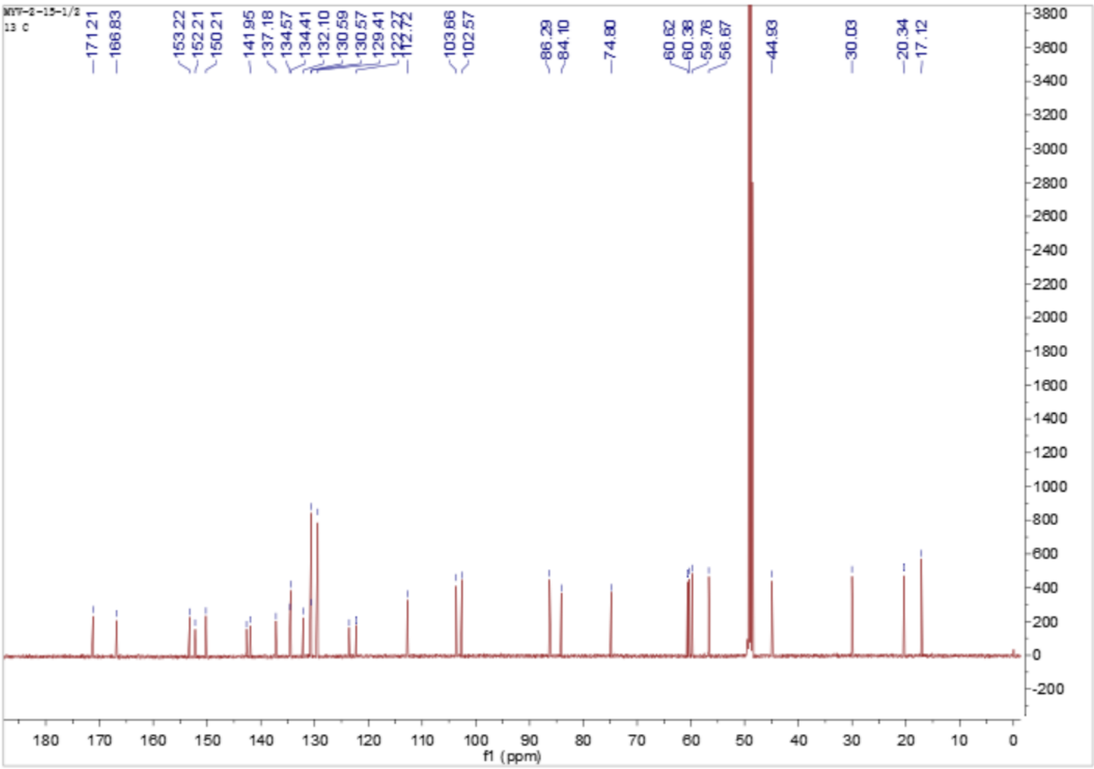

Figure S112. The ^{13}C NMR spectrum of 21 in CD_3OD

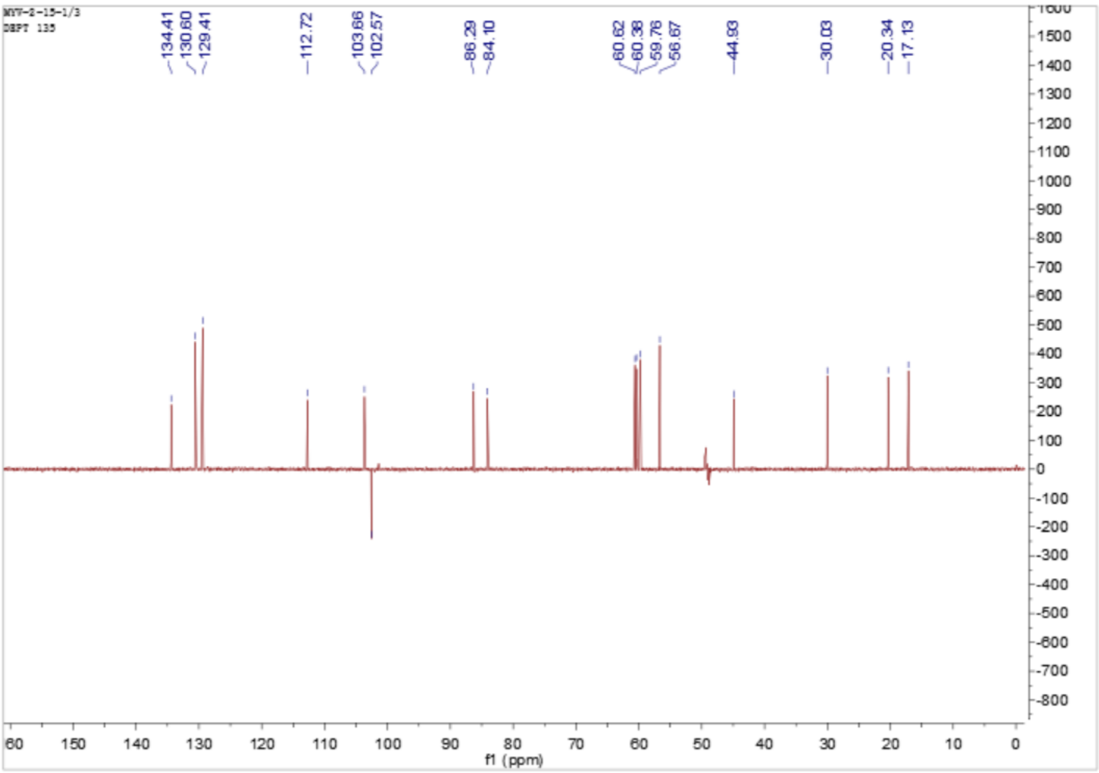

Figure S113. The DEPT spectrum of 21 in CD_3OD

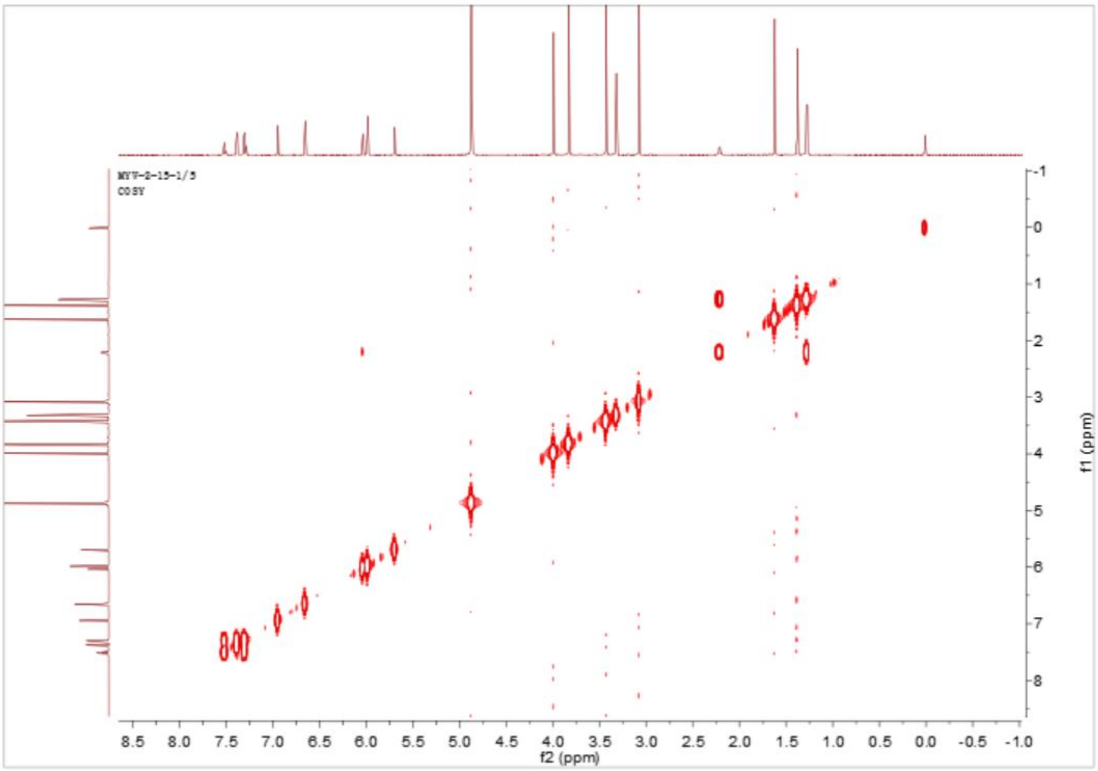

Figure S114. The ^1H-^1H COSY spectrum of 21 in CD_3OD

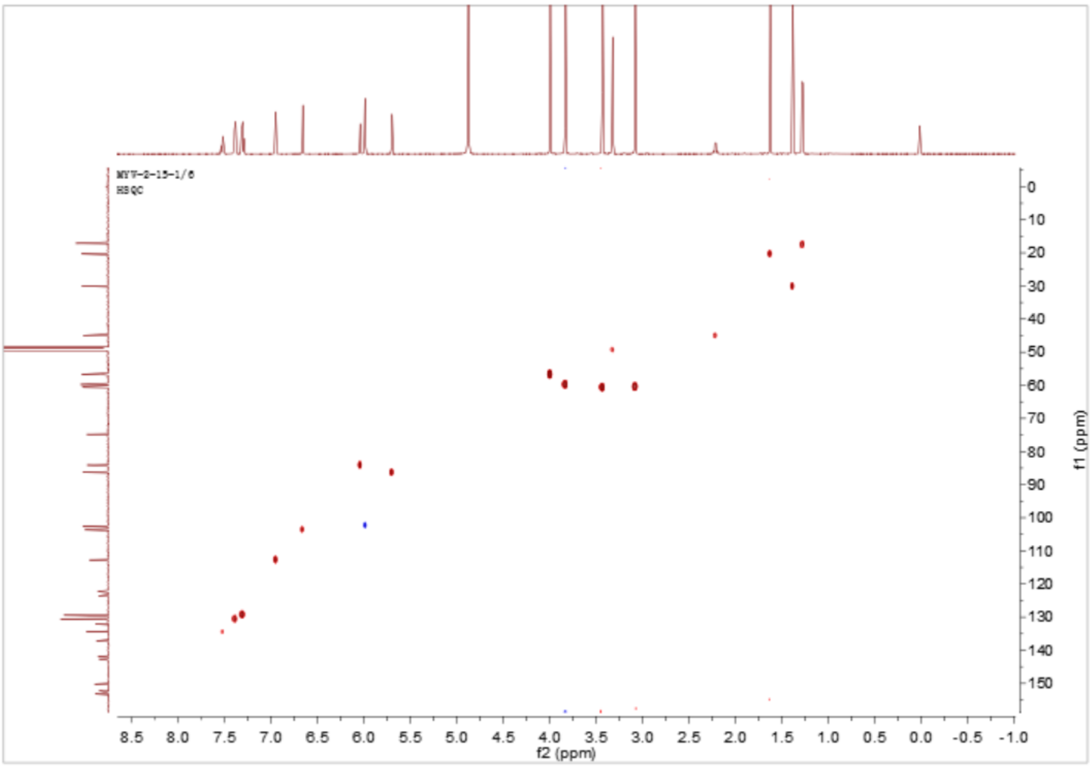

Figure S115. The HSQC spectrum of 21 in CD_3OD

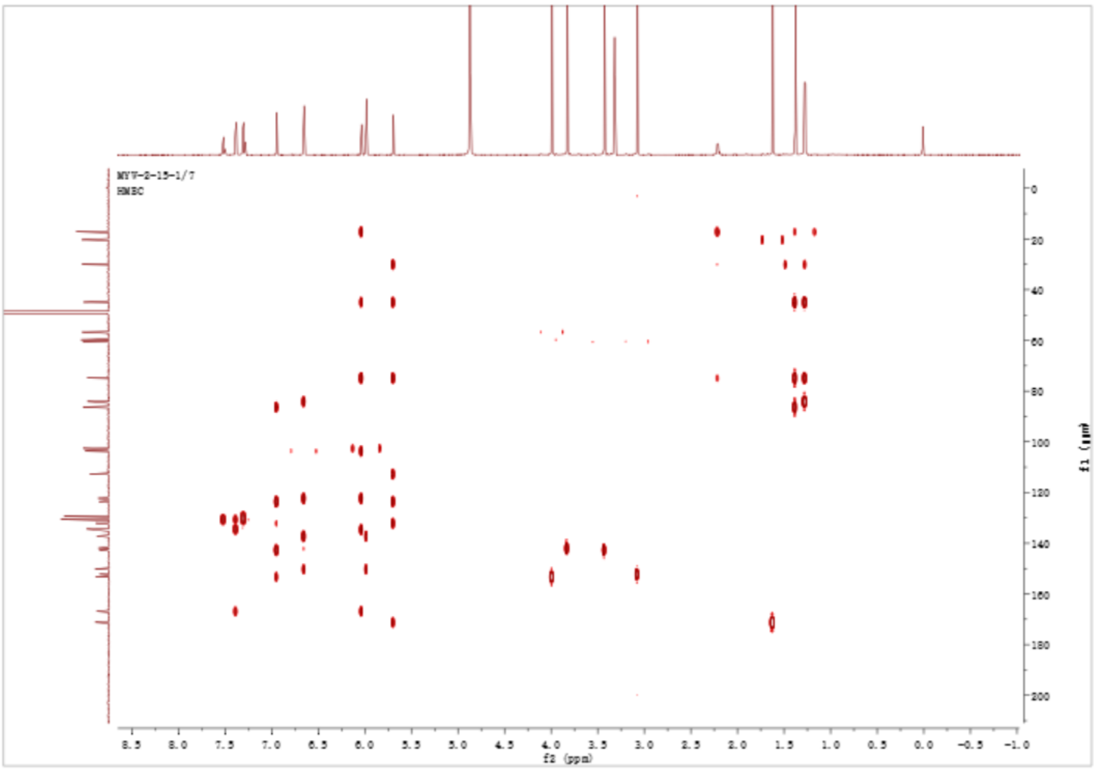

Figure S116. The HMBC spectrum of 21 in CD_3OD

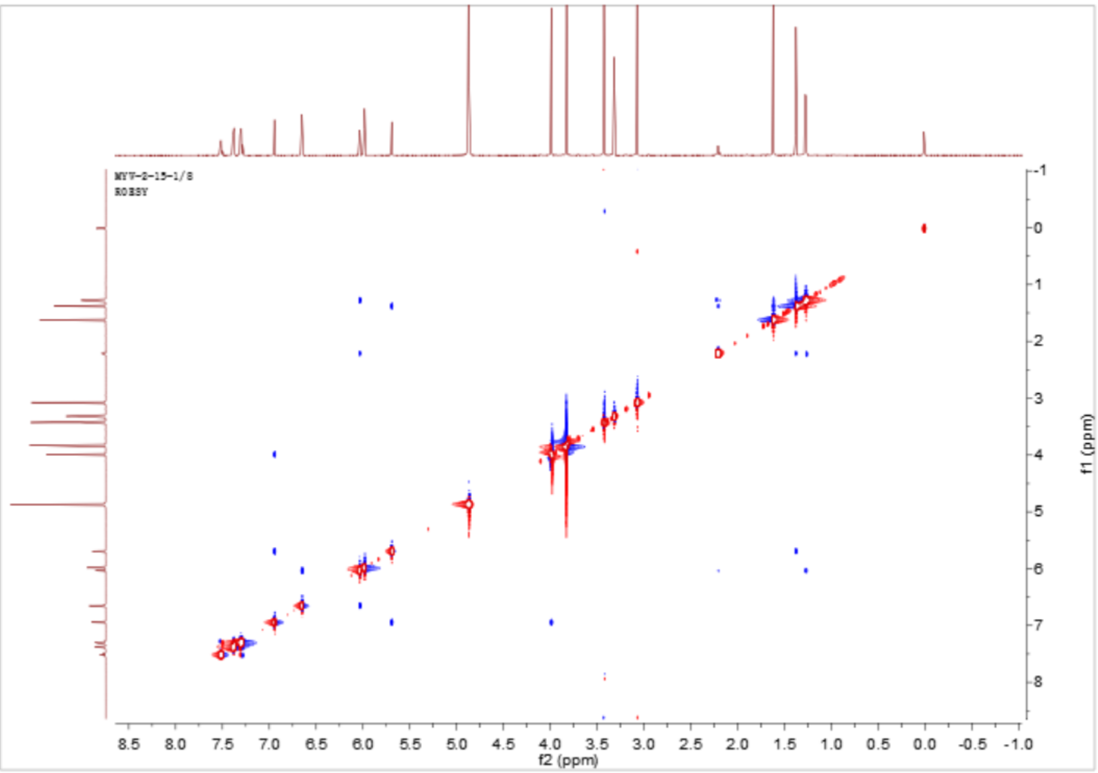

Figure S117. The ROESY spectrum of 21 in CD_3OD

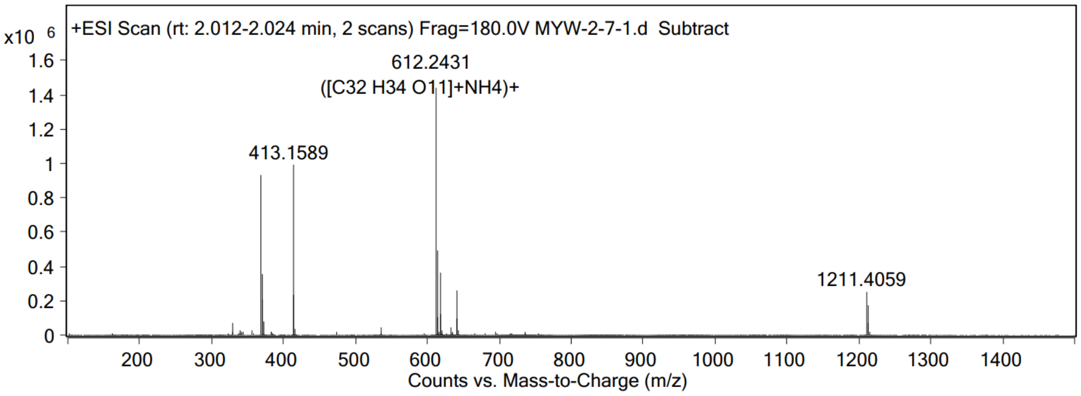

Formula Calculator Results

Formula	Best	Mass	Tgt Mass	Diff (ppm)	Ion Species	Score
C32 H34 O11	True	594.2091	594.2101	1.72	C32 H38 N O11	97.12

Figure S118. The HRESIMS spectrum of 21 in CD_3OD

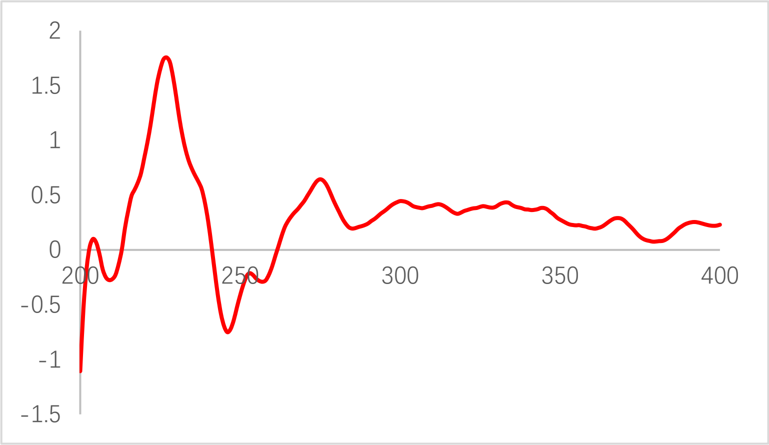

Figure S119. The ECD spectrum of 21 in MeOH

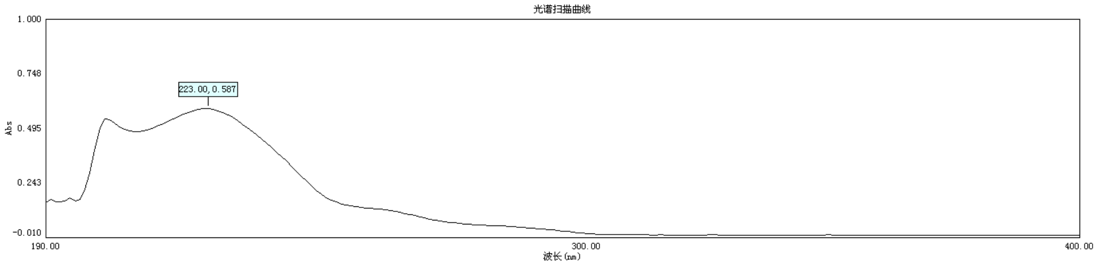

Figure S120. The UV spectrum of 21 in MeOH

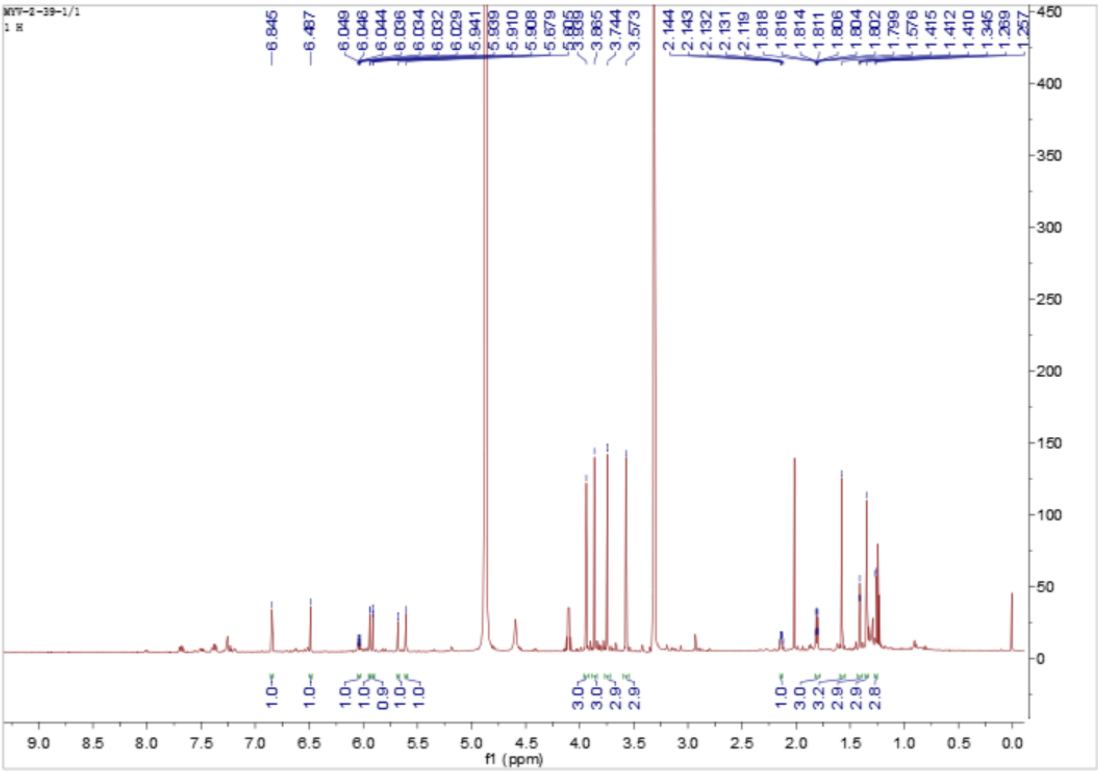

Figure S121. The ^{1}H NMR spectrum of 22 in CD_3OD

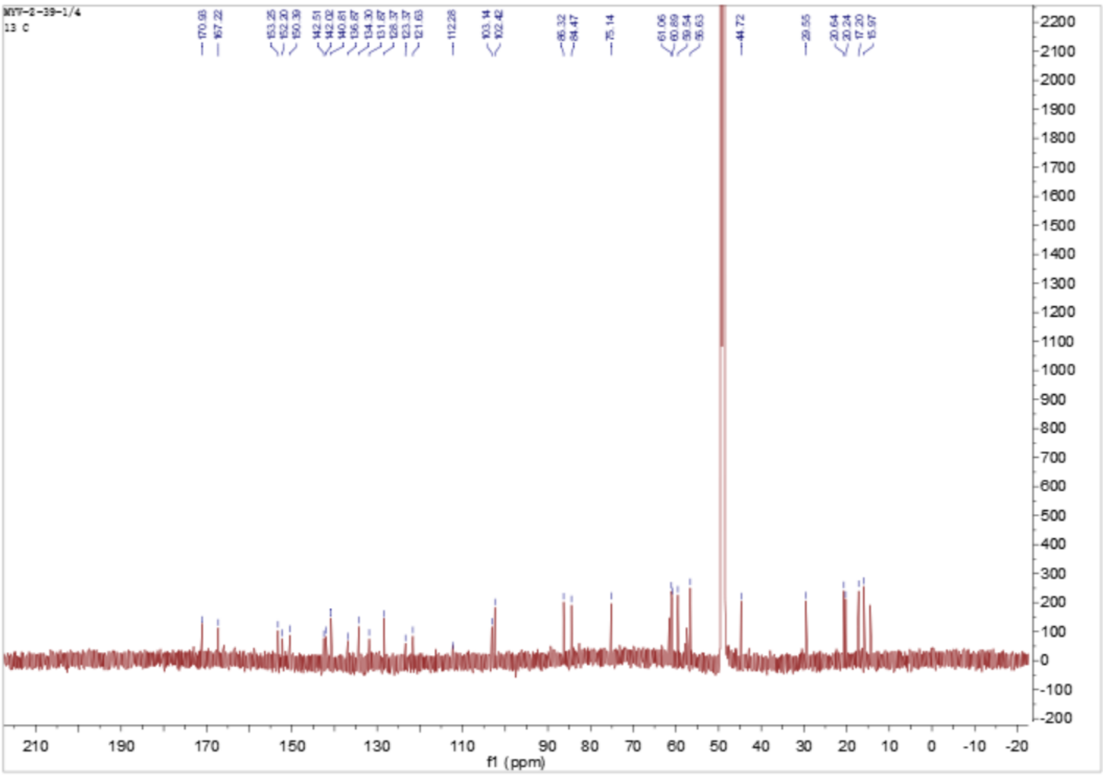

Figure S122. The ^{13}C NMR spectrum of 22 in CD_3OD

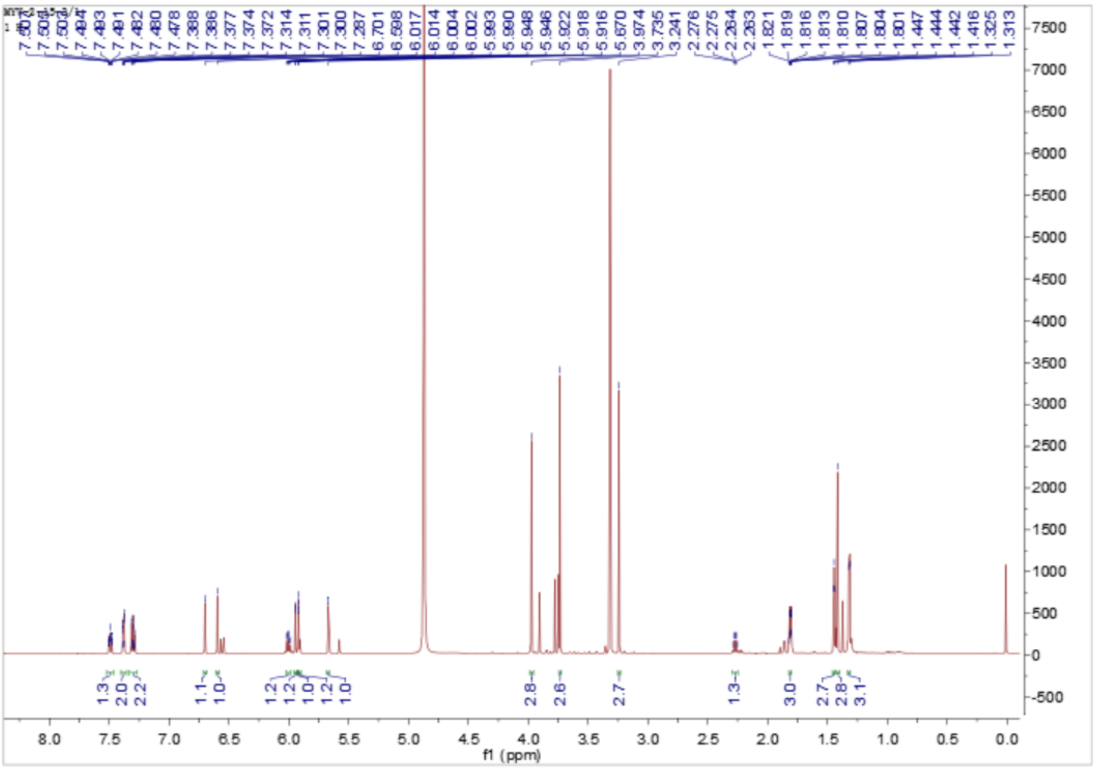

Figure S123. The ^{1}H NMR spectrum of 23 in CD_3OD

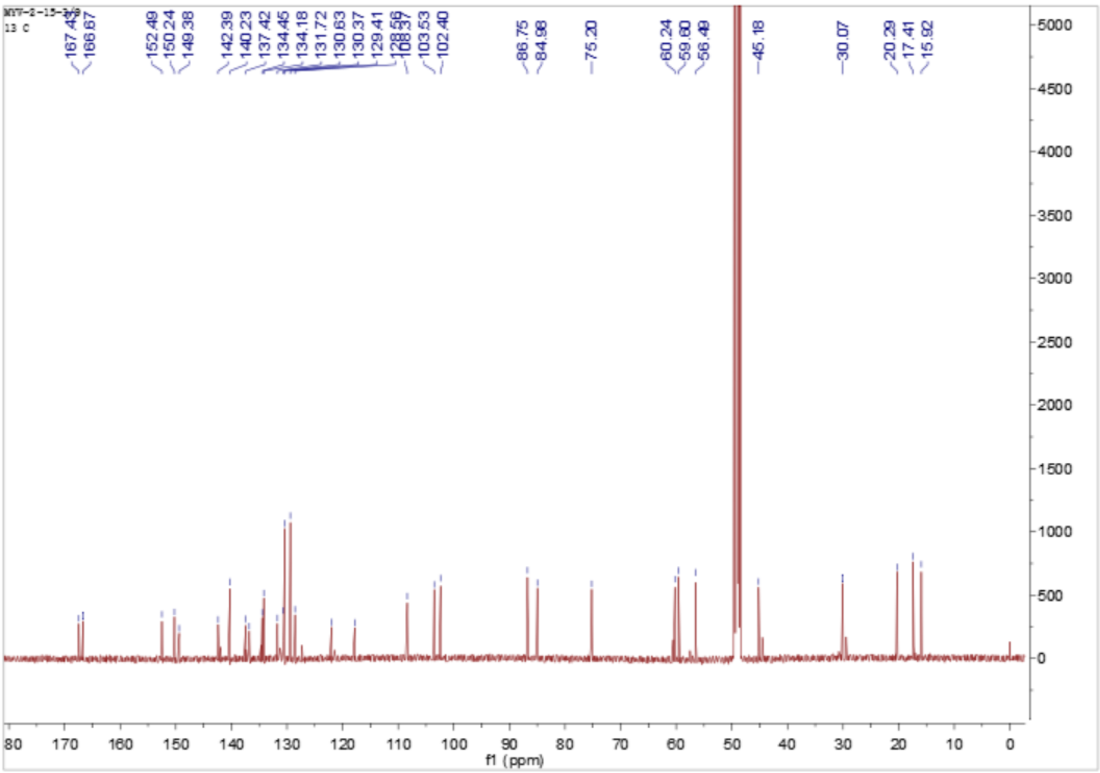

Figure S124. The ^{13}C NMR spectrum of 23 in CD_3OD

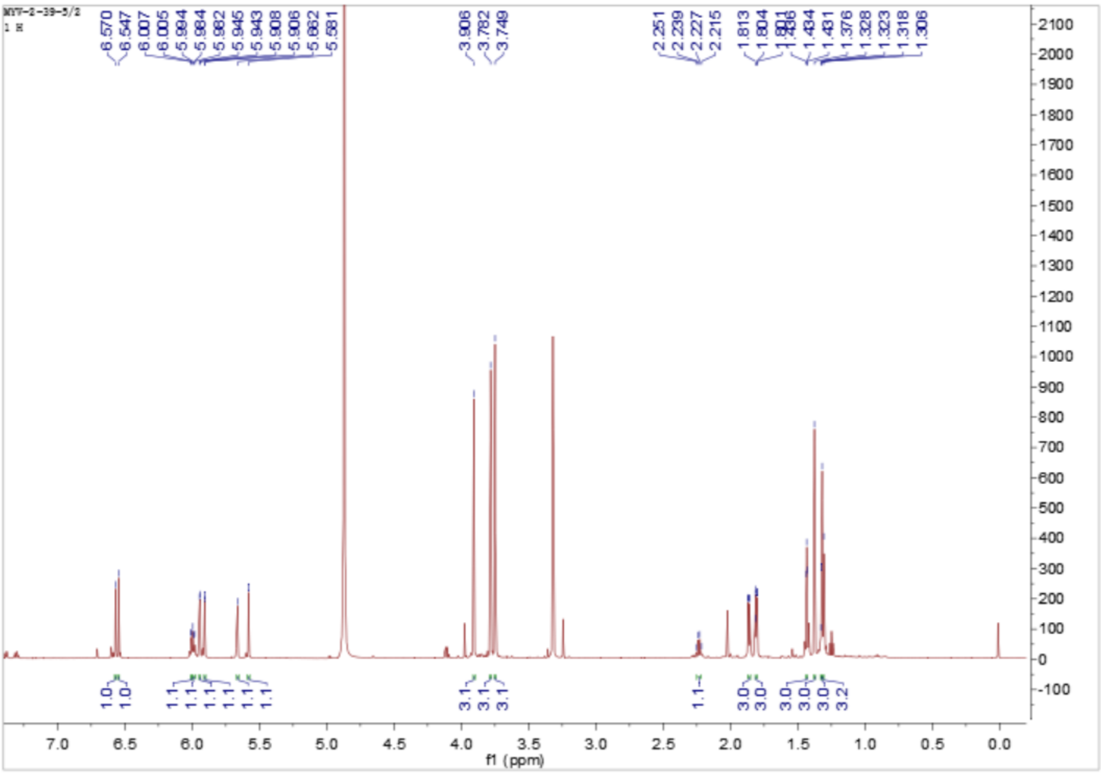

Figure S125. The ^{1}H NMR spectrum of 24 in CD_3OD

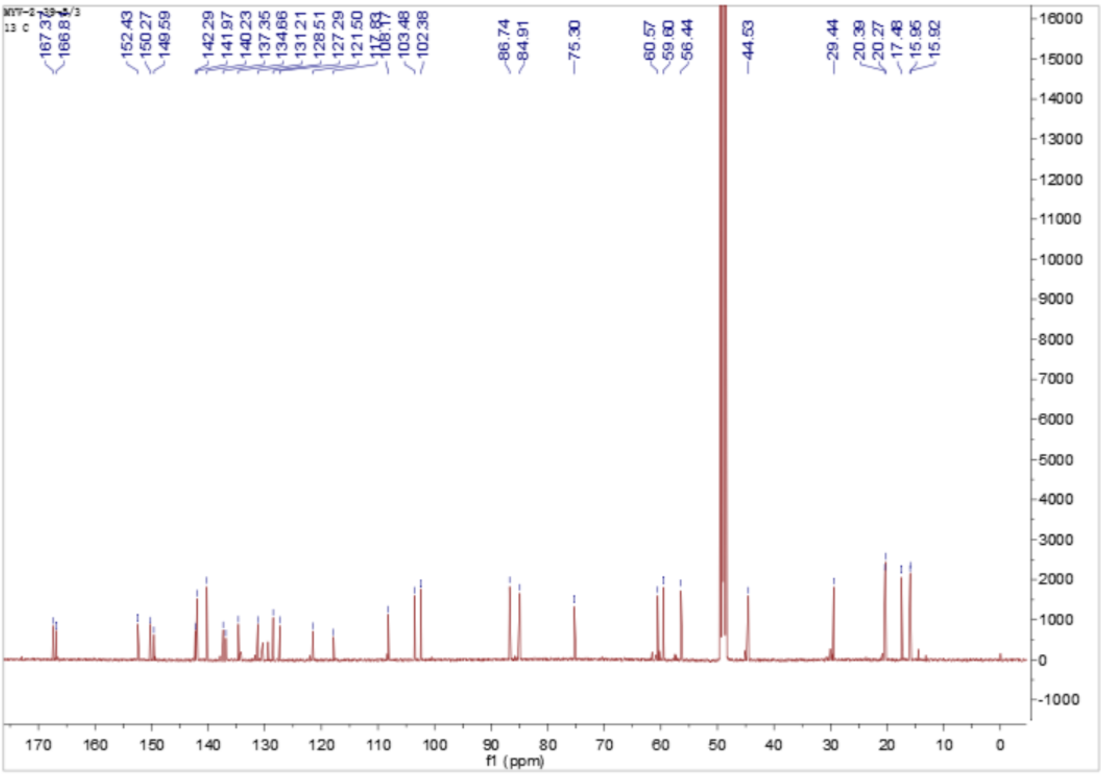

Figure S126. The ^{13}C NMR spectrum of 24 in CD_3OD

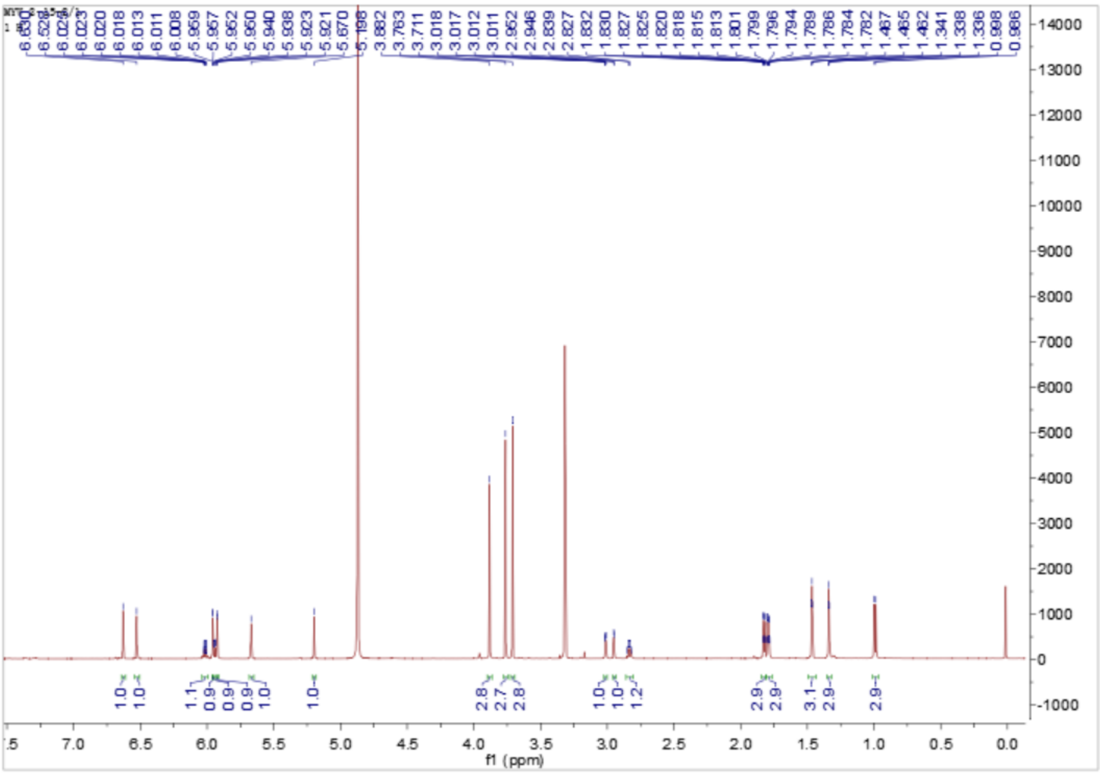

Figure S127. The ^{1}H NMR spectrum of 25 in CD_3OD

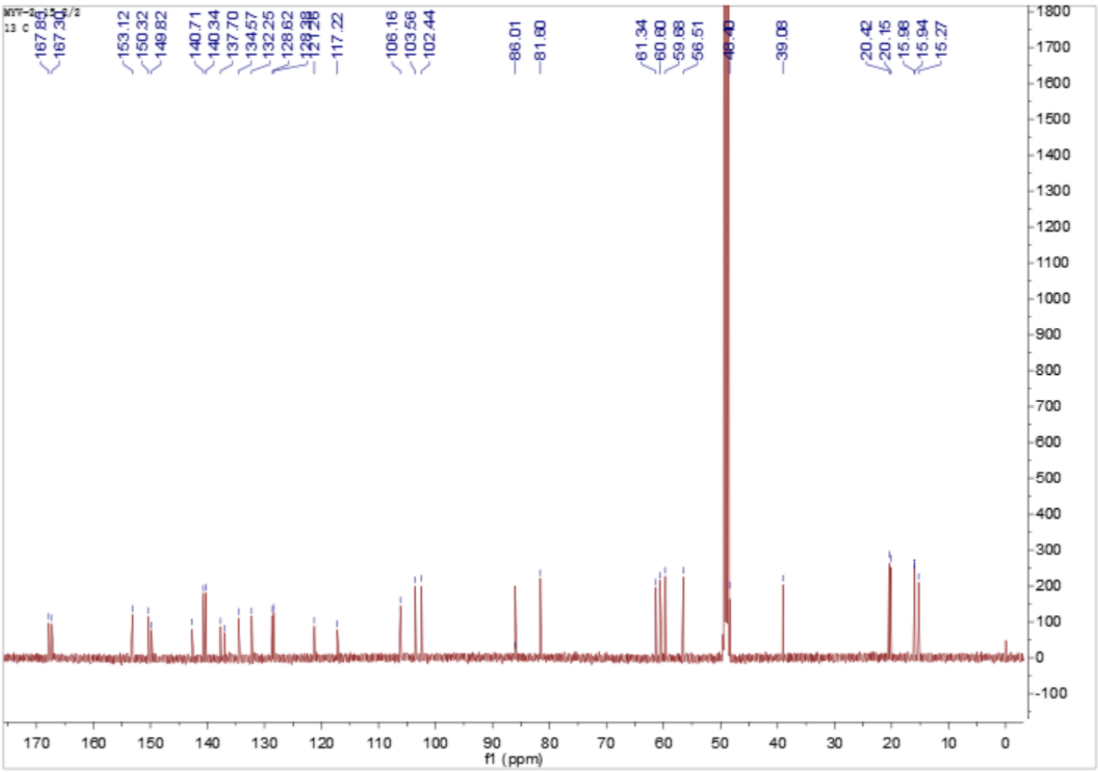

Figure S128. The ^{13}C NMR spectrum of 25 in CD_3OD

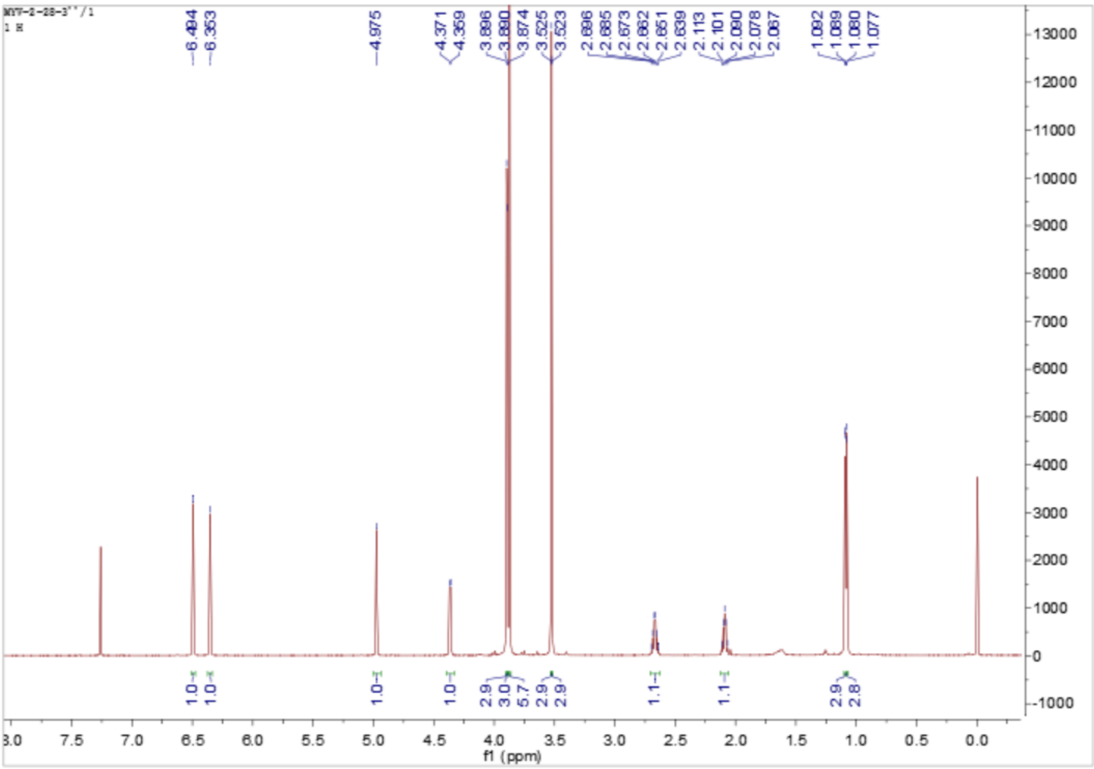

Figure S129. The ^{1}H NMR spectrum of 26 in CD_3OD

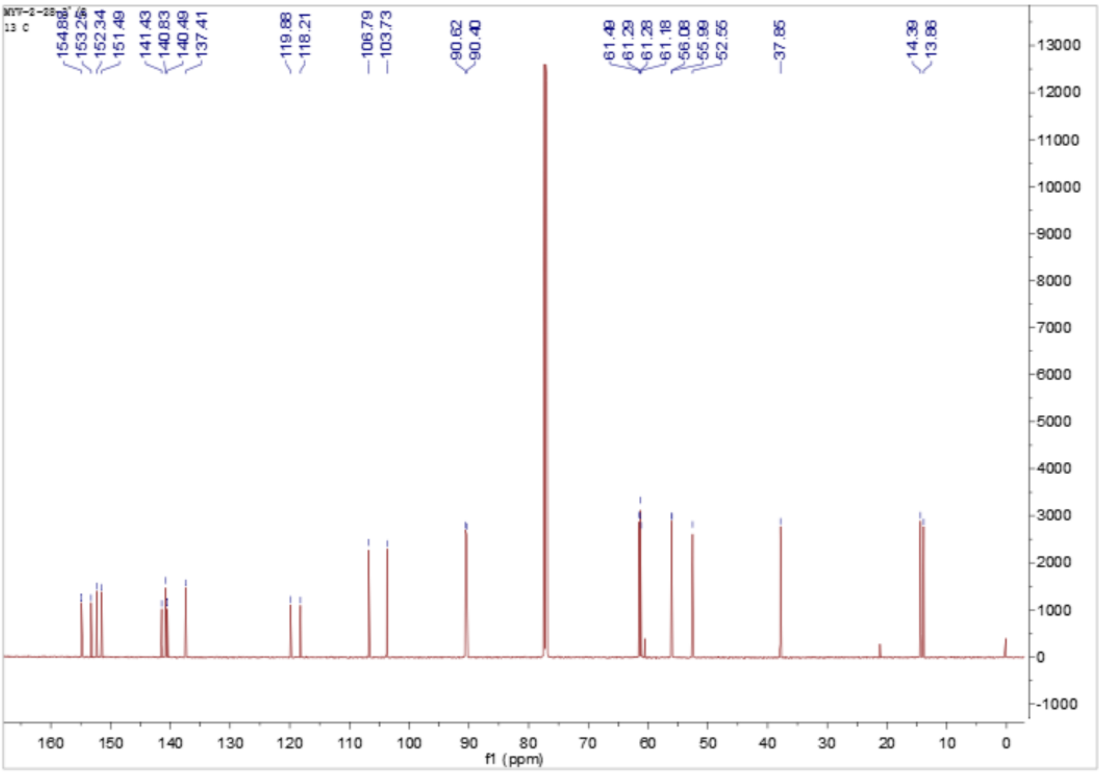

Figure S130. The ^{13}C NMR spectrum of 26 in CD_3OD

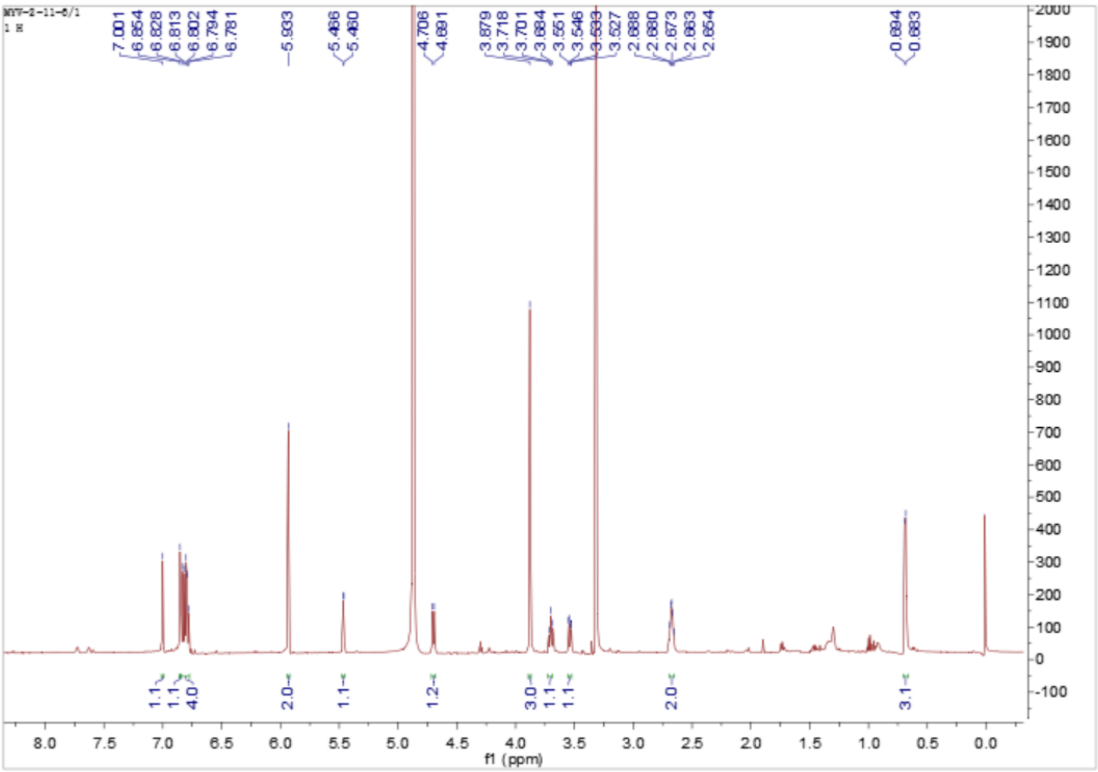

Figure S131. The ^{1}H NMR spectrum of 27 in CD_3OD

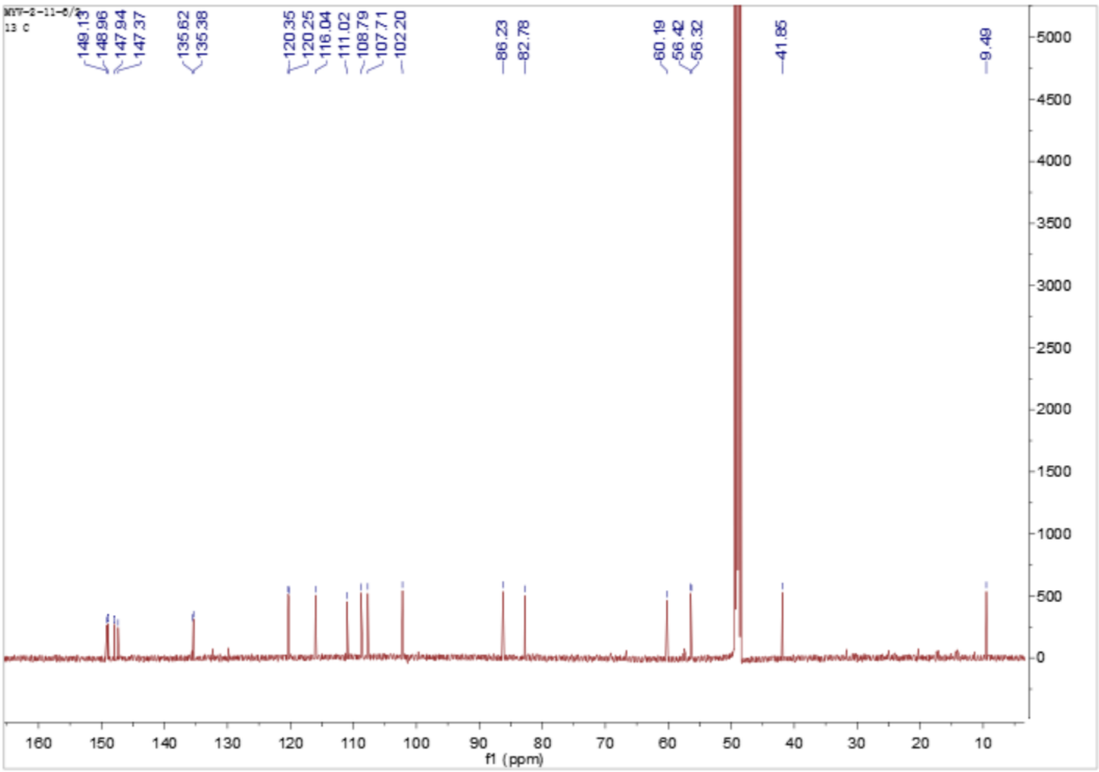

Figure S132. The ^{13}C NMR spectrum of 27 in CD_3OD

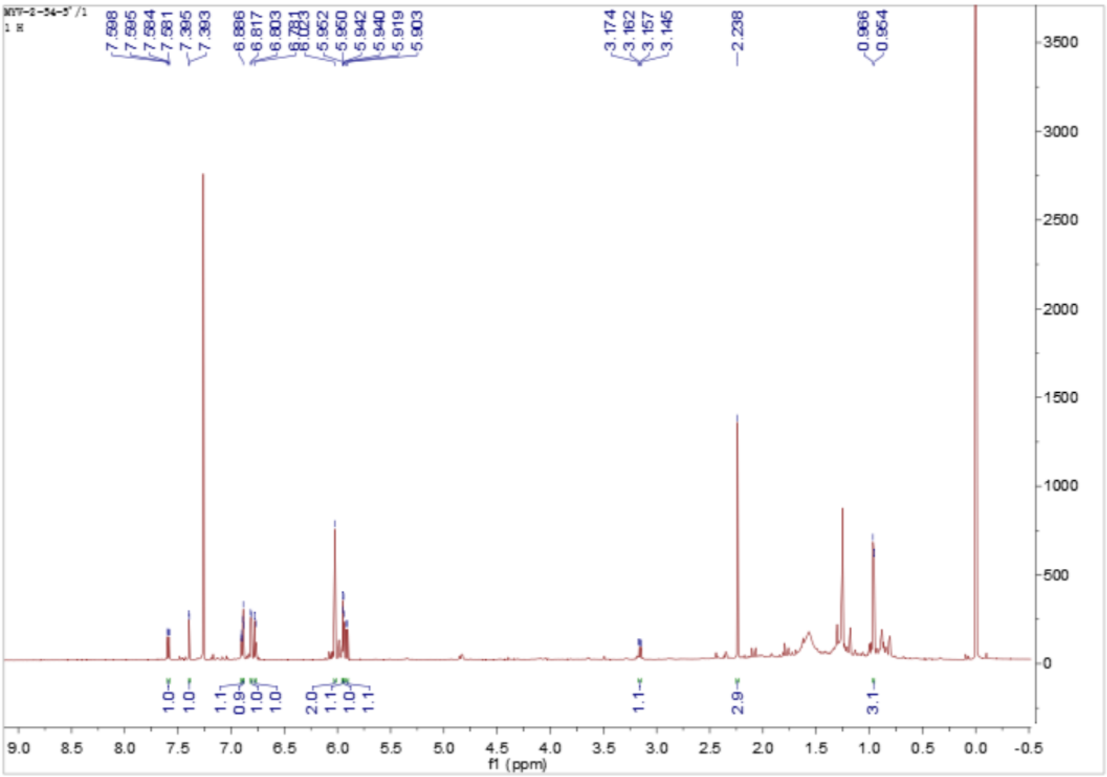

Figure S133. The ^{1}H NMR spectrum of 28 in $CDCl_3$

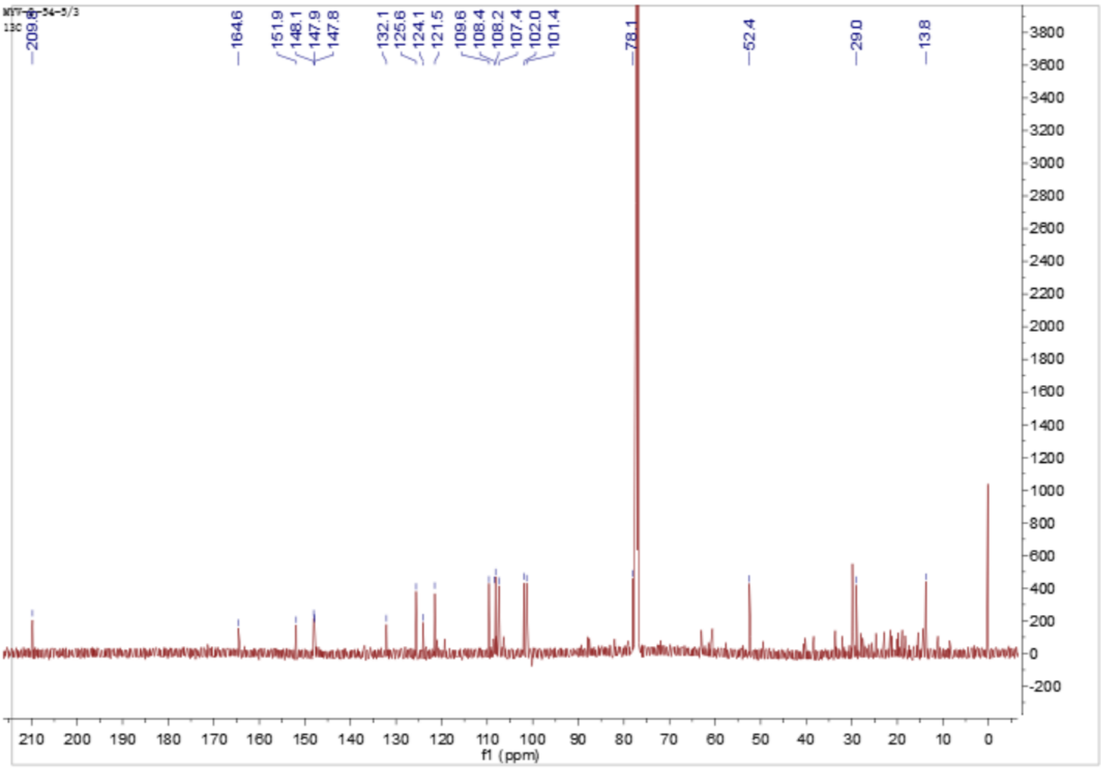

Figure S134. The ^{13}C NMR spectrum of 28 in $CDCl_3$

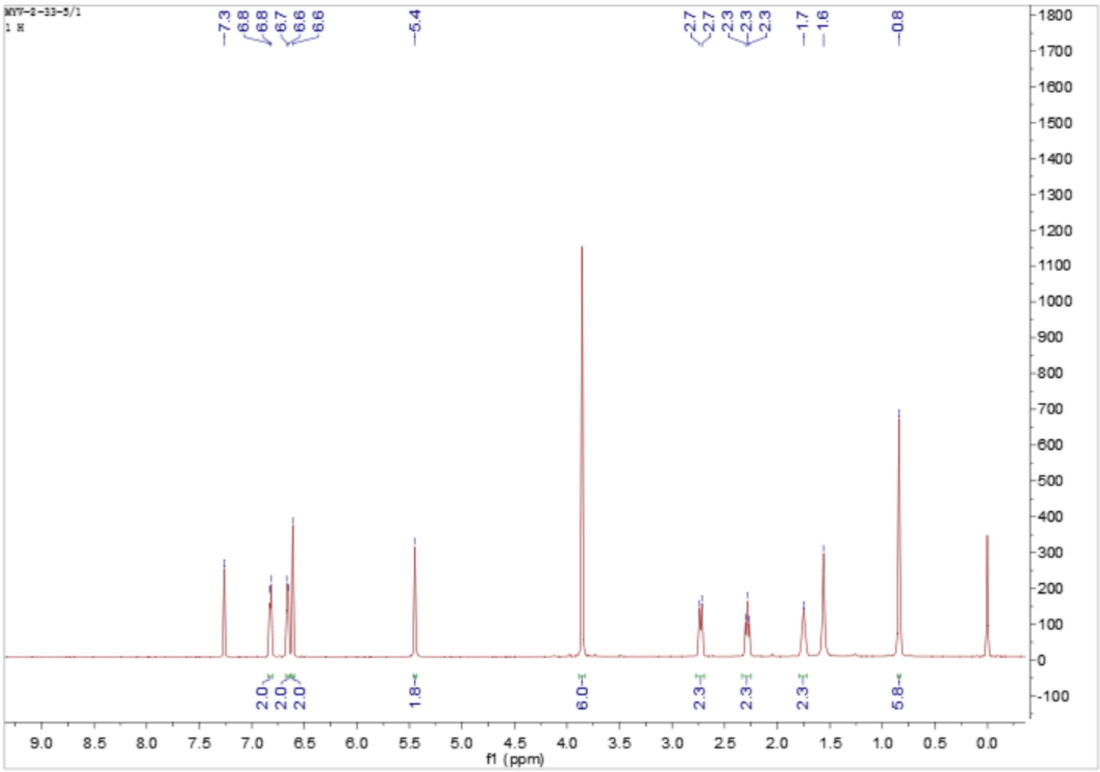

Figure S135. The ^{1}H NMR spectrum of 29 in $CDCl_3$

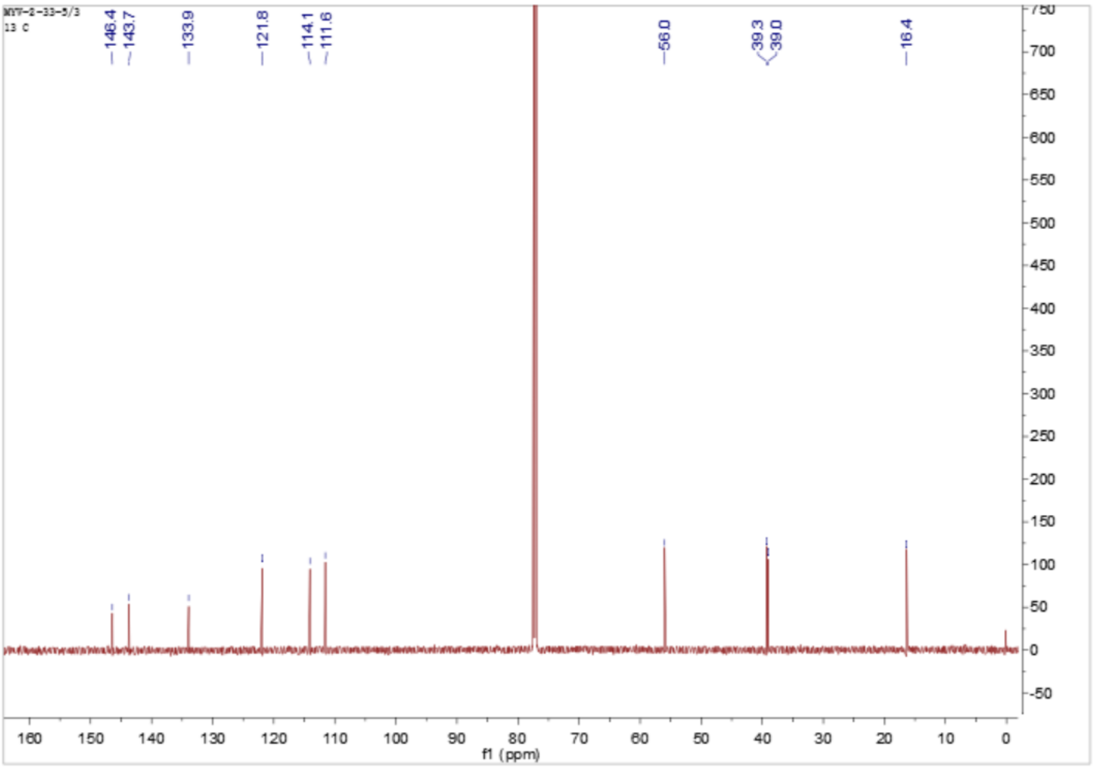

Figure S136. The ^{13}C NMR spectrum of 29 in $CDCl_3$

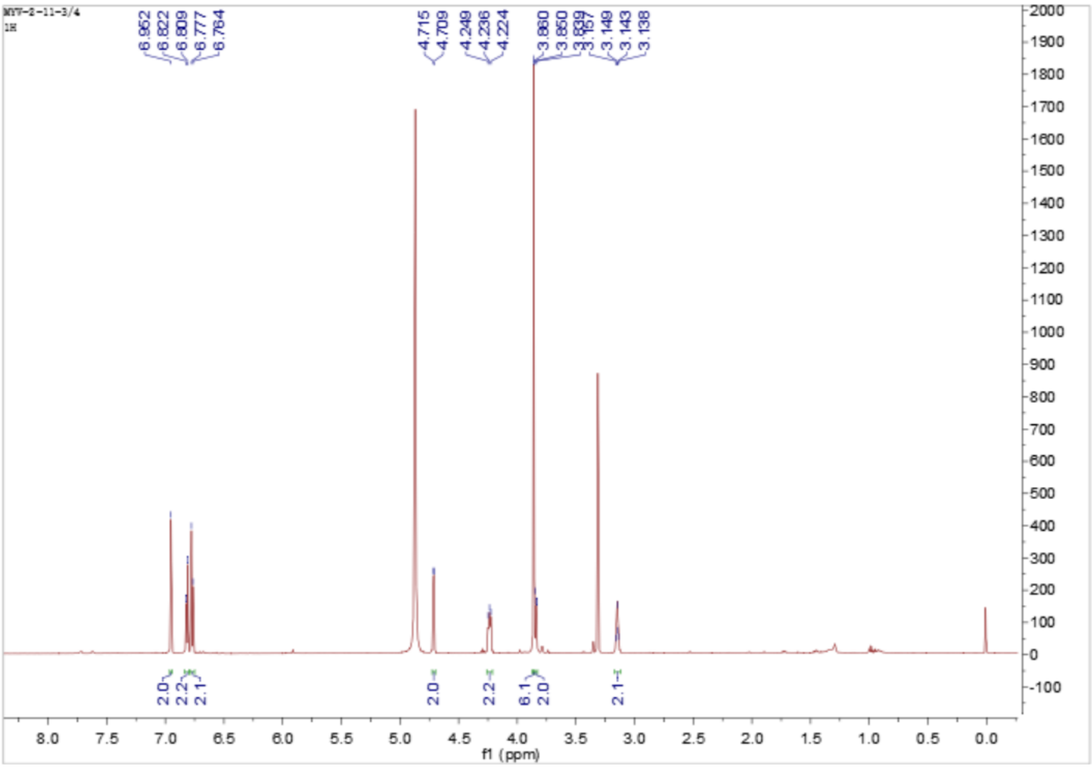

Figure S137. The ^{1}H NMR spectrum of 30 in CD_3OD

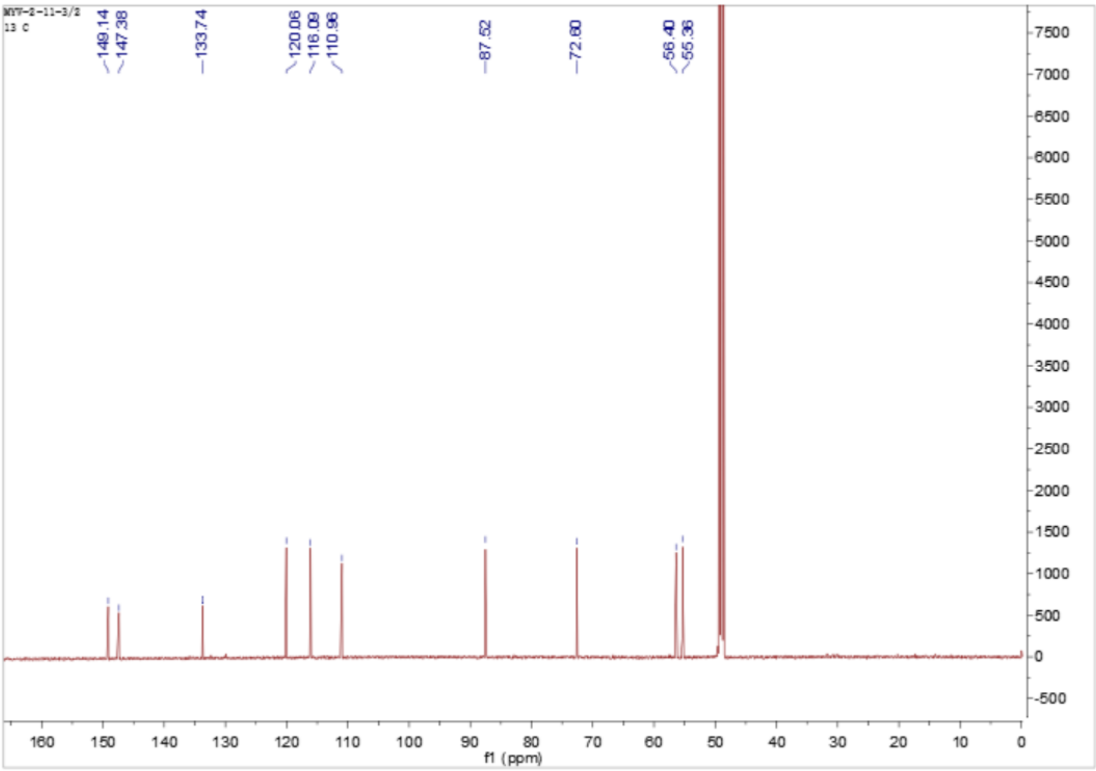

Figure S138. The ^{13}C NMR spectrum of 30 in CD_3OD

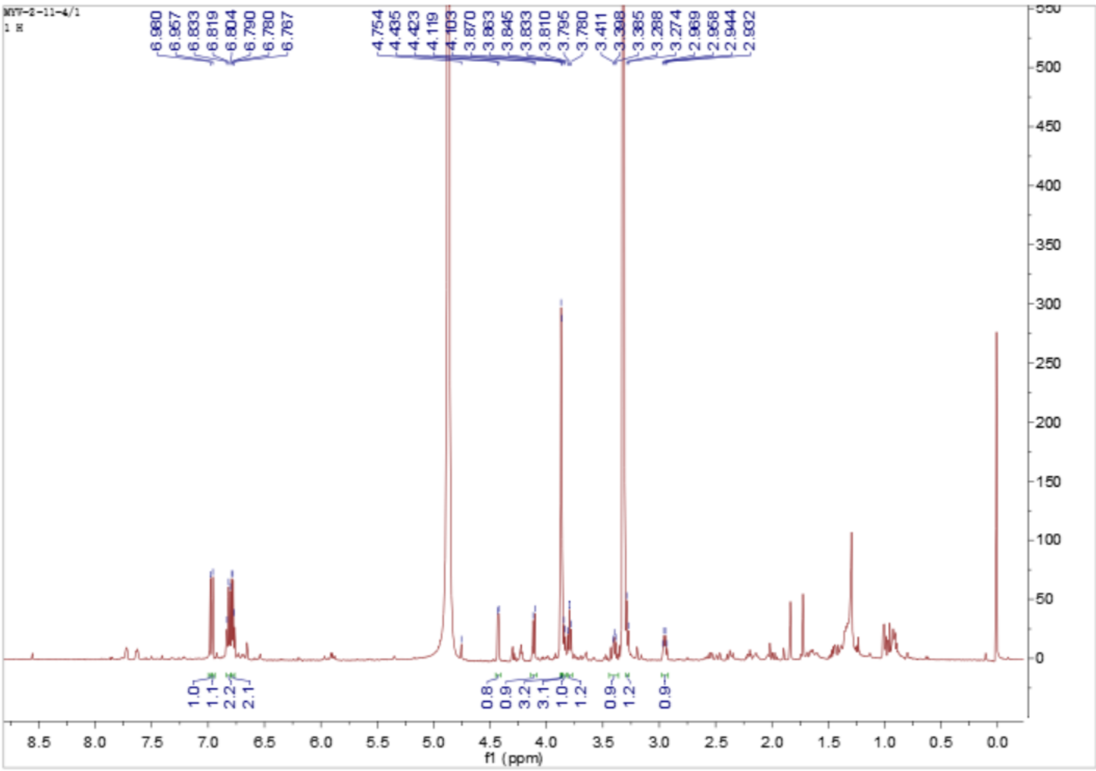

Figure S139. The ^{1}H NMR spectrum of 31 in CD_3OD

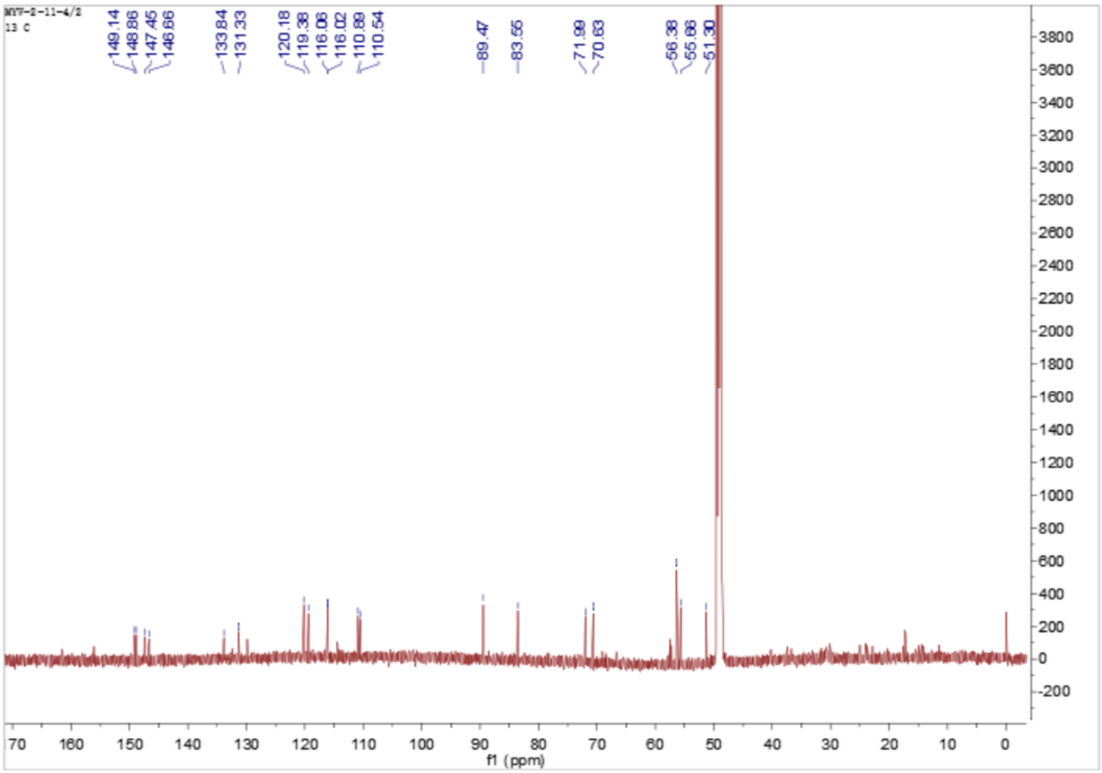

Figure S140. The ^{13}C NMR spectrum of 31 in CD_3OD

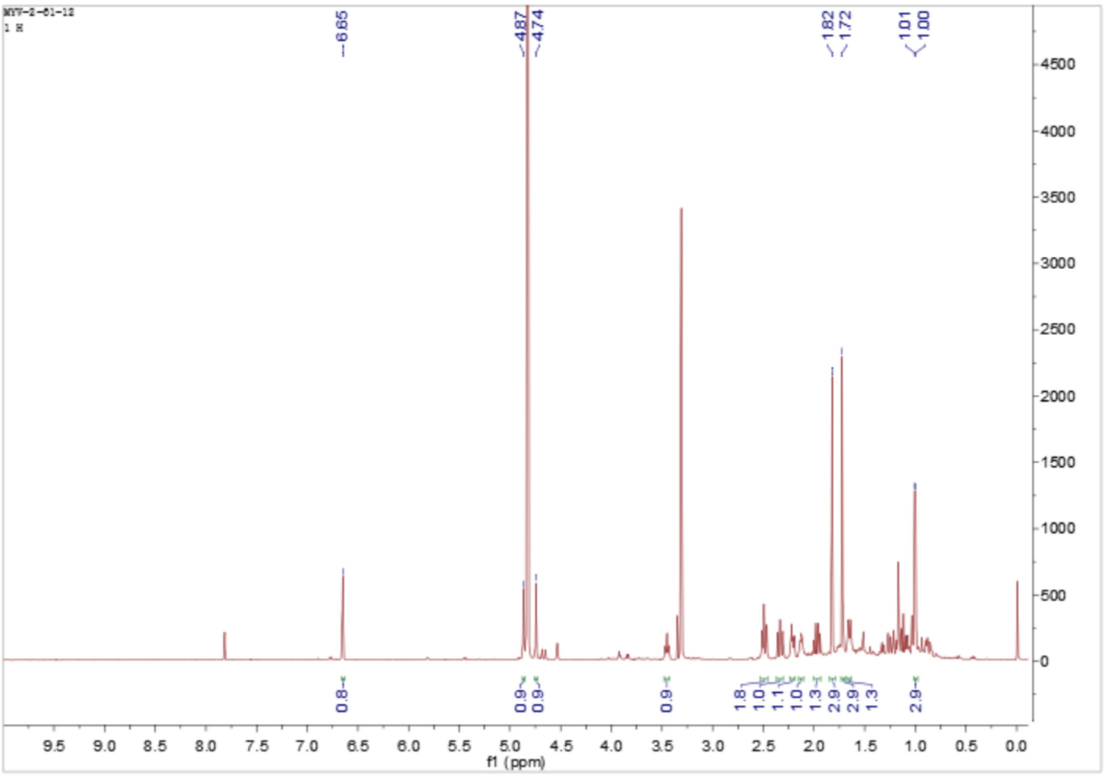

Figure S141. The ^{1}H NMR spectrum of 32 in CD_3OD

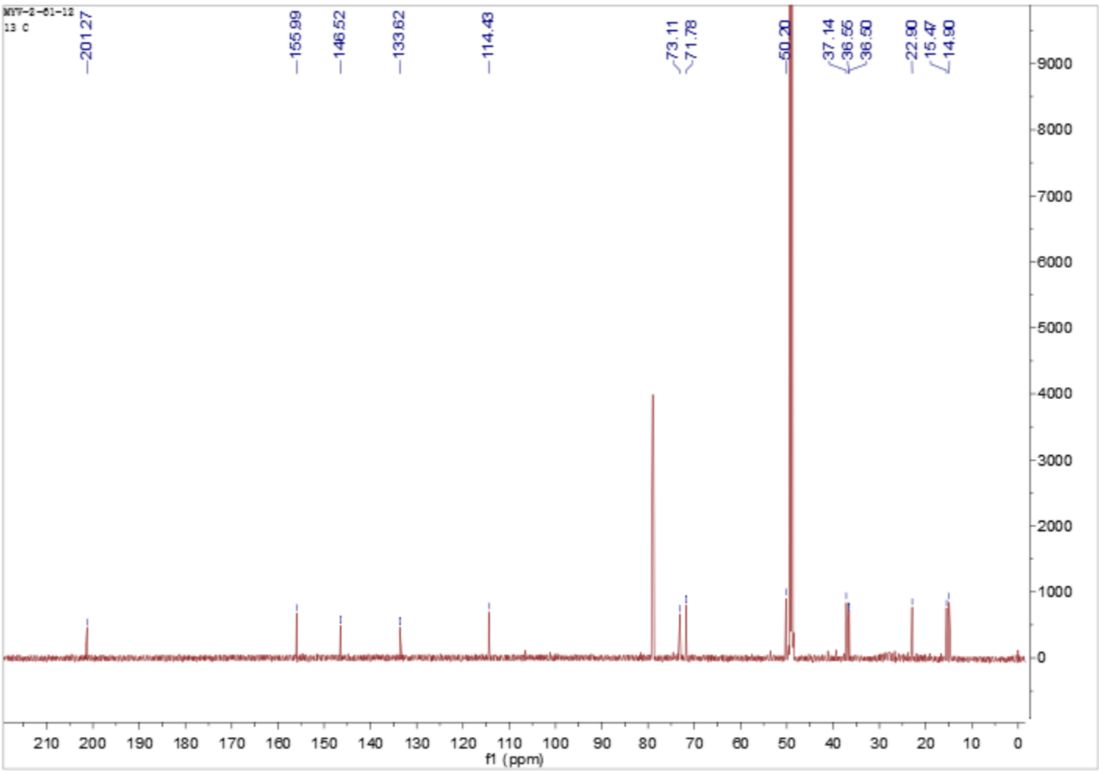

Figure S142. The ^{13}C NMR spectrum of 32 in CD_3OD

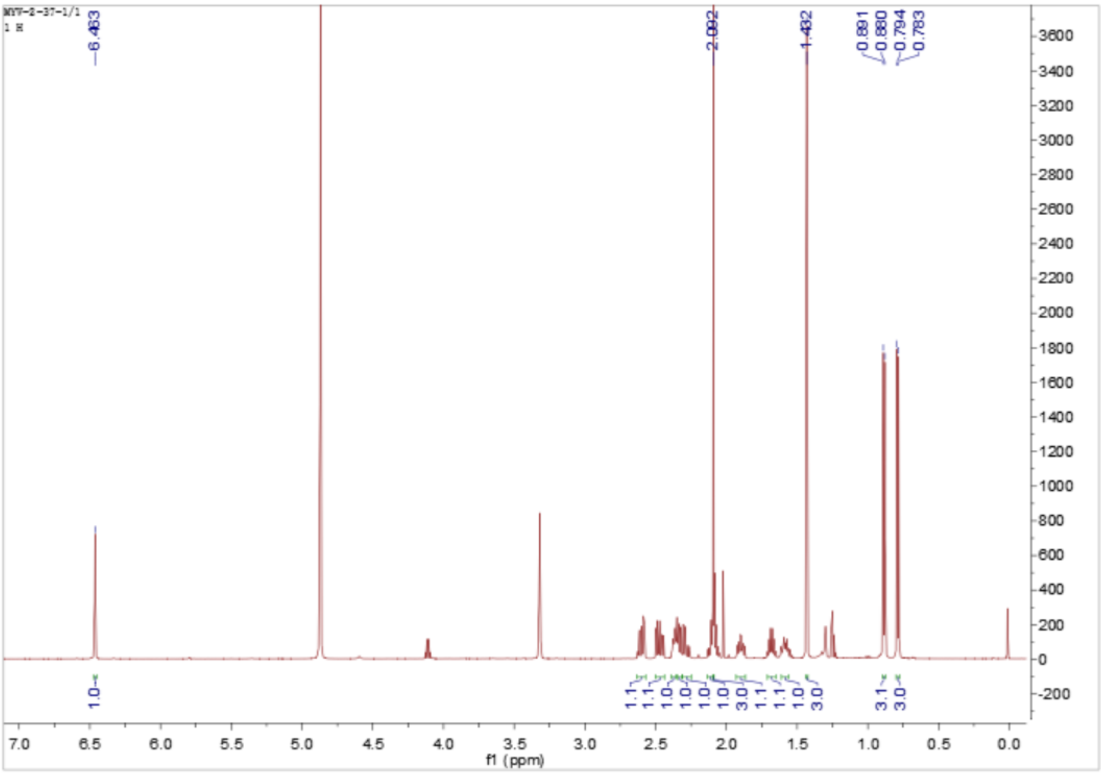

Figure S143. The ^{1}H NMR spectrum of 33 in CD_3OD

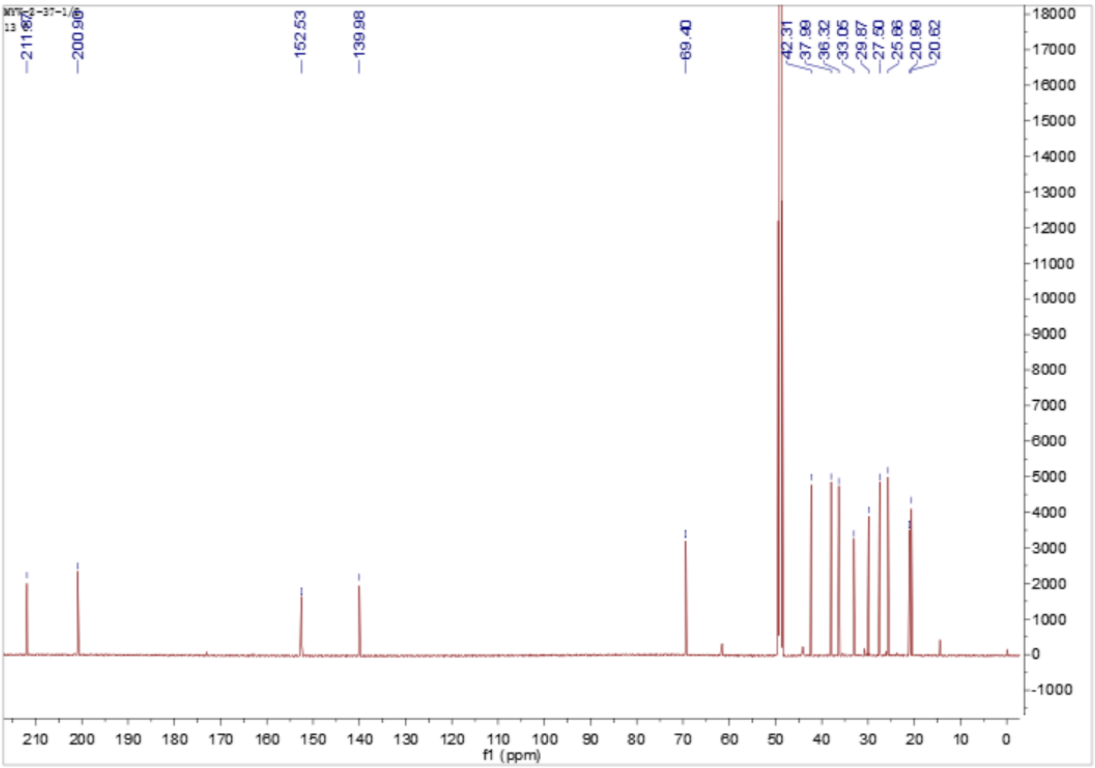

Figure S144. The ^{13}C NMR spectrum of 33 in CD_3OD

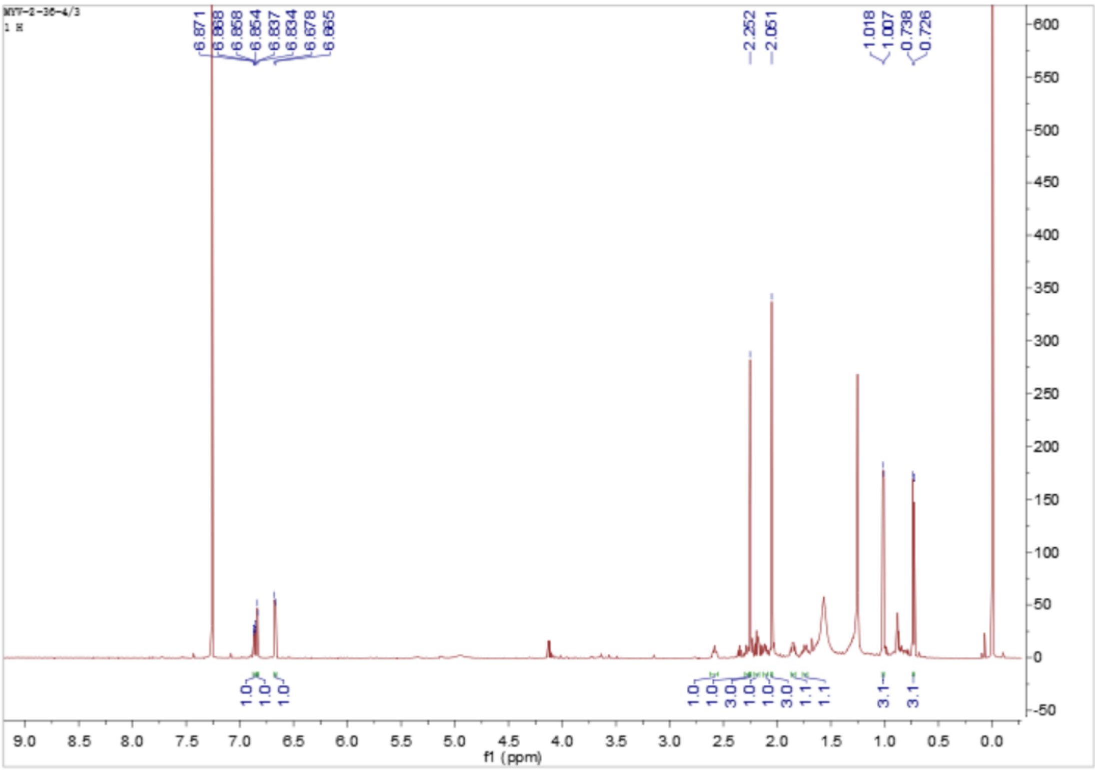

Figure S145. The ^{1}H NMR spectrum of 34 in CD_3OD

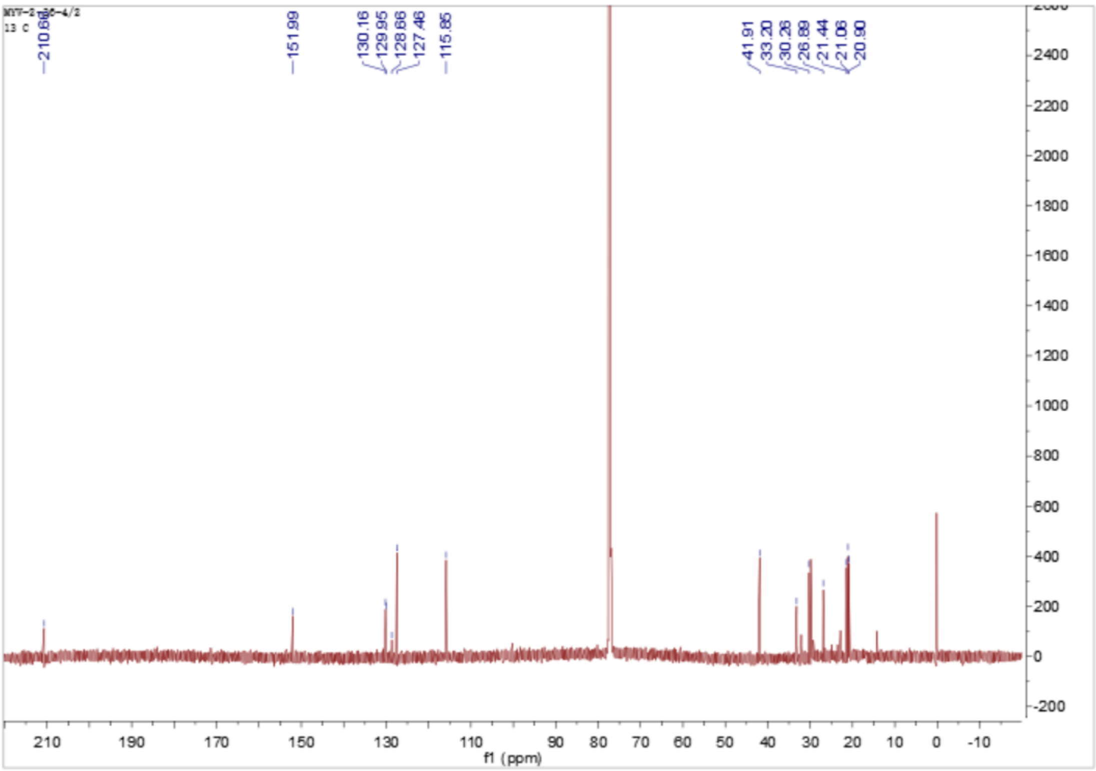

Figure S146. The ^{13}C NMR spectrum of 34 in CD_3OD

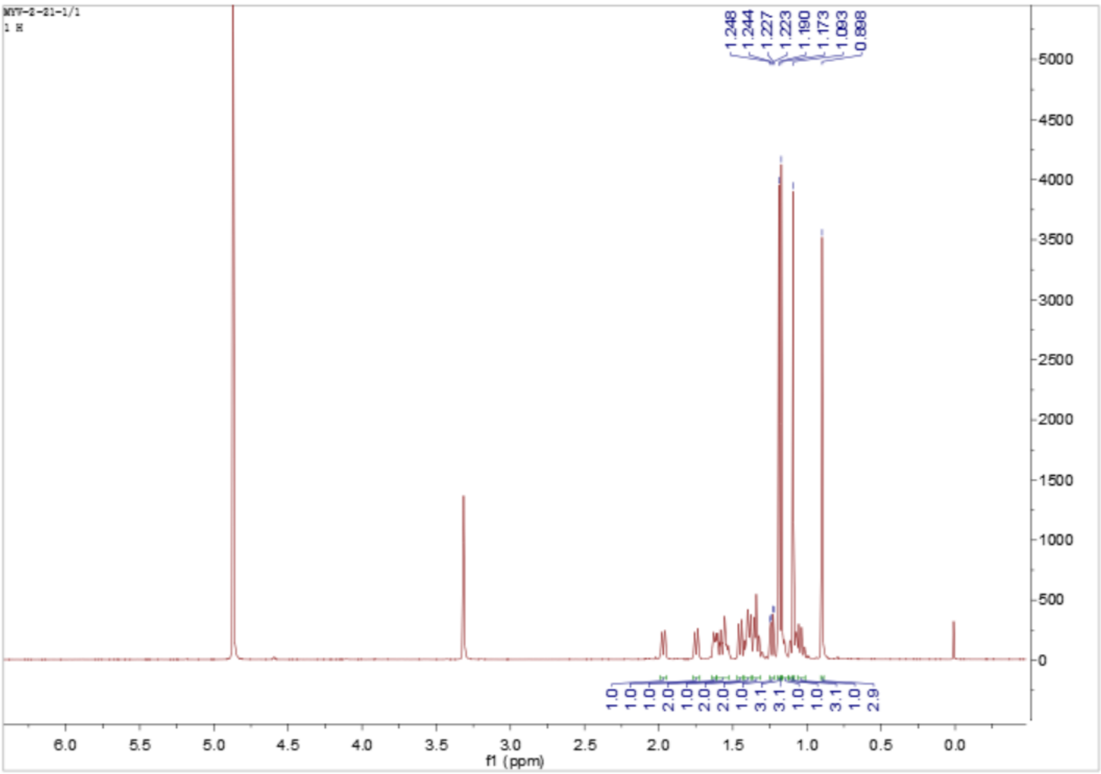

Figure S147. The ^{1}H NMR spectrum of 35 in CD_3OD

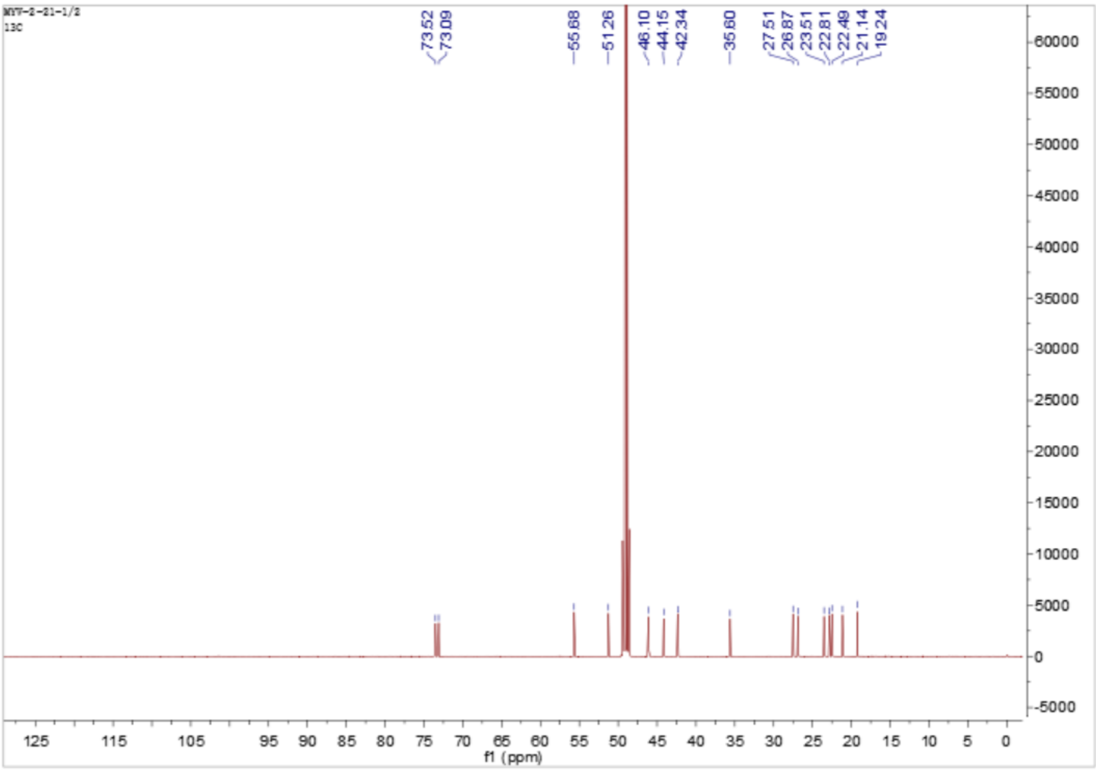

Figure S148. The ^{13}C NMR spectrum of 35 in CD_3OD

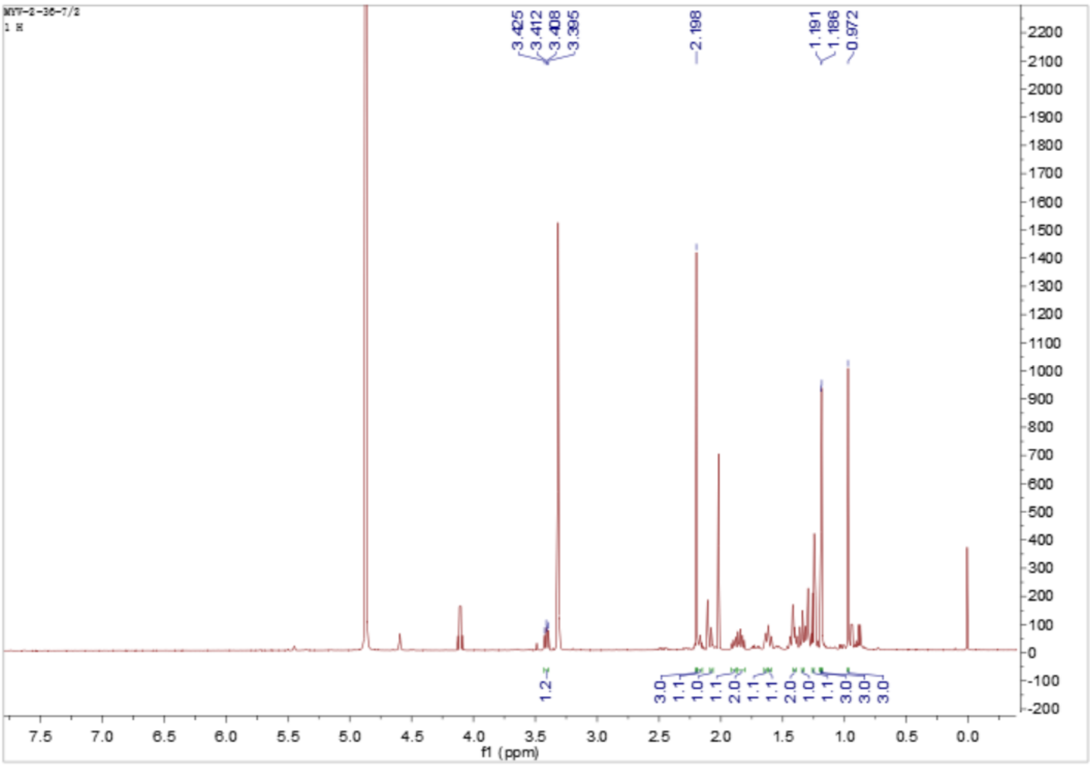

Figure S149. The ^{1}H NMR spectrum of 36 in CD_3OD

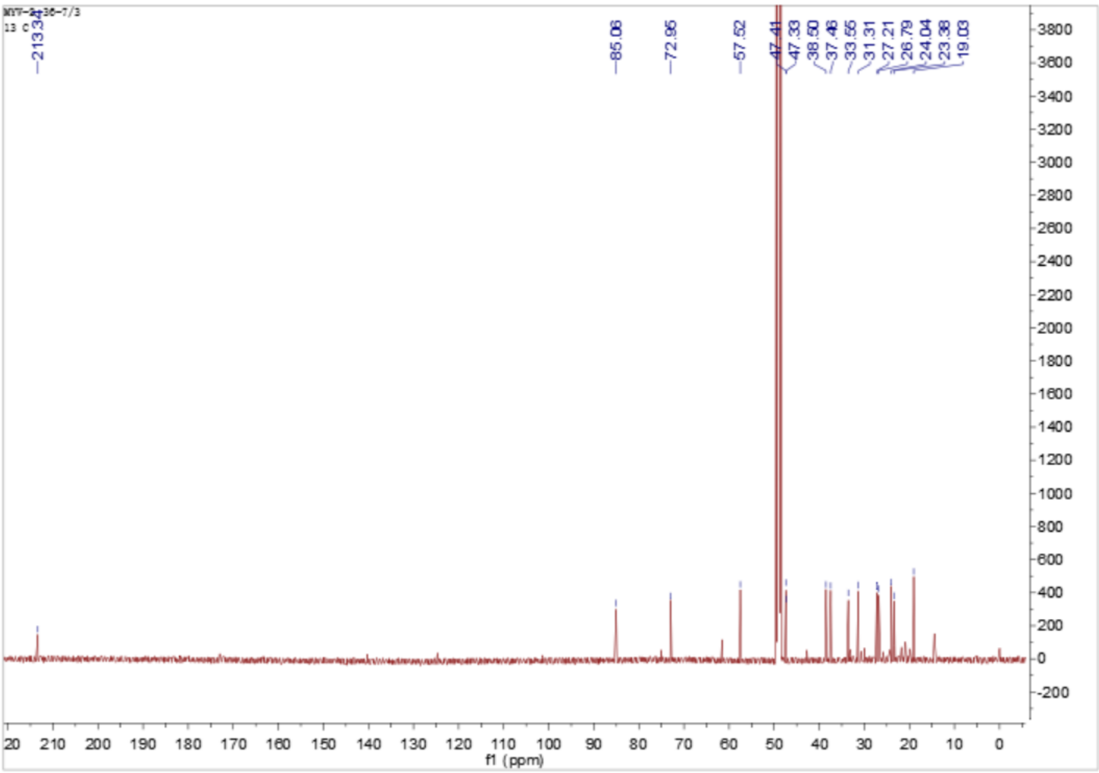

Figure S150. The ^{13}C NMR spectrum of 36 in CD_3OD

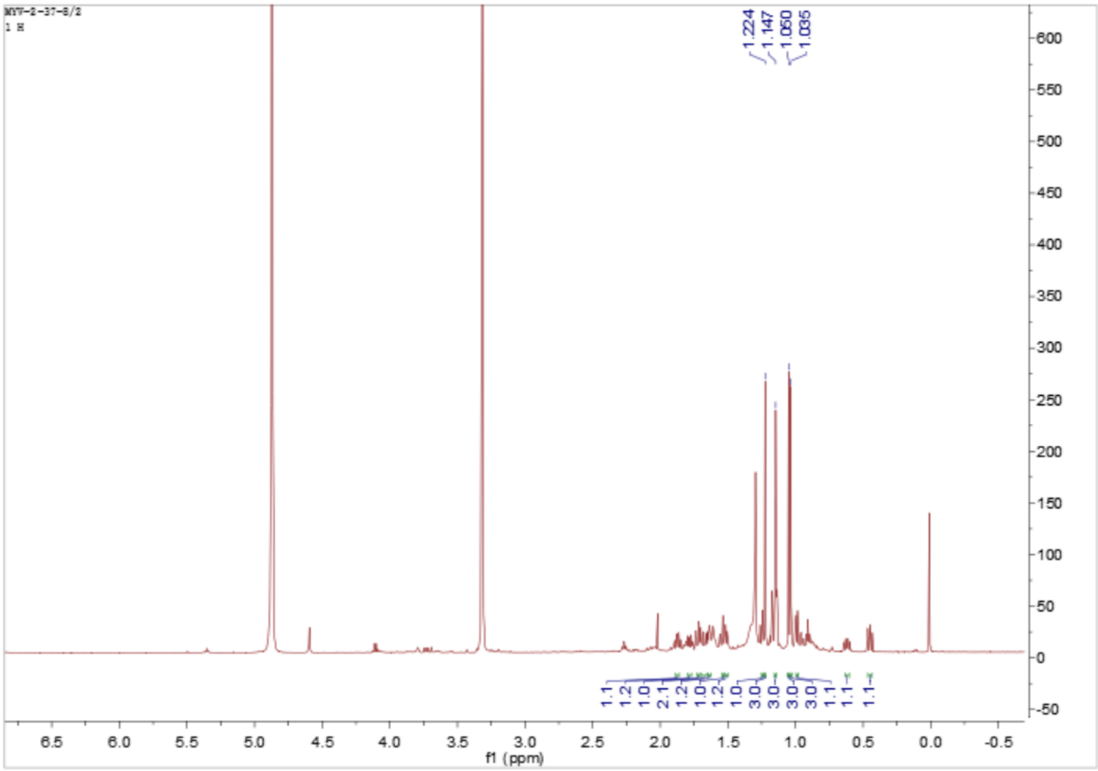

Figure S151. The ^{1}H NMR spectrum of 37 in CD_3OD

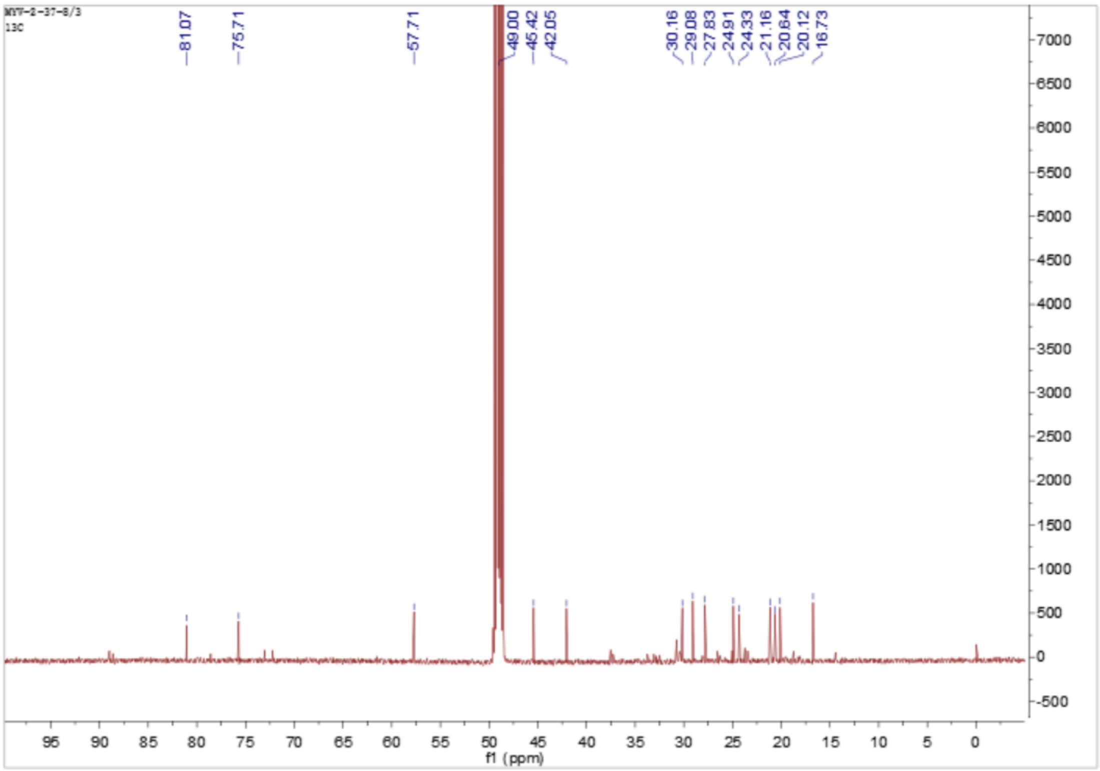

Figure S152. The ^{13}C NMR spectrum of 37 in CD_3OD

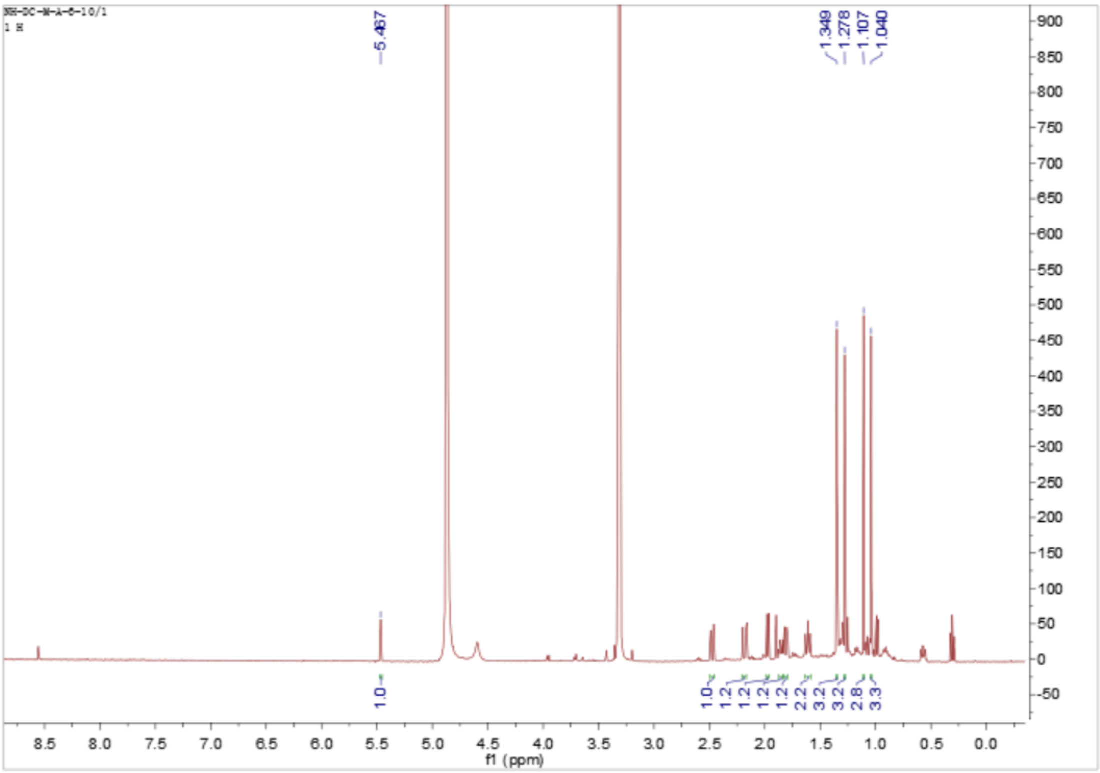

Figure S153. The ^{1}H NMR spectrum of 38 in CD_3OD

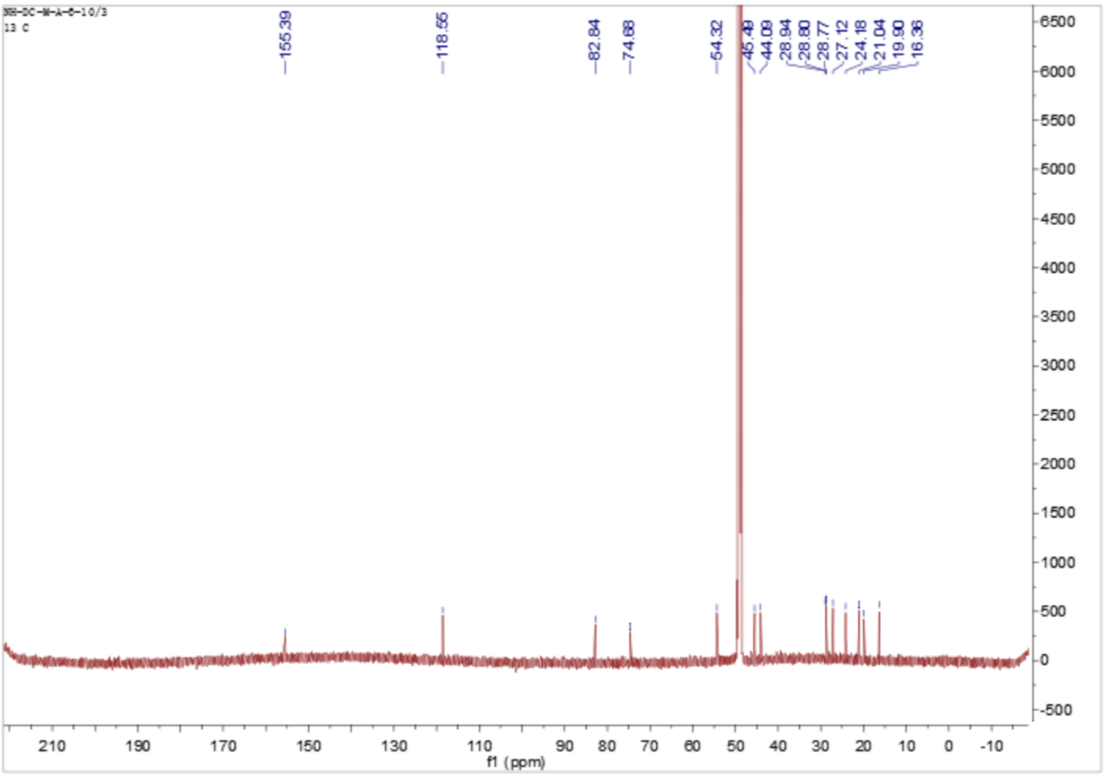

Figure S154. The ^{13}C NMR spectrum of 38 in CD_3OD

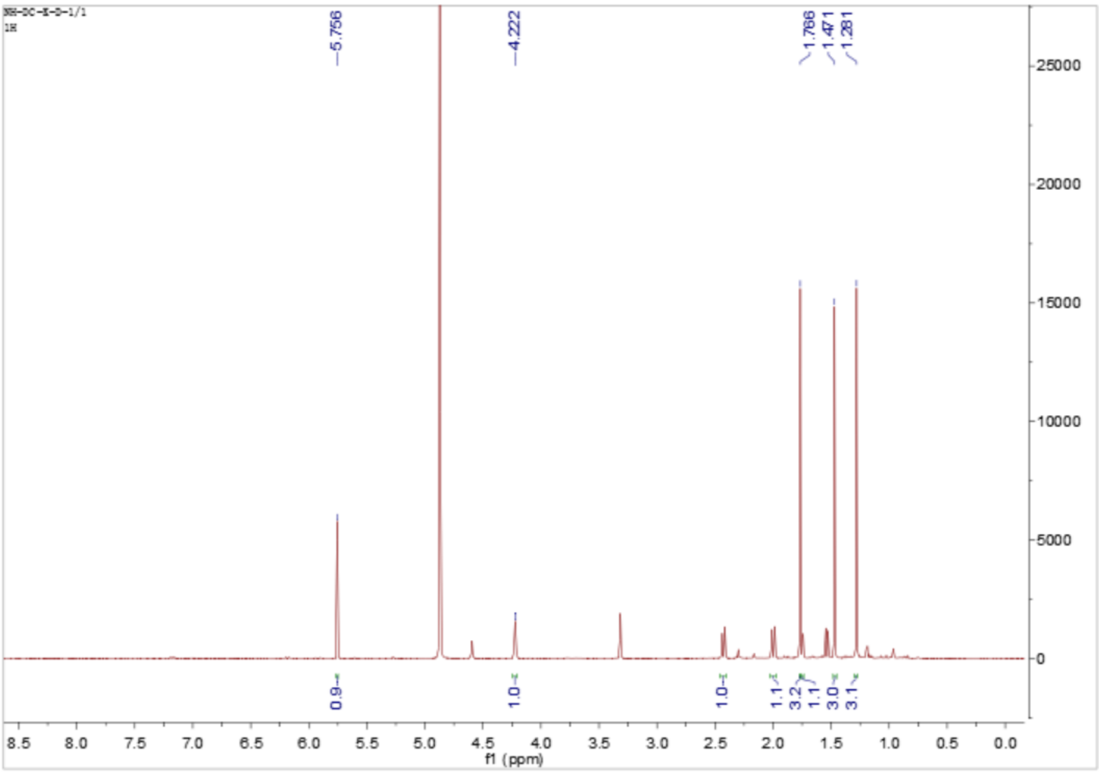

Figure S155. The ^{1}H NMR spectrum of 39 in CD_3OD

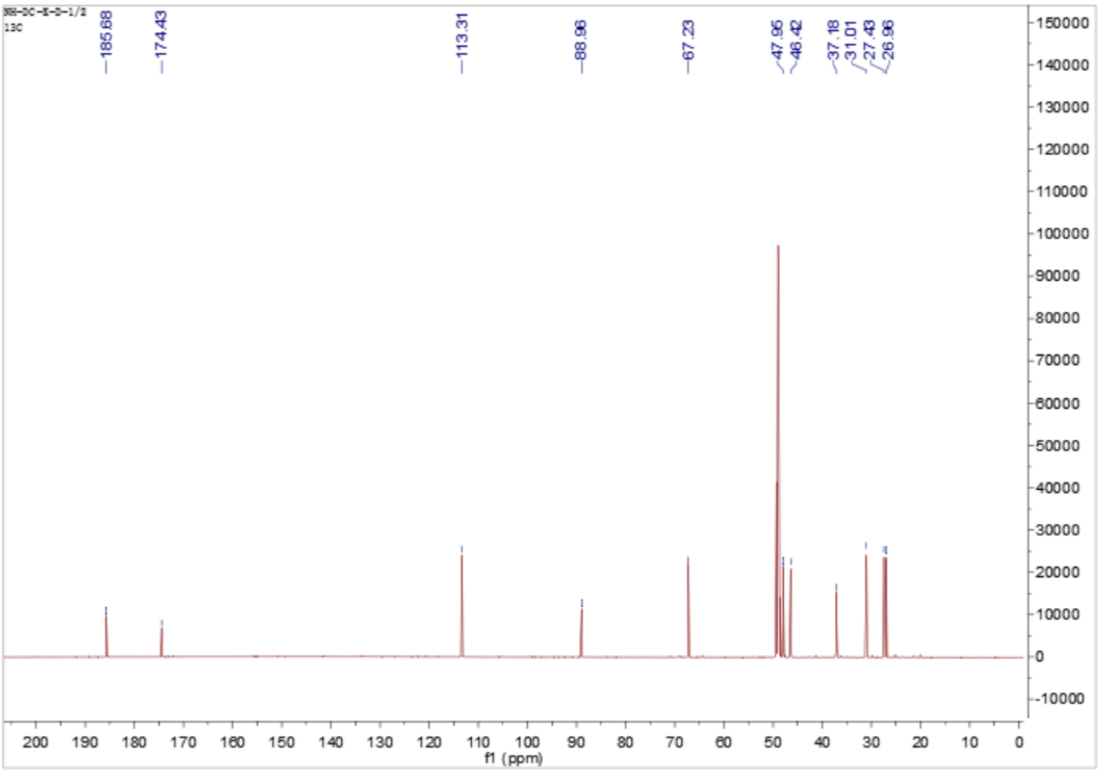

Figure S156. The ^{13}C NMR spectrum of 39 in CD_3OD

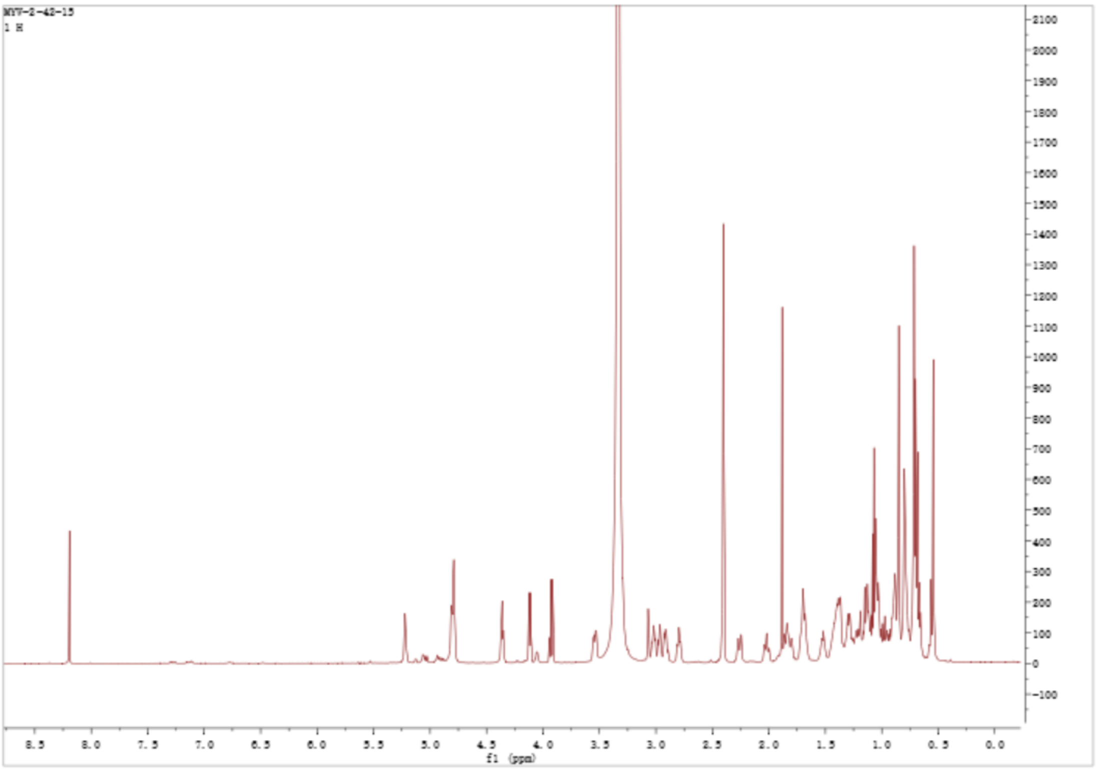

Figure S157. The ^{1}H NMR spectrum of 41 in DMSO-d_6

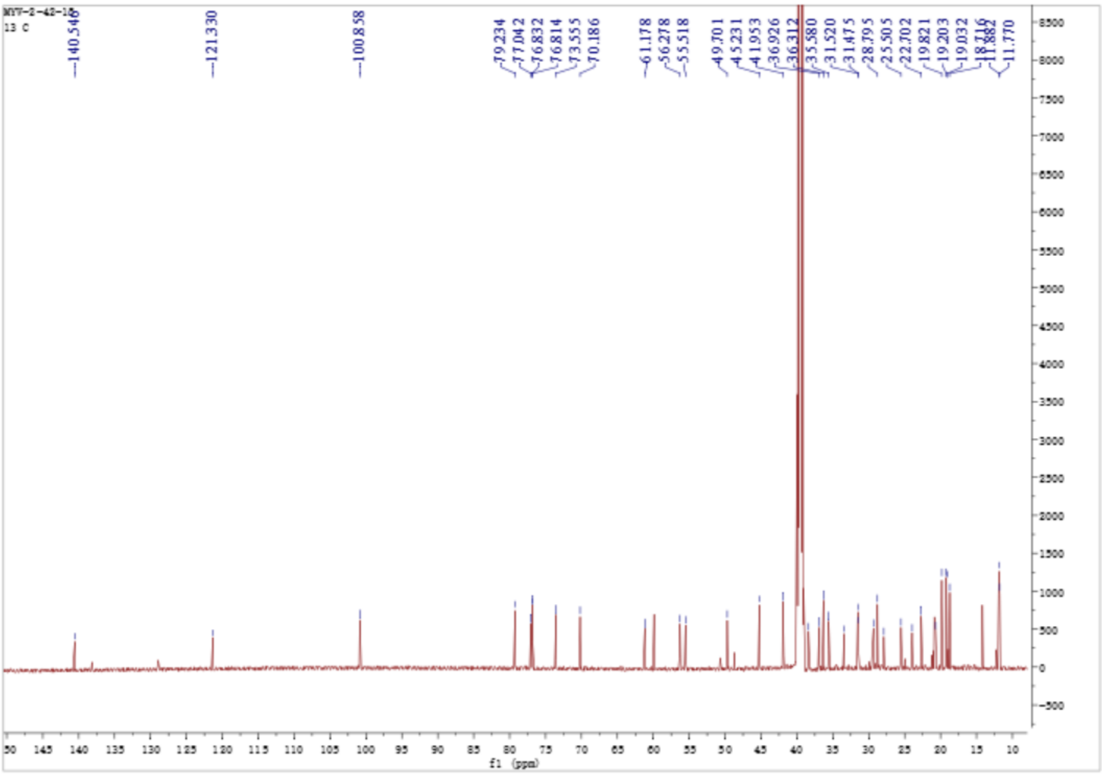

Figure S158. The ^{13}C NMR spectrum of 41 in DMSO-d_6

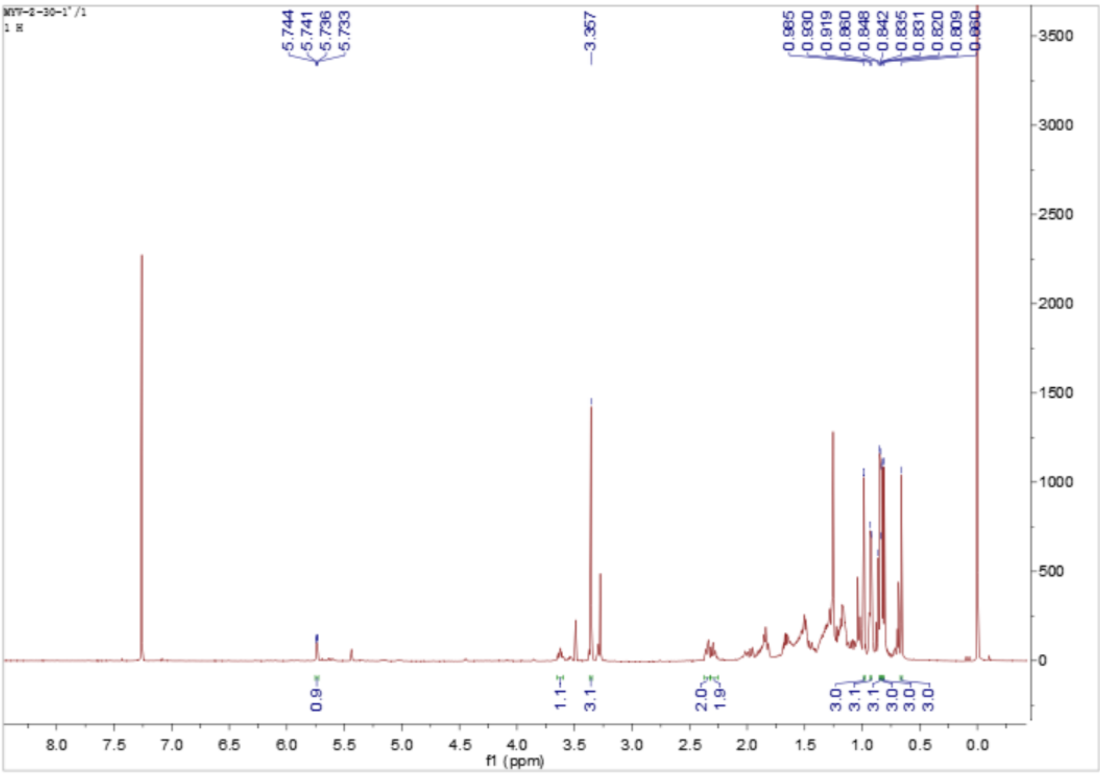

Figure S159. The ^{1}H NMR spectrum of 43 in $CDCl_3$

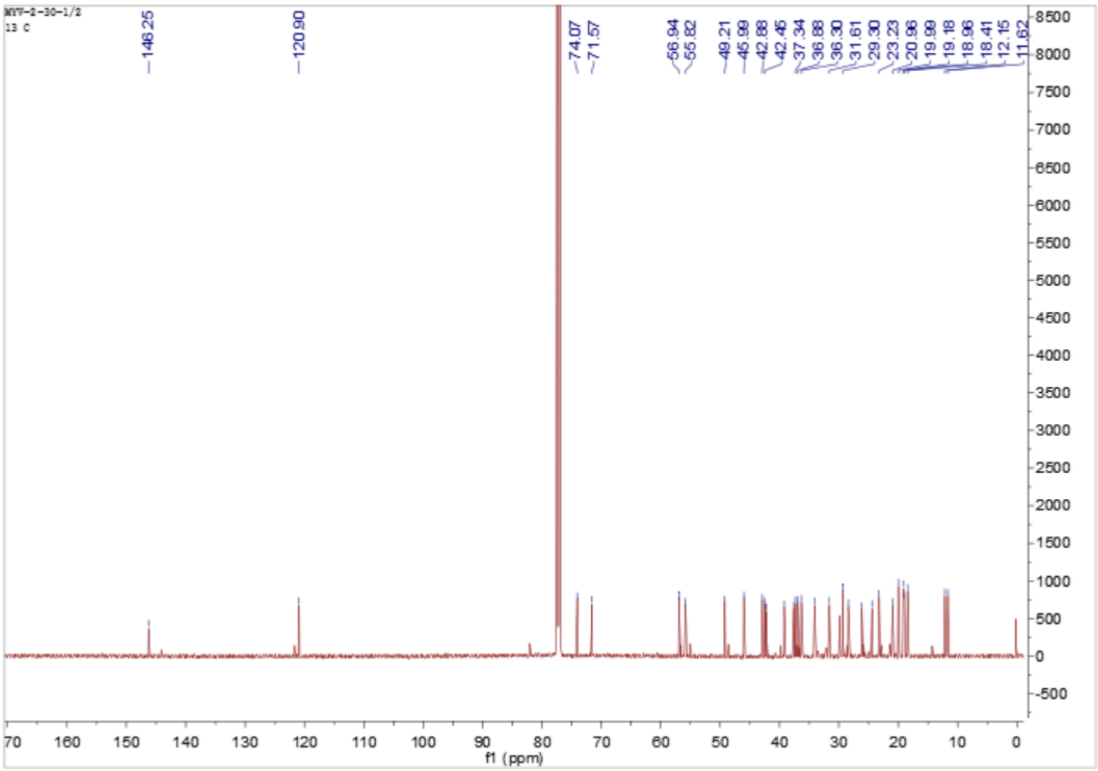

Figure S160. The ^{13}C NMR spectrum of 43 in $CDCl_3$

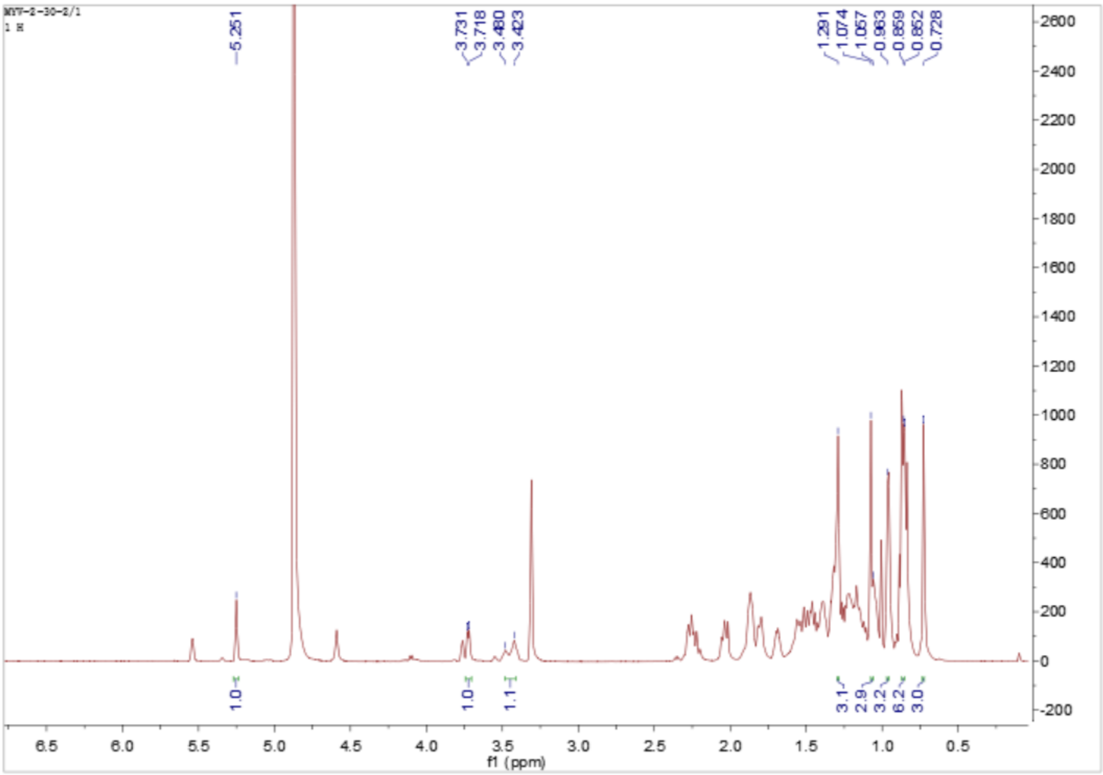

Figure S161. The ^{1}H NMR spectrum of 44 in CD_3OD

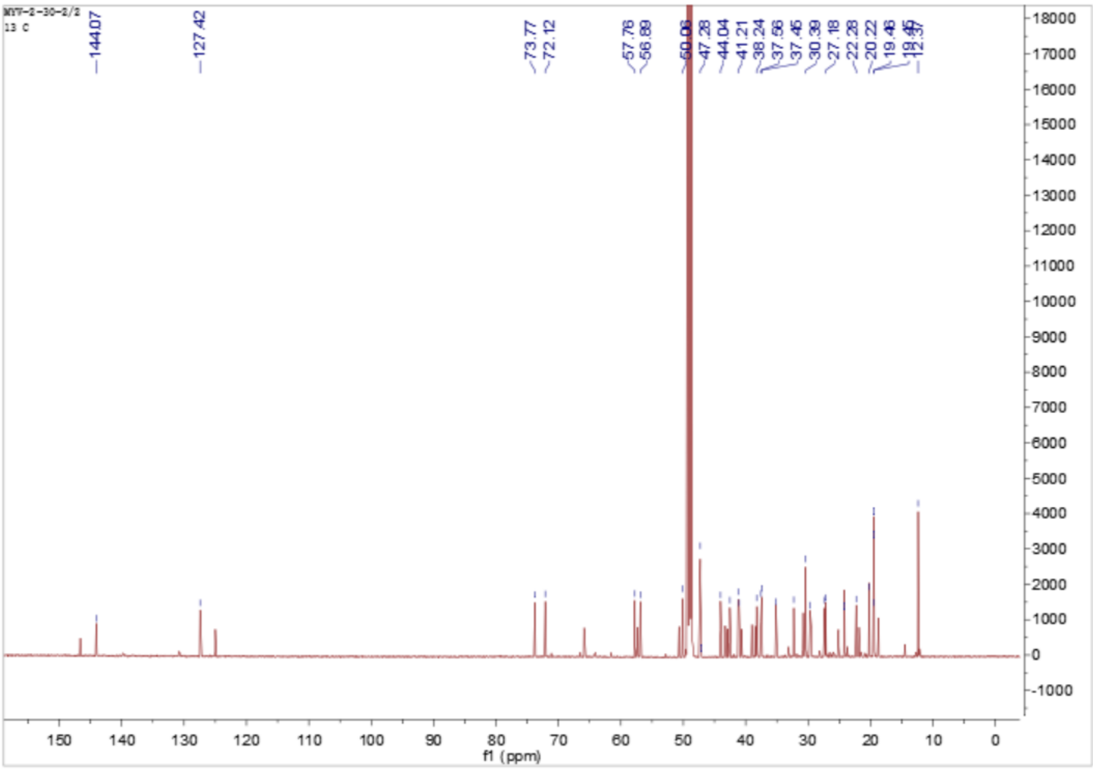

Figure S162. The ^{13}C NMR spectrum of 44 in CD_3OD

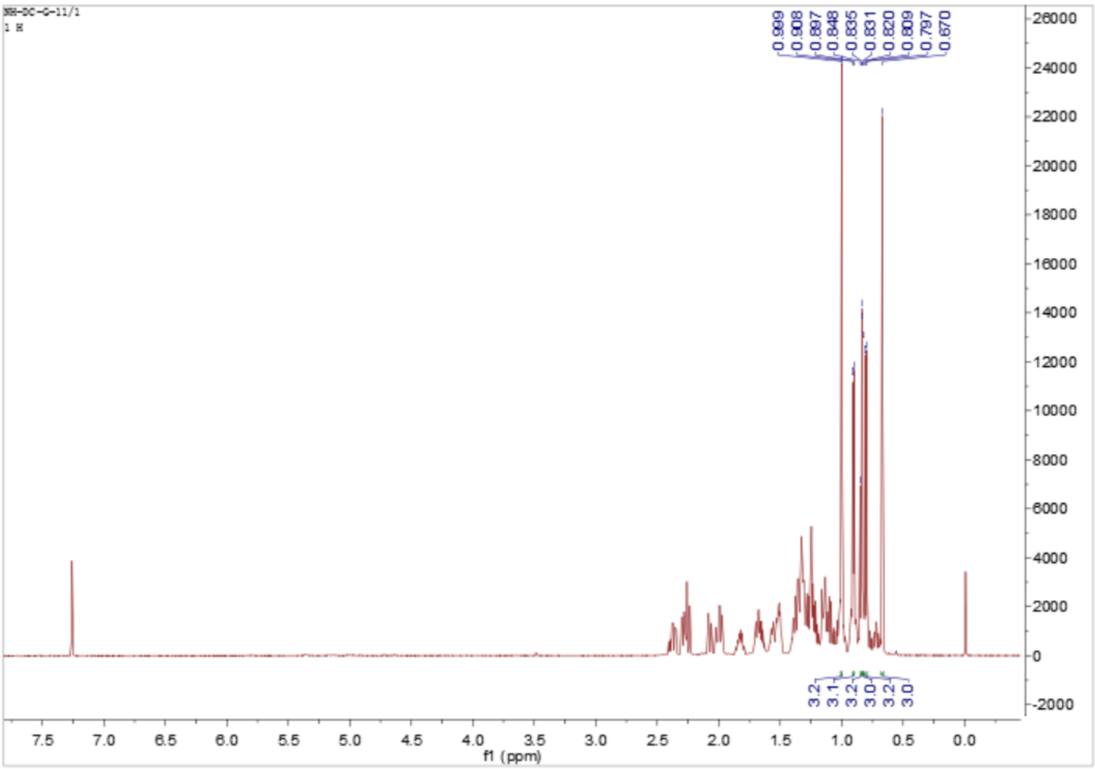

Figure S163. The ^{1}H NMR spectrum of 45 in CD_3OD

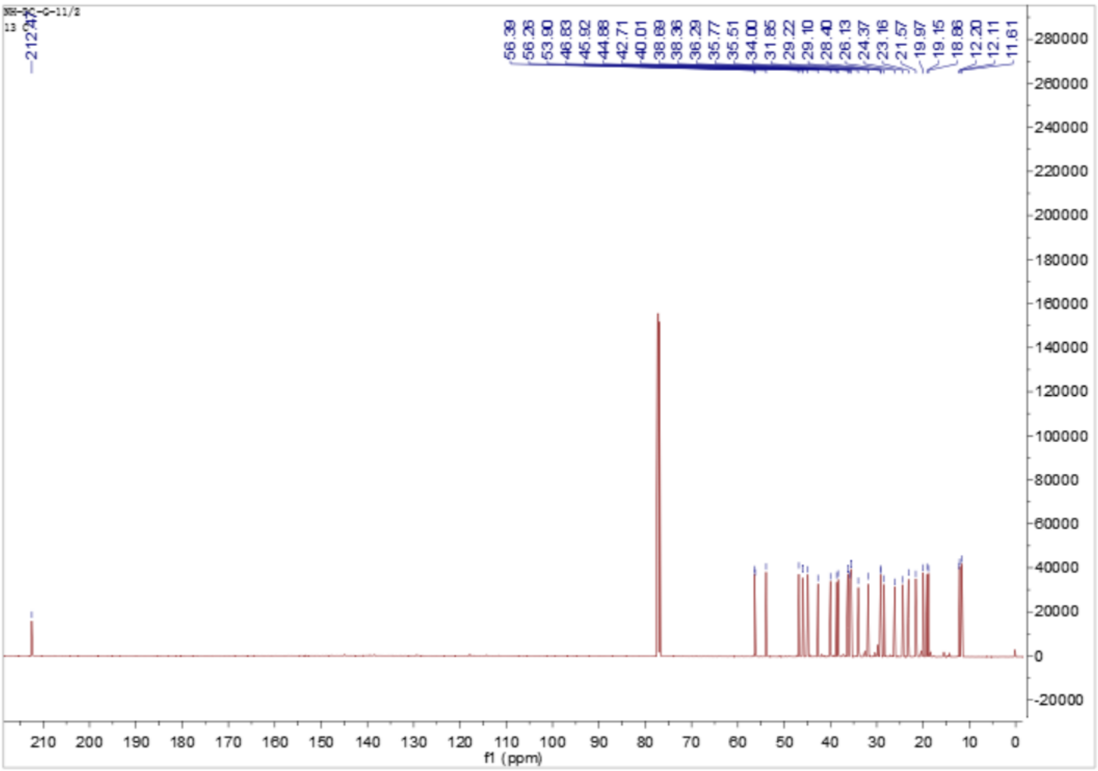

Figure S164. The ^{13}C NMR spectrum of 45 in CD_3OD

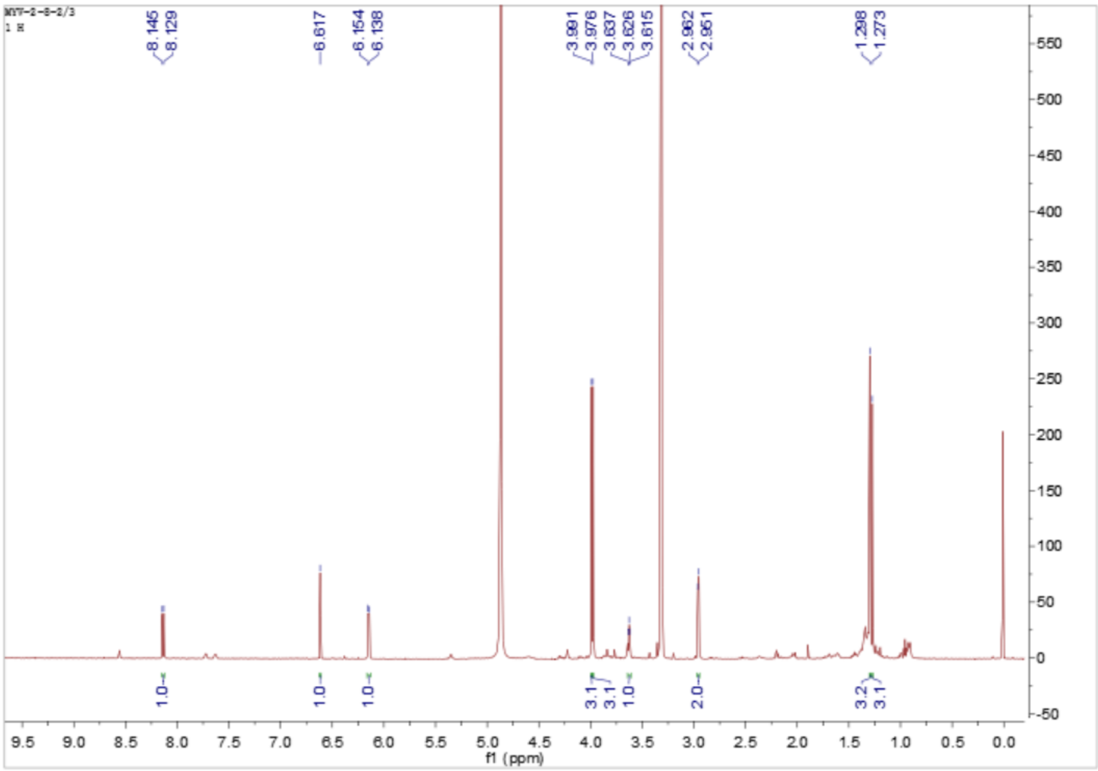

Figure S165. The ^{1}H NMR spectrum of 46 in CD_3OD

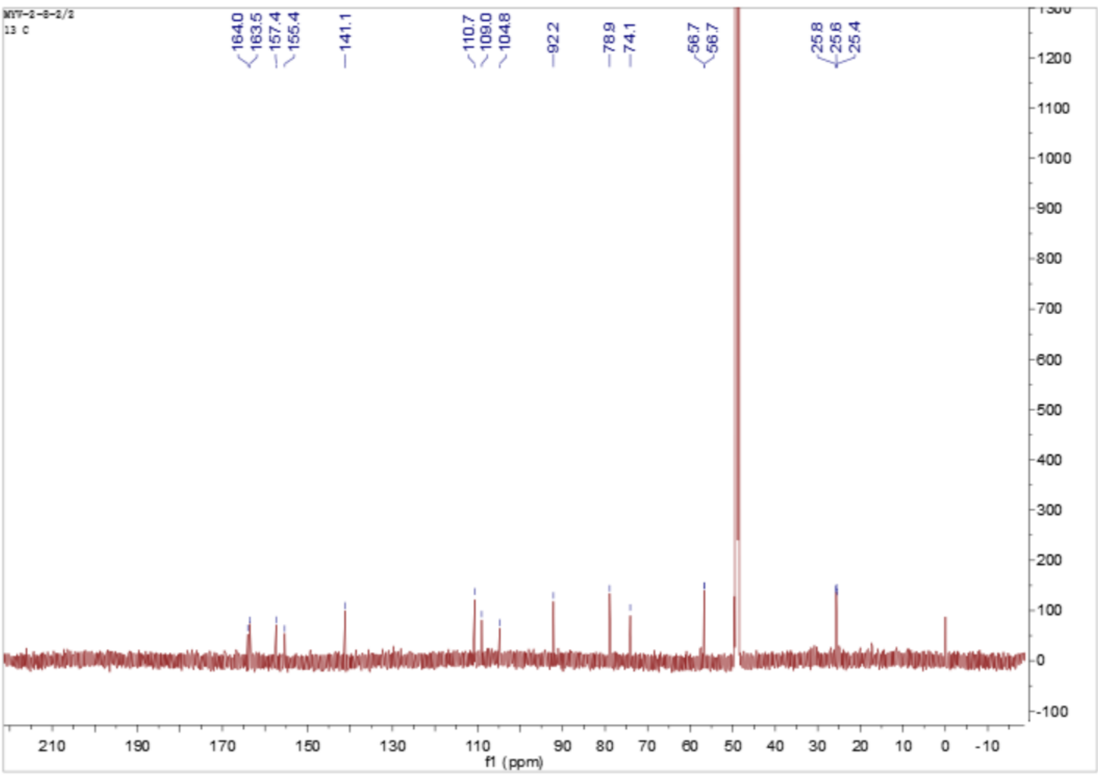

Figure S166. The ^{13}C NMR spectrum of 46 in CD_3OD

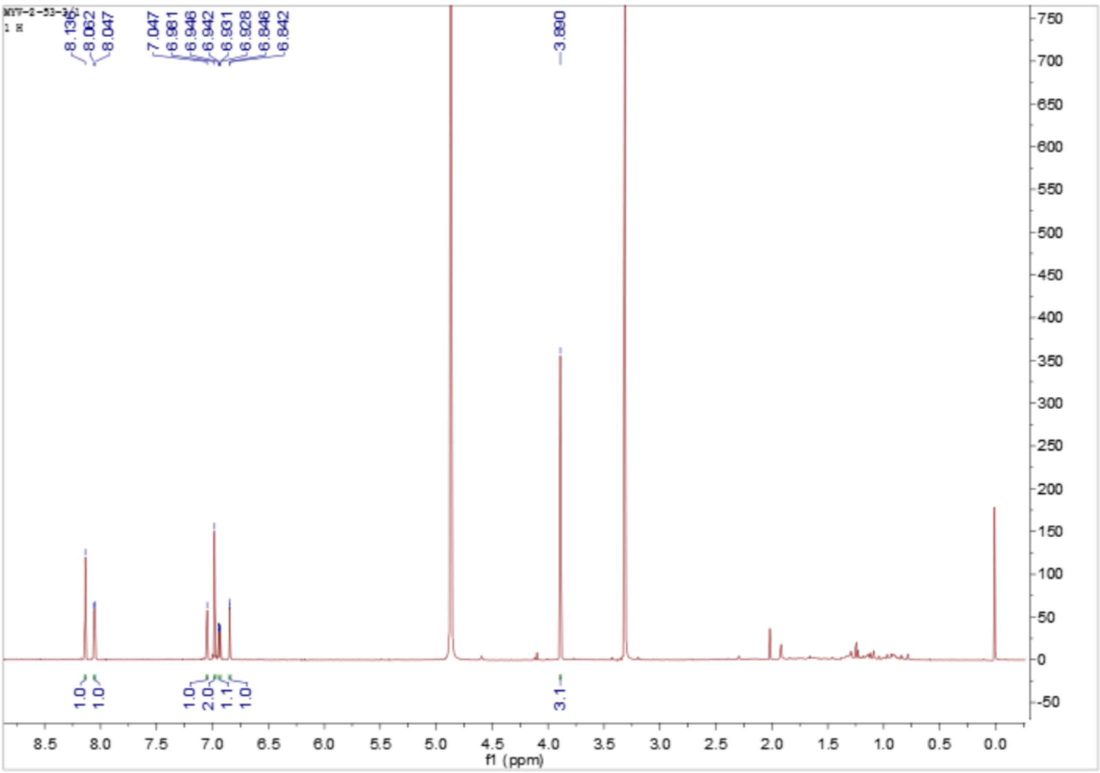

Figure S167. The ^{1}H NMR spectrum of 47 in CD_3OD

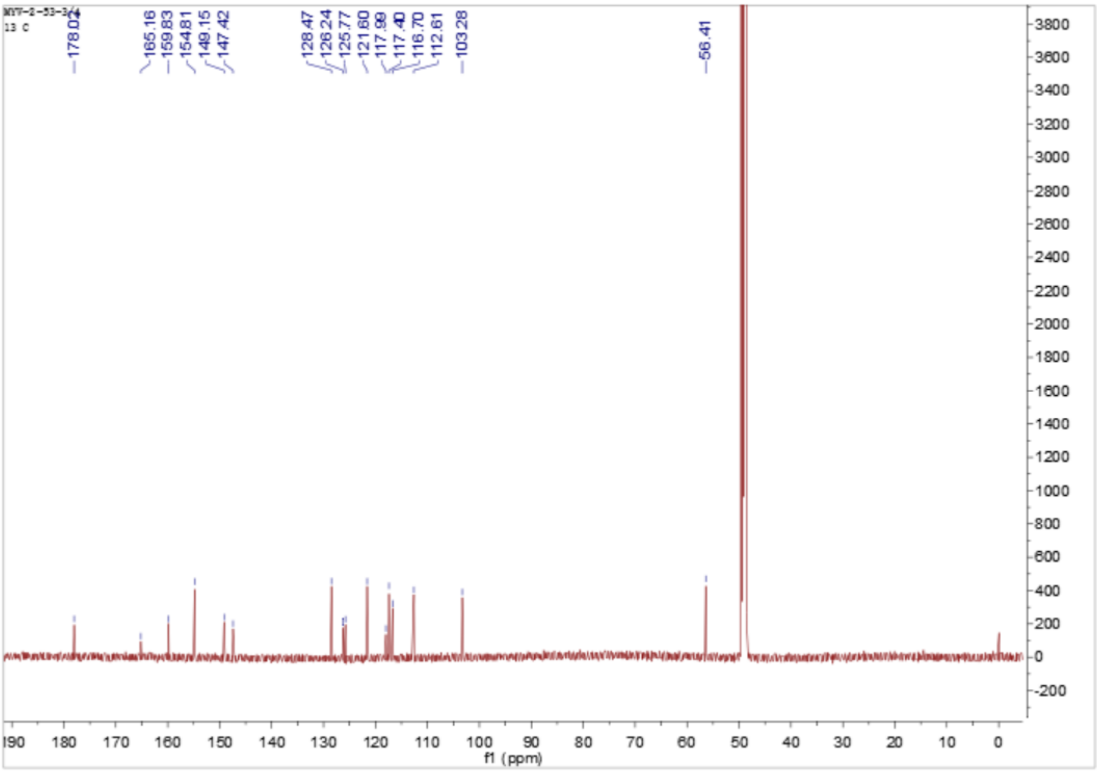

Figure S168. The ^{13}C NMR spectrum of 47 in CD_3OD

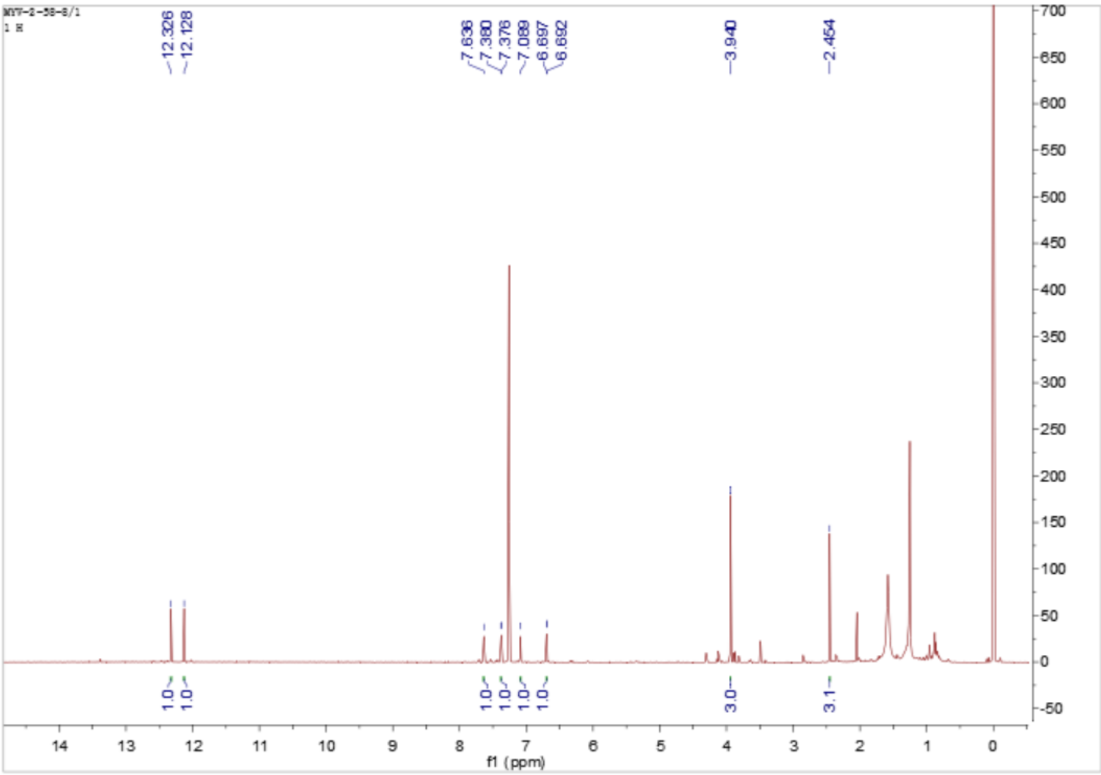

Figure S169. The ^{1}H NMR spectrum of 48 in $CDCl_3$

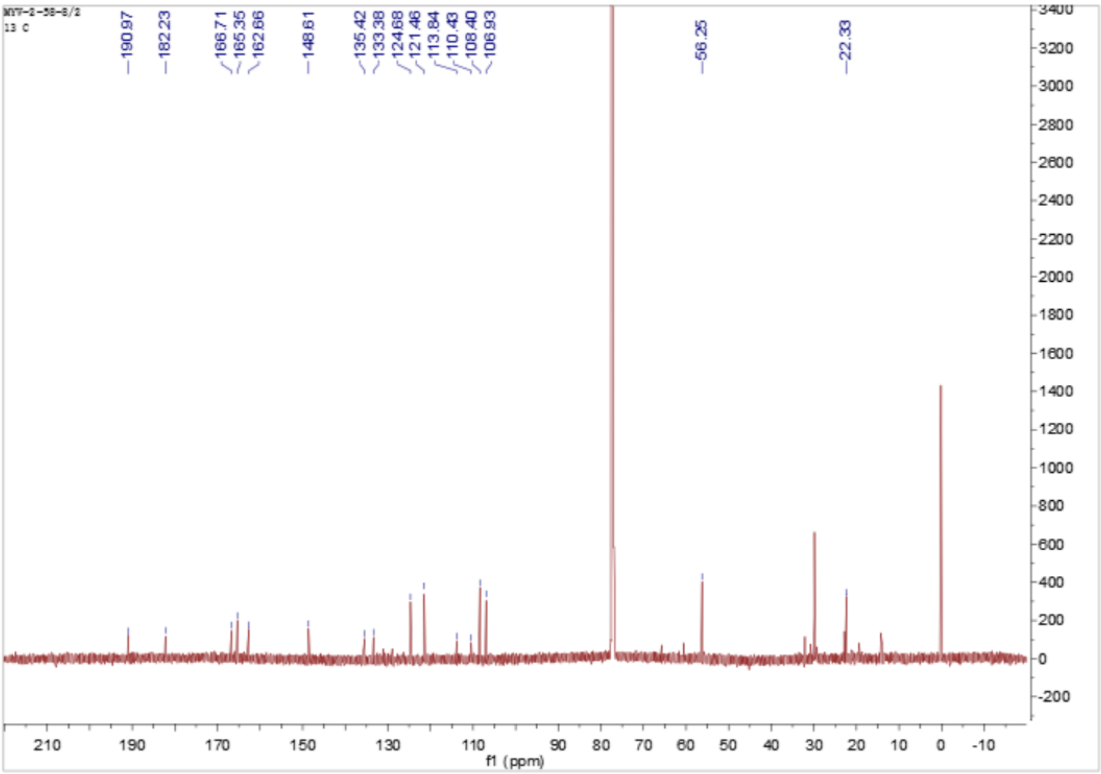

Figure S170. The ^{13}C NMR spectrum of 48 in $CDCl_3$

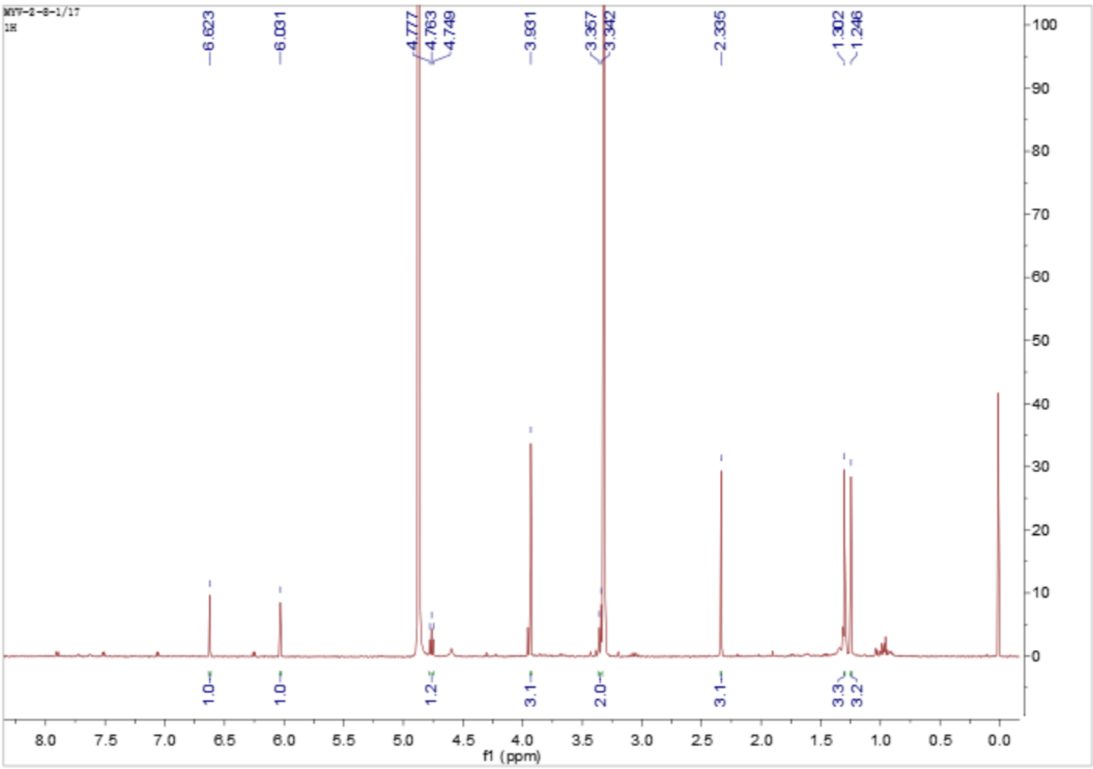

Figure S171. The ^{1}H NMR spectrum of 49 in CD_3OD

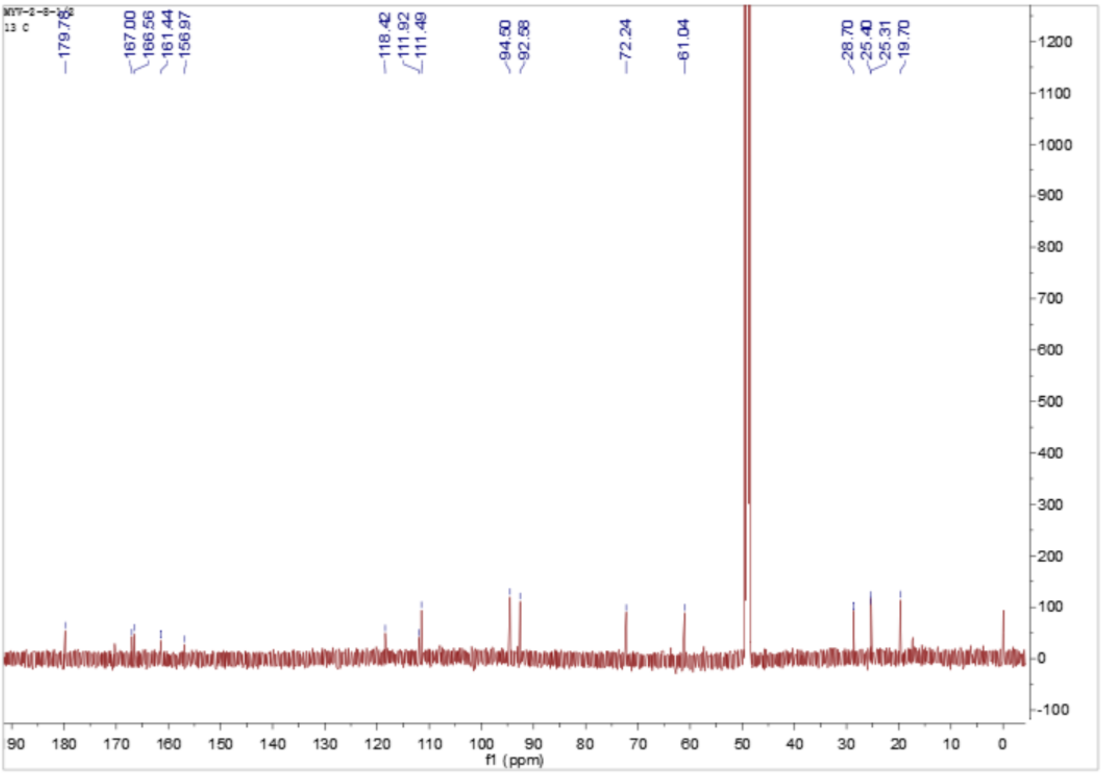

Figure S172. The ^{13}C NMR spectrum of 49 in CD_3OD

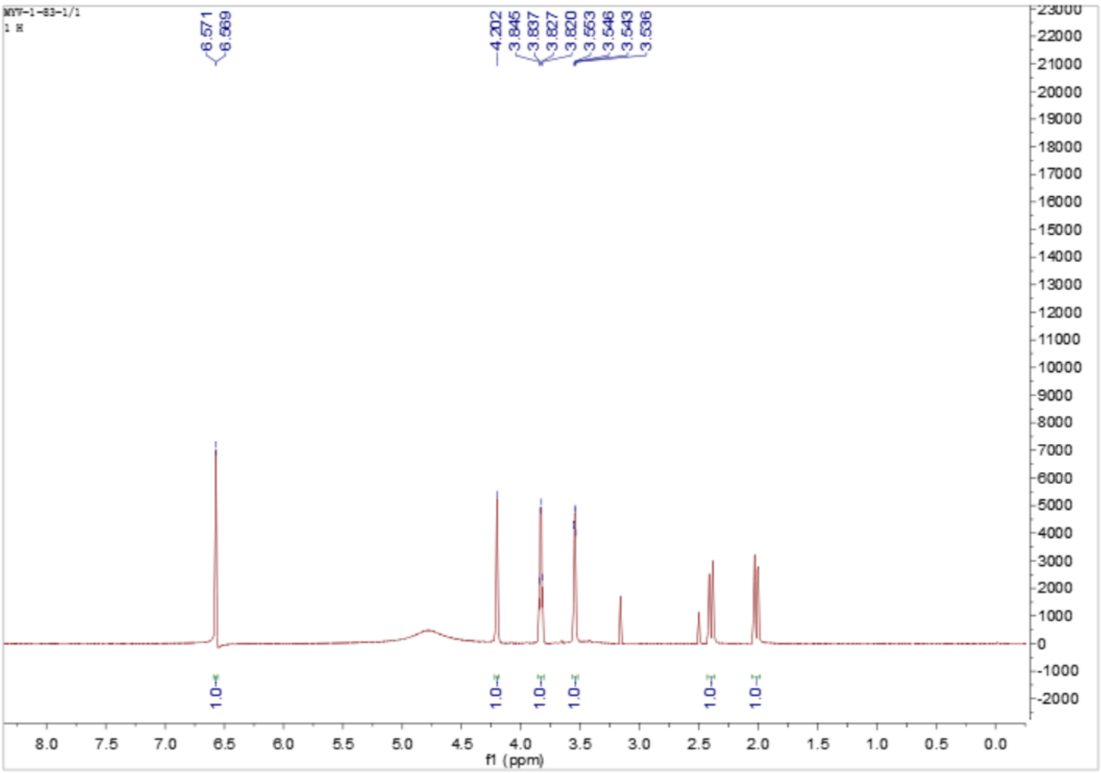

Figure S173. The ^{1}H NMR spectrum of 50 in DMSO-d_6

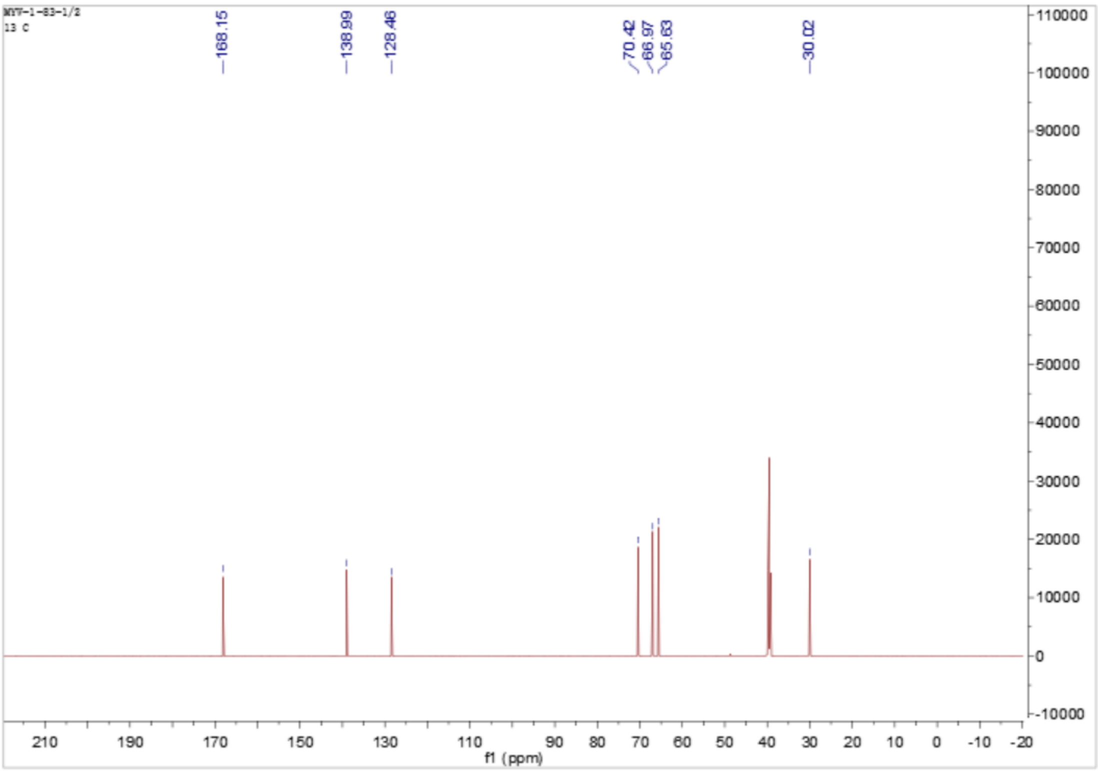

Figure S174. The ^{13}C NMR spectrum of 50 in DMSO-d_6

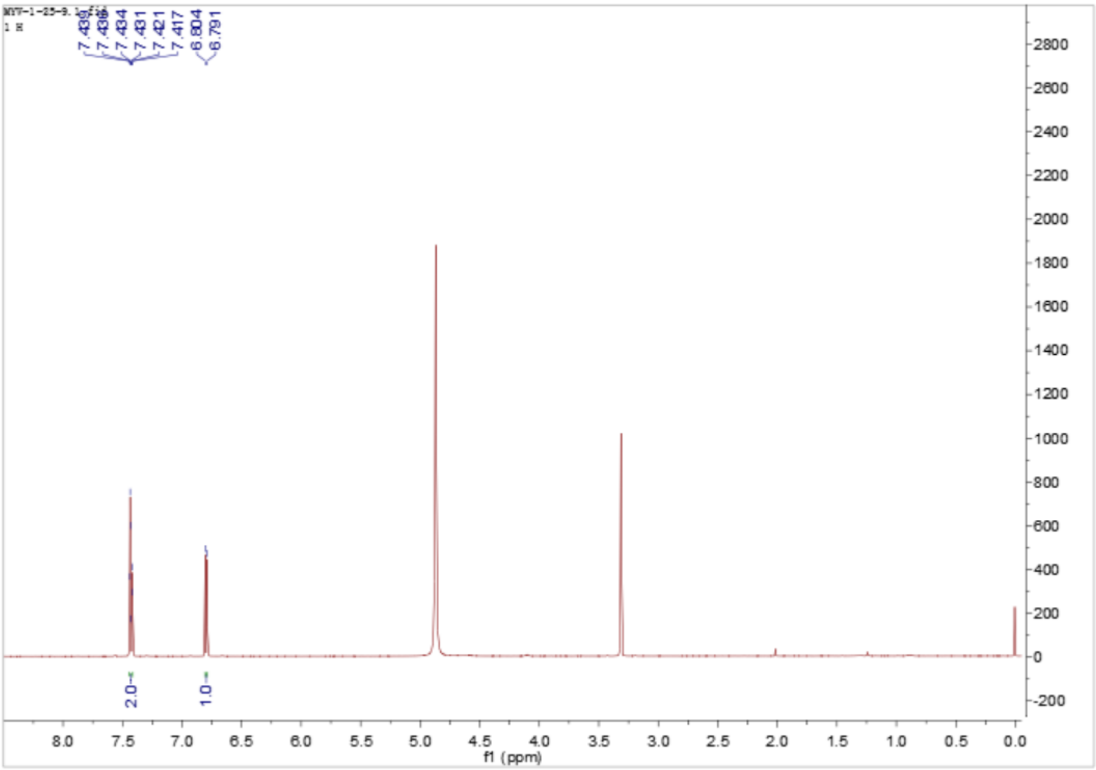

Figure S175. The ^{1}H NMR spectrum of 51 in CD_3OD

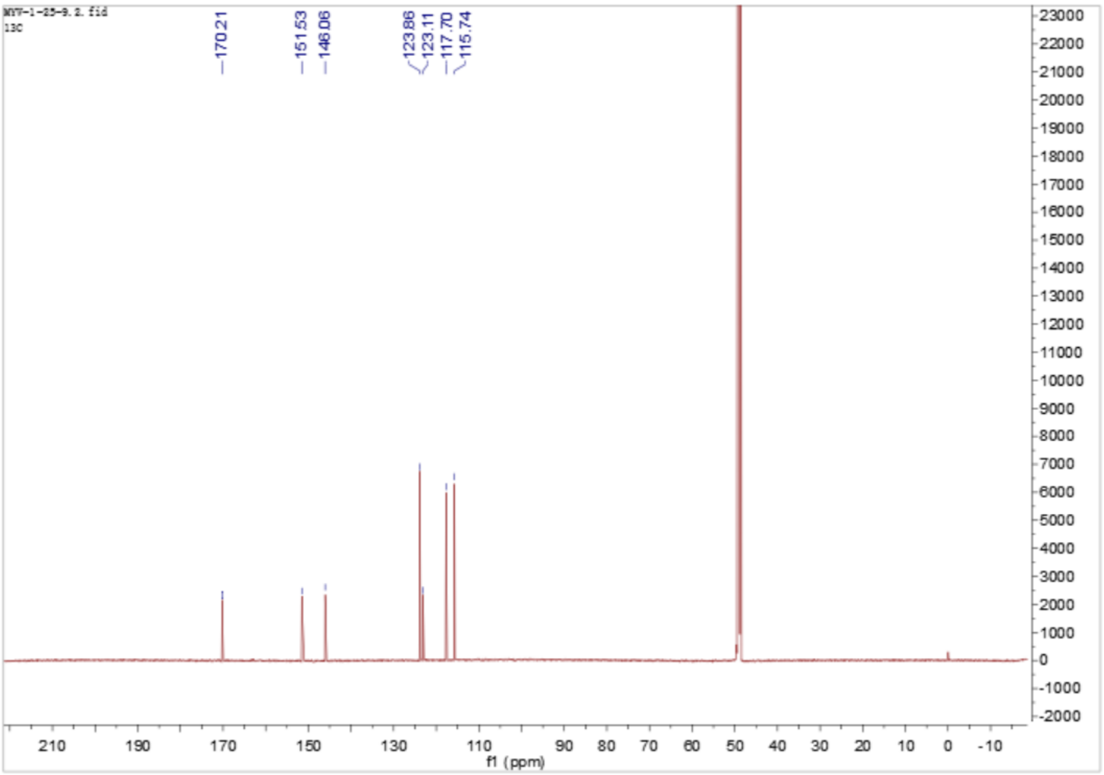

Figure S176. The ^{13}C NMR spectrum of 51 in CD_3OD
